# Elucidating and Optimizing the Photochemical Mechanism of Coumarin-Caged Tertiary Amines

**DOI:** 10.1021/jacs.4c03092

**Published:** 2024-07-18

**Authors:** Sambashiva Banala, Xiao-Tao Jin, Tanya L. Dilan, Shu-Hsien Sheu, David E. Clapham, Ryan M. Drenan, Luke D. Lavis

**Affiliations:** † Janelia Research Campus, Howard Hughes Medical Institute, Ashburn, Virginia 20147, United States; ‡ Department of Translational Neuroscience, Wake Forest University School of Medicine, Winston-Salem, North Carolina 27101, United States

## Abstract

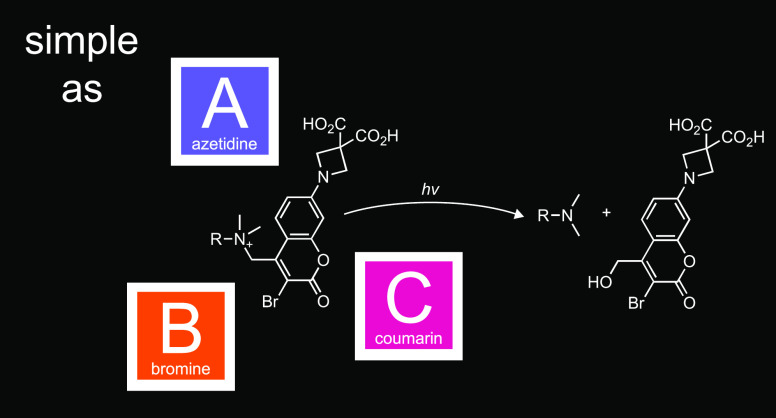

Photoactivatable or “caged” pharmacological agents combine the high spatiotemporal specificity of light application with the molecular specificity of drugs. A key factor in all optopharmacology experiments is the mechanism of uncaging, which dictates the photochemical quantum yield and determines the byproducts produced by the light-driven chemical reaction. In previous work, we demonstrated that coumarin-based photolabile groups could be used to cage tertiary amine drugs as quaternary ammonium salts. Although stable, water-soluble, and useful for experiments in brain tissue, these first-generation compounds exhibit relatively low uncaging quantum yield (Φ_u_ < 1%) and release the toxic byproduct formaldehyde upon photolysis. Here, we elucidate the photochemical mechanisms of coumarin-caged tertiary amines and then optimize the major pathway using chemical modification. We discovered that the combination of 3,3-dicarboxyazetidine and bromine substituents shift the mechanism of release to heterolysis, eliminating the formaldehyde byproduct and giving photolabile tertiary amine drugs with Φ_u_ > 20%—a 35-fold increase in uncaging efficiency. This new “ABC” cage allows synthesis of improved photoactivatable derivatives of escitalopram and nicotine along with a novel caged agonist of the oxytocin receptor.

## Introduction

Photoactivatable pharmacological agents (i.e., “caged” drugs) consist of biologically active agents where a key functionality on the molecule is appended with a photolabile group. Removal of the caging group using light restores biological activity, allowing precise temporal control over the location and concentration of active molecules within a complex biological environment.^1−4^ Classic caging strategies stem from organic chemistry protecting groups and typically involve attachment of the photolabile cage on reactive functionalities such as −OH, −NH_2_, or −CO_2_H. Many pharmacological agents lack these obvious sites for modification, however, which has limited the scope of photoactivatable compounds useful for biological investigation.

**Chart 1:**
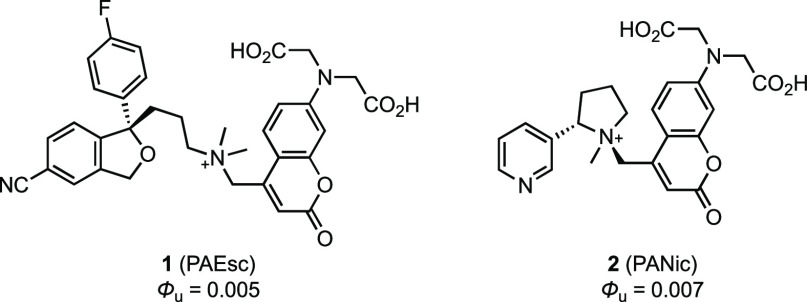
Structures of PAEsc (**1**) and PANic (**2**)

To extend the utility of caged compounds in biology, we recently demonstrated that the known 7-bis(carboxymethyl)aminocoumarin-4-yl-methyl (BCMACM)^5−7^ group could be used to cage pharmacological agents containing tertiary nitrogen atoms.^8^ Tertiary amino groups are a common motif in many drugs and are often critical for biological activity. In this general strategy, the nitrogen is alkylated with the BCMACM moiety to form a quaternary salt. The anionic centers on the BCMACM photolabile group decrease unwanted background activity of caged drugs prior to photolysis and ensure high aqueous solubility. Examples include photoactivatable (PA) derivatives of the selective serotonin reuptake inhibitor (SSRI) escitalopram (PAEsc, **1**) and the nicotinic acetylcholine receptor (nAChR) agonist nicotine (PANic, **2**; Chart 1).^8^

These first-generation compounds show excellent solubility, high extinction coefficients (ε > 14,000 M^–1^cm^–1^) and an absorption maximum (λ_max_) near 405 nm, matching the violet light sources typically used for uncaging molecules. Nevertheless, a major drawback of these compounds is a relatively low uncaging quantum yield (Φ_u_ < 1%).^8^ This poor photochemical efficiency necessitates longer irradiation times and/or application of higher intensity light. These issues limit the utility of the coumarin caged tertiary amines, especially for *in vivo* optopharmacology experiments that utilize microLEDs for photoactivation.^9^ We subsequently discovered that photolysis of BCMACM-caged tertiary amine compounds produced the reactive byproduct formaldehyde. This molecule is generally undesirable in living systems, leading to protein cross-linking^10^ and eventual toxicity. Here, we explore improvements to the BCMACM group by elucidating and then changing the photochemical reactions leading to uncaging. We discovered that the combination of a 3,3-dicarboxyazetidine functionality and a bromine group on the coumarin cage wholly shifts the photochemical mechanism to heterolysis, thereby improving performance. Compared to our first-generation caged escitalopram **1**, the novel 7-(3,3-dicarboxyazetidinyl)-3-bromocoumarin (“ABC”) cage yielded a photoactivatable tertiary amine compound with a >35-fold increase in Φ_u_ and also eliminated photogeneration of formaldehyde. This new photoactivatable group could be used to cage either the pyrrolidine nitrogen or the pyridine nitrogen on nicotine, generating second-generation PANic compounds for optopharmacology experiments in acute brain slices. The ABC cage was also useful in creating a photoactivatable oxytocin receptor agonist for use in living cells. These compounds will enable new biological experiments, and our comprehensive investigation of the photochemistry of coumarin cages will inform the rational design of other photoactivatable tools for biological research.

## Results and Discussion

### Determination of Uncaging Mechanisms of PAEsc

**Figure 1:**
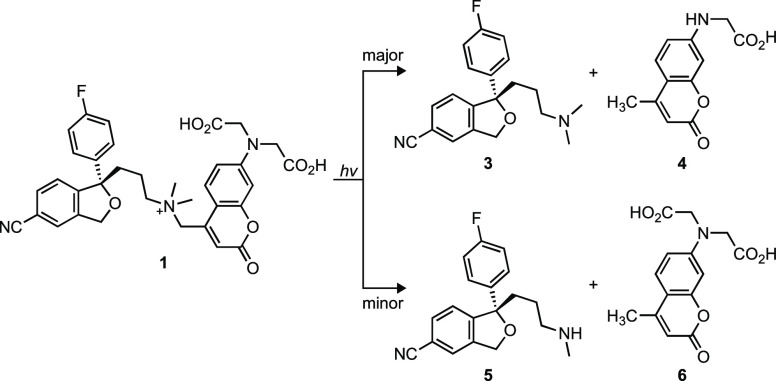
Photochemistry of PAEsc (**1**). Photolysis reaction of photoactivable escitalopram **1** generating escitalopram (**3**) and byproduct **4** along with minor products **5** and **6**.

We first considered the photochemistry of BCMACM-caged tertiary nitrogen compounds such as PAEsc (**1**). We determined the photolysis products of **1** in aqueous solution using tandem high performance liquid chromatography–mass spectrometry (LC–MS; Figure 1 and Figure S1). The major products are escitalopram (**3**) and monoalkylated coumarin **4**; these two are generated in an apparent 1:1 ratio. Uncaging of **1** also results in the minor products norescitalopram (**5**) and coumarin **6**; this pair of products is also generated in apparent equimolar amounts. This set of photoproducts—particularly the dealkylated compounds **4** and **5**—suggest uncaging mechanisms that involve radical intermediates. This result prompted a deeper investigation into the mechanism of uncaging of these coumarin-caged tertiary amines with the goals of improving Φ_u_ and eliminating dealkylation by modifying the structure of the caging group.

**Figure 2:**
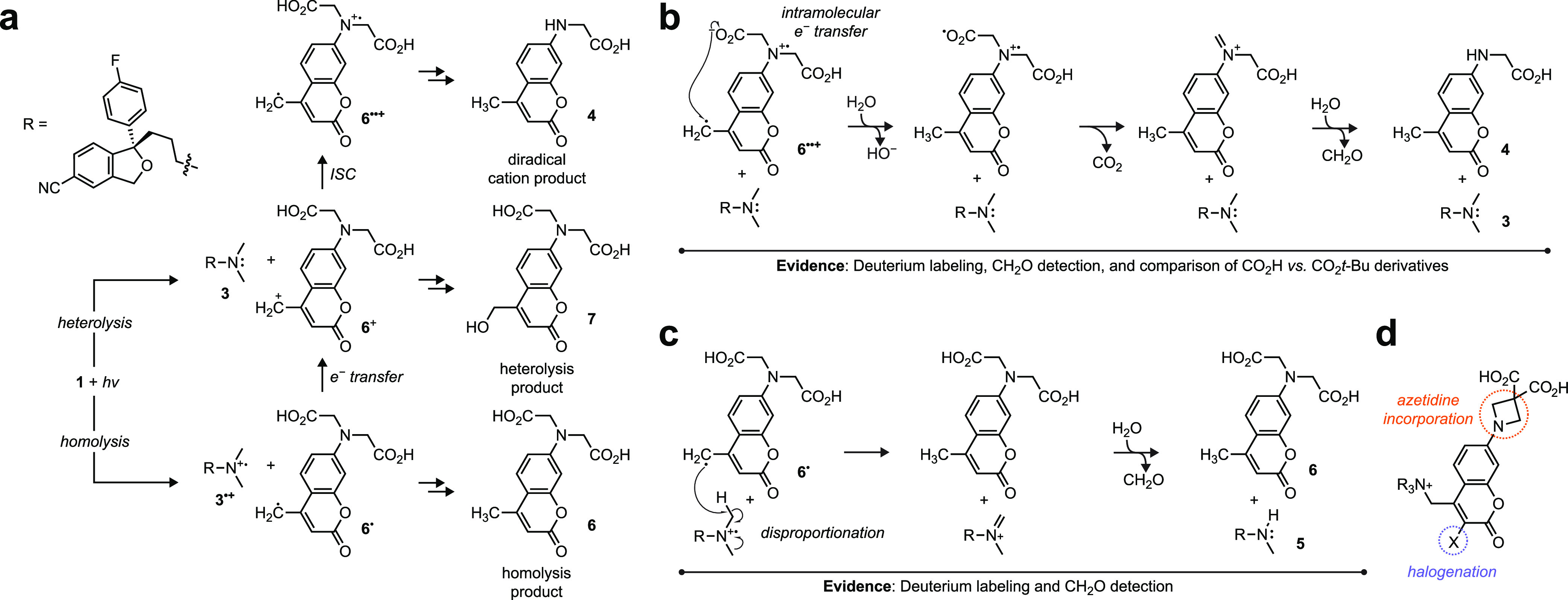
Proposed mechanism of BCMACM-caged tertiary amine compounds. (a) Initial intermediates of BCMACM-caged tertiary amine **1** after photolysis, generating **6**^**•**^ via homolysis or **6**^**+**^ via heterolysis and subsequent intersystem crossing to the diradical cation **6**^**••+**^, followed by generation of the homolysis product **6**, the heterolysis product **7**, and the diradical cation product **4**. (b) Proposed mechanism to generate major diradical cage product **4** and escitalopram (**3**). (c) Proposed mechanism to form minor cage product **6** and dealkylated norescitalopram (**5**). (d) Azetidine and halogen modifications to the BCMACM cage.

In the orthodox view of coumarin uncaging,^7,11−16^ excitation with light results in either homolysis or heterolysis (Figure 2a). Homolysis of compound **1** would yield the BCMACM radical **6**^**•**^ along with the amine radical cation of escitalopram (**3**^**•+**^). Heterolysis yields the BCMACM cation (**6**^**+**^) and the amine **3**, although it remains unclear if these species are generated directly from **1** or result through intermolecular electron transfer between homolysis products **6**^**•**^ and **3**^**•+**^. Although the first pathway can straightforwardly explain the production of minor product **6** (i.e., the “homolysis product”) it is difficult to account for the major dealkylated photoproduct **4** (Figure 1).

**Scheme 1:**
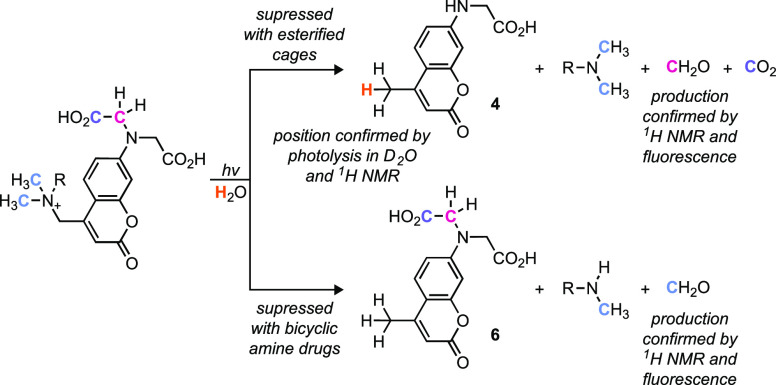
Summary of Support for the Proposed Photochemical Mechanism of BCMACM-Caged Tertiary Amine Compounds

We considered an emerging hypothesis in the coumarin caging field where a triplet diradical cation (**6**^**••+**^) species can result through intersystem crossing (ISC) from **6**^+^ due to the similar energy levels of the two cationic species (Figure 2a).^17−19^ This triplet diradical cation intermediate could lead to the dealkylated coumarin **4**, which we called the “diradical cation product”. Efficient ISC from **6**^+^ to **6**^**••+**^ would also explain why the hydroxymethyl **7** (i.e., the “heterolysis product”) is formed in only trace amounts (<3%). We then formulated possible mechanisms that produce compounds **4** and **6**. For major compound **4**, our proposed pathway (Figure 2b) involves intramolecular electron transfer in the triplet diradical cation (**6**^**••+**^) followed by protonation from solvent. One-electron oxidation of carboxylate groups is effected by relatively mild oxidants and can be followed by loss of CO_2_.^20^ Subsequent iminium hydrolysis generates the dealkylated cage along with formaldehyde. The minor product **6** could stem from the homolysis radical pair **6**^**•**^, and **3**^**•+**^, which undergoes disproportionation by H atom transfer^21^ within the solvent cage to yield **6** (Figure 2c). The resulting iminium species spontaneously hydrolyzes to yield norescitalopram (**5**) and formaldehyde. These mechanisms were supported by the following experiments: identifying the photolysis products of different BCMACM-caged pharmacological agents, performing the photolysis in D_2_O, examining the photolysis of esterified cages, and measuring formaldehyde production using a fluorescence-based assay and by ^1^H NMR (Scheme 1 and Figures S2–S9) as described below.

Photolysis of **1** in D_2_O results in 91% deuterium incorporation into **4** (i.e., **4-***d*_**1**_; Figures S2 and S3), which is consistent with the protonation of the cage by solvent after intramolecular electron transfer (Figure 2b). Deuterium is not incorporated into **6**, however, which fits the proposed intermolecular H^•^ transfer from the radical cation **3**^**•+**^ to **6**^**•**^ (Figure 2c). Photolysis of a 50 μM solution of **1** showed apparent equimolar production of formaldehyde (48 μM) detected using a commercial fluorescence assay kit (Figure S4), supporting formaldehyde formation from both the coumarin cage and the pharmacological agent (Figure 2b,c and Scheme 1). Production of **4-***d*_**1**_ in D_2_O was also observed with the photolysis of PANic (**2**) to yield nicotine (**8**; Figure S5) and a simpler photoactivatable analog, **9**, that releases *N*-methylpyrrolidine (**NMP**, **10**; Figure S6); deuterium incorporation was not observed in homolysis product **6** with either of these compounds. Release of formaldehyde from both **2** and **9** was detected by ^1^H NMR (Figures S5 and S6). Photolysis of a photoactivatable derivative of the bicyclic tertiary amine PNU-282,987 (PAPNU, **11**; 50 μM) resulted in generation of the pharmacological agent **12** along with nearly exclusive production of diradical cation product **4** (or **4-***d*_**1**_ in D_2_O) with no appreciable creation of **6** (Figure S7). Substantial production of formaldehyde was still observed (41 μM; Figure S4), however, even though this compound lacks an *N*-methyl group. These data again support intramolecular electron transfer followed by loss of CO_2_, ultimately resulting in cage dealkylation and formaldehyde formation (Figure 2b and Scheme 1). Finally, the diradical cation product analogous to **4** was not formed from photolysis of compounds containing esterified BCMACM cages including PAEsc-di(*t*-butyl ester) (**13**; Figure S8; 50 μM), and the di(*t*-butyl ester) of BCMACM-caged PNU-282,987 (**14**; 50 μM; Figure S9), showing that blocking the carboxylate moieties suppresses dealkylation of the caging group (Scheme 1). For esterified compound **13**, heterolysis product **15** and homolysis product **16** were formed in approximately equimolar amounts (Figure S8). Photolysis of esterified **13** showed appreciable formaldehyde production (25 μM; Figure S4), presumably due to dealkylation of the *N*-methyl group on the escitalopram pharmacological agent (Figure 2c); this is consistent with the formation of norescitalopram (**5**; Figure S8). Photolysis of bicyclic amine-containing compound **14** resulted in **15** as the main cage byproduct (Figure S9) and did not generate significant amounts of formaldehyde (<1 μM; Figure S4). Overall, these data show that free carboxylate groups are necessary to elicit cage dealkylation (Figure 2b) and acyclic *N*-alkyl groups on the pharmacological agent allow drug dealkylation (Figure 2c) when using the BCMACM group.

### Design and Synthesis of New Caged Escitalopram Compounds

Elucidation of the uncaging mechanism of **1** reveals the established BCMACM photolabile group is suboptimal for caging tertiary amines. The major photochemical pathway stems from the diradical cation species and releases reactive formaldehyde (Figure 2b and Scheme 1). The minor pathway results from homolysis and intermolecular H-transfer, which leads to dealkylation of compounds containing *N*-methylamino groups thereby releasing formaldehyde (Figure 2c and Scheme 1). We considered structural modifications of the coumarin cage that could modulate the photochemistry (Figure 2d). First, we envisioned replacing the iminodiacetic acid group in **1** with a 3,3-dicarboxyazetidine moiety. Our laboratory discovered that incorporation of azetidine can substantially increase the brightness and photostability of small-molecule fluorophores including coumarins,^22^ presumably due to the higher ionization potential of this moiety. We reasoned that incorporation of an azetidine functionality would destabilize the diradical cation intermediate and suppress dealkylation of the cage. Work from other laboratories showed that simple azetidinylcoumarin caged compounds show modestly improved Φ_u_ when caging carboxylic acid groups,^23^ but incorporation of substituted azetidines, such as 3,3-dicarboxylazetidine moieties, has not been explored. Second, we sought to introduce a halogen atom at the 3-positon of the coumarin cage, which has been shown to increase Φ_u_ when caging pyridines as pyridinium salts.^24^ Although both of these strategies independently improve the photochemical efficiency of coumarin-based cages, they have not been applied to the caging of tertiary nitrogen-containing compounds and have not been combined into a single photolabile group.

**Figure 3:**
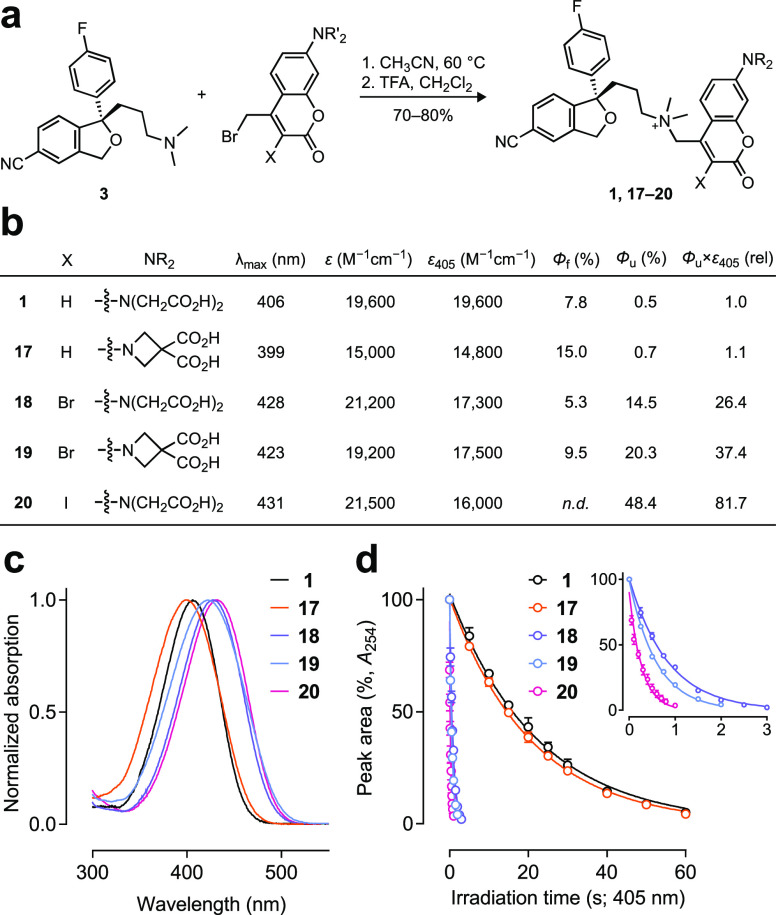
Synthesis and properties of photoactivatable escitalopram derivatives **1**, **17**–**20**. (a) Synthesis of photoactivable escitalopram compounds **1**, **17**–**20**. (b) Photophysical properties of **1**, **17**–**20**; *n.d.* indicates not determined. (c) Normalized absorption spectra of **1**, **17**–**20**. (d) Normalized HPLC chromatogram peak area vs irradiation time for compounds **1**, **17**–**20**; inset shows different irradiation time scale to highlight the higher photolysis rates of compounds **18**–**20**.

To test this idea, we synthesized analogs of caged escitalopram (**1**) where the coumarin cage contains a 3,3-dicarboxyazetidine moiety, a halogen substituent, or both modifications. Following our previous synthesis of **1**, we prepared **17**–**20** by reaction of different bromomethyl coumarin derivatives with **3** in CH_3_CN, followed by deprotection of the intermediate *t*-Bu esters with TFA (Figure 3a and Schemes S1 and S2). We then measured the properties of these new caged escitaloprams, comparing to **1**. Replacing the BCMACM cage in **1** with the 3,3-dicarboxyazetidinylcoumarin in **17** modestly increases the Φ_u_ from 0.48% to 0.69% (Figure 3b). This modification also elicits a small, 7 nm hypsochromic in absorption maximum (λ_max_; Figure 3c). Bromination of the BCMACM coumarin cage in **18** caused a modest bathochromic shift in λ_max_ (22 nm; Figure 3b,c) and a marked increase in Φ_u_ = 14.5% (Figure 3b,d). Combining the azetidine and bromine substitutions to yield compound **19** maintained the red-shifted λ_max_ and further improved Φ_u_ = 20.3%. Finally, incorporation of iodine at the 3-position of the BCMACM cage to yield **20** afforded an even longer λ_max_ and higher Φ_u_ = 48.4%. We also determined the fluorescence quantum yields (Φ_f_) of **1**,**17**–**19**, which showed the azetidine substitution doubles the Φ_f_; this is consistent with the behavior of analogous coumarin-based fluorescent labels.^22^ Bromine substitution substantially decreases Φ_f_, however, suggesting that incorporation of this atom increases intersystem crossing via the heavy atom effect.^25^ Finally, we measured the chemical stability of these compounds to assess utility in biological assays. Compounds **1** and **17** showed excellent stability in aqueous solution, showing only minimal hydrolysis after 12 h. The halogen atoms in compounds **18**–**20** destabilized the lactone functionality in the coumarin, resulting in modestly faster hydrolysis; this can be reversed in acidic conditions (Figure S10). These compounds are stable for years at −20 °C as solid or DMSO solutions; we recommend use of freshly made (<1 h) aqueous solutions prepared from solid or DMSO stocks to limit the hydrolysis of brominated coumarin compounds.

### Photochemistry of PAEsc Compounds

**Figure 4:**
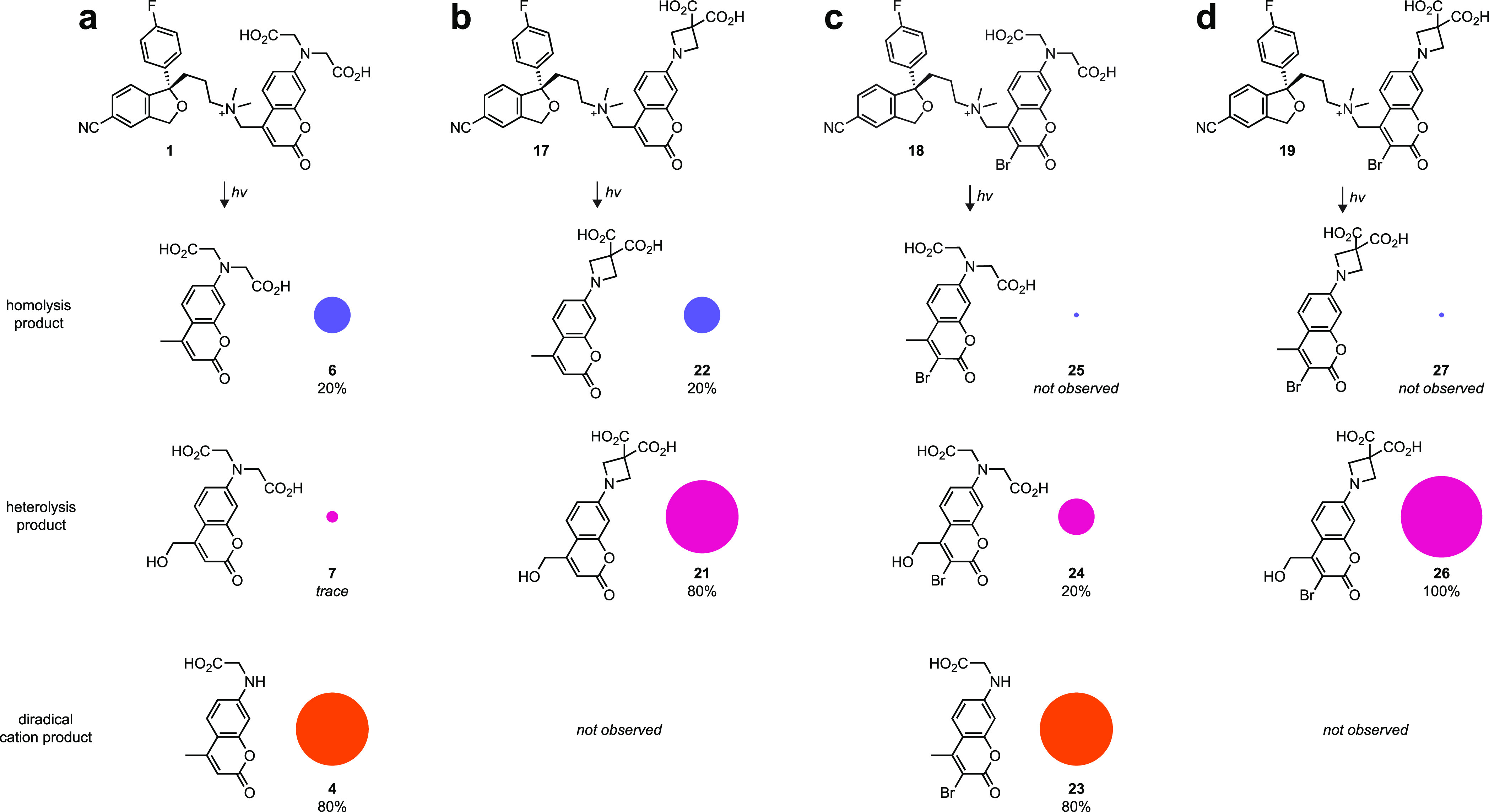
Photochemical outcome of caged tertiary amine compounds **1**, **17**–**20**. (a) Photochemical products of parent caged escitalopram **1** showing homolysis product **6**, the heterolysis product **7**, and the diradical cation product **4**. (b) Photochemical products of caged escitalopram **17** showing homolysis product **22** and the heterolysis product **21**. (c) Photochemical products of caged escitalopram **18** showing homolysis product **25**, the heterolysis product **24**, and the diradical cation product **23**. (d) Photochemical products of caged escitalopram **19** showing homolysis product **27** and the heterolysis product **26**. Area of circles is proportional to the relative yield of the photochemical products.

We then undertook a detailed examination of the photochemistry of caged escitalopram molecules **1**,**17**–**20** to determine if the structural modifications to the caging group modified the photochemistry. As before, we used LC–MS analysis and prepared authentic coumarin cage photoproducts as standards (Scheme S3). As mentioned above, photolysis of **1** yields coumarin byproducts **4** (∼80%) and **6** (∼20%; Figures 1 and 4a and Figure S1), stemming from the diradical cation **6**^**••+**^ and radical **6**^**•**^ intermediates, respectively (Figure 2). The heterolysis product **7** is produced in trace amounts (<3%). Incorporation of the azetidine into the caging group in **17** changes the photochemical mechanism, yielding mostly the hydroxymethyl derivative **21** (i.e., the heterolysis product; ∼80%). The production of the homolysis product coumarin **22** was maintained (∼20%) and a product corresponding to the diradical cation intermediate was not observed (Figure 4b and Figure S11). This result is consistent with our hypothesis that the higher ionization potential of the azetidine substituent would destabilize the diradical cation intermediate, leading to a switch in the major cage photoproduct. Overall, we find the change from iminodiacetic acid in **1** to 3,3-dicarboxyazetidine in **17**—an addition of just one carbon atom—substantially modifies the photochemical mechanism of uncaging.

Having established the effect of the azetidine substituent, we then examined the photochemistry of escitalopram compounds bearing halogenated coumarin cages (**18**–**20**). The 3-bromo-BCMACM-caged compound **18** showed different photochemistry with photolysis yielding the dealkylated **23** as the major product (∼80%; Figure 4c and Figure S12). This yield of dealkylated cage photoproduct is similar to that observed in the photoreaction of compound **1**, showing that bromination does not change the amount of diradical cation intermediate. This result is interesting since bromination does increase intersystem crossing in the excited state of the parent molecule based on the decrease in fluorescence quantum yield (Figure 3b). We surmise that the energy barrier between the cation singlet and diradical cation triplet is already low enough to allow facile interchange,^17^ making the addition of the heavy bromine atom superfluous. Unlike **1**, however, the minor product (∼20%) of photolysis of **18** is the hydroxymethyl heterolysis product **24**; the homolysis product **25** is not observed. This suggests the bromine substitution promotes either direct heterolysis or electron transfer to produce the coumarinylmethyl carbocation intermediate (Figure 2a). Based on the results from **17** and **18**, we hypothesized the azetidine and bromine substituents should complement each other by suppressing homolysis and diradical cation formation, thereby promoting the heterolysis product. True to this prediction, we observed that **19** gave the heterolysis product **26** as the near-exclusive cage derivative; neither the homolysis product **27** or a diradical cation-derived molecule were produced in appreciable amounts (Figure 4d and Figure S13). Finally, we evaluated the iodo-containing compound **20**, which produced the heterolysis products **28** and diradical cation product **29** (Figure S14). The homolysis product **30** was not observed, which was similar to the behavior of bromine-containing analog **18**. The heterolysis product was produced in higher amounts compared to **18**, however, which is consistent with the hypothesis that electron-withdrawing substituents at the 3-position of the coumarin cage promotes direct heterolysis and/or electron transfer to yield the cation species. Although **20** showed the highest Φ_u_, we did not investigate iodo-containing cages further due to small-but-significant (1–3%) photolysis during our standard HPLC purification and lyophilization protocol. We therefore focused on the 7-(3,3-dicarboxyazetidinyl)-3-bromocoumarin (“ABC”) cage found in **19**, which showed the best balance of photochemical efficiency in biological experiments and chemical stability (Figure S15).

### Next-Generation Photoactivatable Nicotine Derivatives

**Figure 5:**
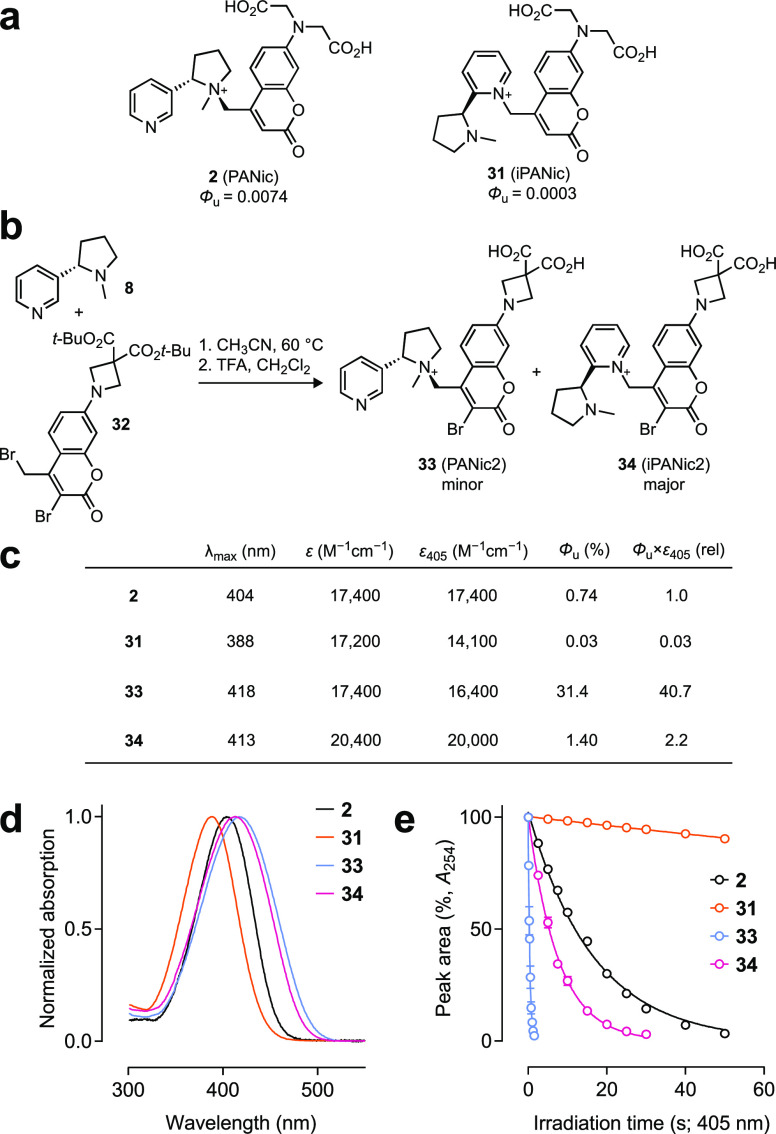
Synthesis and properties of photoactivatable nicotine derivatives. (a) Structures of the first-generation PANic (**2**) and isomer iPANic (**31**). (b) Synthesis of PANic2 (**33**) and iPANic2 (**34**). (c) Photophysical properties of **2**, **31**, **33**, and **34**. (d) Normalized absorption spectra of **2**, **31**, **33**, and **34**. (e) Normalized HPLC chromatogram peak area vs irradiation time for compounds **2**, **31**, **33**, and **34**.

Having established the desirable properties of the ABC cage using escitalopram, we then considered photoactivatable nicotine (PANic) derivatives. The first-generation PANic (**2**; Chart 1 and Figure 5a) has enabled a variety of studies on nAChRs including nicotine mediated upregulation of receptors, mapping functional nAChRs at the subcellular level, and measuring nicotine-induced calcium signaling.^8,26,27^ PANic is not without drawbacks, however, including a relatively low uncaging efficiency (Φ_u_ < 1%), generation of formaldehyde (Figure S5), and a low-yielding synthesis. The nicotine structure consists of a pyridine attached to an *N*-methylpyrrolidine; reaction with a bromomethyl caging group results in the formation of three products. The desired PANic molecule **2** is the minor product along with its diastereomer. The major product is the isomeric pyridinium compound (iPANic; Figure 5a) **31**, which exhibits an extremely low Φ_u_= 0.03%.^8^

Given these issues with the original PANic (**2**), we used the improved ABC cage to prepare new photoactivatable nicotine compounds. Reaction of nicotine (**8**) with ABC cage precursor **32** afforded “PANic2” (**33**) and the isomer “iPANic2” (**34**; Figure 5b and Scheme S4). Evaluation of the photochemical properties of **2** and **33** revealed the improvements observed for the ABC-caged escitalopram **19** were generalizable to caged nicotines (Figure 5c–e). PANic2 (**33**) showed a >40-fold higher Φ_u_ compared to **2** along with a 14 nm bathochromic shift in λ_max_. The pyridinium iPANic2 (**34**) compound also showed an increase in Φ_u_ = 1.4% compared to compound iPANic (**31**) with a λ_max_ = 413 nm. These properties make iPANic2 superior to the original **2** and is consistent with other reports showing efficient photolysis of pyridines caged with 3-bromocoumarins.^24^ We further verified this improvement by preparing a photoactivatable derivative of the pyridine-containing voltage-dependent sodium channel blocker A 887626 (**35**; Scheme S5);^28^ use of the ABC cage yields a pyridinium compound with comparable properties to iPANic2 (Figure S16).

**Figure 6:**
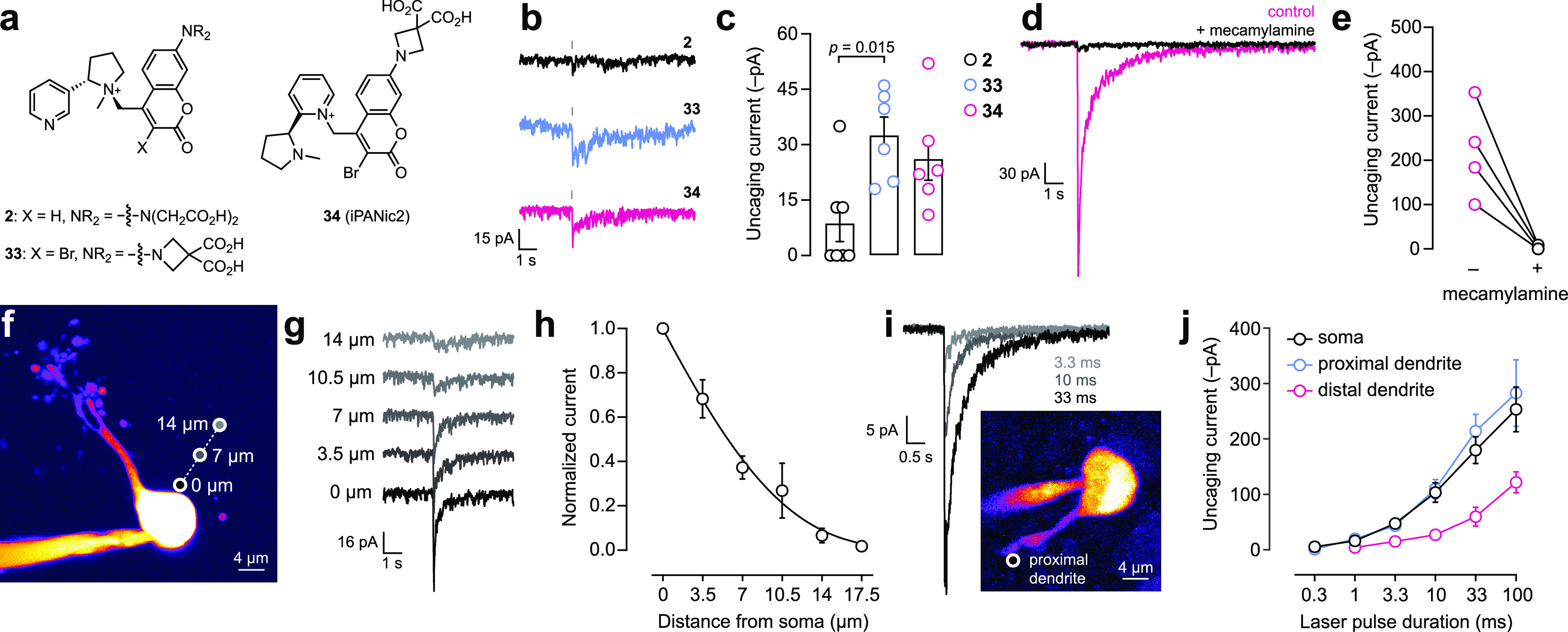
Biological utility of photoactivatable nicotine derivatives. (a) Structures of first- and second-generation PANic compounds. (b) Representative traces of light-evoked currents for compounds **2**, **33**, and **34** (50 μM) uncaged with 1 ms, 1-photon pulse from a single neuron. (c) Summary of 1-photon stimulation uncaging currents for compounds **2**, **33**, and **34** (50 μM). (d) iPANic2 (**34**; 50 μM) voltage clamp responses are antagonized by nAChR antagonist mecamylamine. A representative 10 ms, 1-photon uncaging response of iPANic2 (**34**; 50 μM) before and after mecamylamine application. (e) Before/after scatter plot for all recorded cells using conditions described for panel (d). (f) Representative 2-photon laser scanning microscopy (2PLSM) image of a medial habenula neuron, marked with uncaging positions. (g) Representative 10 ms pulse, 1-photon uncaging currents of signals in panel (f) from different distances from the soma using iPANic2 (**34**; 50 μM). (h) Summary of spatial resolution data of panels (f, g) for iPANic2. Normalized currents are plotted as a function of distance from the soma membrane. (i) Subcellular receptor mapping with iPANic2 (**34**; 50 μM). Representative proximal dendrite uncaging currents are shown for the indicated laser pulse durations. A representative 2PLSM image of a MHb neuron illustrates the proximal dendrite location. (j) Summary of receptor mapping data in panel (i). Uncaging current amplitude is shown for responses at the soma, proximal dendrite, and distal dendrite at the indicated laser pulse duration.

We then evaluated the two new photoactivatable nicotine compounds **33** and **34** in biological experiments, comparing to the original PANic (**2**, Figure 6a). Compounds **2**, **33** and **34** were applied to acute brain slices and activated using pulses of 405 nm light with concurrent electrophysiological measurements. The original PANic (**2**) was unable to evoke a substantial nAChR current with a 1 ms light pulse whereas application of either PANic2 (**33**) or iPANic2 (**34**) showed measurable responses under identical conditions (Figure 6b,c). Considering that iPANic2 (**34**) evoked biological responses similar to those elicited by PANic2 (**33**), combined with the higher synthesis yield of this compound (Figure 5b), we focused our characterization on this compound. Measuring light-activated currents in the presence and absence of the noncompetitive antagonist mecamylamine confirmed that the current induced by photolysis of iPANic2 was mediated by nAChRs (Figure 6d,e). We uncaged iPANic2 using 405 nm light at different distances from the soma (Figure 6f). We observed a strong distance dependence on evoked current (Figure 6g,h) indicating that one-photon activation affords excellent spatial resolution in brain slices. Like the original PANic (**2**), iPANic2 (**34**) was not an nAChR antagonist prior to uncaging (Figure S17). Finally, we found that compound **34** could be used to activate functional nAChR on the soma and dendrites of neurons in brain slices (Figure 6i,j). Overall, these results suggest that PANic2 (**33**) and iPANic2 (**34**) are superior replacements for PANic (**2**) and should allow the study of light-induced currents in neurons using shorter irradiation times or lower intensity light.

### Photoactivatable Oxytocin Receptor Agonists

**Figure 7:**
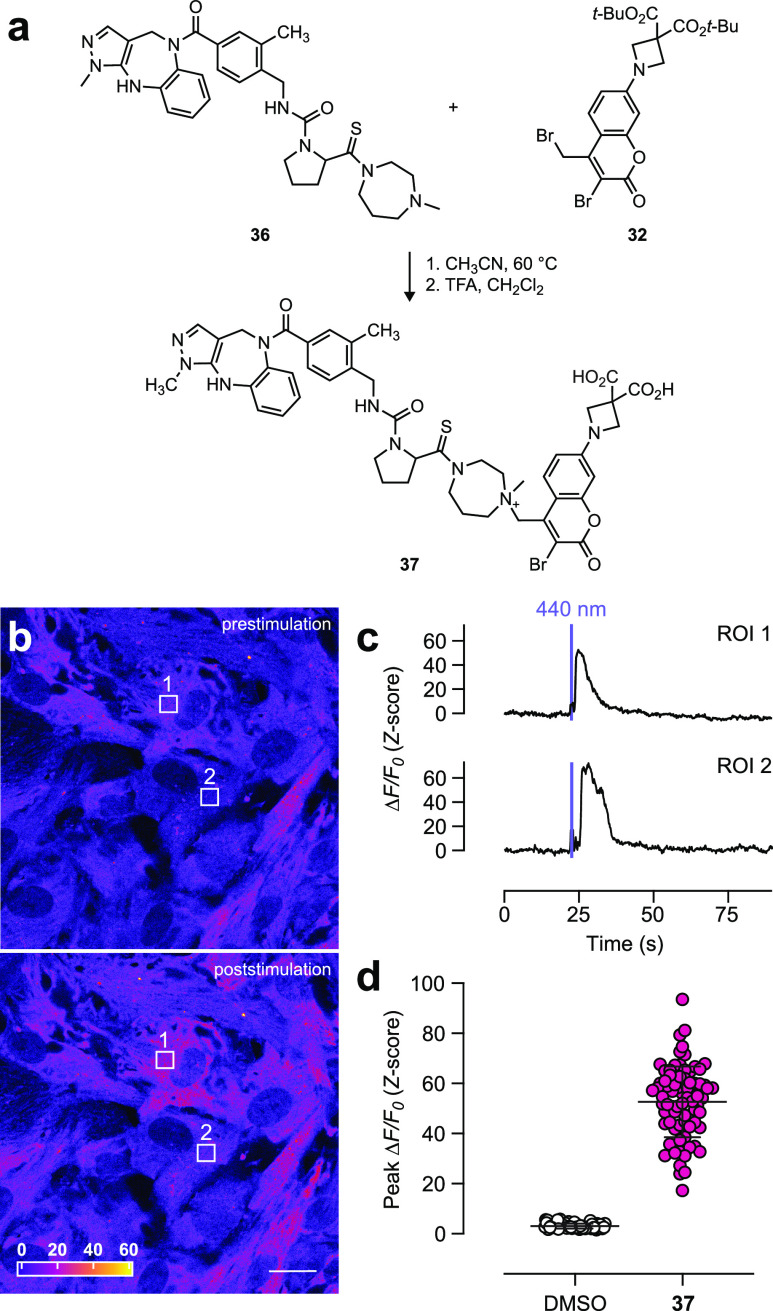
Synthesis and utility of ABC-caged oxytocin agonist. (a) Synthesis of PATCOT (**37**). (b) Pseudocolor fluorescence microscopy images of RPE-hTERT cells expressing HaloTag protein, loaded with BAPTA-JF_549_–HaloTag ligand AM ester,^35^ and incubated with **37** before (top) and after (bottom) application of uncaging light (λ = 440 nm, 1.48 mW/cm^2^, 1 s); scale bar: 20 μm. (c) Change in fluorescence over basal fluorescence (Δ*F*/*F*_0_; *Z*-score) vs time from the regions of interest (ROIs) denoted in panel (b) showing changes in fluorescence in response to the light pulse. (d) Plot of Δ*F*/*F*_0_ (*Z*-score) for cell incubated with DMSO (*n* = 72 ROIs) or **37** (*n* = 79 ROIs); error bars indicate mean ± SD.

We then extended the utility of the new ABC cage beyond escitalopram and nicotine to prepare photoactivatable reagents to modulate the activity of oxytocin receptors. Oxytocin is a peptide hormone involved in a wide variety of physiological responses;^29^ oxytocin receptors (OXTRs) are found across the body in many different tissues. We focused on the awkwardly named pharmacological agent “TC OT 39” (**36**; Figure 7a), which is a potent nonpeptide oxytocin receptor partial agonist and bears a tertiary amine group as part of the terminal 1,4-diazepane functionality.^30,31^ Per the synthesis of **19** (Figure 3) and **33** (Figure 5) compound **36** reacted with ABC-Br (**32**) in CH_3_CN to yield “PATCOT” (**37**; Figure 7a and Scheme S6). Compound **37** showed Φ_u_ = 5.9% and λ_max_ = 426 nm. Similar to the matched pairs of BCMACM/ABC-caged compounds **1**/**19** (Figure 3) and **2**/**33** (Figure 5), PATCOT (**37**) showed a 10-fold higher Φ_u_ and ∼18 nm longer λ_max_ than the BCMACM-caged analog **38** (Scheme S6 and Figure S18). We then assessed the utility of this compound in biological experiments using cultured human telomerase reverse transcriptase (hTERT) immortalized retinal pigment epithelial (RPE-1) cells that exhibit high expression of oxytocin receptors.^32^ Stimulation of the oxytocin receptor increases intracellular calcium due to Gα_q_-mediated calcium ion release from the endoplasmic reticulum in uterine myometrial smooth muscle cells.^33^ OXTR may couple to other G protein subtypes with different biological end points in other cell types.^34^ To measure transient changes in cytosolic Ca^2+^ concentration, we expressed the HaloTag protein in the cytosol of the RPE-1 cells and used this labeling system to localize BAPTA-JF_549_–HaloTag ligand, a small-molecule calcium indicator that is excited with green light.^35^ Photolysis of **37** using 440 nm light elicited robust Ca^2+^ responses in cells close to the area of illumination (Figure 7b,c). These signals were significantly higher than control experiments that lacked the photoactivatable pharmacological agent in the media (Figure 7d and Figure S18). This result demonstrates the generality of the ABC cage beyond escitalopram and nicotine. PATCOT also represents a new, nonpeptidic member of a limited collection of photoactivatable tools for modulating oxytocin receptors.^36^

## Conclusions

Caged compounds are important tools for biological research, allowing release of biologically active molecules with high temporal and spatial control. Here, we investigated the photochemistry of BCMACM-caged tertiary amines where the photoproducts pointed to radical intermediates (Figure 1). We rationalized this chemistry thanks to the recently proposed^17^ and confirmed^19^ diradical cation intermediate and formulated two pathways for photolysis of BCMACM-caged amines (Figure 2). In addition to a low Φ_u_, we discovered our first-generation photoactivable compounds generated formaldehyde upon photolysis. This unwanted photochemical outcome was driven by both the formation of the diradical cation intermediate and the homolysis pathway (Figure 2).

To remedy this problem, we investigated two substitutions on the coumarin and assembled and evaluated a comprehensive set of photoactivable escitaloprams (Figure 3). To prevent formation of the diradical cation, we replaced the iminodiacetic acid moiety with a 3,3-dicarboxyazetidine group, relying on the higher ionization potential of the four-membered ring system to stop electron transfer. We also installed a bromine at the 3-position of the coumarin, which stymies the homolysis pathway. These substitutions worked in concert to fully shift the photochemical mechanism to heterolysis (Figure 4), while also increasing photochemical quantum yield by 35-fold. This new ABC cage could be applied to other molecules to make improved, second-generation photoactivatable nicotine derivatives (Figures 5 and 6) and a new photoactivable agonist of the oxytocin receptor (Figure 7).

Looking forward, we expect the new ABC cage to be useful for the preparation of other chemical tools for biology. This photolabile group can be applied to cage other functional groups in disparate molecules. The reversible hydrolysis of the system (Figure S10) could be exploited to yield molecular AND logic gates that are activated by the combination of light and low pH environments. More generally, our work highlights the importance of careful investigation of the photochemical reactions used in biological experiments. Although the photochemistry of coumarin caging groups has been studied for decades, extension of this strategy to new types of molecules can bring surprises. Caging tertiary amines with coumarins resulted in complicated photochemistry with unwanted generation of formaldehyde from two different sources (Figure 2). Understanding the photochemistry allowed rational design of a new cage where the addition of just two atoms—C and Br—altered the photochemical mechanism. The development of new molecular tools for biology will undoubtably uncover more photochemical mysteries that can be probed and solved using chemistry.

## Supporting Information Available

Supplementary figures and schemes, experimental details, and characterization for all new compounds (PDF)

## ABBREVIATIONS

BCMACM: 7-bis(carboxymethyl)aminocoumarin-4-yl-methyl
PA: photoactivatable
SSRI: selective serotonin reuptake inhibitor
nAChR: nicotinic acetylcholine receptor
ABC: 7-(3,3-dicarboxyazetidinyl)-3-bromocoumarin
LC–MS: tandem high performance liquid chromatography–mass spectrometry
NMR: nuclear magnetic resonance
TFA: trifluoroacetic acid

## References

[ref1] Ellis-Davies G. C. Caged compounds: Photorelease technology for control of cellular chemistry and physiology. Nat. Methods 2007, 4, 619–628. 10.1038/nmeth1072. 17664946 PMC4207253

[ref2] Brieke C.; Rohrbach F.; Gottschalk A.; Mayer G.; Heckel A. Light-controlled tools. Angew. Chem., Int. Ed. 2012, 51, 8446–8476. 10.1002/anie.201202134. 22829531

[ref3] Ellis-Davies G. C. R. Useful caged compounds for cell physiology. Acc. Chem. Res. 2020, 53, 1593–1604. 10.1021/acs.accounts.0c00292. 32692149 PMC7814516

[ref4] Ellis-Davies G. C. R. Reverse engineering caged compounds: Design principles for their application in biology. Angew. Chem., Int. Ed. 2023, 62, e202206083 10.1002/anie.202206083. PMC10015297 36646644

[ref5] Senda N.; Momotake A.; Nishimura Y.; Arai T. Synthesis and photochemical properties of a new water-soluble coumarin, designed as a chromophore for highly water-soluble and photolabile protecting group. Bull. Chem. Soc. Jpn. 2006, 79, 1753–1757. 10.1246/bcsj.79.1753.

[ref6] Senda N.; Momotake A.; Arai T. Synthesis and photocleavage of 7-[[Bis(carboxymethyl)amino]coumarin-4-yl]methyl-caged neurotransmitters. Bull. Chem. Soc. Jpn. 2007, 80, 2384–2388. 10.1246/bcsj.80.2384.

[ref7] Hagen V.; Dekowski B.; Kotzur N.; Lechler R.; Wiesner B.; Briand B.; Beyermann M. 7-[Bis(carboxymethyl)amino]coumarin-4-ylmethoxycarbonyl derivatives for photorelease of carboxylic acids, alcohols/phenols, thioalcohols/thiophenols, and amines. Chemistry 2008, 14, 1621–1627. 10.1002/chem.200701142. 18046693

[ref8] Banala S.; Arvin M. C.; Bannon N. M.; Jin X. T.; Macklin J. J.; Wang Y.; Peng C.; Zhao G.; Marshall J. J.; Gee K. R.; Wokosin D. L.; Kim V. J.; McIntosh J. M.; Contractor A.; Lester H. A.; Kozorovitskiy Y.; Drenan R. M.; Lavis L. D. Photoactivatable drugs for nicotinic optopharmacology. Nat. Methods 2018, 15, 347–350. 10.1038/nmeth.4637. 29578537 PMC5923430

[ref9] Zhang Y.; Castro D. C.; Han Y.; Wu Y.; Guo H.; Weng Z.; Xue Y.; Ausra J.; Wang X.; Li R.; Wu G.; Vazquez-Guardado A.; Xie Y.; Xie Z.; Ostojich D.; Peng D.; Sun R.; Wang B.; Yu Y.; Leshock J. P.; Qu S.; Su C. J.; Shen W.; Hang T.; Banks A.; Huang Y.; Radulovic J.; Gutruf P.; Bruchas M. R.; Rogers J. A. Battery-free, lightweight, injectable microsystem for in vivo wireless pharmacology and optogenetics. Proc. Natl. Acad. Sci. U. S. A. 2019, 116, 21427–21437. 10.1073/pnas.1909850116. 31601737 PMC6815115

[ref10] Ai L.; Tan T.; Tang Y.; Yang J.; Cui D.; Wang R.; Wang A.; Fei X.; Di Y.; Wang X.; Yu Y.; Zhao S.; Wang W.; Bai S.; Yang X.; He R.; Lin W.; Han H.; Cai X.; Tong Z. Endogenous formaldehyde is a memory-related molecule in mice and humans. Commun. Biol. 2019, 2, 446 10.1038/s42003-019-0694-x. 31815201 PMC6884489

[ref11] Eckardt T.; Hagen V.; Schade B.; Schmidt R.; Schweitzer C.; Bendig J. Deactivation behavior and excited-state properties of (coumarin-4-yl)methyl derivatives. 2. Photocleavage of selected (coumarin-4-yl)methyl-caged adenosine cyclic 3′,5′-monophosphates with fluorescence enhancement. J. Org. Chem. 2002, 67, 703–710. 10.1021/jo010692p. 11856009

[ref12] Gandioso A.; Cano M.; Massaguer A.; Marchan V. A green light-triggerable RGD peptide for photocontrolled targeted drug delivery: Synthesis and photolysis studies. J. Org. Chem. 2016, 81, 11556–11564. 10.1021/acs.joc.6b02415. 27934458

[ref13] Geissler D.; Antonenko Y. N.; Schmidt R.; Keller S.; Krylova O. O.; Wiesner B.; Bendig J.; Pohl P.; Hagen V. (Coumarin-4-yl)methyl esters as highly efficient, ultrafast phototriggers for protons and their application to acidifying membrane surfaces. Angew. Chem., Int. Ed. 2005, 44, 1195–1198. 10.1002/anie.200461567. 15696594

[ref14] Schaal J.; Dekowski B.; Wiesner B.; Eichhorst J.; Marter K.; Vargas C.; Keller S.; Eremina N.; Barth A.; Baumann A.; Eisenhardt D.; Hagen V. Coumarin-based octopamine phototriggers and their effects on an insect octopamine receptor. ChemBioChem. 2012, 13, 1458–1464. 10.1002/cbic.201200110. 22674503

[ref15] Shembekar V. R.; Chen Y.; Carpenter B. K.; Hess G. P. Coumarin-caged glycine that can be photolyzed within 3 microseconds by visible light. Biochemistry 2007, 46, 5479–5484. 10.1021/bi700280e. 17425336

[ref16] Klan P.; Solomek T.; Bochet C. G.; Blanc A.; Givens R.; Rubina M.; Popik V.; Kostikov A.; Wirz J. Photoremovable protecting groups in chemistry and biology: Reaction mechanisms and efficacy. Chem. Rev. 2013, 113, 119–191. 10.1021/cr300177k. 23256727 PMC3557858

[ref17] Albright T. R.; Winter A. H. A fine line separates carbocations from diradical ions in donor-unconjugated cations. J. Am. Chem. Soc. 2015, 137, 3402–3410. 10.1021/jacs.5b00707. 25702699

[ref18] Kamatham N.; Da Silva J. P.; Givens R. S.; Ramamurthy V. Melding caged compounds with supramolecular containers: Photogeneration and miscreant behavior of the coumarylmethyl carbocation. Org. Lett. 2017, 19, 3588–3591. 10.1021/acs.orglett.7b01572. 28631486

[ref19] Takano M. A.; Abe M. Photoreaction of 4-(bromomethyl)-7-(diethylamino)coumarin: Generation of a radical and cation triplet diradical during the C-Br bond cleavage. Org. Lett. 2022, 24, 2804–2808. 10.1021/acs.orglett.2c00694. 35394291

[ref20] Zelechonok Y.; Silverman R. B. Silver(I)/peroxydisulfate-induced oxidative decarboxylation of amino acids. A chemical model for a possible intermediate in the monoamine oxidase-catalyzed oxidation of amines. J. Org. Chem. 1992, 57, 5787–5790. 10.1021/jo00047a045.

[ref21] Ratcliff M. A.; Kochi J. K. Solvolytic and radical processes in the photolysis of benzylammonium salts. J. Org. Chem. 1971, 36, 3112–3120. 10.1021/jo00820a010.

[ref22] Grimm J. B.; English B. P.; Chen J.; Slaughter J. P.; Zhang Z.; Revyakin A.; Patel R.; Macklin J. J.; Normanno D.; Singer R. H.; Lionnet T.; Lavis L. D. A general method to improve fluorophores for live-cell and single-molecule microscopy. Nat. Methods 2015, 12, 244–250. 10.1038/nmeth.3256. 25599551 PMC4344395

[ref23] Bassolino G.; Nancoz C.; Thiel Z.; Bois E.; Vauthey E.; Rivera-Fuentes P. Photolabile coumarins with improved efficiency through azetidinyl substitution. Chem. Sci. 2018, 9, 387–391. 10.1039/C7SC03627B. 29629108 PMC5868312

[ref24] Tang X. J.; Wu Y.; Zhao R.; Kou X.; Dong Z.; Zhou W.; Zhang Z.; Tan W.; Fang X. Photorelease of pyridines using a metal-free photoremovable protecting group. Angew. Chem., Int. Ed. 2020, 59, 18386–18389. 10.1002/anie.202005310. 32671906

[ref25] Koziar J. C.; Cowan D. O. Photochemical heavy-atom effects. Acc. Chem. Res. 1978, 11, 334–341. 10.1021/ar50129a003.

[ref26] Arvin M. C.; Wokosin D. L.; Banala S.; Lavis L. D.; Drenan R. M. Probing nicotinic acetylcholine receptor function in mouse brain slices via laser flash photolysis of photoactivatable nicotine. J. Visualized Exp. 2019, e58873 10.3791/58873-v. PMC6494620 30735191

[ref27] Yan Y.; Peng C.; Arvin M. C.; Jin X. T.; Kim V. J.; Ramsey M. D.; Wang Y.; Banala S.; Wokosin D. L.; McIntosh J. M.; Lavis L. D.; Drenan R. M. Nicotinic cholinergic receptors in VTA glutamate neurons modulate excitatory transmission. Cell Rep. 2018, 23, 2236–2244. 10.1016/j.celrep.2018.04.062. 29791835 PMC5999341

[ref28] Zhang X. F.; Shieh C. C.; Chapman M. L.; Matulenko M. A.; Hakeem A. H.; Atkinson R. N.; Kort M. E.; Marron B. E.; Joshi S.; Honore P.; Faltynek C. R.; Krafte D. S.; Jarvis M. F. A-887826 is a structurally novel, potent and voltage-dependent Na(v)1.8 sodium channel blocker that attenuates neuropathic tactile allodynia in rats. Neuropharmacology 2010, 59, 201–207. 10.1016/j.neuropharm.2010.05.009. 20566409

[ref29] Carcea I.; Caraballo N. L.; Marlin B. J.; Ooyama R.; Riceberg J. S.; Mendoza Navarro J. M.; Opendak M.; Diaz V. E.; Schuster L.; Alvarado Torres M. I.; Lethin H.; Ramos D.; Minder J.; Mendoza S. L.; Bair-Marshall C. J.; Samadjopoulos G. H.; Hidema S.; Falkner A.; Lin D.; Mar A.; Wadghiri Y. Z.; Nishimori K.; Kikusui T.; Mogi K.; Sullivan R. M.; Froemke R. C. Oxytocin neurons enable social transmission of maternal behaviour. Nature 2021, 596, 553–557. 10.1038/s41586-021-03814-7. 34381215 PMC8387235

[ref30] Frantz M. C.; Rodrigo J.; Boudier L.; Durroux T.; Mouillac B.; Hibert M. Subtlety of the structure-affinity and structure-efficacy relationships around a nonpeptide oxytocin receptor agonist. J. Med. Chem. 2010, 53, 1546–1562. 10.1021/jm901084f. 20104850

[ref31] Pitt G. R.; Batt A. R.; Haigh R. M.; Penson A. M.; Robson P. A.; Rooker D. P.; Tartar A. L.; Trim J. E.; Yea C. M.; Roe M. B. Non-peptide oxytocin agonists. Bioorg. Med. Chem. Lett. 2004, 14, 4585–4589. 10.1016/j.bmcl.2004.04.107. 15357997

[ref32] Harenza J. L.; Diamond M. A.; Adams R. N.; Song M. M.; Davidson H. L.; Hart L. S.; Dent M. H.; Fortina P.; Reynolds C. P.; Maris J. M. Transcriptomic profiling of 39 commonly-used neuroblastoma cell lines. Sci. Data 2017, 4, 170033 10.1038/sdata.2017.33. 28350380 PMC5369315

[ref33] Sanborn B. M.; Dodge K.; Monga M.; Qian A.; Wang W.; Yue C. Molecular mechanisms regulating the effects of oxytocin on myometrial intracellular calcium. Adv. Exp. Med. Biol. 1998, 449, 277–286. 10.1007/978-1-4615-4871-3_35. 10026815

[ref34] Masuho I.; Kise R.; Gainza P.; Von Moo E.; Li X.; Tany R.; Wakasugi-Masuho H.; Correia B. E.; Martemyanov K. A. Rules and mechanisms governing G protein coupling selectivity of GPCRs. Cell Rep. 2023, 42, 113173 10.1016/j.celrep.2023.113173. 37742189 PMC10842385

[ref35] Deo C.; Sheu S. H.; Seo J.; Clapham D. E.; Lavis L. D. Isomeric tuning yields bright and targetable red Ca(2+) indicators. J. Am. Chem. Soc. 2019, 141, 13734–13738. 10.1021/jacs.9b06092. 31430138

[ref36] Froemke R. C.; Ahmed I. A.; Liu J. J.; Gieniec K. A.; Bair-Marshall C. J.; Adewakun A. B.; Hetzler B. E.; Arp C. J.; Khatri L.; Vanwalleghem G. C.; Seidenberg A. T.; Cowin P.; Trauner D.; Chao M. V.; Davis F. M.; Tsien R. W. Optopharmacological tools for precise spatiotemporal control of oxytocin signaling in the central nervous system and periphery. Res. Square 2023, rs-2715993 10.21203/rs.3.rs-2715993/v1.

