# Supplementary material for: Elucidating and Optimizing the Photochemical Mechanism of Coumarin-Caged Tertiary Amines
Source: J Am Chem Soc. 2024 Jul 18;146(30):20627–35. doi: 10.1021/jacs.4c03092 (PMC11295134; doi:10.1021/jacs.4c03092)
Supplement: Supplementary file 1 — ja4c03092_si_001.pdf [file ja4c03092_si_001.pdf]

SUPPORTING INFORMATION

Elucidating and optimizing the photochemical mechanism of coumarin-caged tertiary amines

Sambashiva Banala[1], Xiao-Tao Jin[2], Tanya L. Dilan[1], Shu-Hsien Sheu[1],
David E. Clapham,[1] Ryan M. Drenan[2], and Luke D. Lavis[1,*]

[1]*Janelia Research Campus, Howard Hughes Medical Institute, Ashburn, VA, USA*
[2]*Department of Translational Neuroscience, Wake Forest University School of Medicine, Winston-Salem, NC, USA*

*Email: lavisl@janelia.hhmi.org

EXPERIMENTAL INFORMATION

Page	Contents
S2	Figures S1–S18
S15	Schemes S1–S6
S19	Experimental Details: Spectroscopy, uncaging, and biological experiments
S24	Experimental Details: Synthesis
S37	References
S38	NMR and LC–MS Data

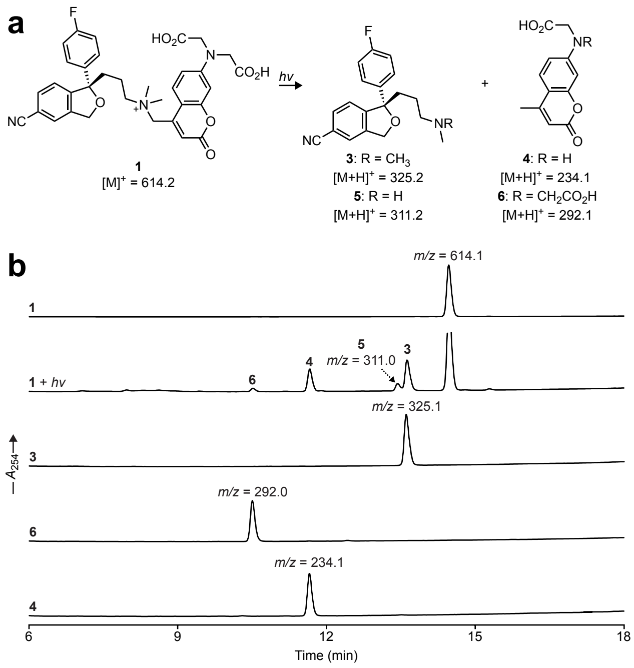

Figure S1. Photochemistry of photoactivable "caged" escitalopram (PAEsc; 1**).** (**a**) Photolysis reaction of photoactivable escitalopram **1** generating escitalopram (**3**) and byproduct **4** along with minor products **5** and **6**. (**b**) LC–MS analysis of the photochemical outcome of **1**.

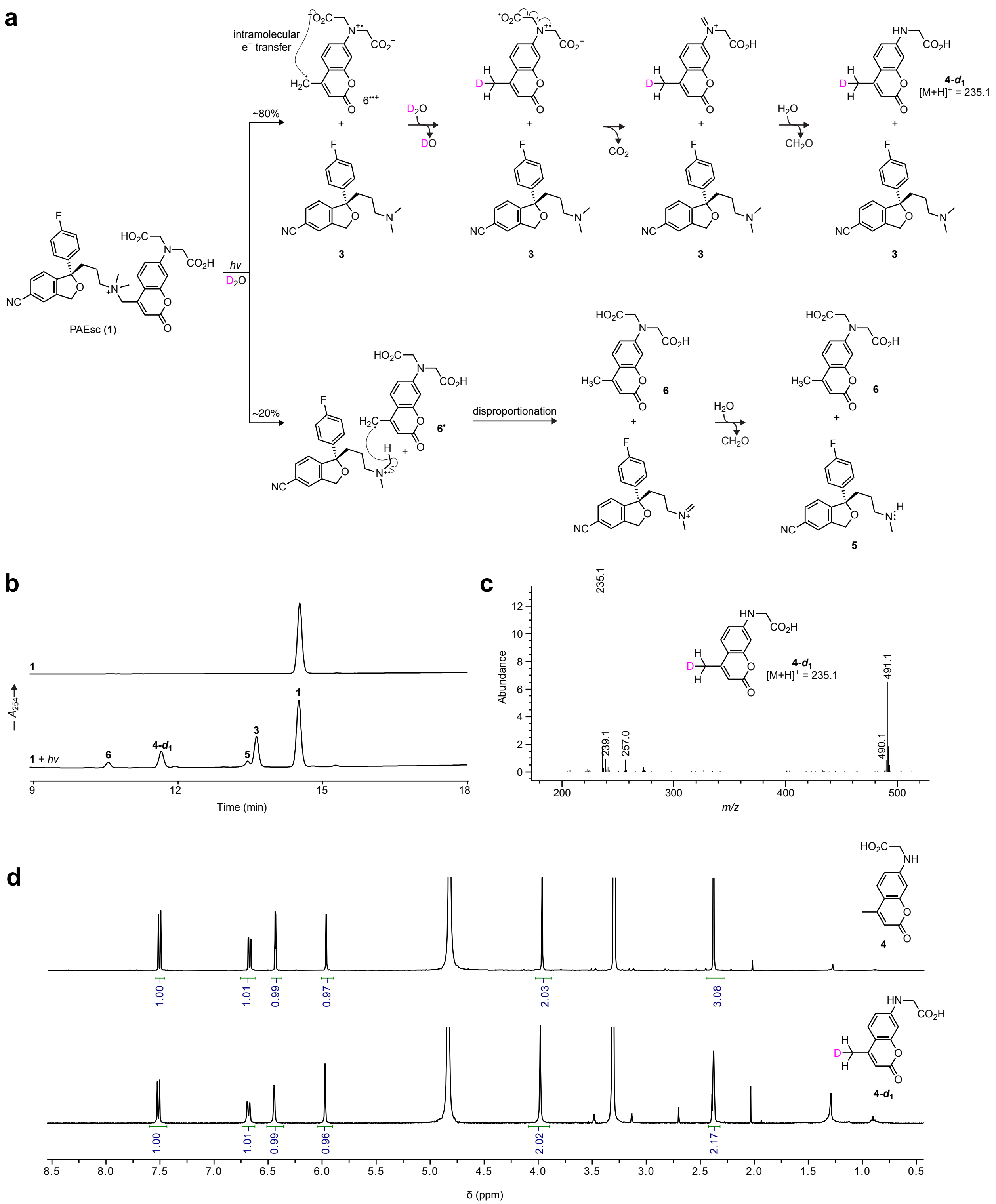

Figure S2. Generation of 4-d_1 **upon photolysis of** 1 **in D_2O.** (**a**) Proposed mechanism of deuterium incorporation into coumarin byproduct **4** in D_2O. (**b**) LC–MS chromatogram of **1** in D_2O before and after illumination. (**c**) Mass spectrogram of **4-*d*₁** peak in the LC–MS chromatogram indicating deuterium incorporation. (**d**) ^1H NMR spectra of compounds **4** and **4-*d*₁** in CD_3OD. Compound **4** was available from previous work;[1] **4-*d*₁** was obtained from HPLC purification of photolysed sample of **1** in D_2O.

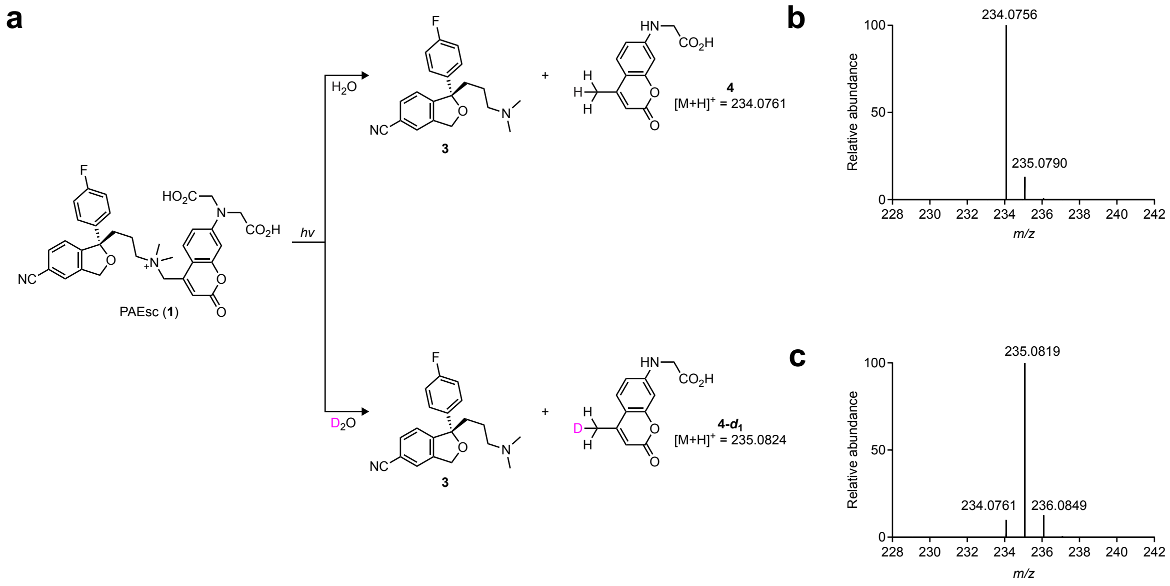

Figure S3. Quantification of deuterium incorporation in 4-*d_1* **upon photolysis of** 1 **in D_2O.** (**a**) Reaction of **1** in either H_2O or D_2O to produce **3** and either **4** or **4-*d_1***, respectively. (**b**) HRMS of **4** produced upon photolysis of **1** in H_2O. (**c**) HRMS of **4-*d_1*** produced upon photolysis of **1** in D_2O; calculated deuterium incorporation = 91%.

a

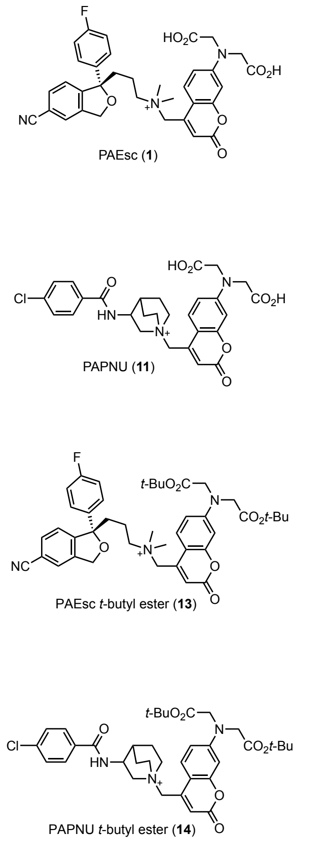

b

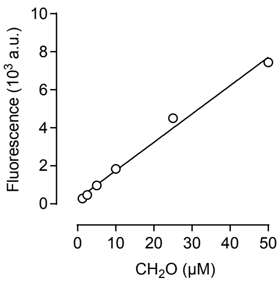

c

compound	concentration	hv	detected CH_2O (μM)
**1**	50 μM	−	<1
	50 μM	+	48
**11**	50 μM	−	<1
	50 μM	+	41
**13**	25 μM	−	<1
	25 μM	+	13
	50 μM	−	<1
	50 μM	+	25
**14**	25 μM	−	<1
	25 μM	+	<1
	50 μM	−	<1
	50 μM	+	<1

Figure S4. Generation of formaldehyde from 1**,** 11**, and** 13**, but not** 14**.** (**a**) Structures of compounds **1**, **11**, **13**, and **14**. (**b**) Representative standard curve showing increase of fluorescence ($\lambda_{ex}/\lambda_{em}$ = 535 nm/587 nm) as [CH_2O] increases. (**c**) Detected [CH_2O] (μM) before and after photolysis of compounds **1**, **11**, **13**, and **14**.

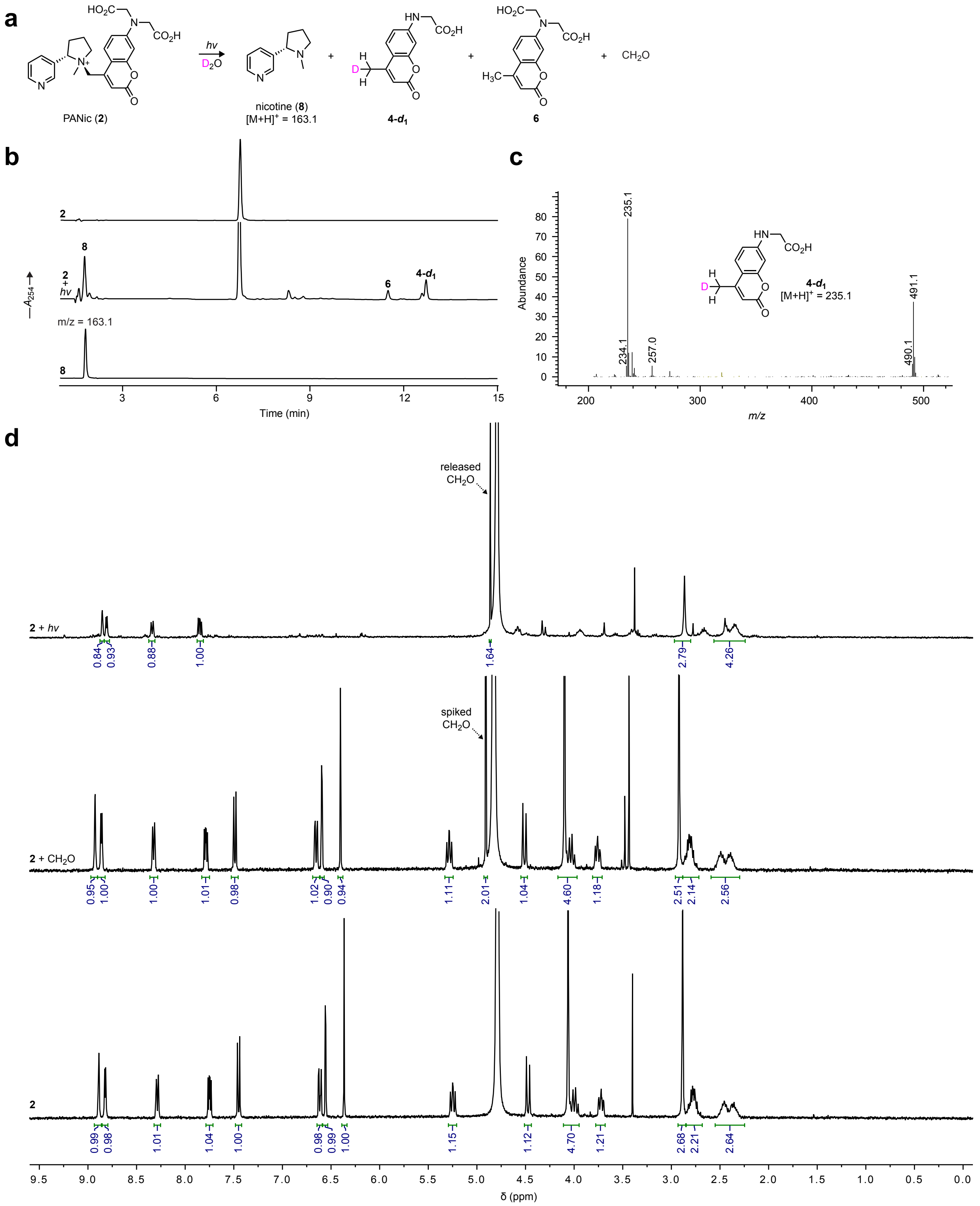

Figure S5. Photolysis of PANic (2). (**a**) Photolysis reaction of photoactivable nicotine (PANic, **2**) in D_2O generating nicotine (**8**), **4-*d*$_1$**, **6** and formaldehyde. (**b**) LC–MS chromatogram of **2** in D_2O before and after illumination with chromatogram of **8** as reference. (**c**) Mass spectrogram of **4-*d*$_1$** peak in the LC–MS chromatogram indicating deuterium incorporation. (**d**) ^{1}H NMR spectrum of **2** before and after illumination with spectra of **2** spiked with CH_2O as reference.

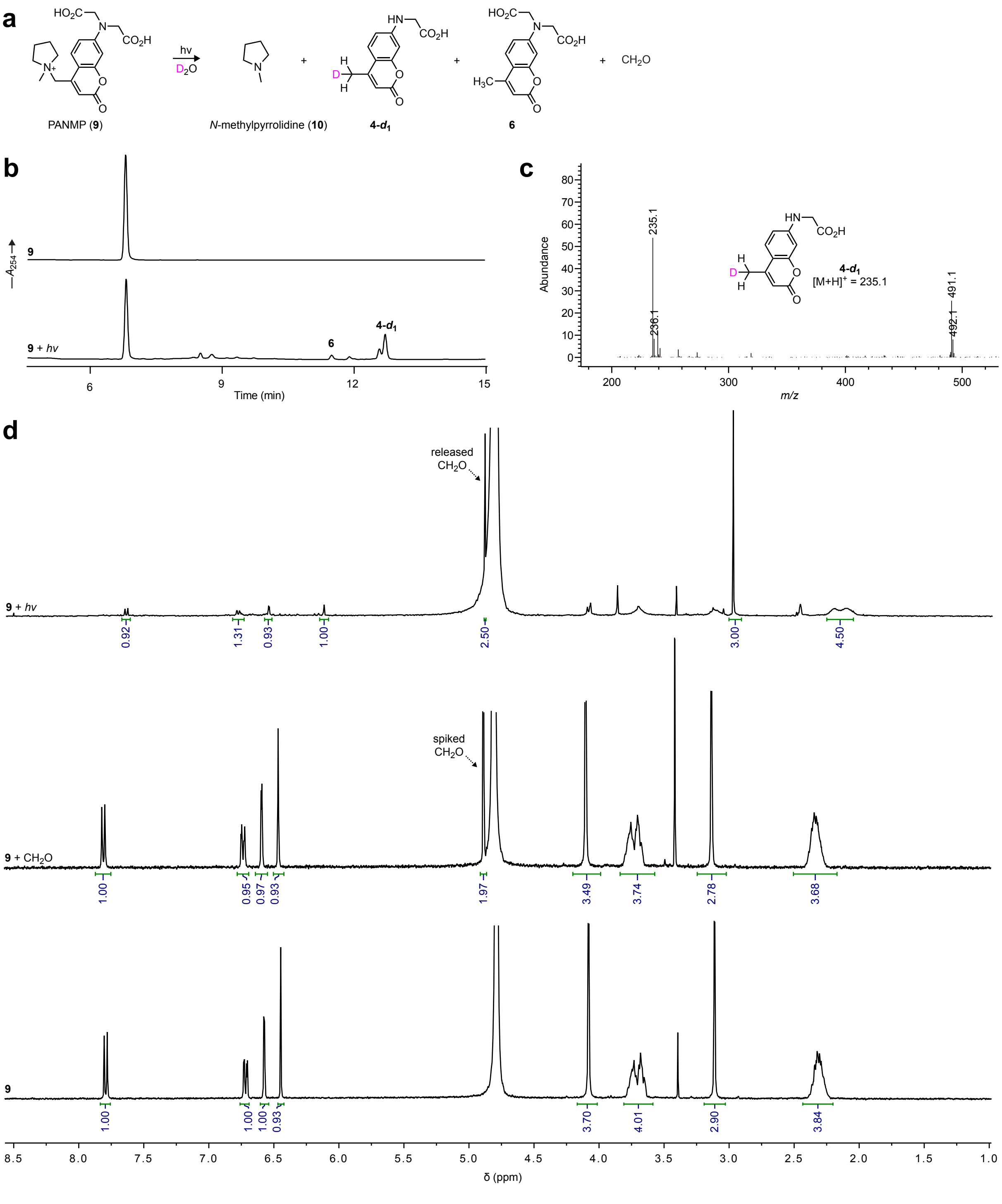

Figure S6. Photolysis of PANMP (9). (**a**) Photolysis reaction of photoactivable *N*-methylpyrrolidine (PANMP, **9**) in D_2O generating *N*-methylpyrrolidine (NMP, **10**), **4-*d*₁**, **6** and formaldehyde. (**b**) LC–MS chromatogram of **9** in D_2O before and after illumination. (**c**) Mass spectrogram of **4-*d*₁** peak in the LC–MS chromatogram indicating deuterium incorporation. (**d**) ^{1}H NMR spectrum of **9** before and after illumination with spectra of **9** spiked with CH_2O as reference.

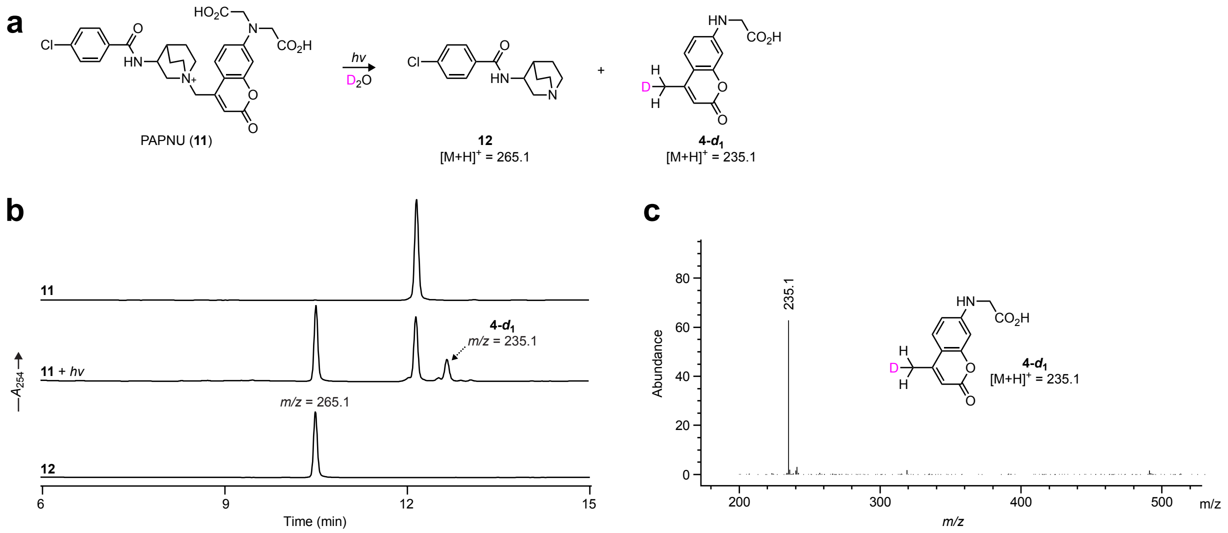

Figure S7. Photolysis of PAPNU (11**).** (**a**) Photolysis reaction of photoactivable PNU 282,987 (PAPNU, **11**) in D_2O generating PNU 282,987 (**12**) and **4**-d_1. (**b**) LC–MS chromatogram of **11** in D_2O before and after illumination with chromatogram of **12** as reference. (**c**) Mass spectrogram of **4**-d_1 peak in the LC–MS chromatogram indicating deuterium incorporation.

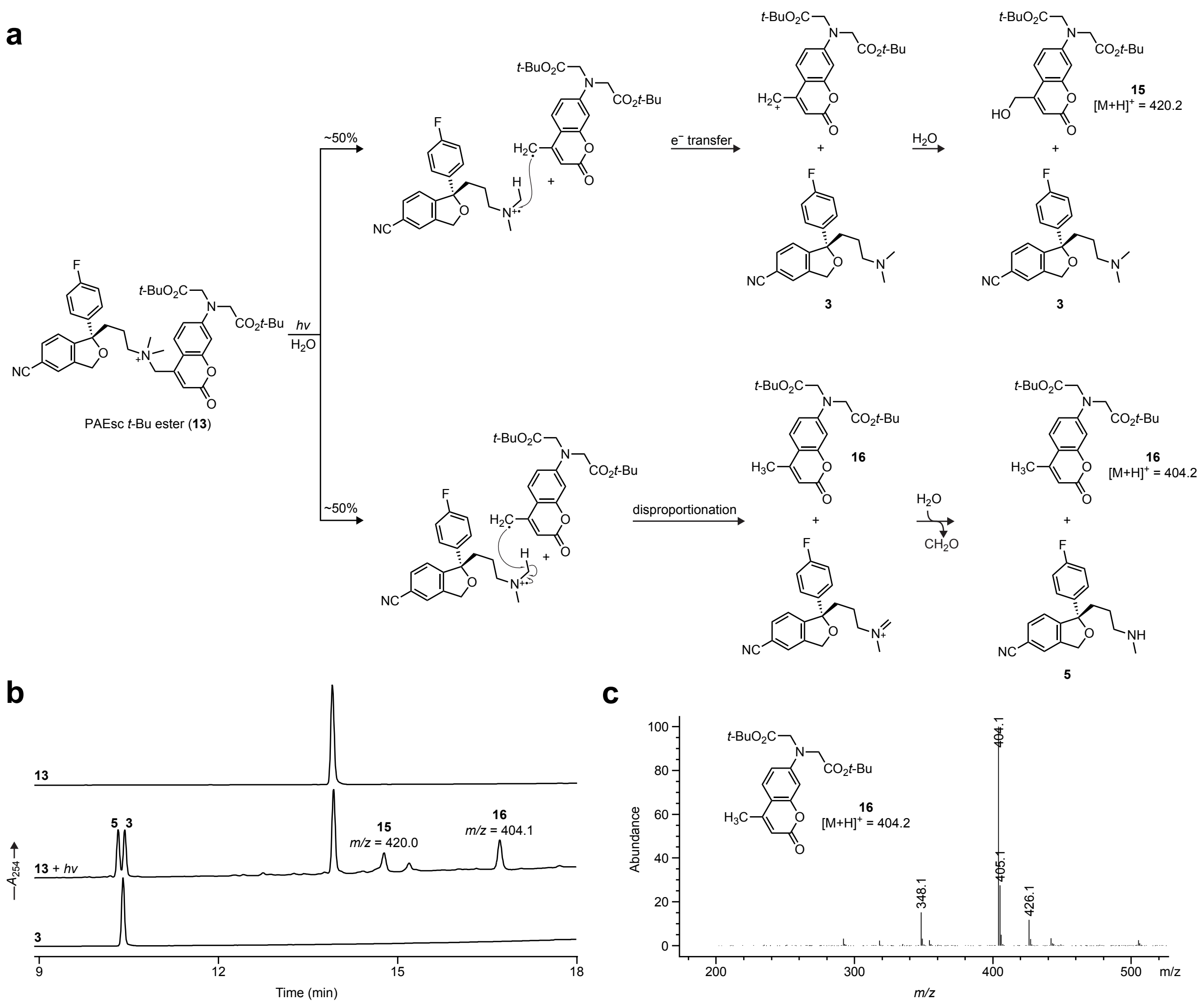

Figure S8. Photolysis of PAEsc di(*t*-butyl ester) (13**).** (**a**) Photolysis reaction of photoactivable escitalopram di(*t*-butyl ester) (PAEsc *t*-butyl ester, **13**) generating escitalopram (**3**), norescitalopram (**5**), heterolysis product **15** and homolysis product **16**. (**b**) LC–MS chromatogram of **13** before and after illumination with chromatogram of **3** as reference. (**c**) Mass spectrogram of **16** peak in the LC–MS chromatogram indicating lack of *N*-dealkylation.

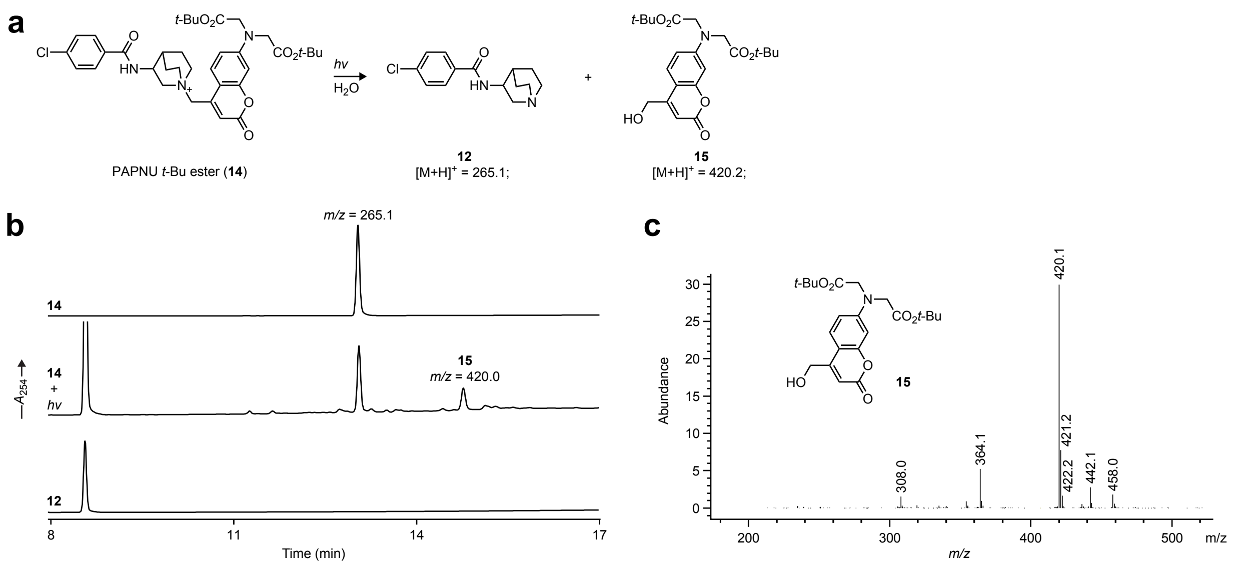

Figure S9. Photolysis of PAPNU di(*t*-butyl ester) (14**).** (**a**) Photolysis reaction of photoactivable PNU 282,987 di(*t*-butyl ester) (PAPNU *t*-butyl ester, **14**) generating PNU 282,987 (**12**) and **15**. (**b**) LC–MS chromatogram of **14** before and after illumination with chromatogram of **12** as reference. (**c**) Mass spectrogram of the **15** peak in the LC–MS chromatogram.

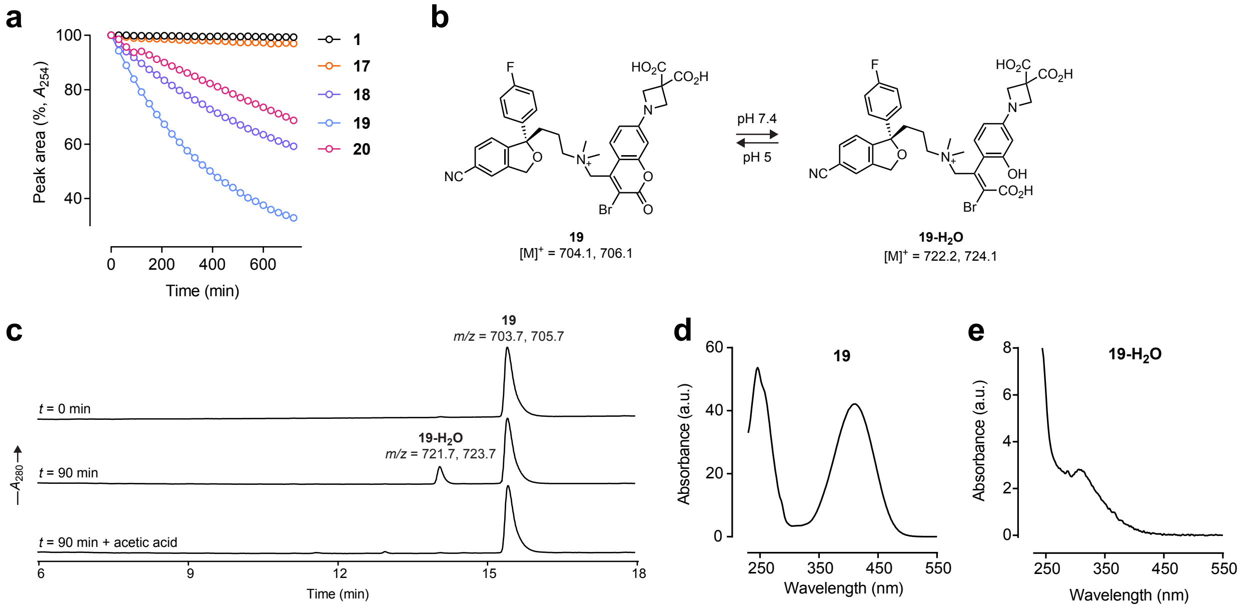

Figure S10. Chemical stability of coumarin-caged escitaloprams 1, **and** 17–20. (**a**) LC–MS peak area *vs.* incubation time of compounds **1,17–20** in PBS. (**b**) Reversible hydrolysis of reaction of ABC-caged escitalopram **19**. (**c**) LC–MS chromatogram of **19** at t = 0 min, t = 90 min, and t = 90 min with addition of acetic acid. (**d**) Absorbance spectrum of the **19** peak in the LC–MS chromatogram. (**e**) Absorbance spectrum of the **19**-H_2O adduct peak in the LC–MS chromatogram.

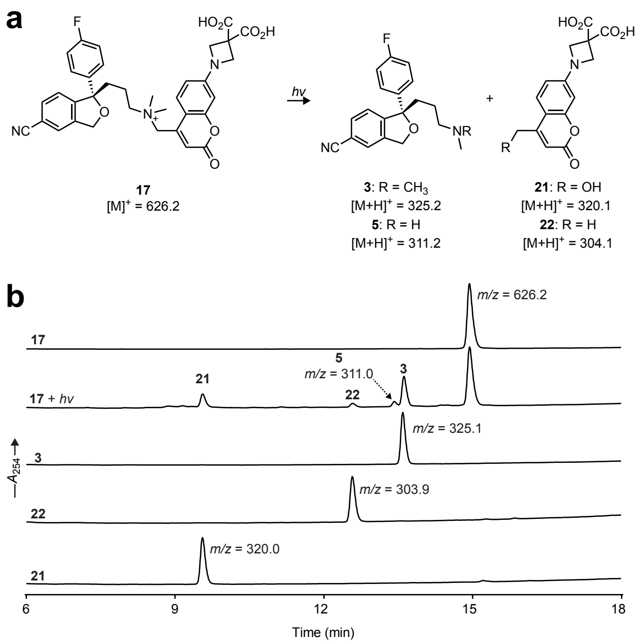

Figure S11. Photolysis of caged escitalopram 17. (**a**) Photolysis reaction of photoactivable escitalopram **17** generating escitalopram (**3**) and byproduct **21** along with minor products **5** and **22**. (**b**) LC–MS analysis of the photochemical outcome of **17**.

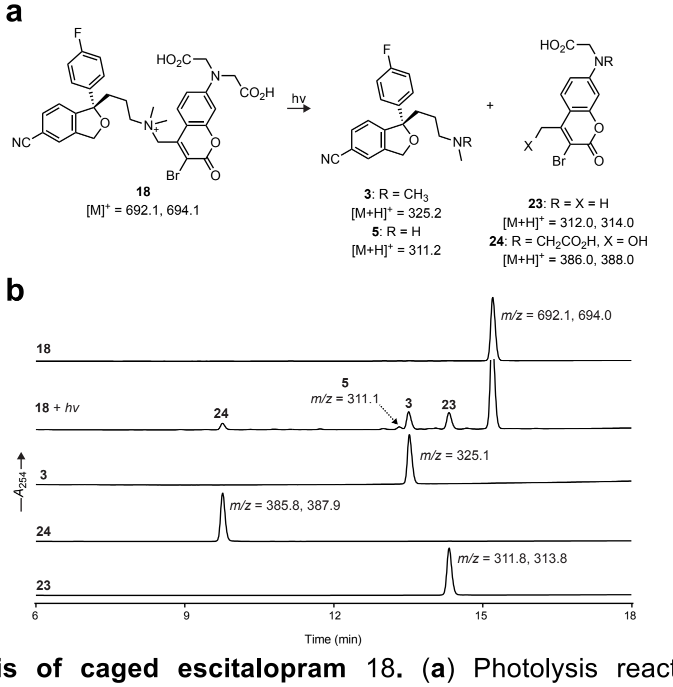

Figure S12. Photolysis of caged escitalopram 18. (**a**) Photolysis reaction of photoactivable escitalopram **18** generating escitalopram (**3**) and byproduct **23** along with minor products **5** and **24**. (**b**) LC–MS analysis of the photochemical outcome of **18**.

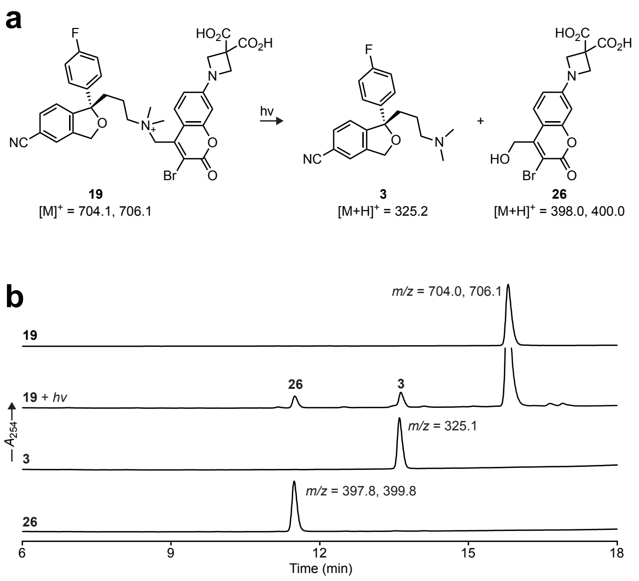

Figure S13. Photolysis of caged escitalopram 19. (**a**) Photolysis reaction of photoactivable escitalopram **19** generating escitalopram (**3**) and byproduct **26**. (**b**) LC–MS analysis of the photochemical outcome of **19**.

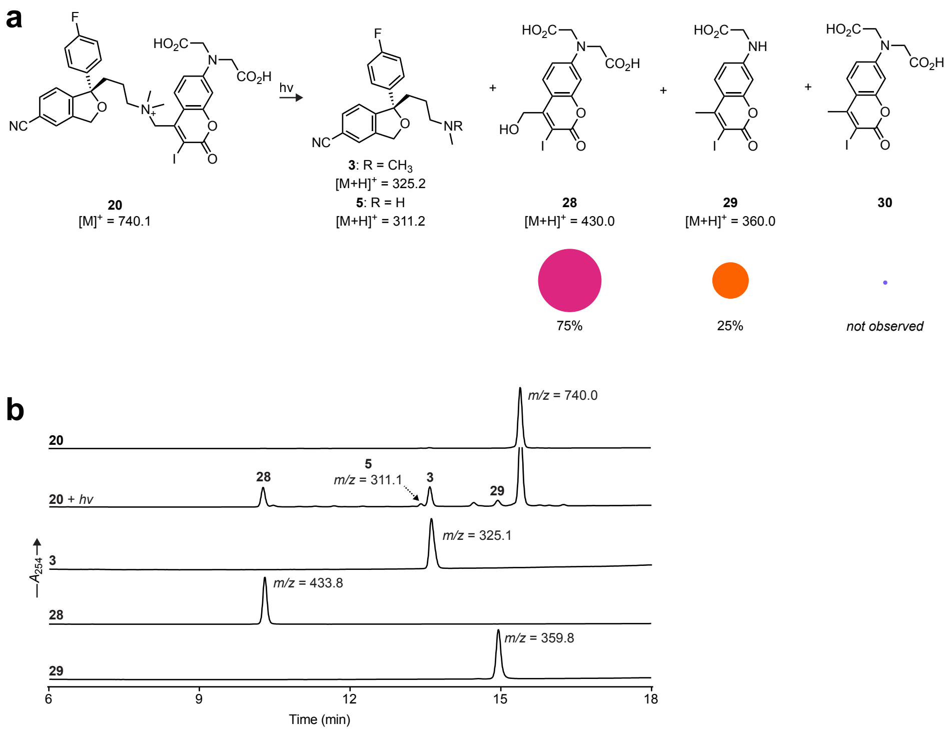

Figure S14. Photolysis of caged escitalopram 20. (**a**) Photolysis reaction of **20** generating escitalopram (**3**) and byproduct **28** along with minor products **5** and **29**; area of circles is proportional to the relative yield of the photochemical products. (**b**) LC–MS analysis of the photochemical outcome of **20**.

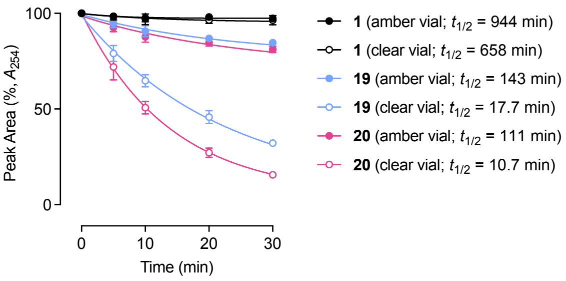

Figure S15. Stability of compounds 1, 19 **and** 20 **to ambient light.** LC–MS peak area *vs.* incubation time of compounds **1**, **19**, and **20** in PBS in clear glass vials (open circles) or amber glass vials (closed circles). Half-life ($t_{1/2}$) values were performed using a one phase decay curve fit constraining the initial value to 100% and the plateau to 0%.

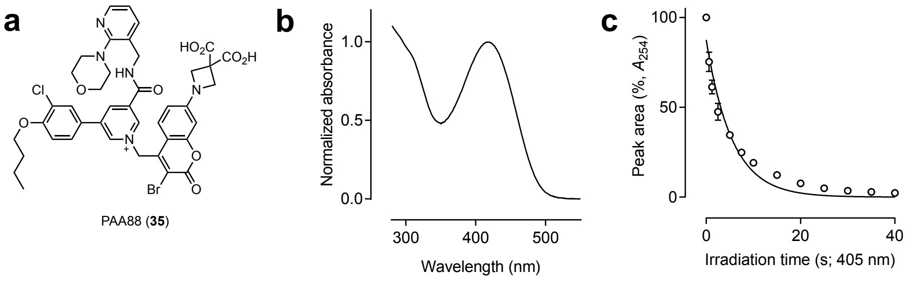

d

Compound	λ_{max} (nm)	ε ($M^{-1}cm^{-1}$)	ε_{405} ($M^{-1}cm^{-1}$)	Φ_u (%)
**35**	419	18,800	17,900	2.02

Figure S16. Properties of ABC-caged A88 (35**).** (**a**) Chemical structure of PAA88 (**35**). (**b**) Normalized absorbance spectrum of **35**. (**c**) LC–MS peak area *vs.* irradiation time of compound **35**. (**d**) Spectral and photochemical properties of **35**.

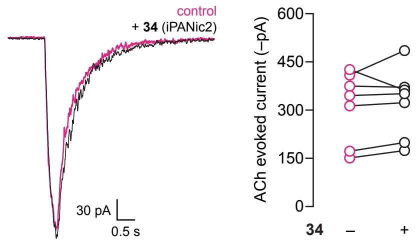

Figure S17. iPANic2 (34) is not a nAChR antagonist. Evoked current in the presence or absence of **34**. Acetylcholine (100 μM) was applied to a voltage-clamped MHb neuron via pressure ejection before (black trace) and after (red trace) superfusion of iPANic2 (**34**; 80 μM).

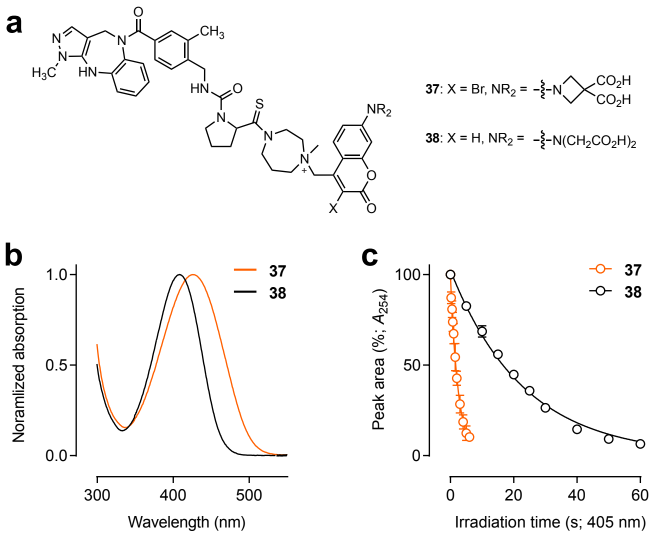

d

Compound	λ_{max} (nm)	ε ($M^{-1}cm^{-1}$)	ε_{405} ($M^{-1}cm^{-1}$)	Φ_u (%)	$\Phi_u \times \varepsilon_{405}$
**37**	426	19,500	17,100	5.9	1002
**38**	408	20,500	20,400	0.56	115

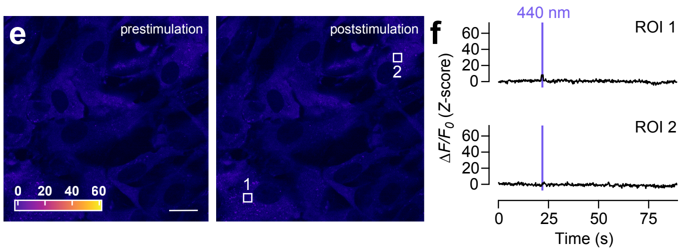

Figure S18. Properties of PATCOT (37) and BCMACM-caged derivative 38 (**a**) Chemical structures of PATCOT (**37**) and BCMACM-caged analog **38**. (**b**) Normalized absorbance spectra of **37** and **38**. (**c**) LC-MS peak area *vs.* irradiation time of compounds **37** and **38**. (**d**) Spectral and photochemical properties of **37** and **38**. (**e**) Pseudo-color fluorescence microscopy images of RPE-hTERT cells expressing HaloTag protein, loaded with BAPTA-JF_{549}-HaloTag ligand, AM ester, and incubated with DMSO. Left image shows cellular fluorescence before application of uncaging light (λ = 440 nm, 1.48 mW/cm^2, 1 s); right image shows cellular fluorescence after application of uncaging light; scale bar: 20 µm. (**f**) The change in fluorescence over basal fluorescence ($\Delta F/F_0$; Z-score) from the regions of interest (ROIs) denoted in **e** showing changes in fluorescence in response to the uncaging light pulse.

Scheme S1. Synthesis of bromomethyl coumarin cages.

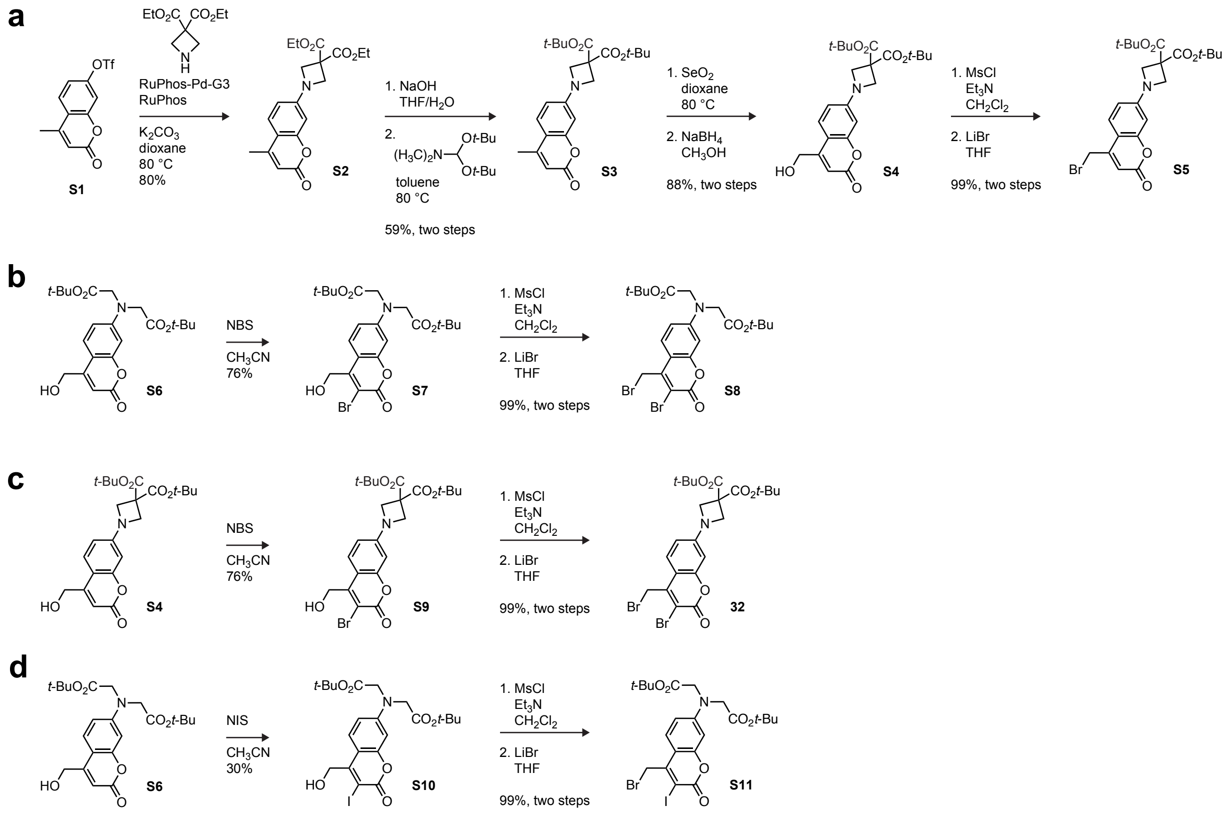

Scheme S2. Synthesis of photoactivatable escitalopram variants.

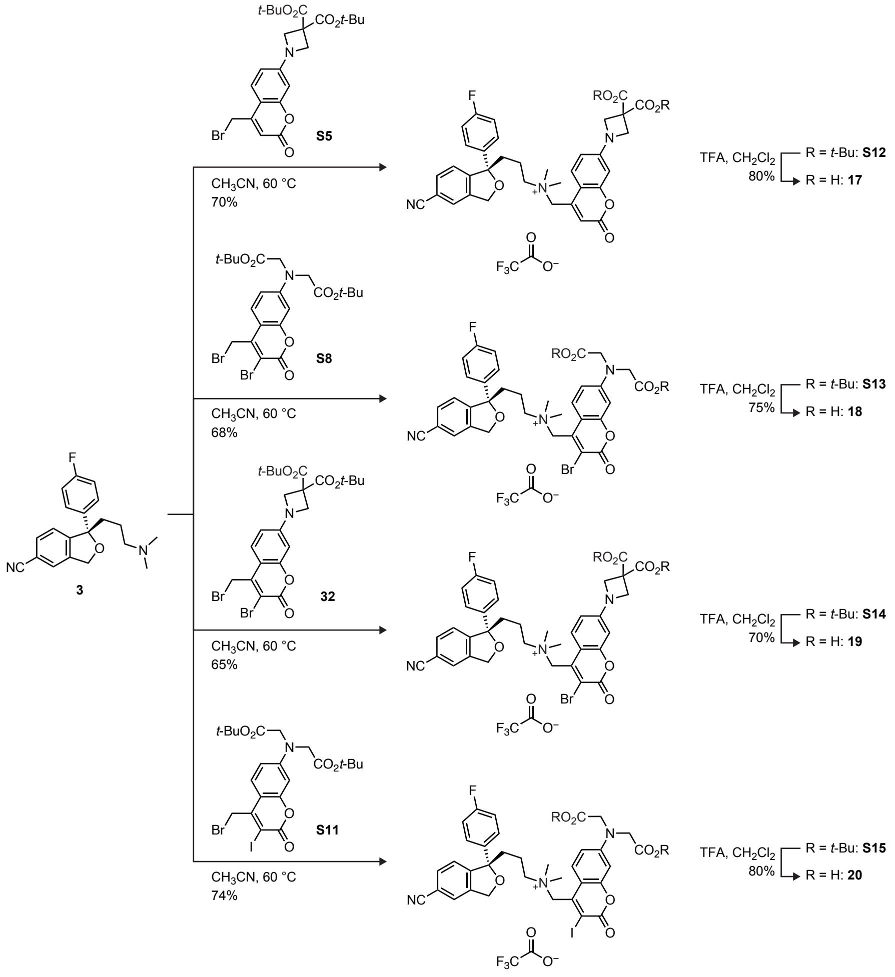

Scheme S3. Synthesis of authentic coumarin cage photoproduct samples.

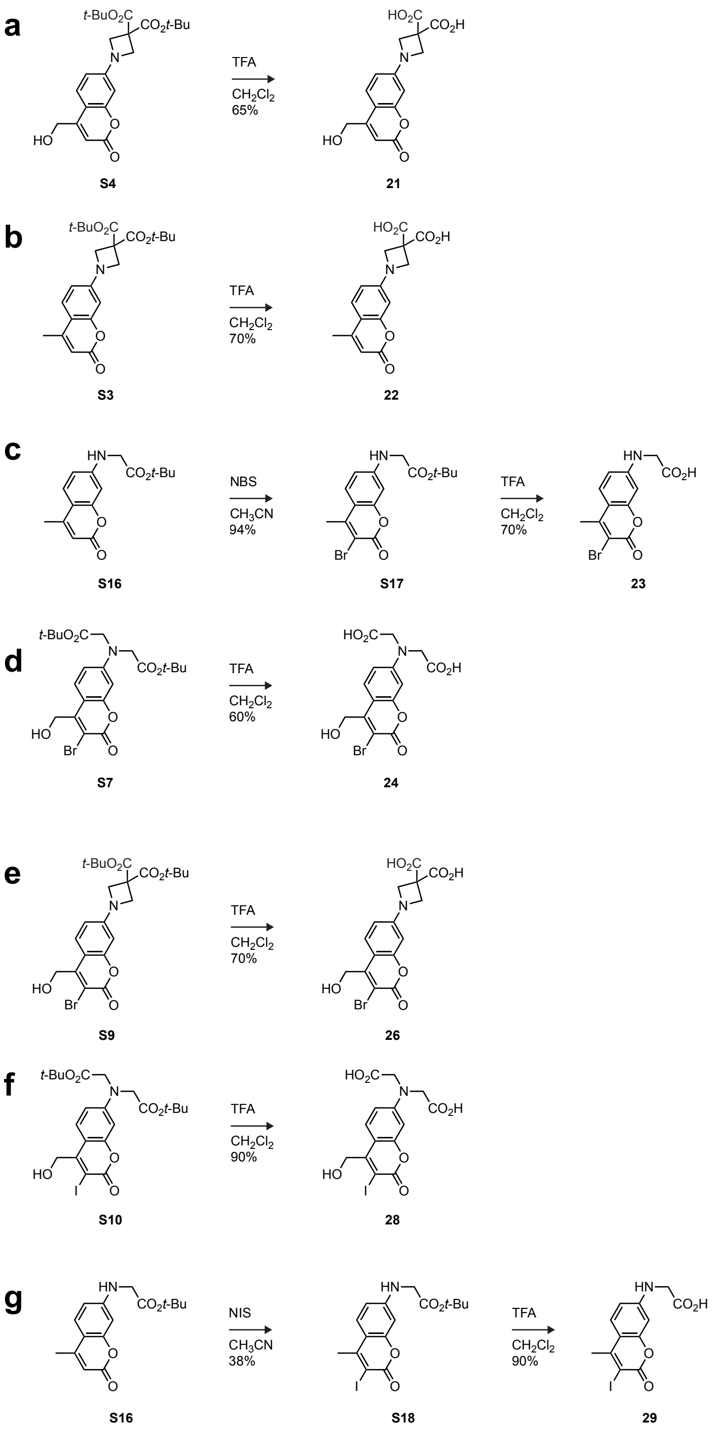

Scheme S4. Synthesis of photoactivatable nicotine variants.

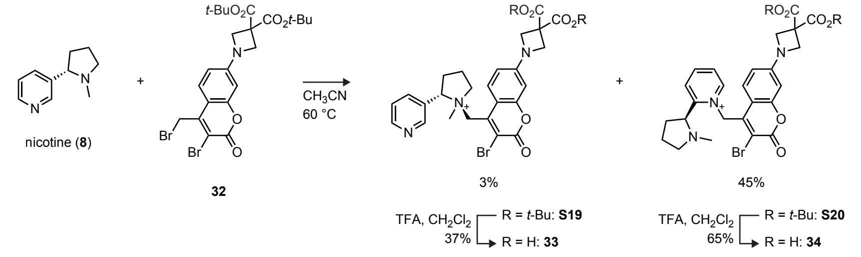

Scheme S5. Synthesis of photoactivatable A 887626.

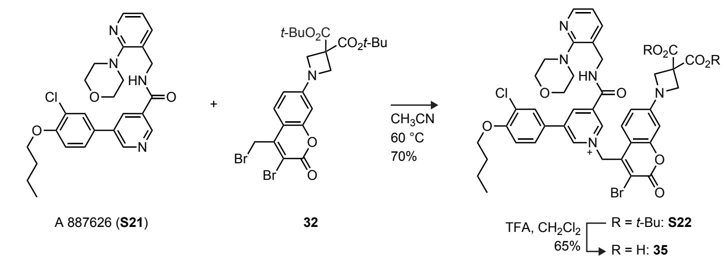

Scheme S6. Synthesis of photoactivatable TC OT 39 variants.

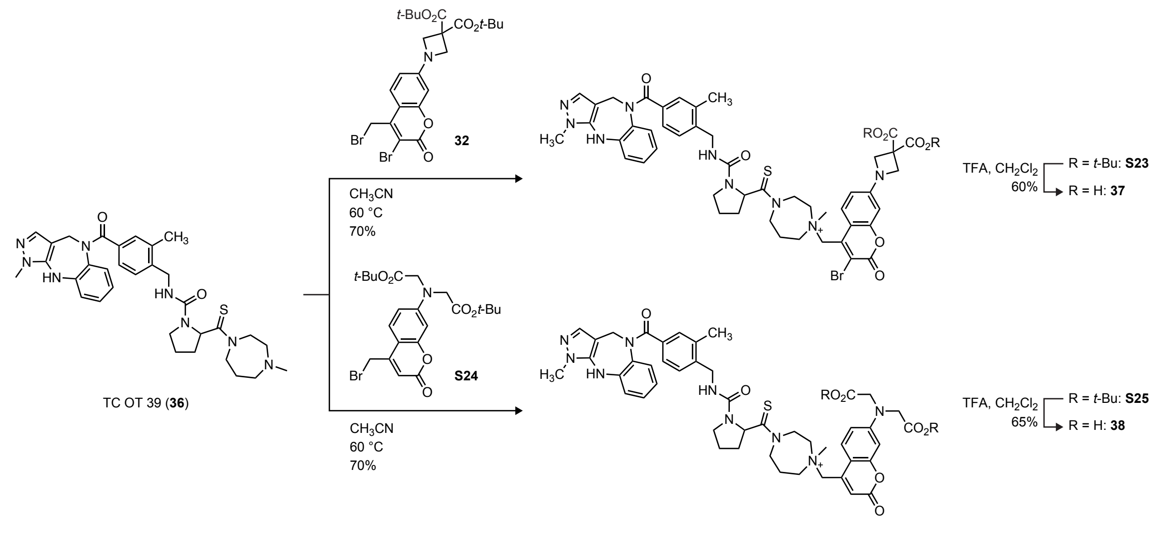

EXPERIMENTAL DETAILS: SPECTROSCOPY, UNCAGING, AND BIOLOGICAL EXPERIMENTS

Compound sources. Phosphate-buffered saline (PBS), pH 7.4 was prepared from a 10× stock (Corning, 46-013-CM). DMSO for stock solutions was purchased from Sigma-Aldrich (D2650). Compounds TC OT 39 and A 887626 were purchased from Tocris (Catalog numbers 4625 and 4249, respectively). Escitalopram oxalate was purchased from Combi-Blocks, and neutral escitalopram was obtained by dissolving it in saturated $NaHCO_3$ solution and extraction with EtOAc. Compounds **1**, **2**, **4**, **6, 7**, **9**, **11**, **13**, **14**, and **31** were available from previous work.[1]

UV–Vis and fluorescence spectroscopy. Spectroscopy was performed using 1-cm path length, 3.5-mL quartz cuvettes from Starna Cells. All measurements were taken at ambient temperature (22 ± 2 °C). Absorption spectra were recorded on a Cary Model 100 spectrometer (Agilent). Fluorescence emission spectra were recorded on a Cary Eclipse (Varian). Absolute fluorescence quantum yields were recorded on a Quantaurus-QY spectrometer (model C11374; Hamamatsu). All spectroscopy measurements were performed in phosphate-buffered saline (PBS), pH 7.4 and the values of maximum absorption wavelength (λ_{max}) and extinction coefficient (ε) are averages (n = 3). Normalized spectra are shown for clarity.

Uncaging Quantum Yield (Φ_u) Determination. Photochemistry was performed in 1-cm path length, 3.5-mL quartz cuvettes (Starna) in a Luzchem LZC 4V photoreactor equipped with 365 nm UV lamps, a carousel, and a timer as previously described. Briefly, the light intensity was calibrated by potassium ferrioxalate actinometry. A solution of 60 mM $K_3Fe(C_2O_4)_3$ was irradiated using the photoreactor setup and released Fe^{2+} was determined by complexometry with 1,10 phenanthroline. Using the known quantum yield of this process (Φ = 1.21), we determined the photon flux (I) = 3.57×10^{-7} ein/min·cm^2. For the conversion of PA-drug, 25 µM samples were irradiated and a small aliquot (50 µL) was placed in an amber glass high recovery HPLC vial. These samples were analyzed by high-performance liquid chromatography (HPLC) was performed on an Agilent 1200 Analytical HPLC system equipped with an autosampler and diode array detector. The uncaging quantum yield (Φ_u, mol/ein) was determined by fitting a plot of HPLC peak integral signal (S) *versus* irradiation time to a one-phase exponential decay described by equation 1:

$$S_t = S_0 - S_0(e^{-I\sigma\Phi_u t}) \qquad (1)$$

where S_0 = signal prior to irradiation, t = irradiation time (min), S_t = signal at time t, I = irradiation (ein/min·cm^2), and σ = decadic extinction coefficient (in units of cm^2/mol; 1000-fold higher than the ε value with units of $M^{-1}cm^{-1}$ based on cuvette geometry). For the conversion of compound **1** to compound **3**, we determined Φ_u= 0.48%, and for the compound **18** Φ_u is 14.5%. For other caged compounds, the uncaging quantum yield was determined by illumination with 405 nm LED (LOCTITE CL20 flood array) using **1** as a standard.

Analysis of photolysis products by tandem high-pressure liquid chromatography–mass spectrometry (LC–MS). To analyze photolysis products, solutions of photoactivatable compounds **1, 17–20** (100 µM) were prepared in PBS and placed in a glass vial and irradiated with 405 nm LED. An aliquot of this freshly prepared solution was analyzed by LC–MS using an Agilent 1200 LC–MS system equipped with an autosampler, diode array detector, and mass spectrometry detector (ESI; positive ion mode) using a 4.6 × 150 mm Gemini NX-C18 column with a 5–95% or 5–

50% gradient of CH_3CN in H_2O containing constant 0.1% (v/v) TFA. Chromatograms were monitored using absorbance at 254 nm or 400 nm. Parent drugs and photo byproducts synthesized in-house were also analyzed for confirmation.

Quantification of deuterium incorporation in 4-d_1 upon photolysis of 1 in D_2O using high-resolution mass spectrometry (HRMS). Solutions of **1** (100 μM) were prepared in H_2O or D_2O (99.9 atom % D). These samples were photolyzed for 20 s using 405 nm light. HRMS analysis of the samples was performed on an Orbitrap Fusion Lumos Tribrid mass spectrometer coupled with the Vanquish Flex UHPLC system. Illuminated samples loaded onto an Agilent ZORBAX Eclipse Plus C18 column (2.1 × 50 mm, 1.8 μm) using a linear gradient of 10–95% (v/v) CH_3CN in H_2O containing a constant 0.1% (v/v) formic acid additive. The eluent was introduced to the mass spectrometer through electrospray ionization in positive mode. Orbitrap was chosen as the mass analyzer with mass range set at *m/z* = 150–700 for full MS scan. A mass resolution of 120,000 was used and the RF lens was set at 30%. Incorporation of deuterium was quantified from the intensities of ions detected.

Measurement of chemical stability. To examine the dark stability of the photoactivatable compounds in PBS, we assessed samples by using an Agilent 1200 system equipped with an autosampler and diode array detector using a 4.6 × 150 mm Gemini NX-C18 column with a 5–95% gradient of CH_3CN in H_2O containing constant 0.1% (v/v) TFA. Chromatograms were monitored using absorbance at 254 nm or 400 nm. To investigate chemical stability, solutions of photoactivatable compounds (25 μM) were prepared in PBS, pH 7.4. This freshly prepared solution was immediately analyzed (t = 0), and continued injections with 30 min interval.

To explore the reversibility of the hydrolysis, 100 μM samples were prepared in PBS, pH 7.4. An aliquot of this freshly prepared solution was immediately analyzed by LC–MS. The solution was then incubated at ambient temperature protected from light for different durations and analyzed again by LC–MS. For coumarin ring closing, 15 μL acetic acid was added to 50 μL sample and waited for 45 min. These samples were then analyzed by LC–MS.

Formaldehyde assay. To detect the released formaldehyde, we used the Formaldehyde Assay Kit (Fluorometric) from Abcam (Catalog number ab196997). Briefly, 50 μL assay reagents mix were added to 50 μL of 25 μM or 50 μM pre- or post photolysed PAEsc, or other photoactivable compounds, in PBS. Samples were taken in a black, clear-bottom 96-well microplate (Nunc, 165305), incubated at room temperature for 20 min and protected from light. The fluorescence was monitored using 535 nm excitation and 587 nm emission, reading from the bottom of the plate using an Infinite M1000Pro microplate reader (TECAN). A fresh formaldehyde calibration curve was generated for each experiment.

Evaluation of stability to ambient light. To examine the stability of compounds under ambient light, solutions of photoactivatable compounds (25 μM) were prepared in PBS in clear glass vials or amber glass vials with a Teflon-lined lid. These solutions were placed on the lab bench and exposed to ambient light. A small aliquot (50 μL) was removed at specific timepoints and analyzed by LC–MS as described above. Points were fit to a one-phase decay curve.

Recommended handling procedures for ABC-caged compounds. Compounds **19**, **33**, **34**, and **37** are sensitive to ambient light and susceptible to spontaneous hydrolysis after extended time in aqueous solution. Compounds stored as solid or as DMSO stock solutions (10 mM) at −20 °C showed >99% purity after >1 year storage time. DMSO stock solutions (10 mM) are stable at ambient temperature for >7 days when protected from light. We recommend preparing and storing single-use aliquots as either solid material or DMSO stocks and preparing working solutions by dissolving in appropriate buffer immediately before use. We also recommend covering laboratory lights with RC–3 film (coloredfilms.com) to filter out short wavelength light.

Brain slice preparation and nAChR activation using photoactivatable nicotine compounds. Brain slices preparation and current evoked by nAChR activation were performed as previously described.[1] Mice were anesthetized with Euthasol (sodium pentobarbital, 100 mg/kg; sodium phenytoin, 12.82 mg/kg) before trans-cardiac perfusion at 4 °C with oxygenated (95% O_2/5% CO_2) N-methyl-D-glucamine (NMDG)-based recovery solution that contains (in mM): 93 NMDG, 2.5 KCl, 1.2 NaH_2PO_4, 30 $NaHCO_3$, 20 HEPES, 25 glucose, 5 sodium ascorbate, 2 thiourea, 3 sodium pyruvate, 10 $MgSO_4 \cdot 7H_2O$, and 0.5 $CaCl_2 \cdot 2H_2O$; 300–310 mOsm; pH 7.3–7.4). Brains were immediately dissected after the perfusion and held in oxygenated, 4 °C recovery solution for one minute before cutting a brain block containing the medial habenula and sectioning the brain with a vibratome (VT1200S; Leica). Coronal slices (250 μm) were sectioned through the MHb and transferred to oxygenated, 33 °C recovery solution for 12 min. Slices were then kept in holding solution (containing in mM: 92 NaCl, 2.5 KCl, 1.2 NaH_2PO_4, 30 $NaHCO_3$, 20 HEPES, 25 glucose, 5 sodium ascorbate, 2 thiourea, 3 sodium pyruvate, 2 $MgSO_4 \cdot 7H_2O$, and 2 $CaCl_2 \cdot 2H_2O$; 300–310 mOsm; pH 7.3–7.4) for 60 min or more before recordings.

For uncaging, brain slices were prepared as follows. Animals were deeply anesthetized by inhalation of isoflurane, decapitated, and the brain was rapidly removed and immersed in ice-cold oxygenated artificial cerebrospinal fluid (ACSF) containing (in mM) 127 NaCl, 2.5 KCl, 25 $NaHCO_3$, 1.25 NaH_2PO_4, 2.0 $CaCl_2$, 1.0 $MgCl_2$, and 25 glucose (osmolarity ~310 mOsm/L). Tissue was blocked and transferred to a slicing chamber containing ice-cold ACSF, supported by a small block of 4% agar. Bilateral 250 μm-thick slices containing the MHb were cut on a Leica VT1000S and transferred into a holding chamber with ACSF equilibrated with 95% O_2/5% CO_2. Slices were incubated at 34 °C for 15–30 minutes prior to electrophysiological recording.

For PANic uncaging experiments, brain slices were transferred to a recording chamber being continuously superfused at a rate of 1.5–2.0 mL/min with oxygenated 32 °C recording solution. The recording solution contained (in mM): 124 NaCl, 2.5 KCl, 1.2 NaH_2PO_4, 24 $NaHCO_3$, 12.5 glucose, 2 $MgSO_4 \cdot 7H_2O$, and 2 $CaCl_2 \cdot 2H_2O$; 300–310 mOsm; pH 7.3–7.4). Picrotoxin (100 μM), CNQX (20 μM), and D-AP5 (50 μM) were added during subcellular uncaging experiments. Patch pipettes were pulled from borosilicate glass capillary tubes (1B150F-4; World Precision Instruments) using a programmable microelectrode puller (P-97; Sutter Instrument). Tip resistance ranged from 4.5 to 8.0 MΩ when filled with internal solution. The following internal solution was used (in mM): 135 potassium gluconate, 5 EGTA, 0.5 $CaCl_2$, 2 $MgCl_2$, 10 HEPES, 2 MgATP, and 0.1 GTP; pH adjusted to 7.25 with Tris base; osmolarity adjusted to 290 mOsm with sucrose. For subcellular uncaging, this internal solution also contained QX-314 (2 mM) for improved voltage control. We recorded from neurons in the ventral 50–60% of the MHb.

Some experiments involved recording inward currents following pressure ejection application of drug to the recorded cell using a drug-filled pipette, which was moved to within 20–40 µm of the recorded neuron using a micromanipulator. Using a Picospritzer (Parker Hannifin), a pressure ejection dispensed drug (dissolved in the same superfusion medium used on the slice) onto the recorded neuron. Ejection volume, duration, and ejection pressure varied depending on whether a short (ms) or long (s) pulse was required. The change in amplitude between the baseline and the peak was measured. Atropine (1 µM) was present in the superfusion medium when using ACh to prevent activation of muscarinic ACh receptors.

For focal nicotine uncaging using 1-photon laser (405 nm) flash photolysis, an Olympus BX51 upright microscope with a 60× (1.0 NA) objective was used to visualize cells. Prairie View 5 (Bruker Nano) software was used for acquisition via a Multiclamp 700B patch clamp amplifier (Molecular Devices). Analog signals were sampled at 5 kHz and low-pass filtered at 1 kHz, and an A/D converter (PCI-NI6052e; National Instruments) was used for digitization. Patch clamp recordings were carried out using the internal solution mentioned above, except that Alexa 568 (50 µM) or Alexa 488 (100 µM) was also included in the recording pipette to visualize cells using 2-photon laser scanning microscopy (2PLSM). After break-in, the internal solution with the Alexa dye was allowed to equilibrate for 15–20 min before imaging was initiated. 2PLSM 1-photon uncaging experiments experiments were conducted with a pulsed infrared laser (Ultra I; Coherent) tuned to 920 nm. A pockels cell was used for power attenuation. The dual-channel, 2-photon fluorescence was detected by two non-descanned detectors; green and red channels (dual emission filters: 525/70 nm and 595/50 nm) were were detected by multi-alkali photomultiplier tubes (R3896, Hamamatsu). A 405 nm continuous wave laser (100 mW OBIS FP LX; Coherent) was used for photostimulation/uncaging via a second set of x–y galvanometers incorporated into the scanhead (Cambridge Technologies).

PANic compounds **2**, **33**, and **34** were applied (80 µM in 5 mL) via superfusion to the slice using a recirculating perfusion system to conserve compound. Prairie View 5 software was used to select spots in the field of view for focal uncaging of nicotine via 405 nm laser light flashes.

Oxytocin receptor activation and Ca^{2+} Imaging in RPE-1 cells. A CMV-mEmerald-HaloTag construct on a piggyBac backbone was co-transfected with the hyperactive piggyBac transposase plasmid[2] in 2.5-to-1 ratio on doxy-inducible HTR6-HaloTag-expressing hRPE-1 cells.[3] Fluorescence-activated cell sorting was used to identify mEmerald-HaloTag positive cells, and a single clone was identified. The mEmerald-HaloTag; doxy-HTR6-HaloTag hRPE-1 cells were plated in 35mm glass bottom dishes (Cellvis D35-20-1.5-N) in 10% FBS DMEM:F12 medium (American Type Culture Collection, ATCC 30-2006). The cells were labeled with BAPTA-JF_{549}-HaloTag ligand[3] (1 µM, 1 hour) in Live Cell Imaging solution (140 mM NaCl, 2.5 mM KCl, 1.8 mM $CaCl_2$, 1.0 mM $MgCl_2$, 20 mM HEPES, pH=7.4, mOsm = 300, Thermo Fisher Scientific). The cells were then rinsed 3 times in fresh Live Cell Imaging Solution, and loaded with either control (0.2% DMSO), PA-TCOT (**37**; 2 µM; stock 1mM in DMSO, diluted 1:500) or **38** (2 µM; stock 1mM in DMSO, diluted 1:500) for subsequent experiments.

Time-lapse images were captured with a Nikon CSU-W1 inverted spinning disk confocal microscope, housed within a 37°C and CO2 5% environment chamber (Oko-Lab) using a 60× objective (Plan Apo λ 60× Oil, NA 1.42). Cells

were excited with a 561.0 nm laser line and emission was sampled through a 610/75 bandpass filter. Acquisition was performed using a sCMOS camera (Hamamatsu ORCA-Fusion BT), taken at a rate of 5.43 Hz without camera binning, and using the perfect focus system module from NIS-Elements AR v4.40.00 (Nikon). Photostimulation events were performed using 1-second pulses at an irradiance of 1.48 mW/cm^2, delivered by a DMD-based pattern illuminator (Mightex Polygon 400). This illuminator was paired with a 440 nm LED (Lumencor Spectra-III) and was controlled via TTL input from the NIS Elements AR software.

For data analysis, regions of interest (ROIs) were delineated around individual cells from the time-lapse videos. These videos originated from three distinct experimental replicates for each tested condition. Using Fiji's multimeasure tool, mean pixel intensity values were extracted for each frame over time. Additional ROIs were established in cell-free areas to account for background fluorescence noise. The mean pixel intensity values from these cell-free ROIs were then subtracted from the values of the cell-positive ROIs. To normalize the mean pixel intensity values across different experimental replicates and to analyze calcium influx responses after photostimulation, we utilized the equation 2:[4]

$$Z - scored\ \Delta F_t/F_0 = (F_t - F_0)/(SDF_0) \qquad (2)$$

where F_t is the mean pixel intensity at time t, F_0 represents the baseline fluorescence, determined by averaging the pixel intensity values during the 20-second period right before the onset of photostimulation, and SDF_0, is the standard deviation of F_0. The peak Ca^{2+} signal amplitude was extracted as the maximum fluorescence value post-photostimulation.

EXPERIMENTAL DETAILS: SYNTHESIS

General. Commercial reagents were obtained from reputable suppliers and used as received. All solvents were purchased in septum-sealed bottles stored under an inert atmosphere. All reactions were sealed with septa through which an argon atmosphere was introduced unless otherwise noted. Reactions were conducted in round-bottomed flasks containing Teflon-coated magnetic stir bars. Heating of reactions was accomplished with an aluminum reaction block on top of a stirring hotplate equipped with an electronic contact thermometer to maintain the indicated temperatures. Compounds **S1**, **S6**, **S16**, and **S24** were synthesized as previously described.[1, 5]

Reactions were monitored by thin layer chromatography (TLC) on precoated TLC glass plates (silica gel 60 F_{254} 250 µm thickness) or by LC/MS (Phenomenex Gemini NX-C18 4.6 mm × 150 mm 5 µm C18 column; 5 µL injection; 05–95% CH_3CN/H_2O, linear gradient, with constant 0.1% v/v TFA additive; 20 min run; 1 mL/min flow; ESI; positive ion mode; UV detection at 254 nm). TLC chromatograms were visualized by UV illumination or developed with $KMnO_4$ stain. Flash chromatography was performed on an automated purification system using pre-packed silica gel columns. Preparative HPLC was performed on Agilent 1200 system equipped with an autosampler and diode array detector using a Phenomenex Gemini NX 30 × 150 mm 5 µm C18 column, with a gradient of CH_3CN in H_2O containing constant 0.1% (v/v) TFA. High-resolution mass spectrometry was performed by the High-Resolution Mass Spectrometry Center at the University of Iowa.

NMR spectra were recorded on a 400 MHz spectrometer. ^1H and ^{13}C chemical shifts were referenced to TMS or residual solvent peaks. Data for ^1H NMR spectra are reported as follows: chemical shift (δ ppm), multiplicity (s = singlet, d = doublet, t = triplet, q = quartet, dd = doublet of doublets, m = multiplet), coupling constant (Hz), integration. Data for ^{13}C NMR spectra are reported by chemical shift (δ ppm) with hydrogen multiplicity (C, CH, CH_2, CH_3) information obtained from DEPT spectra. To determine the equivalents of TFA in the preparative HPLC-purified products, samples were prepared in $CDCl_3$ or CD_3CN. For photoactivatable escitalopram compounds, the fluorine atom in the escitalopram structure was used as internal standard. For other photoactivatable compounds an internal fluorobenzene standard was added. Integration of the ^1H NMR and ^{19}F NMR signals allowed calculation of TFA equivalents. We found compounds **17**–**20** contain one equivalent of TFA, PANic variants (compounds **2, 31, 33,** and **34**) contain two equivalents of TFA, compound **35** contains three equivalents of TFA, and compounds **37** and **38** contain five equivalents of TFA.

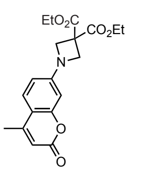

Diethyl 1-(4-methyl-2-oxo-2H-chromen-7-yl)azetidine-3,3-dicarboxylate (S2). A vial was charged with 4-methylumbelliferone triflate[5] (**S1**) (815 mg, 2.64 mmol, 1 equiv), diethyl azetidine-3,3-dicarboxylate 2,2,2-trifluoroacetic acid (1 g, 3.17 mmol, 1.2 equiv), RuPhos-G3-palladacycle (110 mg, 0.131 mmol, 0.05 eq), RuPhos (61 mg, 0.131 mmol, 0.05 equiv), and K_2CO_3 (910 mg, 6.6 mmol, 2.5 equiv). The vial was sealed and evacuated/backfilled with nitrogen (3×). Dioxane (10 mL) was added, and the reaction was flushed again with nitrogen (3×). The reaction was stirred at 80 °C for 15 h. It was then cooled to room temperature, deposited onto Celite, and concentrated to dryness. Purification by silica gel chromatography (0→40% v/v EtOAc/hexanes, linear gradient) afforded **S2** (760 mg, 80%) as a yellow solid. ^1H NMR (400 MHz, $CDCl_3$) δ:

7.41 (d, *J* = 8.6 Hz, 1H), 6.35 (dd, *J* = 8.6, 2.3 Hz, 1H), 6.28 (d, *J* = 2.3 Hz, 1H), 6.01 (q, *J* = 1.2 Hz, 1H), 4.33 (s, 4H), 4.28 (q, *J* = 7.1 Hz, 4H), 2.34 (d, *J* = 1.2 Hz, 3H), 1.30 (t, *J* = 7.1 Hz, 6H); ^{13}C NMR ($CDCl_3$, 101 MHz) δ: 169.0 (C), 161.7 (C), 155.5 (C), 153.0 (C), 152.9 (C), 125.7 (CH), 111.6 (C), 110.5 (CH), 108.2 (CH), 98.2 (CH), 62.5 (CH_2), 57.5 (CH_2), 48.9 (C), 18.7 (CH_3), 14.1 (CH_3). HRMS (ESI) calcd for $C_{19}H_{21}NO_6Na$ $[M+Na]^+$ 382.1267, found 382.1249.

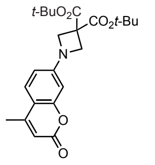

Di-*tert*-butyl 1-(4-methyl-2-oxo-2H-chromen-7-yl)azetidine-3,3-dicarboxylate (S3). Compound **S2** (1.08 g, 3.0 mmol, 1 equiv) was taken up in 2:1 THF/H_2O (30 mL), and 1 M NaOH (6.6 mL, 6.6 mmol, 2.2 equiv) was added. The reaction was stirred at room temperature for 16 h. It was then acidified with 1 M HCl (5 mL). The reaction mixture was concentrated under reduced pressure and the crude material was diluted with water and extracted with EtOAc (3×). The combined organics were washed with H_2O and saturated NaCl(aq), and then dried over $MgSO_4$(s). The resulting solution was concentrated under reduced pressure and then dried under high vacuum. The crude material was taken up in toluene (15 mL) and heated to 80 °C, after which *N,N*-dimethylformamide di-*tert*-butyl acetal (7.19 mL, 30 mmol, 10 equiv) was added dropwise over 10 min. The reaction was heated to 80 °C for 2 h, cooled to ambient temperature, diluted with 10% $NaHCO_3$, and extracted with EtOAc (2×). The combined organics were washed with H_2O and saturated NaCl(aq), dried over $MgSO_4$(s), and concentrated under reduced pressure. Flash chromatography on silica gel (0→40% v/v EtOAc/hexanes, linear gradient; dry load with Celite) provided compound **S3** as a yellow solid (690 mg, 55%). ^1H NMR ($CDCl_3$, 400 MHz) δ: 7.39 (d, *J* = 8.6 Hz, 1H), 6.34 (dd, *J* = 8.6, 2.3 Hz, 1H), 6.26 (d, *J* = 2.2 Hz, 1H), 5.99 (q, *J* = 1.2 Hz, 1H), 4.23 (s, 4H), 2.33 (d, *J* = 1.2 Hz, 3H), 1.48 (s, 18H); ^{13}C NMR ($CDCl_3$, 101 MHz) δ: 168.2 (C), 161.8 (C), 155.5 (C), 153.1 (C), 153.0 (C), 125.6 (CH), 111.3 (C), 110.3 (CH), 108.2 (CH), 98.1 (CH), 82.9 (C), 57.1 (CH_2), 50.1 (C), 27.9 (CH_3), 18.7 (CH_3). HRMS (ESI) calcd for $C_{23}H_{30}NO_6$ $[M+H]^+$ 416.2068, found 416.2062.

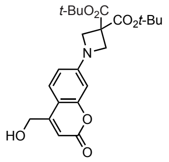

Di-*tert*-butyl 1-(4-(hydroxymethyl)-2-oxo-2H-chromen-7-yl)azetidine-3,3-dicarboxylate (S4). Compound **S3** (1.65 g, 3.97 mmol, 1 equiv) and SeO_2 (0.53 g, 4.77 mmol, 1.2 equiv) were suspended in 20 mL dioxane, and the reaction mixture was heated to reflux for 24 h. The reaction was cooled to room temperature and then concentrated under reduced pressure. The crude material was dissolved in CH_3OH (20 mL) after which $NaBH_4$ (150 mg, 3.97 mmol, 1 equiv) was added. The reaction was stirred for 1 h at ambient temperature, quenched by addition of saturated NH_4Cl(aq) (1 mL), and then concentrated under reduced pressure. Flash chromatography on silica gel (0→80% v/v EtOAc/hexanes, linear gradient; dry load with Celite) provided **S4** as a yellow solid (1.12 g, 65%). ^1H NMR (400 MHz, $CDCl_3$) δ: 7.29 (d, *J* = 8.6 Hz, 1H), 6.33 (t, *J* = 1.4 Hz, 1H), 6.29 (dd, *J* = 8.7, 2.3 Hz, 1H), 6.25 (d, *J* = 2.3 Hz, 1H), 4.81 (d, *J* = 1.4 Hz, 2H), 4.22 (s, 4H), 1.49 (s, 18H); ^{13}C NMR ($CDCl_3$, 101 MHz) δ: 168.2 (C), 162.2 (C), 155.5 (C), 154.9 (C), 152.9 (C), 124.4 (CH), 108.6 (C), 108.4 (CH), 107.0 (CH), 98.2 (C), 83.0 (C), 61.0 (CH_2), 57.1 (CH_2), 50.1 (C), 28.0 (CH_3). HRMS (ESI) calcd for $C_{23}H_{30}NO_7$ $[M+H]^+$ 432.2017, found 432.2015.

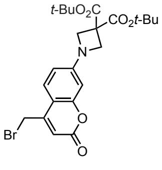

Di-*tert*-butyl 1-(4-(bromomethyl)-2-oxo-2H-chromen-7-yl)azetidine-3,3-dicarboxylate (S5). Compound **S4** (205 mg, 0.475 mmol, 1 equiv) was dissolved in anhydrous CH_2Cl_2 (10 mL). Et_3N (200 µL, 1.43 mmol, 3 equiv) was added and the resulting solution was cooled to 0 °C using an ice bath. Methanesulfonyl chloride (55 µL, 0.71 mmol, 1.5 equiv) was added, and the reaction was stirred at 0 °C for 30 min and at ambient temperature for 2 h. The reaction mixture was washed with cold 5% w/v $NaHCO_3$(aq) and saturated NaCl(aq). The organic layer was separated, dried over $MgSO_4$(s), and concentrated under reduced pressure. The crude material was dissolved in anhydrous THF (10 mL), to which LiBr (165 mg, 1.90 mmol, 4 equiv) was added. This reaction mixture was stirred at ambient temperature for 2 h. The reaction was concentrated under reduced pressure and the crude material was dissolved in CH_2Cl_2 (30 mL). This solution was washed with H_2O and saturated NaCl(aq). The organic layer was dried over $MgSO_4$(s) and concentrated under reduced pressure to afford **S5** as a yellow solid. The product was used in the next step without further purification. Yield: 99% (quantitative). ^{1}H NMR ($CDCl_3$, 400 MHz) δ: 7.52 (d, J = 8.7 Hz, 1H), 6.38 (dd, J = 8.7, 2.3 Hz, 1H), 6.28 (d, J = 2.3 Hz, 1H), 6.21 (s, 1H), 4.40 (s, 2H), 4.26 (s, 4H), 1.50 (s, 18H); ^{13}C NMR ($CDCl_3$, 101 MHz) δ: 168.1 (C), 161.3 (C), 156.2 (C), 153.3 (C), 150.4 (C), 125.5 (CH), 111.0 (CH), 108.4 (CH), 108.3 (C), 98.2 (CH), 83.0 (C), 57.1 (CH_2), 50.1 (C), 28.0 (CH_3), 27.2 (CH_2). HRMS (ESI) calcd for $C_{23}H_{29}BrNO_6$ $[M+H]^+$ 494.1173, found 494.1167 and 494.1145.

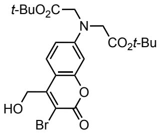

Di-*tert*-butyl 2,2'-((3-bromo-4-(hydroxymethyl)-2-oxo-2H-chromen-7-yl)azanediyl) diacetate (S7). Di-*tert*-butyl 2,2'-((4-(hydroxymethyl)-2-oxo-2H-chromen-7-yl)-azanediyl)diacetate[6] (**S6**; 100 mg, 0.24 mmol, 1 equiv) was dissolved in CH_3CN (5 mL). *N*-bromosuccinimide (NBS; 42.5 mg, 0.24 mmol, 1 equiv) was added. The reaction was stirred at ambient temperature for 15 h. The reaction was concentrated under reduced pressure. Flash chromatography on silica gel (0→50% v/v EtOAc/hexanes, linear gradient; dry load with Celite) provided **S7** as a yellow solid (90 mg, 76%). ^{1}H NMR ($CDCl_3$, 400 MHz) δ: 7.73 (d, J = 9.0 Hz, 1H), 6.57 (dd, J = 9.0, 2.7 Hz, 1H), 6.46 (d, J = 2.6 Hz, 1H), 4.98 (s, 2H), 4.05 (s, 4H), 1.48 (s, 18H); ^{13}C NMR ($CDCl_3$, 101 MHz) δ: 168.9 (C), 158.1 (C), 154.7 (C), 151.6 (C), 150.8 (C), 126.8 (CH), 110.0 (CH), 109.8 (C), 107.8 (C), 99.0 (CH), 82.8 (C), 61.9 (CH_2), 54.5 (CH_2), 28.2 (CH_3). HRMS (ESI) calcd for $C_{22}H_{28}BrNO_7$ $[M+Na]^+$ 520.0947, found 520.0932 and 522.0911.

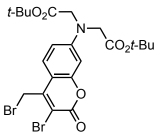

Di-*tert*-butyl 2,2'-((3-bromo-4-(bromomethyl)-2-oxo-2H-chromen-7-yl)azanediyl)diacetate (S8). Compound **S7** (45 mg, 90.3 µmol, 1 equiv) was dissolved in anhydrous CH_2Cl_2 (5 mL). Et_3N (38 µL, 271 µmol, 3 equiv) was added and the resulting solution was cooled to 0 °C using an ice bath. Methanesulfonyl chloride (MsCl; 11 µL, 135 µmol, 1.4 mmol, 1.4 equiv) was added, and the reaction was stirred at 0 °C for 30 min and at ambient temperature for 2 h. The reaction mixture was washed with cold 5% w/v $NaHCO_3$(aq) and saturated NaCl(aq). The organic layer was separated, dried over $MgSO_4$(s), and concentrated under reduced pressure. The crude material was dissolved in anhydrous THF (10 mL), to which LiBr (31.4 mg, 361 µmol, 4 equiv) was added. This reaction mixture was stirred at ambient temperature for 2 h. The reaction was concentrated under reduced pressure and the crude material was dissolved in CH_2Cl_2 (20 mL). This solution was washed with H_2O and saturated NaCl(aq). The organic layer was dried over $MgSO_4$(s) and concentrated under reduced pressure to afford afford **S8** as a yellow solid. The product was used in the next step without further purification. Yield: 99%

(quantitative). ^{1}H NMR ($CDCl_3$, 400 MHz) δ 7.52 (d, *J* = 9.1 Hz, 1H), 6.60 (dd, *J* = 9.1, 2.7 Hz, 1H), 6.46 (d, *J* = 2.6 Hz, 1H), 4.64 (s, 2H), 4.06 (s, 4H), 1.48 (s, 18H); ^{13}C NMR ($CDCl_3$, 101 MHz) δ 168.8 (C), 157.7 (C), 154.6 (C), 151.7 (C), 148.7 (C), 125.9 (CH), 110.0 (CH), 108.6 (C), 108.5 (C), 99.2 (CH), 82.8 (C), 54.4 (CH_2), 28.2 (CH_3), 26.6 (CH_2). HRMS (ESI) calcd for $C_{22}H_{28}Br_2NO_6$ $[M+Na]^+$ 582.0103, found 582.0087.

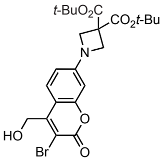

Di-*tert*-butyl 1-(3-bromo-4-(hydroxymethyl)-2-oxo-2H-chromen-7-yl)azetidine-3,3-dicarboxylate (S9). Compound **S4** (1.10 g, 2.55 mmol, 1 equiv) was dissolved in CH_3CN (20 mL). *N*-bromosuccinimide (NBS; 454 mg, 2.55 mmol, 1 equiv) was added slowly over 10 min. The reaction was stirred at ambient temperature for 30 min. The reaction was concentrated under reduced pressure. Flash chromatography on silica gel (0→50% v/v EtOAc/hexanes, linear gradient; dry load with Celite) provided **S9** as a yellow solid (1.10 g, 85%). ^{1}H NMR ($CDCl_3$, 400 MHz) δ: 7.68 (d, *J* = 8.8 Hz, 1H), 6.33 (d, *J* = 8.9 Hz, 1H), 6.18 (d, *J* = 2.4 Hz, 1H), 4.92 (s, 2H), 4.23 (s, 4H), 2.66 (brs, 1H), 1.48 (s, 18H); ^{13}C NMR ($CDCl_3$, 101 MHz) δ: 168.1 (C), 158.1 (C), 154.4 (C), 152.9 (C), 151.2 (C), 126.8 (CH), 109.6 (CH), 108.9 (CH), 107.0 (C), 97.5 (CH), 83.0 (C), 61.8 (CH_2), 56.9 (CH_2), 50.0 (C), 27.9 (CH_3). HRMS (ESI) calcd for $C_{23}H_{29}BrNO_7$ $[M+H]^+$ 510.1122, found 510.1122 and 512.1102.

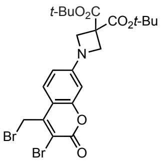

Di-*tert*-butyl 1-(3-bromo-4-(bromomethyl)-2-oxo-2H-chromen-7-yl)azetidine-3,3-dicarboxylate (32). Compound **S9** (1.0 g, 1.96 mmol, 1 equiv) was dissolved in anhydrous CH_2Cl_2 (20 mL). Et_3N (819 µL, 5.88 mmol, 3 equiv) was added and the resulting solution was cooled to 0 °C using an ice bath. Methanesulfonyl chloride (MsCl; 227 µL, 2.94 mmol, 1.5 equiv) was added, and the reaction was stirred at 0 °C for 30 min and at ambient temperature for 2 h. The reaction mixture was washed with cold 5% w/v $NaHCO_3$(aq) and saturated NaCl(aq). The organic layer was separated, dried over $MgSO_4$(s), and concentrated under reduced pressure. The crude material was dissolved in anhydrous THF (20 mL), to which LiBr (680 mg, 7.84 mmol, 4 equiv) was added. This reaction mixture was stirred at ambient temperature for 2 h. The reaction was concentrated under reduced pressure and the crude material was dissolved in CH_2Cl_2 (50 mL). This solution was washed with H_2O and saturated NaCl(aq). The organic layer was dried over $MgSO_4$(s) and concentrated under reduced pressure to afford afford **32** as a yellow solid. The product was used in the next step without further purification. Yield: 99% (quantitative). ^{1}H NMR ($CDCl_3$, 400 MHz) δ: 7.50 (d, *J* = 8.8 Hz, 1H), 6.41 (dd, *J* = 8.8, 2.3 Hz, 1H), 6.27 (d, *J* = 2.3 Hz, 1H), 4.65 (s, 2H), 4.26 (s, 4H), 1.50 (s, 18H); ^{13}C NMR ($CDCl_3$, 101 MHz) δ: 168.0 (C), 157.6 (C), 154.5 (C), 153.2 (C), 148.8 (C), 125.8 (CH), 109.0 (CH), 108.4 (C), 108.0 (C), 97.8 (CH), 83.1 (C), 56.7 (CH_2), 50.0 (C), 28.0 (CH_3), 26.8 (CH_2). HRMS (ESI) calcd for $C_{23}H_{27}Br_2NO_6$ $[M+Na]^+$ 594.0103, found 594.0088.

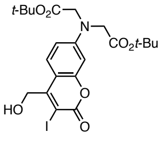

Di-*tert*-butyl 2,2'-((4-(hydroxymethyl)-3-iodo-2-oxo-2H-chromen-7-yl)azanediyl)diacetate (S10). Di-*tert*-butyl 2,2'-((4-(hydroxymethyl)-2-oxo-2H-chromen-7-yl)azanediyl)diacetate[6] (**S6**; 263 mg, 0.63 mmol, 1 equiv) was dissolved in CH_3CN (2 mL). *N*-iodosuccinimide (NIS; 128 mg, 0.57 mmol, 0.90 equiv) was added. The reaction was stirred at ambient temperature for 16 h. The reaction was concentrated under reduced pressure. Flash chromatography on silica gel (0→50% v/v EtOAc/hexanes, linear gradient; dry load with Celite) provided **S10** as a yellow solid (103 mg, 30%). ^{1}H NMR (CD_3CN, 400 MHz) δ: 7.78 (d, *J* = 9.1 Hz, 1H), 6.58 (dd, *J* = 9.1, 2.7 Hz, 1H), 6.45 (d, *J* = 2.7 Hz, 1H), 4.87 (d, *J* = 5.9 Hz, 2H), 4.08 (s, 4H),

3.59 (t, J = 6.0 Hz, 1H), 1.45 (s, 18H); ^{13}C NMR (101 MHz, CD_3CN) δ: 169.8 (C), 159.4 (C), 157.5(C), 156.0(C), 152.5 (C), 128.1 (CH), 110. 2 (CH), 98.7 (CH), 86.2 (C), 82.6 (C), 67.4 (CH_2), 54.6 (CH_2), 28.2 (CH_3). HRMS (ESI) calcd for $C_{22}H_{29}INO_7$ $[M+H]^+$ 546.0983, found 546.0982.

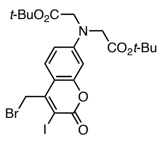

Di-*tert*-butyl 2,2'-((4-(bromomethyl)-3-iodo-2-oxo-2H-chromen-7-yl)azanediyl)diacetate (S11). Compound **S10** (100 mg, 0.18 mmol, 1 equiv) was dissolved in anhydrous CH_2Cl_2 (5 mL). Et_3N (77 µL, 0.55 µmol, 3 equiv) was added and the resulting solution was cooled to 0 °C using an ice bath. Methanesulfonyl chloride (MsCl; 21 µL, 0.27 mmol, 1.5 eq) was added, and the reaction was stirred at 0 °C for 30 min and at ambient temperature for 2 h. The reaction mixture was washed with cold 5% (w/v) $NaHCO_3$(aq) and saturated NaCl(aq). The organic layer was separated, dried over $MgSO_4$(s), and concentrated under reduced pressure. The crude material was dissolved in anhydrous THF (5 mL), to which LiBr (63 mg, 0.73 mmol, 4 equiv) was added. This reaction mixture was stirred at ambient temperature for 2 h. The reaction was concentrated under reduced pressure and the crude material was dissolved in CH_2Cl_2 (20 mL). This solution was washed with H_2O and saturated NaCl(aq). The organic layer was dried over $MgSO_4$(s) and concentrated under reduced pressure to afford **S11** as a yellow solid. The product was used in the next step without further purification. Yield: 99% (quantitative). ^{1}H NMR (CD_3CN, 400 MHz) δ: 7.64 (d, J = 9.0 Hz, 1H), 6.61 (dd, J = 9.1, 2.7 Hz, 1H), 6.49 (d, J = 2.7 Hz, 1H), 4.75 (s, 2H), 4.09 (s, 4H), 1.45 (s, 18H). ^{13}C NMR (CD_3CN, 101 MHz) δ: 169.7 (C), 159.2 (C), 156.0 (C), 155.0 (C), 153.0 (C), 127.2 (CH), 110.9, 110.5 (CH), 99.1 (CH), 87.7, 82.6, 54.6 (CH_2), 34.4 (CH_2), 28.2 (CH_3). HRMS (ESI) calcd for $C_{22}H_{28}BrINO_6$ $[M+H]^+$ 608.0139, found 608.0134 and 610.0115.

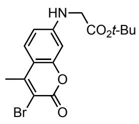

***Tert*-butyl (3-bromo-4-methyl-2-oxo-2H-chromen-7-yl)glycinate (S17).** *Tert*-butyl (4-methyl-2-oxo-2H-chromen-7-yl)glycinate[6] (**S16**; 138 mg, 0.48 mmol, 1 equiv) was dissolved in CH_3CN (5 mL). *N*-bromosuccinimide (NBS; 85 mg, 0.48 mmol, 1 equiv) was added. The reaction was stirred at ambient temperature for 15 h. The reaction was concentrated under reduced pressure. Flash chromatography on silica gel (0→40% v/v EtOAc/hexanes, linear gradient; dry load with Celite) provided **S17** as a yellow solid (165 mg, 94%). ^{1}H NMR ($CDCl_3$, 400 MHz) δ: 7.41 (d, J = 8.8 Hz, 1H), 6.56 (dd, J = 8.8, 2.4 Hz, 1H), 6.38 (d, J = 2.3 Hz, 1H), 3.83 (s, 2H), 2.53 (s, 3H), 1.50 (s, 9H); ^{13}C NMR ($CDCl_3$, 101 MHz) δ 169.2 (C), 157.9 (C), 154.3 (C), 151.6 (C), 150.3 (C), 126.3 (CH), 111.5 (CH), 111.2 (C), 107.4 (CH), 98.4 (C), 83.1 (C), 45.8 (CH_2), 28.2 (CH_3), 19.4 (CH_3). HRMS (ESI) calcd for $C_{16}H_{19}BrNO_4$ $[M+H]^+$ 368.0492, found 368.0479 and 370.0457.

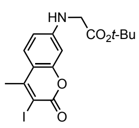

***Tert*-butyl (3-iodo-4-methyl-2-oxo-2H-chromen-7-yl)glycinate (S18):** *Tert*-butyl (4-methyl-2-oxo-2H-chromen-7-yl)glycinate (**S16**; 289 mg, 1 mmol; 1 equiv) was dissolved in CH_3CN (10 mL). *N*-iodosuccinimide (225 mg, 1 mmol, 1 equiv) was added. The reaction was stirred at ambient temperature for 15 h. The reaction was concentrated under reduced pressure. Flash chromatography on silica gel (0→40% v/v EtOAc/hexanes, linear gradient; dry load with Celite) provided **S18** as a yellow solid (158 mg, 38%). ^{1}H NMR ($CDCl_3$, 400 MHz) δ: 7.43 (d, J = 8.8 Hz, 1H), 6.51 (dd, J = 8.8, 2.4 Hz, 1H), 6.35 (d, J = 2.4 Hz, 1H), 4.92 (t, J = 5.0 Hz, 1H), 3.83 (d, J = 5.1 Hz, 2H), 2.58 (s, 3H), 1.50 (s, 9H). ^{13}C NMR ($CDCl_3$, 101 MHz) δ: 169.2 (C), 158.6 (C), 156.9 (C), 155.0 (C), 150.7 (C), 126.5 (CH), 111.2 (CH), 110.8 (C), 97.8 (CH), 85.6 (C), 83.0 (C), 45.7 (CH_2), 28.2 (CH_3), 25.3 (CH_3). HRMS (ESI) calcd for $C_{19}H_{19}INO_4$ $[M+H]^+$ 416.0353, found 416.0347.

General procedure for the reaction of pharmacological agents with bromomethyl coumarin derivatives. The following procedure for compound **S12** is representative. Escitalopram **3** (20 mg, 60.3 μmol, 1 equiv) was dissolved in anhydrous CH_3CN (5 mL). Bromomethyl coumarin **S5** (29.1 mg, 60.3 mmol, 1 equiv) was added, and the reaction was heated to 60 °C and stirred for 18 h. The reaction was cooled to room temperature and concentrated under reduced pressure. Purification by HPLC (5→95% v/v CH_3CN/H_2O, linear gradient, constant 0.1% v/v TFA additive) and lyophilization afforded the desired product **S12** as pale-yellow powder (35 mg, 70%).

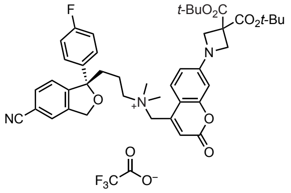

Compound S12. This compound was prepared using escitalopram (**3**) and bromomethyl coumarin **S5** according to the general procedure described above. Method for reverse phase HPLC: 25→95% v/v CH_3CN/H_2O, linear gradient, with constant 0.1% v/v TFA additive. Yield: 70%, pale yellow powder. ^{1}H NMR (CD_3CN, 400 MHz) δ: 7.70 – 7.65 (m, 3H), 7.55 – 7.51 (m, 3H), 7.13 – 7.08 (m, 2H), 6.48 (dd, J = 8.8, 2.3 Hz, 1H), 6.39 (d, J = 2.2 Hz, 1H), 6.23 (s, 1H), 5.19 (AB quartet, ν_A = 2057.5 Hz, ν_B = 2025.7 Hz, J_{AB} =13.3 Hz, 2H), 4.41 (s, 2H), 4.28 (s, 4H), 3.44 – 3.32 (m, 2H), 2.93 (s, 3H), 2.92 (s, 3H), 2.21 – 2.14 (m, 2H), 1.82 – 1.71 (m, 1H), 1.67 – 1.57 (m, 1H), 1.47 (s, 18H). ^{13}C NMR (CD_3CN, 101 MHz) δ: 169.0 (C), 160.7 (C), 157.1 (C), 154.5 (C), 149.6 (C), 142.4 (C), 141.3 (C), 133.1 (CH), 128.0 (2CH), 127.2 (CH), 126.8 (CH), 123.9 (CH), 119.6 (C), 116.4 (CH), 116.2 (CH), 112.6 (C), 110.3 (C), 109.5 (CH), 98.8 (CH), 91.4 (CH), 83.7 (C), 72.4 (CH_2), 66.6 (CH_2), 62.9 (CH_2), 57.6 (CH_2), 51.5 (CH_3), 50.8 (C), 37.8 (CH_2), 28.0 (CH_3), 18.9 (CH_2). HRMS (ESI) calcd for $C_{43}H_{49}FN_3O_7$ $[M]^+$ 738.3555, found 738.3541.

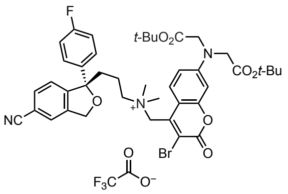

Compound S13. This compound was prepared using escitalopram (**3**) and bromomethyl coumarin **S8** according to the general procedure described above for compound **S12**. Method for reverse phase HPLC: 5→80% v/v CH_3CN/H_2O, linear gradient, with constant 0.1% v/v TFA additive. Yield: 68%, pale yellow powder. ^{1}H NMR (CD_3CN, 400 MHz) δ: 7.71 – 7.65 (m, 3H), 7.56 – 7.52 (m, 3H), 7.13 – 7.08 (m, 2H), 6.63 (dd, J = 9.2, 2.7 Hz, 1H), 6.55 (d, J = 2.7 Hz, 1H), 5.23 – 5.14 (m, 2H), 4.86 – 4.56 (m, 2H), 4.11 (s, 4H), 3.60 – 3.44 (m, 2H), 2.99 (s, 6H), 2.26 – 2.12 (m, 2H), 1.86 – 1.73 (m, 1H), 1.71 – 1.60 (m, 1H), 1.44 (s, 18H). ^{13}C NMR (CD_3CN, 101 MHz) δ: 169.6 (C), 164.3 (C), 161.9 (C), 157.9 (C), 155.3 (C), 153.1 (C), 149.6 (C), 141.3 (2C), 140.7 (C), 133.2 (CH), 128.1 (2CH), 128.0 (CH), 126.8 (CH), 123.9 (CH), 119.6 (C), 116.5 (C), 116.4 (CH), 116.2 (CH), 112.6 (CH), 110.8 (CH_3), 99.6 (CH), 91.4 (CH), 82.4 (C), 72.4 (CH_2), 67.8 (CH_2), 63.7 (CH_2), 54.9 (CH_2), 37.8 (CH_2), 28.2 (CH_3), 18.5 (CH_2). HRMS (ESI) calcd for $C_{42}H_{48}BrFN_3O_7$ $[M]^+$ 804.2660, found 804.2646 and 806.2630.

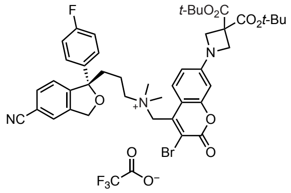

Compound S14. This compound was prepared using using escitalopram (**3**) and bromomethyl coumarin **32** according to the general procedure described above for compound **S12**. Method for reverse phase HPLC: 25→95% v/v CH_3CN/H_2O, linear gradient, with constant 0.1% v/v TFA additive. Yield: 65%, pale yellow powder. ^{1}H NMR (CD_3CN, 400 MHz) δ: 7.71 – 7.64 (m, 3H), 7.57 – 7.52 (m, 3H), 7.14 – 7.09 (m, 2H), 6.49 (dd, J = 8.9, 2.3 Hz, 1H), 6.41 (d, J = 2.3 Hz, 1H), 5.24 – 5.15 (m, 2H), 4.78 – 4.64 (m, 2H), 4.28 (s, 4H), 3.58 – 3.42 (m, 2H), 2.98 (s, 6H), 2.24 – 2.15 (m, 2H), 1.84 – 1.60 (m, 2H), 1.47 (s, 18H). ^{13}C NMR (CD_3CN, 101

MHz) δ: 168.9 (C), 161.2 (C), 157.8 (C), 155.3 (C), 154.3 (C), 149.5 (C), 141.5 (C), 141.3 (C), 140.6 (C), 133.1 (CH), 128.1 (2CH), 128.0 (CH), 126.8 (CH), 123.9 (CH), 119.6 (C), 116.4 (CH), 116.2 (CH), 115.9 (C), 112.6 (C), 110.8 (C), 109.8 (CH), 98.3 (CH), 91.4 (C), 83.7 (C), 72.4 (CH_2), 67.8 (CH_2), 63.6 (CH_2), 57.6 (CH_2), 50.8 (CH_3), 37.8 (CH_2), 28.0 (CH_3), 19.0 (CH_2). HRMS (ESI) calcd for $C_{43}H_{48}BrFN_3O_7$ $[M]^+$ 816.2654, found 816.2649 and 818.2622.

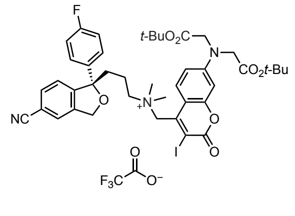

Compound S15. This compound was prepared using using escitalopram (**3**) and bromomethyl coumarin **S11** according to the general procedure described above for compound **S12**. Method for reverse phase HPLC: 20→95% v/v CH_3CN/H_2O, linear gradient, with constant 0.1% v/v TFA additive. Yield: 74%, pale yellow powder. ^1H NMR (CD_3CN, 400 MHz) δ: 7.72 – 7.67 (m, 2H), 7.65 (s, 1H), 7.58 – 7.51 (m, 2H), 7.17 – 7.04 (m, 2H), 6.59 (dd, *J* = 9.2, 2.7 Hz, 1H), 6.53 (d, *J* = 2.6 Hz, 1H), 5.28 – 5.12 (m, 2H), 4.99 (*d*, J = 13.9 Hz, 1H), 4.54 (d, *J* = 13.8 Hz, 1H), 4.10 (s, 4H), 3.63 – 3.45 (m, 2H), 3.04 (d, *J* = 7.1 Hz, 3H), 2.98 (*d*, J = 5.44 Hz, 3H), 2.28 – 2.14 (m, 2H), 1.87 – 1.61 (m, 2H), 1.44 (s, 18H); HRMS (ESI) calcd for $C_{42}H_{48}FIN_3O_7$ $[M]^+$ 852.2515, found 852.2505.

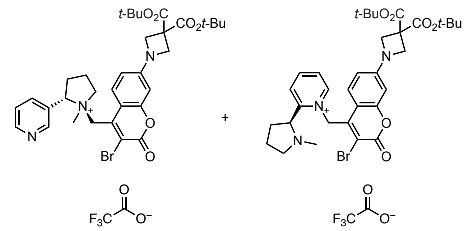

Compounds S19 and S20: These compounds were prepared using nicotine (**8**) and bromomethyl coumarin **32** according to the general procedure described above for compound **S12**. This reaction yielded two isomers, compound **S19** (3%) and **S20** (45%); the diastereomer of **S19** was not isolated. Method for reverse phase HPLC: 05→50% v/v CH_3CN/H_2O, linear gradient, with constant 0.1% v/v TFA additive. Yield: 48 % (pale yellow powder). **Compound S19.** ^1H NMR (CD_3OD 400 MHz) δ: 8.99 (s, 1H), 8.87 (s, 1H), 8.33 (d, *J* = 7.8 Hz, 1H), 7.95 – 7.60 (m, 1H), 7.73 (s, 1H), 6.67 – 6.39 (m, 1H), 6.47 (s, 1H), 5.38– 5.21 (m, 1H), 5.14– 5.02 (m, 1H), 4.76 – 4.65 (m, 1H), 4.28 (s, 4H), 4.21 – 4.10 (m, 1H), 3.84– 3.54 (m, 1H), 3.20 (s, 1H), 2.88 (s, 2H), 2.82– 2.63 (m, 2H), 2.50– 2.38 (m, 1H), 2.37 – 2.24 (m, 1H), 1.50 (s, 18H). HRMS (ESI) calcd for $C_{33}H_{41}BrN_3O_6$ $[M]^+$ 654.2173, found 654.2165 and 656.2138. **Compound S20.** ^1H NMR (CD_3CN, 400 MHz) δ: 9.34 (s, 1H), 8.82 (d, *J* = 8.1 Hz, 1H), 8.71 (d, *J* = 6.1 Hz, 1H), 8.04 (dd, *J* = 8.1, 6.1 Hz, 1H), 7.60 (d, *J* = 8.7 Hz, 1H), 6.46 – 6.37 (m, 2H), 6.12 (s, 2H), 4.50 – 4.41 (m, 1H), 4.26 (s, 4H), 3.83 – 3.74 (m, 1H), 3.18 – 3.04 (m, 1H), 2.65 (s, 3H), 2.59 – 2.50 (m, 1H), 2.31 – 2.13 (m, 3H), 1.46 (s, 18H); ^{13}C NMR (CD_3CN, 101 MHz) δ: 168.9 (C), 157.8 (C), 155.7 (C), 154.5 (C), 149.4 (C), 148.1 (CH), 146.8 (CH), 145.4 (CH), 143.6 (C), 129.9 (CH), 127.0 (CH), 113.3 (C), 110.2 (CH), 109.7 (C), 98.4 (CH), 83.7 (C), 60.7 (CH_2), 57.5 (CH_2), 57.1 (CH_2), 50.7 (C), 39.3 (CH_3), 28.0 (CH_3), 22.7 (CH_2). HRMS (ESI) calcd for $C_{33}H_{41}BrN_3O_6$ $[M]^+$ 654.2173, found 654.2169 and 656.2148.

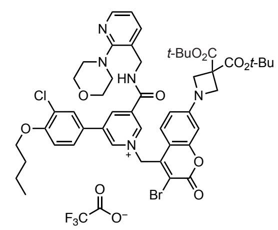

Compound S22. This compound was prepared using A 887626 (**S21**) and bromomethyl coumarin **32** according to the general procedure described above for compound **S12**. Method for reverse phase HPLC: 5→95% v/v CH_3CN/H_2O, linear gradient, with constant 0.1% v/v TFA additive. Yield: 70%, pale yellow powder. ^1H NMR (CD_3CN, 400 MHz) δ: 9.14 (dd, *J* = 3.6, 1.7 Hz, 2H), 9.06 (t, *J* = 5.3 Hz, 1H), 8.97 (s, 1H), 8.22 (dd, *J* = 5.0, 1.8 Hz, 1H), 7.85 (d, *J* = 2.4 Hz, 1H), 7.74 (dd, *J* = 7.6, 1.8 Hz, 1H), 7.70 (dd, *J* = 8.7, 2.4 Hz,

1H),7.62 (d, J = 8.8 Hz, 1H), 7.22 (d, J = 8.7 Hz, 1H), 7.01 (dd, J = 7.6, 4.9 Hz, 1H), 6.46 (dd, J = 8.8, 2.3 Hz, 1H), 6.41 (d, J = 2.3 Hz, 1H), 6.13 (s, 2H), 4.62 (d, J = 5.6 Hz, 2H), 4.27 (s, 4H), 4.15 (t, J = 6.5 Hz, 1H), 3.80 – 3.73 (m, 4H), 3.12 – 3.07 (m, 4H), 1.85 – 1.76 (m, 2H), 1.57 – 1.48 (m, 2H), 1.46 (s, 18H), 0.99 (t, J = 7.4 Hz, 3H); ^{13}C NMR (CD_3CN, 101 MHz) δ: 168.9 (C), 162.2 (C), 161.3 (C), 161.0 (C), 160.6 (C), 157.9 (C), 157.6 (C), 155.7 (C), 154.5 (C), 146.9 (CH), 144.0 (CH), 143.5 (C), 143.0 (CH), 142.9 (CH), 141.1 (C), 138.8 (CH), 136.1 (C), 130.3 (CH), 128.9 (CH), 126.9 (CH), 126.8 (C), 126.1 (C), 124.3 (C), 119.6 (CH), 116.1 (C), 115.1 (CH), 113.5 (C), 110.2 (CH), 109.9 (C), 98.4 (CH), 83.7 (C), 70.1 (CH_2), 67.6 (CH_2), 61.1 (CH_2), 57.5 (CH_2), 51.8 (CH_2), 50.7 (C), 40.5 (CH_2), 31.7 (CH_2), 28.0 (CH_3), 19.8 (CH_2). 14.0 (CH_3); HRMS (ESI) calcd for $C_{49}H_{56}BrClN_5O_9$ $[M]^+$ 972.2944, found 972.2945 and 974.2929.

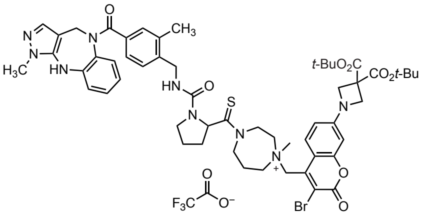

Compound S23. This compound was prepared using TC OT 39 (**36**) and bromomethyl coumarin **32** according to the general procedure described above for compound **S12**. Method for reverse phase HPLC: 5→95% v/v CH_3CN/H_2O, linear gradient, with constant 0.1% v/v TFA additive. Yield: 70%, pale yellow powder. ^{1}H NMR (DMSO-d_6, 400 MHz) δ: 8.61 (s, 2H), 8.07 – 7.87 (m, 1H), 7.28 (d, J = 8.1 Hz, 1H), 7.17 (s, 1H), 7.13 – 6.79 (m, 4H), 6.76 – 6.59 (m, 2H), 6.58 – 6.42 (m, 2H), 5.66 (d, J = 14.5 Hz, 1H), 5.21 – 4.47 (m, 5H), 4.42 – 4.18 (m, 5H), 4.17 – 3.84 (m, 8H), 3.66 – 3.31 (m, 5H), 3.27 – 2.90 (m, 3H), 2.31 – 1.96 (m, 5H), 1.97 – 1.66 (m, 2H), 1.44 (s, 14H); HRMS (ESI) calcd for $C_{55}H_{67}BrN_9O_8S$ $[M]^+$ 1092.4011, found 1092.4002 and 1094.3989.

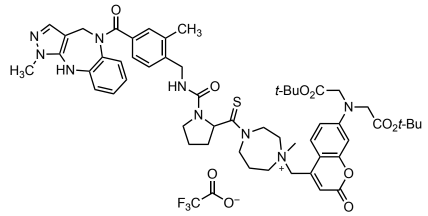

Compound S25. This compound was prepared using using TC OT 39 (**36**) and bromomethyl coumarin **S24**[1] according to the general procedure described above for compound **S12**. Method for reverse phase HPLC: 5→95% v/v CH_3CN/H_2O, linear gradient, with constant 0.1% v/v TFA additive. Yield: 70%, pale yellow powder. ^{1}H NMR (DMSO-d_6, 400 MHz) δ: 8.60 (s, 2H), 7.98 – 7.81 (m, 1H), 7.28 (d, J = 7.7 Hz, 1H), 7.16 (s, 1H), 7.13 – 6.80 (m, 4H), 6.70 – 6.52 (m, 3H), 6.52 – 6.38 (m, 2H), 5.67 (d, J = 14.6 Hz, 1H), 5.05 – 4.92 (m, 1H), 4.86 – 4.35 (m, 4H), 4.30 – 4.00 (m, 7H), 3.96 – 3.78 (m, 5H), 3.73 – 3.45 (m, 11H), 3.23 – 2.94 (m, 3H), 2.30 – 2.01 (m, 5H), 1.96 – 1.67 (m, 2H), 1.41 (s, 15H); HRMS (ESI) calcd for $C_{54}H_{68}N_9O_8S$ $[M]^+$ 1002.4906, found 1002.4905.

General procedure for deprotection of *tert*-butyl groups. The following procedure for compound **17** is representative. To deprotect the *tert*-butyl ester groups, compound **S12** (33 mg, 39.3 μmol, 1 equiv) was dissolved in CH_2Cl_2 (5 mL). Trifluoroacetic acid (TFA; 1 mL) was added and the reaction was stirred at ambient temperature for 2 h. The reaction mixture was concentrated under reduced pressure and purified by reverse phase HPLC (5→70% v/v CH_3CN/H_2O, linear gradient, constant 0.1% v/v TFA additive). Lyophilization of combined HPLC fractions yielded 23 mg (80%) of compound **17** as pale-yellow powder.

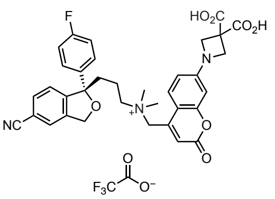

Compound 17. This compound was prepared from compound **S12** according to the general procedure described above. Method for reverse phase HPLC: 5→95% v/v CH_3CN/H_2O, linear gradient, with constant 0.1% v/v TFA additive. Yield: 80%, pale yellow powder. ^1H NMR (DMSO-d_6, 400 MHz) δ: 7.91 (d, J = 8.7 Hz, 1H), 7.83 – 7.75 (m, 3H), 7.61 – 7.57 (m, 2H), 7.18 (t, J = 8.9 Hz, 2H), 6.54 – 6.50 (m, 2H), 6.37 (s, 1H), 5.20 (AB quartet, ν_A = 2067.3 Hz, ν_B = 2039.9 Hz, J_{AB} =13.7 Hz, 2H), 4.57 (s, 2H), 4.28 (s, 4H), 3.44 (t, J = 8.4 Hz, 2H), 2.98 (s, 3H), 2.96 (s, 3H), 2.56 – 2.12 (m, 2H), 1.74 – 1.50 (m, 2H). ^{13}C NMR (DMSO-d_6, 101 MHz) δ: 170.5 (C), 160.2 (C), 159.7 (C), 158.0 (C) , 157.7 (C), 155.6 (C), 153.2 (C), 148.8 (C), 142.2 (C), 139.8 (2C), 133.2 (CH), 127.0 (2CH), 126.7 (CH), 125.8 (CH), 123.2 (CH), 118.8 (C), 117.0 (CH), 115.4 (CH), 115.2 (CH), 111.8 (C), 109.7 (C), 108.5 (CH), 97.7 (CH), 90.2 (CH_2), 71.2 (CH_2), 64.7 (CH_2), 60.9 (CH_2), 57.3 (CH_2), 49.9 (CH_3), 48.3 (CH_2), 36.9 (CH_2), 17.5 (CH_2). HRMS (ESI) calcd for $C_{35}H_{33}FN_3O_7$ $[M]^+$ 626.6609, found 626.2289.

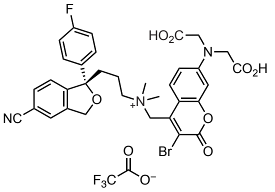

Compound 18. This compound was prepared from **S13** according to the general procedure described above for compound **17**. Method for reverse phase HPLC: 5→30% v/v CH_3CN/H_2O, linear gradient, with constant 0.1% v/v TFA additive. Yield: 75%, pale yellow powder. ^1H NMR (DMSO-d_6, 400 MHz) δ: 7.92 (d, J = 9.2 Hz, 1H), 7.85 – 7.75 (m, 3H), 7.65 – 7.53 (m, 2H), 7.24 – 7.11 (m, 2H), 6.62 (dd, J = 9.4, 2.6 Hz, 1H), 6.55 (d, J = 2.6 Hz, 1H), 5.30 – 5.07 (m, 2H), 4.97 (d, J = 13.7 Hz, 1H), 4.60 (d, J = 13.5 Hz, 1H), 4.25 (s, 4H), 3.68 – 3.50 (m, 2H), 3.03 (s, 6H), 2.30 – 2.12 (m, 2H), 1.77 – 1.51 (m, 2H). HRMS (ESI) calcd for $C_{34}H_{32}BrFN_3O_7$ $[M]^+$ 692.1402, found 692.1396 and 694.1375.

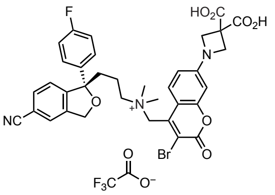

Compound 19. This compound was prepared from compound **S14** according to the general procedure described above for compound **17**. Method for reverse phase HPLC: 5→30% v/v CH_3CN/H_2O, linear gradient, with constant 0.1% v/v TFA additive. Yield: 70%, pale yellow powder. ^1H NMR (DMSO-d_6, 400 MHz) δ: 7.94 (d, J = 9.5 Hz, 1H), 7.87 – 7.72 (m, 3H), 7.66 – 7.55 (m, 2H), 7.18 (t, J = 8.8 Hz, 2H), 6.59 – 6.49 (m, 2H), 5.30 – 5.06 (m, 2H), 4.99 (d, J = 13.8 Hz, 1H), 4.60 (d, J = 13.8 Hz, 1H), 4.29 (s, 4H), 3.64 – 3.51 (s, 2H), 3.02 (s, 6H), 2.23 – 2.10 (m, 2H), 1.76 – 1.49 (m, 2H). ^{13}C NMR (DMSO-d_6, 101 MHz) δ 170.6 (C), 160.2 (C), 157.9 (C), 156.8 (C), 153.7 (C), 153.0 (C), 148.7 (C), 141.0 (C), 139.9 (C), 132.2 (CH), 127.7 (CH), 127.1 (CH), 127.0 (CH), 125.8 (CH), 123.2 (CH), 118.8 (C), 115.4 (CH), 115.2 (CH), 114.5 (C), 110.8 (C), 110.2 (C), 108.6 (CH), 97.2 (CH), 90.1 (C), 71.2 (CH_2), 65.5 (CH_2), 62.0 (CH_2), 57.3 (CH_2), 48.1 (CH_3), 37.1 (CH_2), 17.6 (CH2). HRMS (ESI) calcd for $C_{35}H_{32}BrFN_3O_7$ [M]+ 704.1402, found 704.1396 and 706.1378.

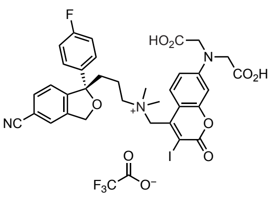

Compound 20: This compound was prepared from compound **S15** according to the general procedure described above for compound **17**. Method for reverse phase HPLC: 20→75% v/v CH_3CN/H_2O, linear gradient, with constant 0.1% v/v TFA additive. Yield: 80%, pale yellow powder. ^1H NMR (DMSO-d_6, 400 MHz) δ: 7.90 (d, J = 9.1 Hz, 1H), 7.86 – 7.73 (m, 3H), 7.66 – 7.51 (m, 2H), 7.19 (t, J = 8.8 Hz, 2H), 6.58 (d, J = 9.3 Hz, 1H), 6.53

(d, J = 2.6 Hz, 1H), 5.28 – 5.06 (m, 3H), 4.47 (d, J = 13.8 Hz, 1H), 4.25 (s, 4H), 3.69 – 3.54 (m, 2H), 3.10 – 2.90 (m, 6H), 2.28 – 2.13 (m, 2H), 1.80 – 1.52 (m, 2H); HRMS (ESI) calcd for $C_{34}H_{32}FIN_3O_7$ $[M]^+$ 740.1263, found 740.1252.

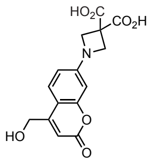

1-(4-(hydroxymethyl)-2-oxo-2H-chromen-7-yl)azetidine-3,3-dicarboxylic acid (21). This compound was prepared from **S4** according to the general procedure described above for compound **17** Method for reverse phase HPLC: 5→50% v/v CH_3CN/H_2O, linear gradient, with constant 0.1% v/v TFA additive. Yield: 65%, pale yellow powder. ^1H NMR (DMSO-d_6, 400 MHz) δ: 7.49 (d, J = 8.6 Hz, 1H), 6.48 (dd, J = 8.6, 2.3 Hz, 1H), 6.44 (d, J = 2.3 Hz, 1H), 6.16 (d, J = 1.5 Hz, 1H), 5.52 (s, 1H), 4.68 (d, J = 1.6 Hz, 2H), 4.23 (s, 4H); ^{13}C NMR (DMSO-d_6, 101 MHz) δ: 170.6 (C), 160.8 (C), 156.9 (C), 154.9 (C), 153.0 (C), 125.0 (CH), 108.4 (CH), 107.9 (C), 105.3 (CH), 97.4 (CH), 59.1 (CH_2), 57.4 (CH_2), 48.2 (C). HRMS (ESI) calcd for $C_{15}H_{14}NO_7$ $[M–H]^-$ 318.0619, found 318.0613.

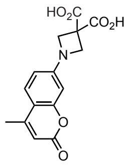

1-(4-methyl-2-oxo-2H-chromen-7-yl)azetidine-3,3-dicarboxylic acid (22). This compound was prepared from **S3** according to the general procedure described above for compound **17**. Method for reverse phase HPLC: 5→70% v/v CH_3CN/H_2O, linear gradient, with constant 0.1% v/v TFA additive. Yield: 70%, pale yellow powder. ^1H NMR (DMSO-d_6, 400 MHz) δ: 7.55 (d, J = 8.6 Hz, 1H), 6.51 (dd, J = 8.7, 2.3 Hz, 1H), 6.42 (d, J = 2.2 Hz, 1H), 6.03 (d, J = 1.3 Hz, 1H), 4.24 (s, 4H), 2.34 (d, J = 1.2 Hz, 3H); (DMSO-d_6, 101 MHz) δ: 170.5 (C), 160.4 (C), 154.9 (C), 153.7 (C), 153.2 (C), 126.1 (CH), 110.4 (C), 109.0 (CH), 108.4 (CH), 97.4 (CH), 57.3 (CH_2), 48.3 (C), 18.1 (CH_3). HRMS (ESI) calcd for $C_{15}H_{13}NO_6$ $[M–H]^-$ 302.0670, found 302.0663.

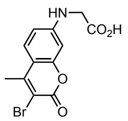

(3-bromo-4-methyl-2-oxo-2H-chromen-7-yl)glycine (23). This compound was prepared from **S17** according to the general procedure described above for compound **17**. Method for reverse phase HPLC: 5→40% v/v CH_3CN/H_2O, linear gradient, with constant 0.1% v/v TFA additive. Yield: 70%, pale yellow powder. ^1H NMR (DMSO-d_6, 400 MHz) δ: 7.57 (d, J = 8.9 Hz, 1H), 7.02 (t, J = 6.1 Hz, 1H), 6.69 (dd, J = 8.9, 2.3 Hz, 1H), 6.45 (d, J = 2.3 Hz, 1H), 3.94 (d, J = 5.9 Hz, 2H), 3.34 (s, 9H), 2.49 (s, 3H); ^{13}C NMR (DMSO-d_6, 101 MHz) δ: 171.7 (C), 156.9 (C), 153.9 (C), 152.4 (C), 126.7 (CH), 111.1 (CH), 109.1 (C), 104.6 (C), 96.6 (CH), 44.1 (CH_2), 19.1 (CH_3). HRMS (ESI) calcd for $C_{12}H_9BrNO_4$ $[M–H]^-$ 309.9720, found 309.9718 and 311.9695.

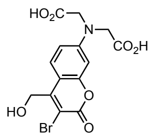

1-(3-bromo-4-(hydroxymethyl)-2-oxo-2H-chromen-7-yl)azetidine-3,3-dicarboxylic acid (24). This compound was prepared from **S7** according to the general procedure described above for compound **17**. Method for reverse phase HPLC: 5→95% v/v CH_3CN/H_2O, linear gradient, with constant 0.1% v/v TFA additive. Yield: 60%, pale yellow powder. ^1H NMR (DMSO-d_6, 400 MHz) δ: 7.81 (d, J = 8.9 Hz, 1H), 6.56 (dd, J = 8.9, 2.3 Hz, 1H), 6.48 (d, J = 2.3 Hz, 1H), 5.67 (s, 1H), 4.79 (s, 2H), 4.25 (s, 4H). ^{13}C NMR (DMSO-d_6, 101 MHz) δ: 170.6 (C), 157.1 (C), 153.9 (C), 153.0 (C), 152.5 (C), 127.5 (CH), 109.1 (C), 108.9 (CH), 105.5 (C), 96.9 (CH), 60.6 (CH_2), 57.3 (CH_2), 48.1 (C). HRMS (ESI) calcd for $C_{15}H_{11}BrNO_7$ $[M–H]^-$ 395.9724, found 395.9718, 397.9695.

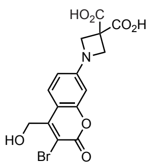

2,2'-((3-bromo-4-(hydroxymethyl)-2-oxo-2H-chromen-7-yl)azanediyl)diacetic acid (26). This compound was prepared from compound **S9** according to the general procedure described above for compound **17**. Method for reverse phase HPLC: 5→50% v/v CH_3CN/H_2O, linear gradient, with constant 0.1% v/v TFA additive. Yield: 70%, pale yellow powder. ^1H NMR (DMSO-d_6, 400 MHz) δ: 7.79 (d, J = 9.2 Hz, 1H), 6.64 (dd, J = 9.2, 2.6 Hz, 1H), 6.45 (d, J = 2.6 Hz, 1H), 5.66 (t, J = 5.5 Hz, 1H), 4.78 (d, J = 4.4 Hz, 2H), 4.21 (s, 4H); ^{13}C NMR (DMSO-d_6, 101 MHz) δ: 171.6 (C), 157.3 (C), 153.9 (C), 152.5 (C), 151.2 (C), 127.5 (CH), 109.5 (CH), 108.6 (C), 105.4 (C), 97.5 (CH), 60.5 (CH_2), 53.6 (CH_2). HRMS (ESI) calcd for $C_{14}H_{11}BrNO_7$ $[M–H]^-$ 383.9724, found 383.9721 and 385.9695.

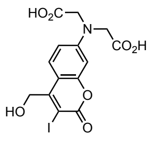

2,2'-((4-(hydroxymethyl)-3-iodo-2-oxo-2H-chromen-7-yl)azanediyl)diacetic acid (28). This compound was prepared from compound **S10** according to the general procedure described above for compound **17**. Method for reverse phase HPLC: 5–95% v/v CH_3CN/H_2O, linear gradient, with constant 0.1% v/v TFA additive. Yield: 90%, pale yellow powder. ^1H NMR (DMSO-d_6, 400 MHz) δ: 13.07 (s, 2H), 7.79 (d, J = 9.2 Hz, 1H), 6.61 (dd, J = 9.2, 2.6 Hz, 1H), 6.45 (d, J = 2.6 Hz, 1H), 5.64 (t, J = 5.6 Hz, 1H), 4.79 (d, J = 3.9 Hz, 2H), 4.21 (s, 4H). ^{13}C NMR (DMSO-d_6, 101 MHz) δ: 171.4 (C), 158.3 (C), 157.1 (C), 154.5 (C), 151.4 (C), 127.4 (CH), 109.3 (CH), 108.7 (C), 97.2 (CH), 85.1 (C), 66.0 (CH_2), 53.2 (CH_2). HRMS (ESI) calcd for $C_{14}H_{13}INO_7$ $[M+H]^+$ 433.9731, found 433.9727.

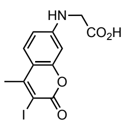

(3-iodo-4-methyl-2-oxo-2H-chromen-7-yl)glycine (29). This compound was prepared from **S18** according to the general procedure described above for the compound **17**. Method for reverse phase HPLC: 5→50% v/v CH_3CN/H_2O, linear gradient, with constant 0.1% v/v TFA additive. Yield: 30%, pale yellow powder. ^1H NMR (DMSO-d_6, 400 MHz) δ: 12.77 (s, 1H), 7.57 (d, J = 8.9 Hz, 1H), 7.01 (t, J = 4.4 Hz, 1H), 6.65 (dd, J = 8.9, 2.3 Hz, 1H), 6.42 (d, J = 2.3 Hz, 1H), 3.94 (d, J = 4.4 Hz, 2H), 2.55 (s, 3H). ^{13}C NMR (DMSO-d_6, 101 Hz) δ: 171.8 (C), 157.9 (C), 157.2 (C), 154.5 (C), 154.4 (C), 152.5 (C), 126.9 (CH), 110.9 (CH), 109.1 (C), 96.4 (CH), 84.0 (C), 44.1 (CH_2), 24.8 (CH_3). HRMS (ESI) calcd for $C_{12}H_{11}INO_4$ $[M+H]^+$ 359.9727, found 359.9721.

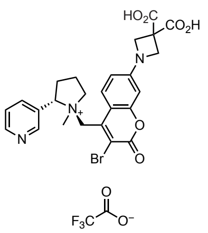

PANic2 (33). This compound was prepared from compound **S19** according to the general procedure described above for the compound **17**. Method for reverse phase HPLC: 5→30% v/v CH_3CN/H_2O, linear gradient, with constant 0.1% v/v TFA additive. Yield: 37%, pale yellow powder. ^1H NMR (CD_3CN, 400 MHz) δ: 8.87 (s, 1H), 8.80 (d, J = 4.8 Hz, 1H), 8.14 (d, J = 8.0 Hz, 1H), 7.80 – 7.58 (m, 1H), 7.63 (dd, J = 8.0, 4.8 Hz, 1H), 6.61 – 6.35 (m, 1H), 6.41 (s, 1H), 5.12 (t, J = 10.0 Hz, 1H), 4.88 (d, J = 13.7 Hz, 1H), 4.60 (d, J = 13.8 Hz, 1H), 4.34 (s, 4H), 2.74 (s, 3H), 2.65 – 2.59 (m, 2H), 2.42 – 2.28 (m, 1H), 2.27 – 2.14 (m, 1H). HRMS (ESI) calcd for $C_{25}H_{25}BrN_3O_6$ $[M]^+$ 542.0921, found 542.0916 and 544.0896.

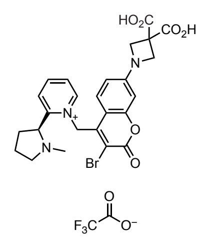

iPANic2 (34). This compound was prepared from compound **S20** according to the general procedure described above for the compound **17**. Method for reverse phase HPLC: 5→30% v/v CH_3CN/H_2O, linear gradient, with constant 0.1% v/v TFA additive. Yield: 65%, pale yellow powder. ^{1}H NMR (D_2O, 400 MHz) δ: 8.94 (s, 1H), 8.76 (d, *J* = 6.1 Hz, 1H), 8.63 (d, *J* = 8.2 Hz, 1H), 8.04 (dd, *J* = 8.2, 6.2 Hz, 1H), 7.34 (d, *J* = 9.0 Hz, 1H), 6.26 (dd, *J* = 8.9, 2.3 Hz, 1H), 5.97 (s, 2H), 5.93 (d, J = 2.3 Hz, 1H), 4.51 – 4.40 (m, 1H), 4.08 (s, 4H), 3.75 – 3.59 (m, 1H), 3.20 – 3.05 (m, 1H), 2.50 – 2.35 (m, 1H), 2.18 – 1.95 (m, 3H). ^{13}C NMR (D_2O, 101 MHz) δ: 172.1 (C), 162.6 (C), 162.3 (C), 161.9 (C), 161.6 (C), 158.8 (C), 153.8 (C), 153.4 (C), 146.7 (CH), 145.7 (CH), 144.6 (CH), 143.3 (C), 134.6 (C), 129.6 (CH), 125.3 (CH), 120.1 (C), 113.3 (C), 117.2 (C), 114.3 (C), 111.5 (C), 110.4 (C), 110.0 (CH), 108.7 (C), 97.3 (CH), 68.1 (CH), 59.3 (CH_2), 56.9 (CH_2), 56.5 (CH_2), 48.5 (C), 38.5 (CH_3), 30.7 (CH_2), 21.2 (CH_2); HRMS (ESI) calcd for $C_{25}H_{25}BrN_3O_6$ $[M]^+$ 542.0921, found 542.0919 and 544.0898.

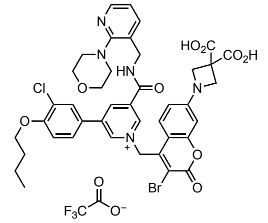

Compound 35. This compound was prepared from compound **S22** according to the general procedure described above for the compound **17**. Method for reverse phase HPLC: 5–70% v/v CH_3CN/H_2O, linear gradient, with constant 0.1% v/v TFA additive. Yield: 65%, pale yellow powder**.** ^{1}H NMR (CD_3CN with 20% CD_3OD, 400 MHz) δ: 9.08 – 9.01 (m, 2H), 8.91 (s, 1H), 8.19 (dd, *J* = 5.1, 1.8 Hz, 1H), 7.87 (d, *J* = 2.4 Hz, 1H), 7.76 (dd, *J* = 7.7, 1.8 Hz, 1H), 7.69 (dd, *J* = 8.7, 2.4 Hz, 1H), 7.58 (d, *J* = 8.9 Hz, 1H), 7.25 (d, *J* = 8.7 Hz, 1H), 7.06 (dd, *J* = 7.5, 5.0 Hz, 1H), 6.49 (dd, *J* = 8.9, 2.3 Hz, 1H), 6.44 (d, *J* = 2.2 Hz, 1H), 6.10 (s, 2H), 4.61 (s, 2H), 4.35 (s, 4H), 4.15 (t, *J* = 6.4 Hz, 2H), 3.81 – 3.74 (m, 4H), 3.16 – 3.09 (m, 4H), 1.84 – 1.77 (m, 2H), 1.55 – 1.49 (m, 2H), 0.98 (t, *J* = 7.4 Hz, 3H); HRMS (ESI) calcd for $C_{41}H_{40}BrClN_5O_9$ $[M]^+$ 860.1692, found 860.1700 and 862.1682.

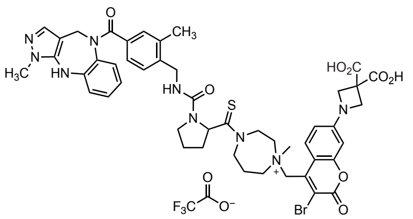

Compound 37. This compound was prepared from compound **S23** according to the general procedure described above for the compound **17**. Method for reverse phase HPLC: 5→50% v/v CH_3CN/H_2O, linear gradient, with constant 0.1% v/v TFA additive. Yield: 60%, pale yellow powder. ^{1}H NMR (DMSO-d_6, 400 MHz) δ: 8.61 (s, 2H), 8.05 – 7.84 (m, 1H), 7.28 (d, *J* = 8.1 Hz, 1H), 7.17 (s, 1H), 7.13 – 6.78 (m, 4H), 6.71 – 6.57 (m, 2H), 6.56 – 6.35 (m, 2H), 5.67 (d, *J* = 14.5 Hz, 1H), 5.25 – 4.44 (m, 5H), 4.42 – 4.17 (m, 5H), 4.17 – 3.74 (m, 11H), 3.58 – 3.34 (m, 5H), 3.26 – 2.90 (m, 3H), 2.35 – 1.99 (m, 5H), 1.97 – 1.66 (m, 2H); HRMS (ESI) calcd for $C_{47}H_{51}BrN_9O_8S$ $[M]^+$ 980.2759, found 980.2758 and 982.2743.

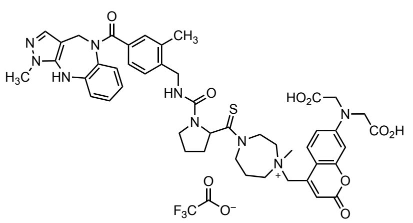

Compound 38. This compound was prepared from compound **S25** according to the general procedure described above for the compound **17**. Method for reverse phase HPLC: 5–70% v/v CH_3CN/H_2O, linear gradient, with constant 0.1% v/v TFA additive. Yield: 65%, pale yellow powder**.** ^{1}H NMR (DMSO-d_6, 400 MHz) δ: 8.60 (s, 2H), 7.97 – 7.78 (m, 1H), 7.32– 7.24 (m, 1H), 7.16 (s, 1H), 7.13 – 6.80 (m, 4H), 6.76 – 6.55 (m, 3H), 6.54 – 6.37 (m, 2H), 5.67 (d, *J* = 14.6 Hz, 1H), 5.05 – 4.92 (m, 1H), 4.87 – 4.36 (m, 4H), 4.34 – 4.00 (m, 7H), 3.93 – 3.81 (m, 3H), 3.80 – 3.61 (m, 8H), 3.46 – 3.32 (m, 3H), 3.22 – 2.98

(m, 3H), 2.30 – 1.99 (m, 5H), 1.967– 1.65 (m, 2H); HRMS (ESI) calcd for $C_{46}H_{52}N_9O_8S$ $[M]^+$ 890.3654, found 890.3652.

References

1. Banala, S.; Arvin, M. C.; Bannon, N. M.; Jin, X. T.; Macklin, J. J.; Wang, Y.; Peng, C.; Zhao, G.; Marshall, J. J.; Gee, K. R.; Wokosin, D. L.; Kim, V. J.; McIntosh, J. M.; Contractor, A.; Lester, H. A.; Kozorovitskiy, Y.; Drenan, R. M.; Lavis, L. D. Photoactivatable drugs for nicotinic optopharmacology. *Nat. Methods* **2018**, *15*, 347-350.

2. Yusa, K.; Zhou, L.; Li, M. A.; Bradley, A.; Craig, N. L. A hyperactive piggyBac transposase for mammalian applications. *Proc. Natl. Acad. Sci. U. S. A.* **2011**, *108*, 1531-1536.

3. Deo, C.; Sheu, S. H.; Seo, J.; Clapham, D. E.; Lavis, L. D. Isomeric tuning yields bright and targetable red Ca(2+) indicators. *J. Am. Chem. Soc.* **2019**, *141*, 13734-13738.

4. Ohtani, S.; Fujita, S.; Hasegawa, K.; Tsuda, H.; Tonogi, M.; Kobayashi, M. Relationship between the fluorescence intensity of rhodamine-labeled orexin A and the calcium responses in cortical neurons: An in vivo two-photon calcium imaging study. *J. Pharm. Sci.* **2018**, *138*, 76-82.

5. Grimm, J. B.; English, B. P.; Chen, J.; Slaughter, J. P.; Zhang, Z.; Revyakin, A.; Patel, R.; Macklin, J. J.; Normanno, D.; Singer, R. H.; Lionnet, T.; Lavis, L. D. A general method to improve fluorophores for live-cell and single-molecule microscopy. *Nat. Methods* **2015**, *12*, 244-250.

6. Hagen, V.; Dekowski, B.; Nache, V.; Schmidt, R.; Geissler, D.; Lorenz, D.; Eichhorst, J.; Keller, S.; Kaneko, H.; Benndorf, K.; Wiesner, B. Coumarinylmethyl esters for ultrafast release of high concentrations of cyclic nucleotides upon one- and two-photon photolysis. *Angew. Chem. Int. Ed.* **2005**, *44*, 7887-7891.

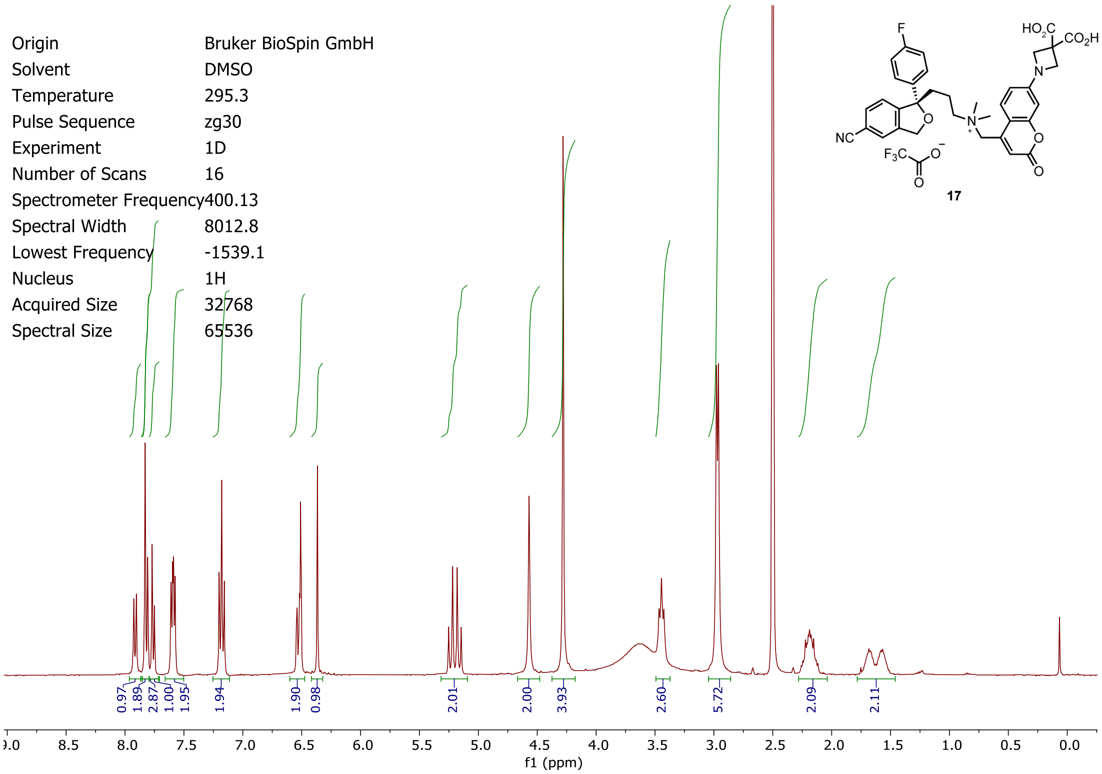
Origin Bruker BioSpin GmbH
Solvent DMSO
Temperature 295.3
Pulse Sequence zg30
Experiment 1D
Number of Scans 16
Spectrometer Frequency 400.13
Spectral Width 8012.8
Lowest Frequency -1539.1
Nucleus 1H
Acquired Size 32768
Spectral Size 65536
F
NC
HO2C
CO2H
N
O
O
O
F3C
17
0.97 1.89 2.87 1.00 1.95 1.94 1.90 0.98 2.01 2.00 3.93 2.60 5.72 2.09 2.11
8.5 8.0 7.5 7.0 6.5 6.0 5.5 5.0 4.5 4.0 3.5 3.0 2.5 2.0 1.5 1.0 0.5 0.0
f1 (ppm)

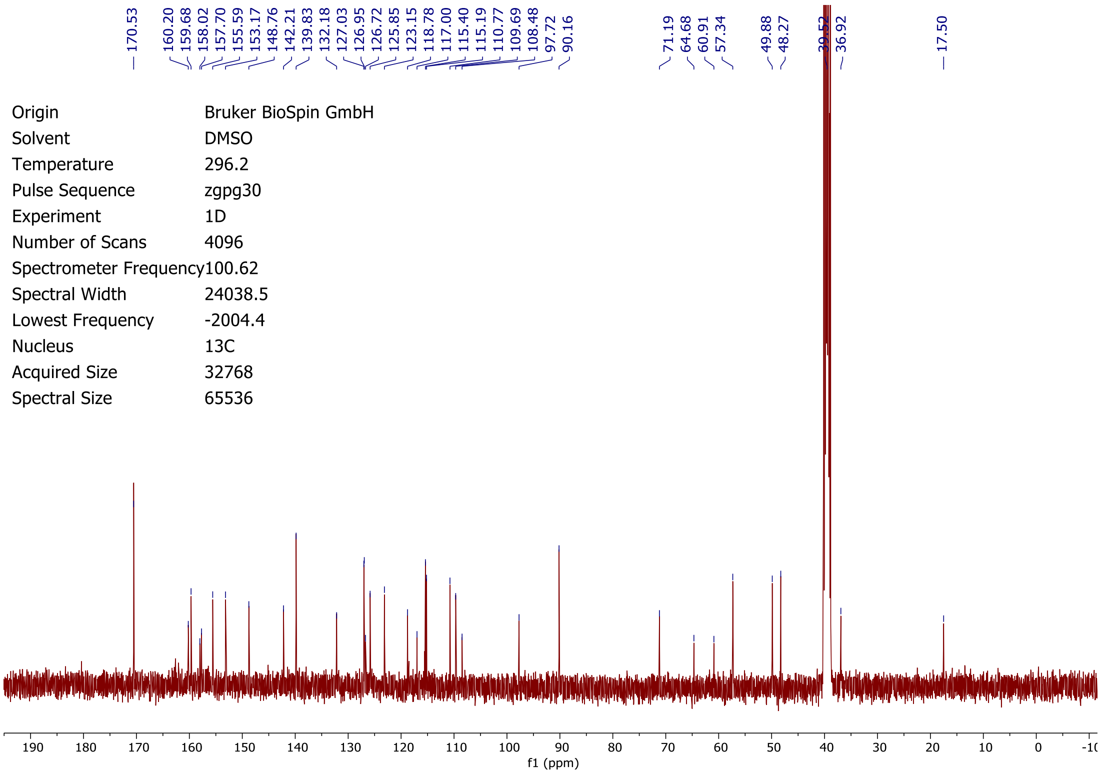
170.53 160.20 159.68 158.02 157.70 155.59 153.17 148.76 142.21 139.83 132.18 127.03 126.95 126.72 125.85 123.15 118.78 117.00 115.40 115.19 110.77 109.69 108.48 97.72 90.16 71.19 64.68 60.91 57.34 49.88 48.27 39.52 36.92 17.50
Origin Bruker BioSpin GmbH
Solvent DMSO
Temperature 296.2
Pulse Sequence zgpg30
Experiment 1D
Number of Scans 4096
Spectrometer Frequency 100.62
Spectral Width 24038.5
Lowest Frequency -2004.4
Nucleus 13C
Acquired Size 32768
Spectral Size 65536
190 180 170 160 150 140 130 120 110 100 90 80 70 60 50 40 30 20 10 0
f1 (ppm)

Origin	Bruker BioSpin GmbH
Solvent	DMSO
Temperature	295.5
Pulse Sequence	zg30
Experiment	1D
Number of Scans	16
Spectrometer Frequency	400.13
Spectral Width	8012.8
Lowest Frequency	-1539.2
Nucleus	1H
Acquired Size	32768
Spectral Size	65536

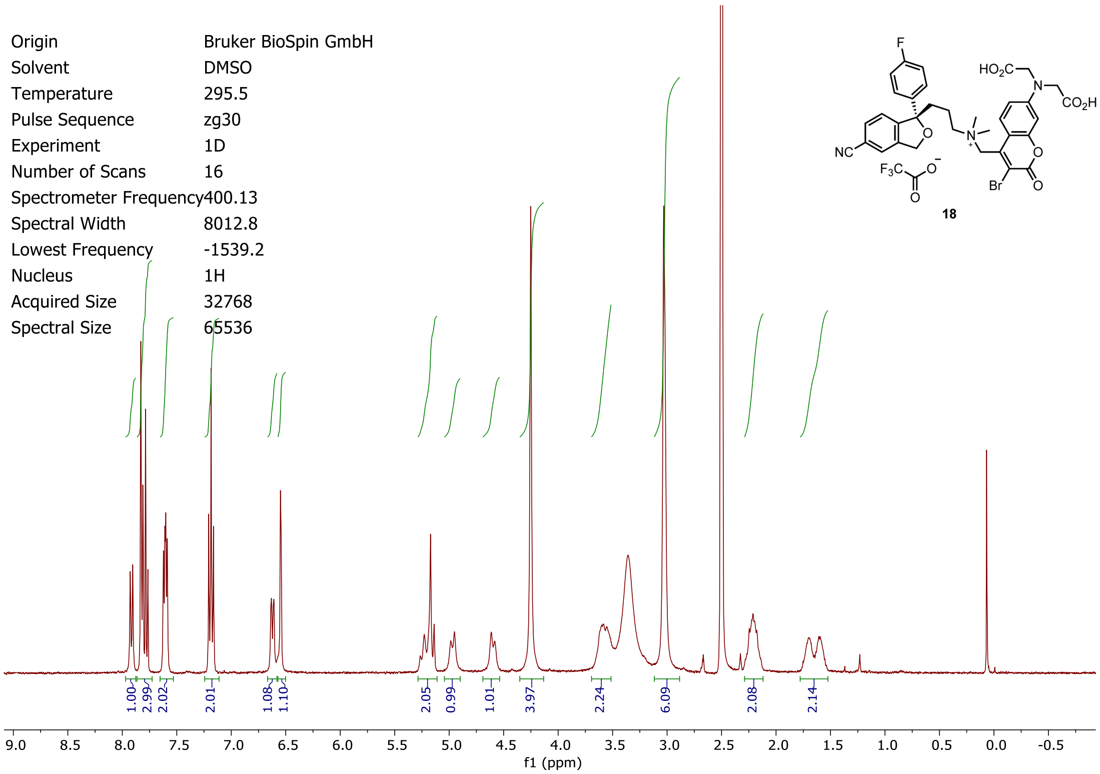

Origin	Bruker BioSpin GmbH
Solvent	DMSO
Temperature	300.0
Pulse Sequence	zgpg30
Experiment	1D
Number of Scans	6144
Spectrometer Frequency	100.62
Spectral Width	24038.5
Lowest Frequency	-2007.3
Nucleus	13C
Acquired Size	32768
Spectral Size	65536

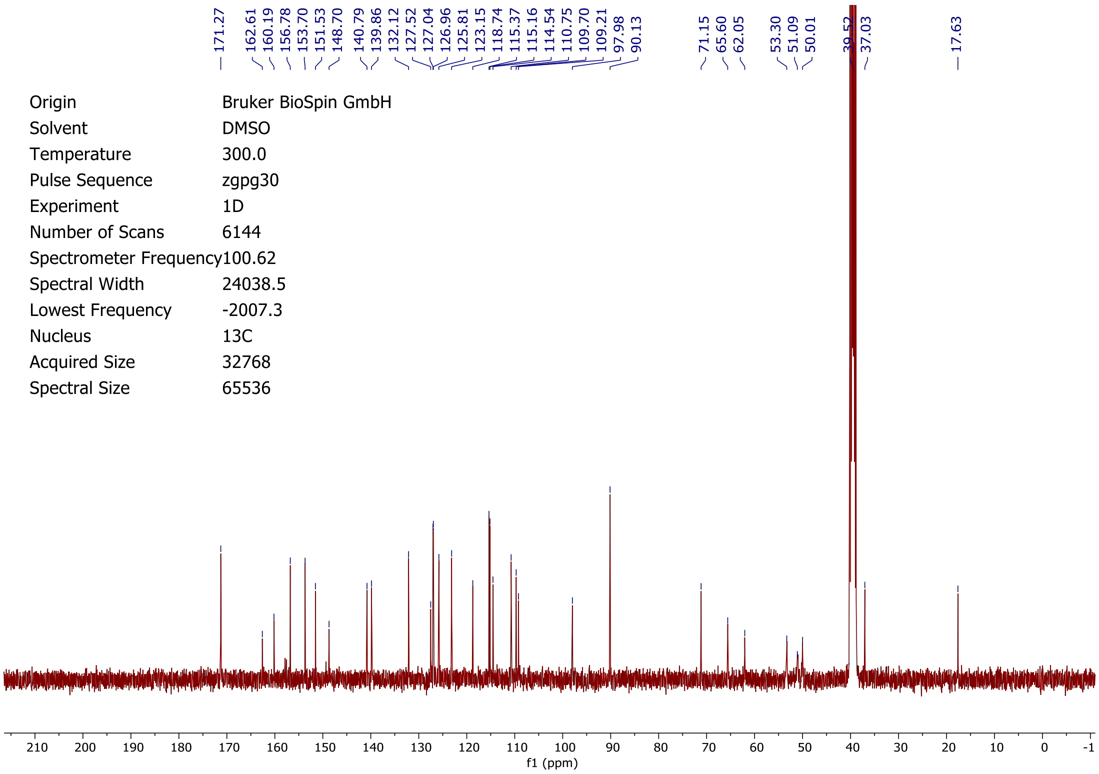

Origin	Bruker BioSpin GmbH
Solvent	DMSO
Temperature	295.3
Pulse Sequence	zg30
Experiment	1D
Number of Scans	16
Spectrometer Frequency	400.13
Spectral Width	8012.8
Lowest Frequency	-1539.1
Nucleus	1H
Acquired Size	32768
Spectral Size	65536

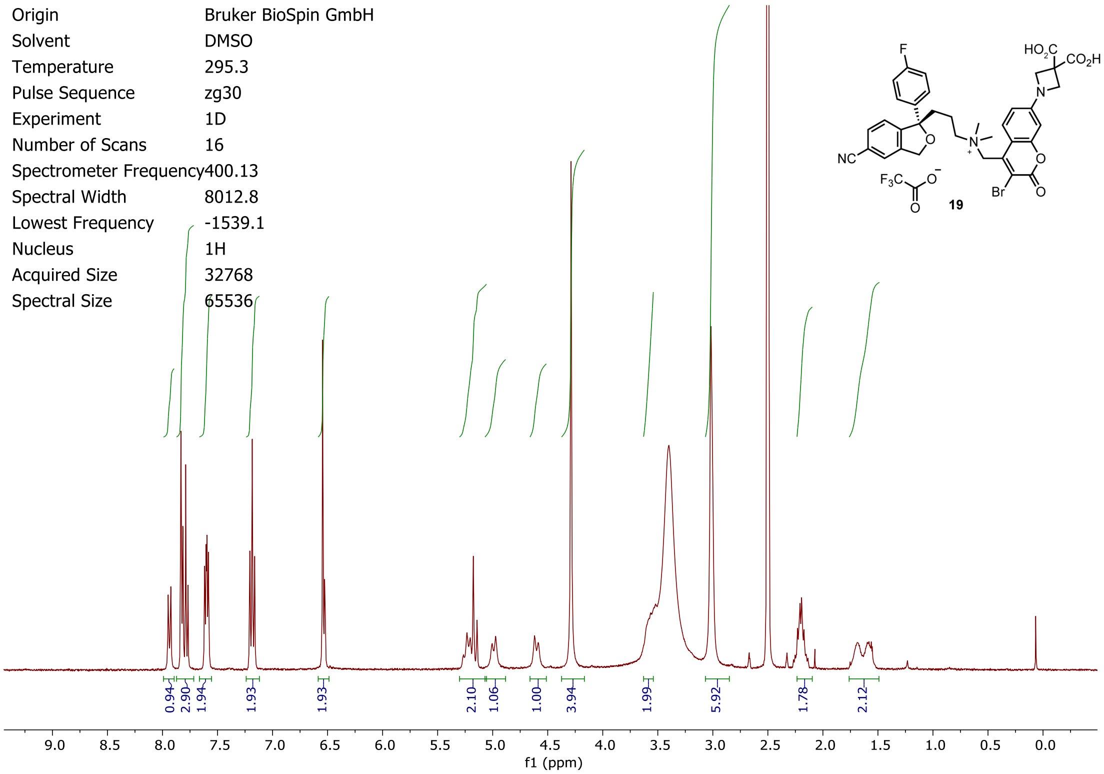

Origin	Bruker BioSpin GmbH
Solvent	DMSO
Temperature	296.1
Pulse Sequence	zgpg30
Experiment	1D
Number of Scans	10240
Spectrometer Frequency	100.62
Spectral Width	24038.5
Lowest Frequency	-2004.9
Nucleus	13C
Acquired Size	32768
Spectral Size	65536

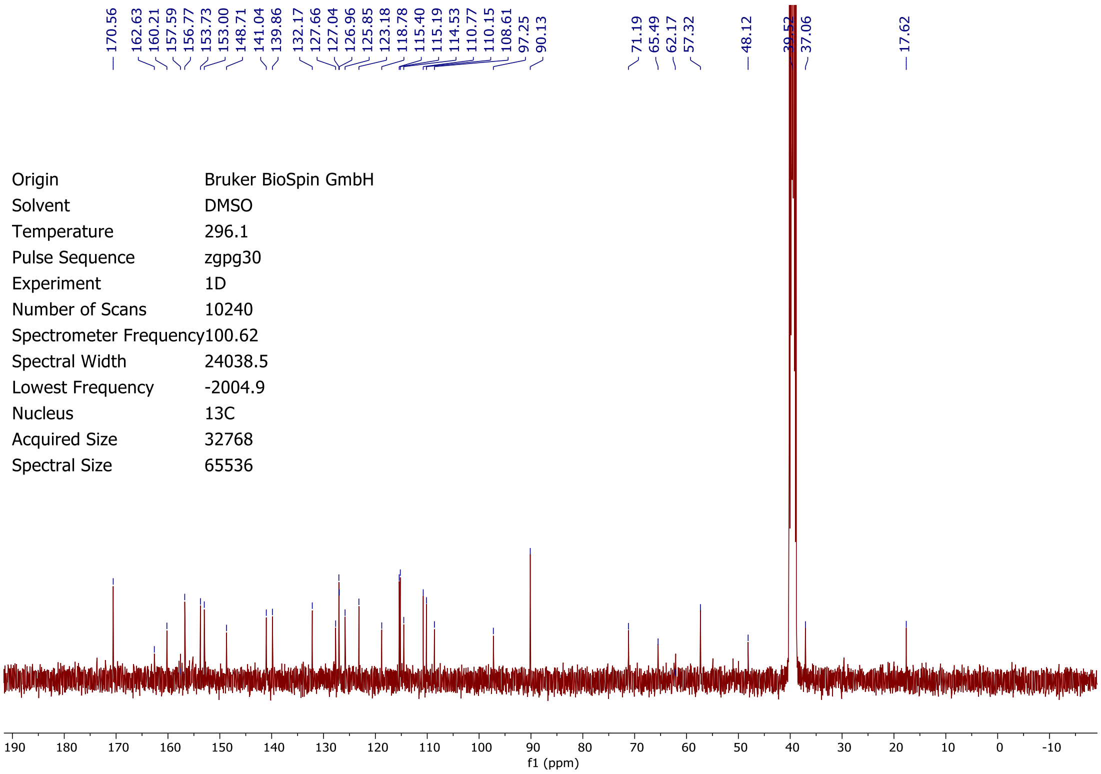

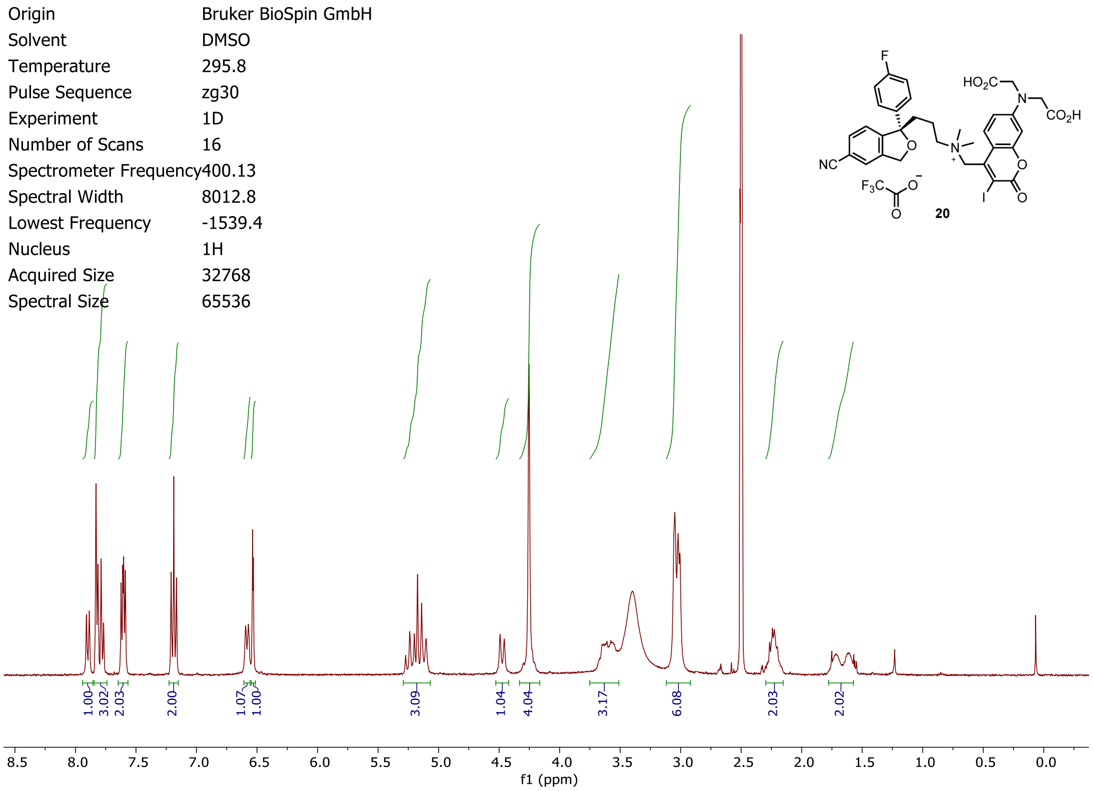
Origin Bruker BioSpin GmbH
Solvent DMSO
Temperature 295.8
Pulse Sequence zg30
Experiment 1D
Number of Scans 16
Spectrometer Frequency 400.13
Spectral Width 8012.8
Lowest Frequency -1539.4
Nucleus 1H
Acquired Size 32768
Spectral Size 65536
F
NC
O
HO_2C
N
CO_2H
O
O
I
F_3C
O^-
O
20
1.00
3.02
2.03
2.00
1.07
1.00
3.09
1.04
4.04
3.17
6.08
2.03
2.02
8.5 8.0 7.5 7.0 6.5 6.0 5.5 5.0 4.5 4.0 3.5 3.0 2.5 2.0 1.5 1.0 0.5 0.0
f1 (ppm)

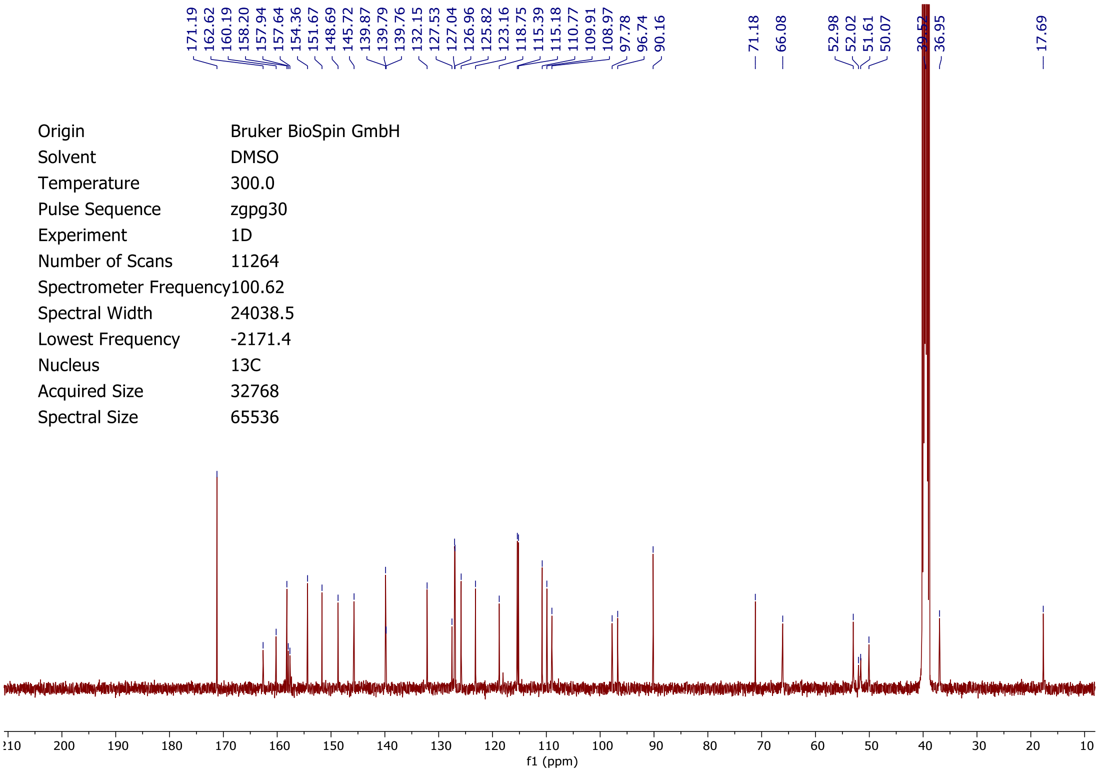
171.19
162.62
160.19
158.20
157.94
157.64
154.36
151.67
148.69
145.72
139.87
139.79
139.76
132.15
127.53
127.04
126.96
125.82
123.16
118.75
115.39
115.18
110.77
109.91
108.97
97.78
96.74
90.16
71.18
66.08
52.98
52.02
51.61
50.07
39.52
36.95
17.69
Origin Bruker BioSpin GmbH
Solvent DMSO
Temperature 300.0
Pulse Sequence zgpg30
Experiment 1D
Number of Scans 11264
Spectrometer Frequency 100.62
Spectral Width 24038.5
Lowest Frequency -2171.4
Nucleus 13C
Acquired Size 32768
Spectral Size 65536
210 200 190 180 170 160 150 140 130 120 110 100 90 80 70 60 50 40 30 20 10
f1 (ppm)

Origin	Bruker BioSpin GmbH
Solvent	DMSO
Temperature	300.0
Pulse Sequence	zg30
Experiment	1D
Number of Scans	16
Spectrometer Frequency	400.13
Spectral Width	8012.8
Lowest Frequency	-1539.3
Nucleus	1H
Acquired Size	32768
Spectral Size	65536

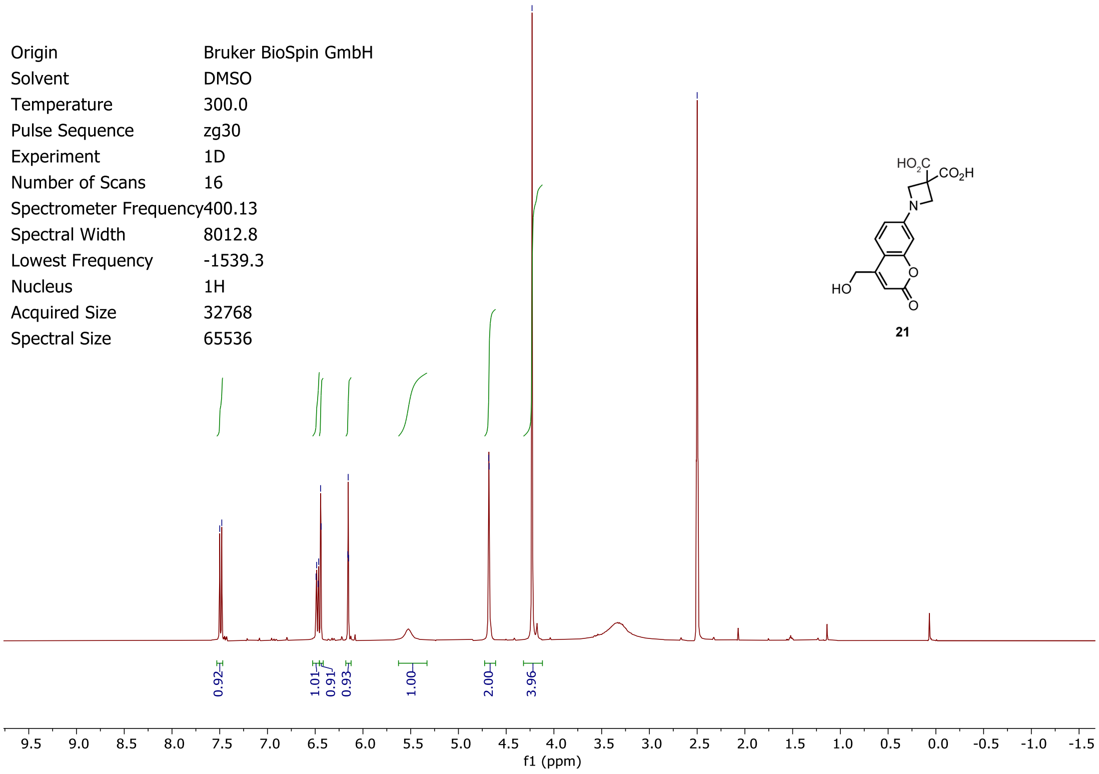

21

Origin	Bruker BioSpin GmbH
Solvent	DMSO
Temperature	300.0
Pulse Sequence	zgpg30
Experiment	1D
Number of Scans	10240
Spectrometer Frequency	100.62
Spectral Width	24038.5
Lowest Frequency	-2008.2
Nucleus	13C
Acquired Size	32768
Spectral Size	65536

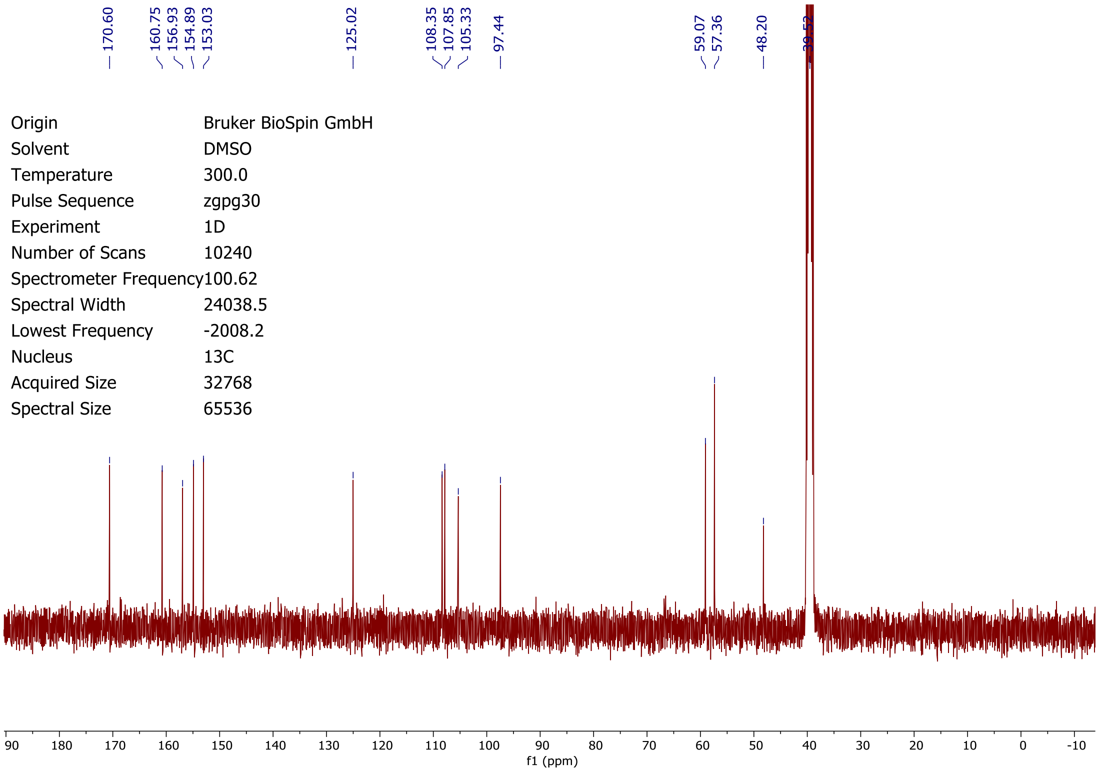

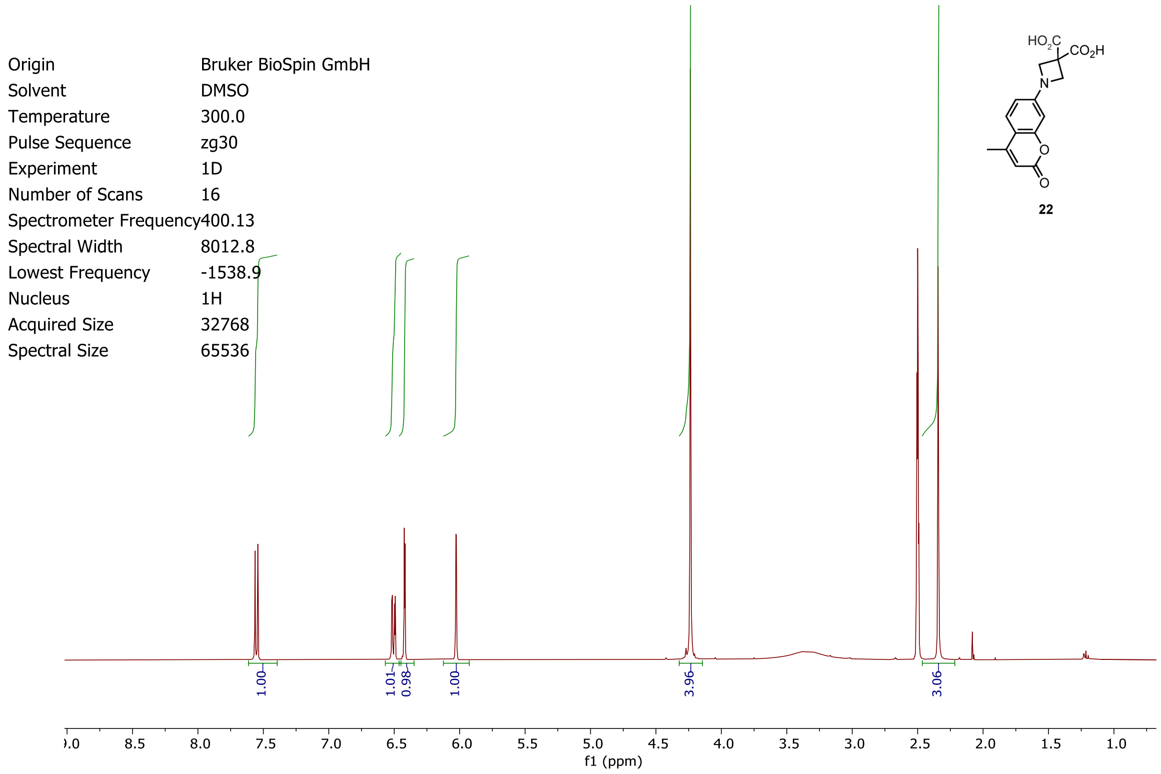
Origin Bruker BioSpin GmbH
Solvent DMSO
Temperature 300.0
Pulse Sequence zg30
Experiment 1D
Number of Scans 16
Spectrometer Frequency 400.13
Spectral Width 8012.8
Lowest Frequency -1538.9
Nucleus 1H
Acquired Size 32768
Spectral Size 65536
1.00
1.01
0.98
1.00
3.96
3.06
f1 (ppm)
22

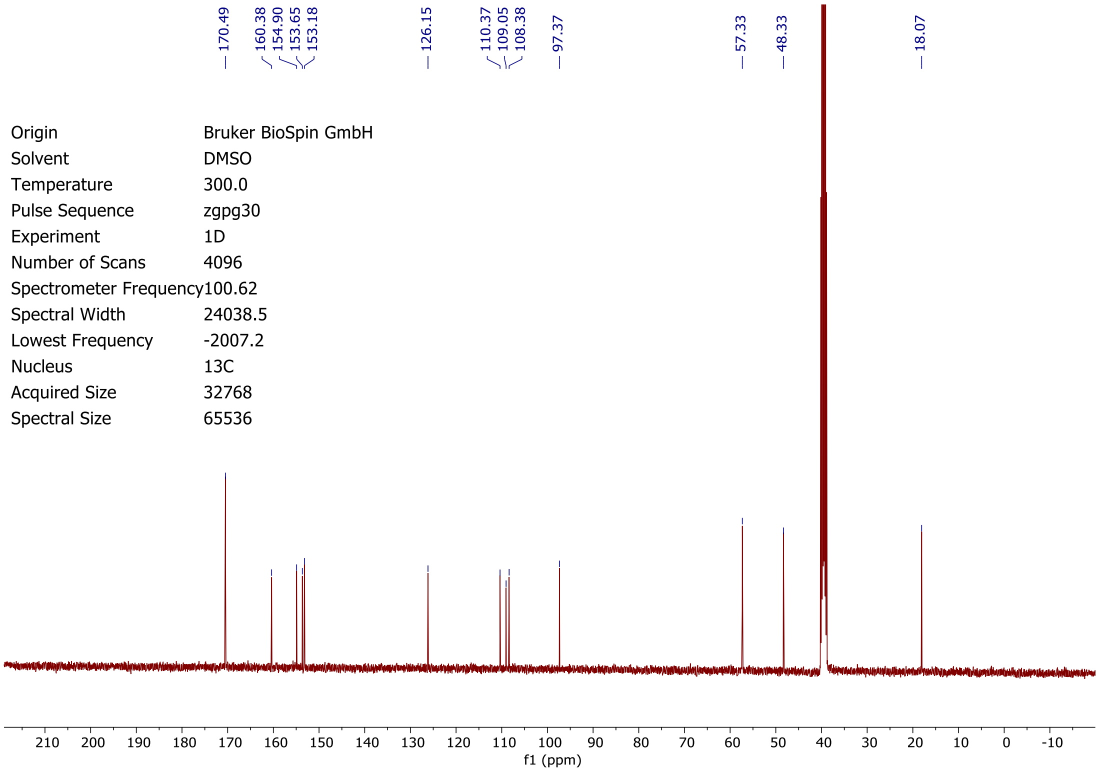
170.49
160.38
154.90
153.65
153.18
126.15
110.37
109.05
108.38
97.37
57.33
48.33
18.07
Origin Bruker BioSpin GmbH
Solvent DMSO
Temperature 300.0
Pulse Sequence zgpg30
Experiment 1D
Number of Scans 4096
Spectrometer Frequency 100.62
Spectral Width 24038.5
Lowest Frequency -2007.2
Nucleus 13C
Acquired Size 32768
Spectral Size 65536
f1 (ppm)

Origin	Bruker BioSpin GmbH
Solvent	DMSO
Temperature	295.3
Pulse Sequence	zg30
Experiment	1D
Number of Scans	16
Spectrometer Frequency	400.13
Spectral Width	8012.8
Lowest Frequency	-1540.5
Nucleus	1H
Acquired Size	32768
Spectral Size	65536

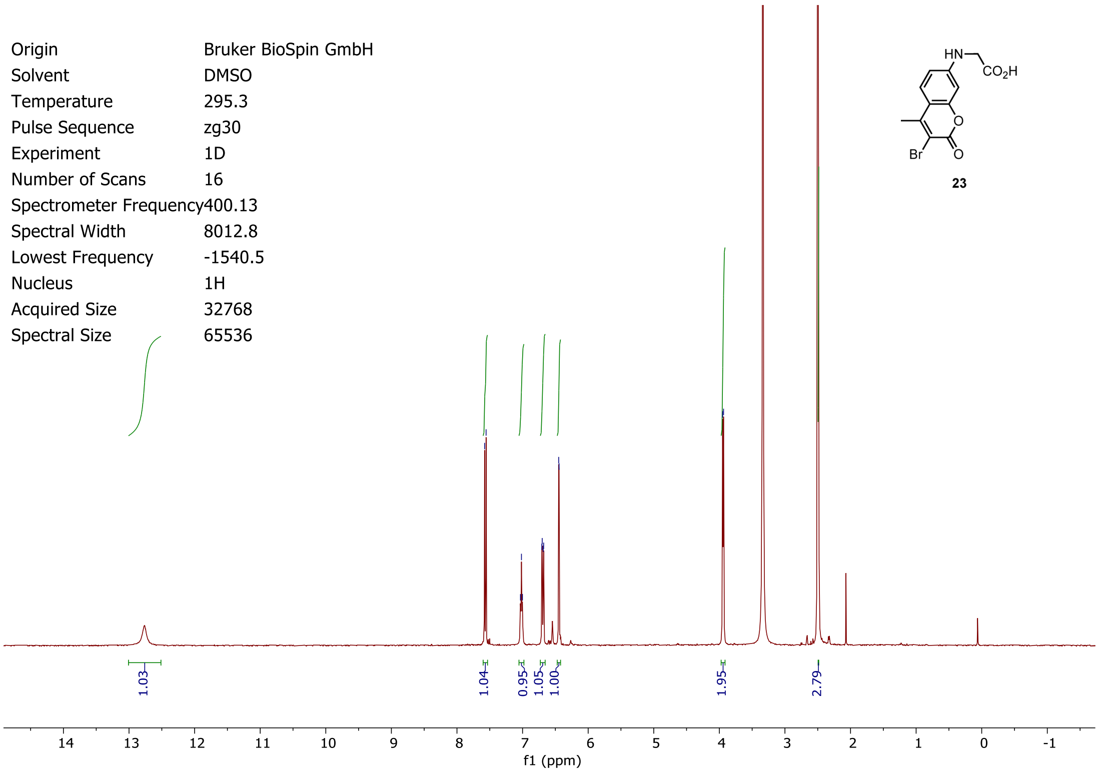

23

Origin	Bruker BioSpin GmbH
Solvent	DMSO
Temperature	296.2
Pulse Sequence	zgpg30
Experiment	1D
Number of Scans	6144
Spectrometer Frequency	100.62
Spectral Width	24038.5
Lowest Frequency	-2004.6
Nucleus	13C
Acquired Size	32768
Spectral Size	65536

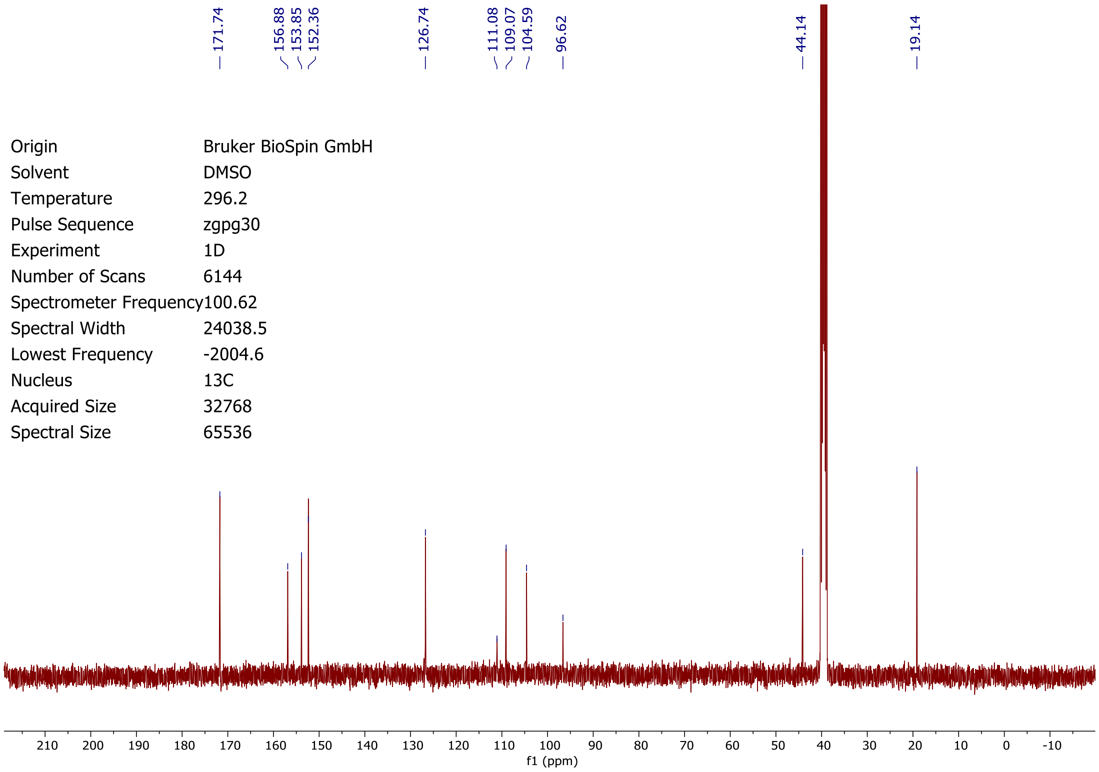

Origin	Bruker BioSpin GmbH
Solvent	DMSO
Temperature	295.4
Pulse Sequence	zg30
Experiment	1D
Number of Scans	16
Spectrometer Frequency	400.13
Spectral Width	8012.8
Lowest Frequency	-1539.3
Nucleus	1H
Acquired Size	32768
Spectral Size	65536

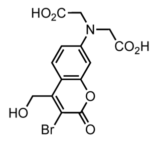

24

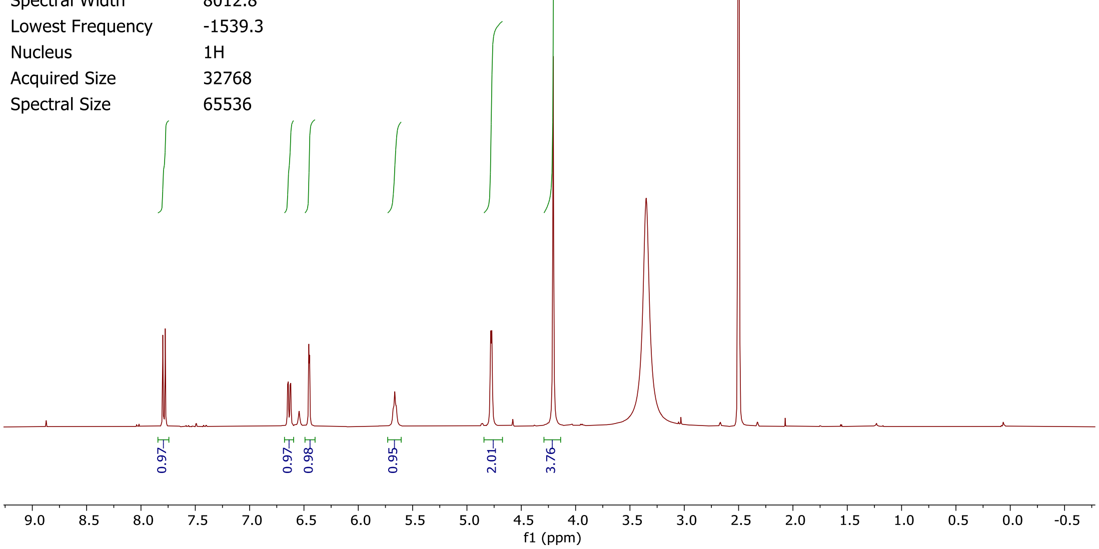

Origin	Bruker BioSpin GmbH
Solvent	DMSO
Temperature	296.1
Pulse Sequence	zgpg30
Experiment	1D
Number of Scans	5120
Spectrometer Frequency	100.62
Spectral Width	24038.5
Lowest Frequency	-2003.5
Nucleus	13C
Acquired Size	32768
Spectral Size	65536

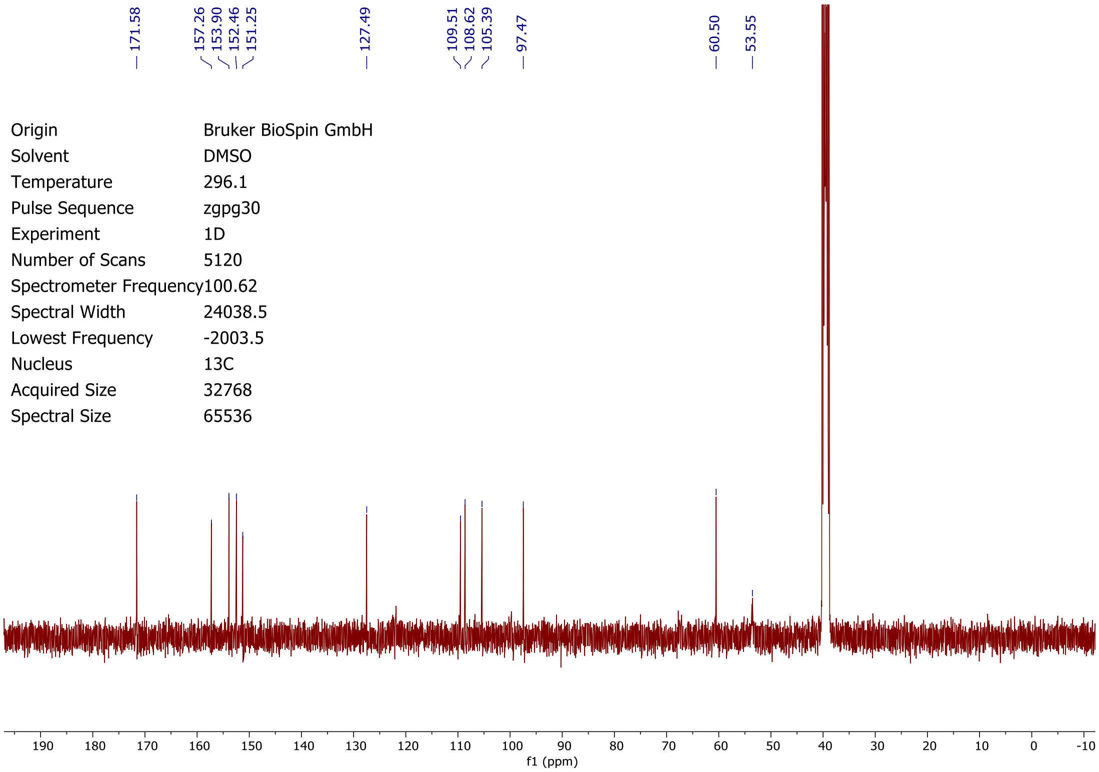

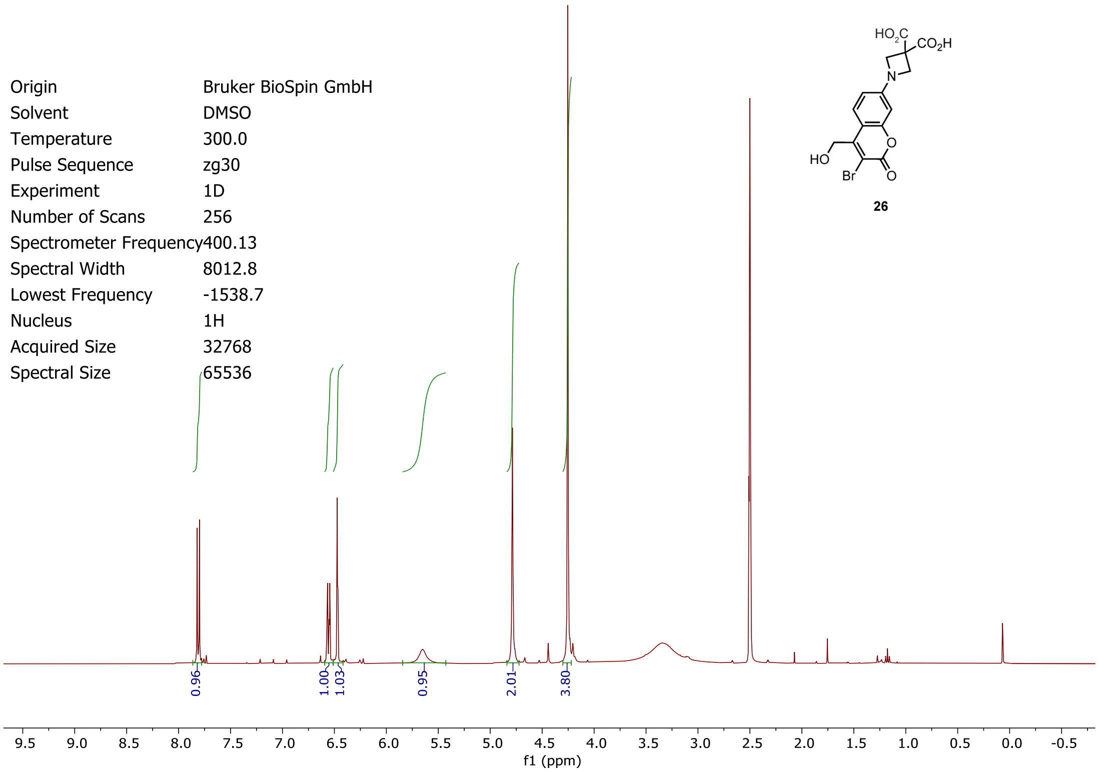
HO2C
CO2H
N
HO
Br
O
O
26
Origin Bruker BioSpin GmbH
Solvent DMSO
Temperature 300.0
Pulse Sequence zg30
Experiment 1D
Number of Scans 256
Spectrometer Frequency 400.13
Spectral Width 8012.8
Lowest Frequency -1538.7
Nucleus 1H
Acquired Size 32768
Spectral Size 65536
0.96
1.00
1.03
0.95
2.01
3.80
9.5 9.0 8.5 8.0 7.5 7.0 6.5 6.0 5.5 5.0 4.5 4.0 3.5 3.0 2.5 2.0 1.5 1.0 0.5 0.0 -0.5
f1 (ppm)

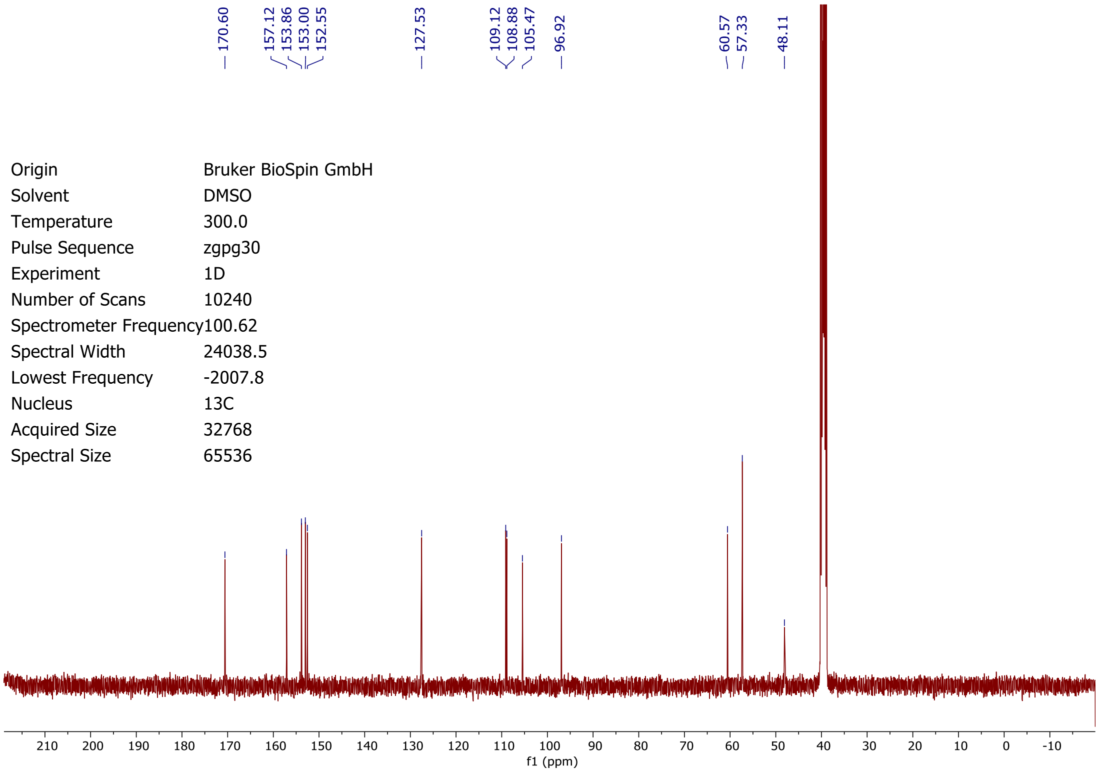
170.60
157.12
153.86
153.00
152.55
127.53
109.12
108.88
105.47
96.92
60.57
57.33
48.11
Origin Bruker BioSpin GmbH
Solvent DMSO
Temperature 300.0
Pulse Sequence zgpg30
Experiment 1D
Number of Scans 10240
Spectrometer Frequency 100.62
Spectral Width 24038.5
Lowest Frequency -2007.8
Nucleus 13C
Acquired Size 32768
Spectral Size 65536
210 200 190 180 170 160 150 140 130 120 110 100 90 80 70 60 50 40 30 20 10 0 -10
f1 (ppm)

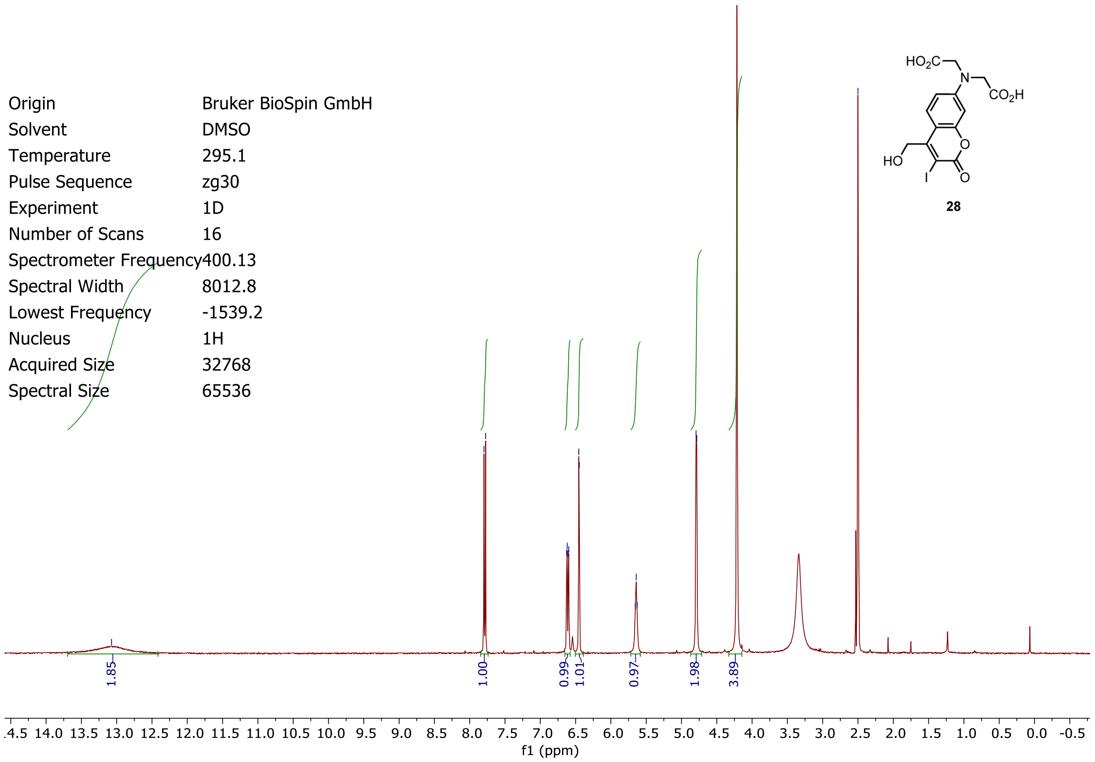
HO2C
N
CO2H
HO
O
I
O
28
Origin Bruker BioSpin GmbH
Solvent DMSO
Temperature 295.1
Pulse Sequence zg30
Experiment 1D
Number of Scans 16
Spectrometer Frequency 400.13
Spectral Width 8012.8
Lowest Frequency -1539.2
Nucleus 1H
Acquired Size 32768
Spectral Size 65536
1.85
1.00
0.99
1.01
0.97
1.98
3.89
f1 (ppm)

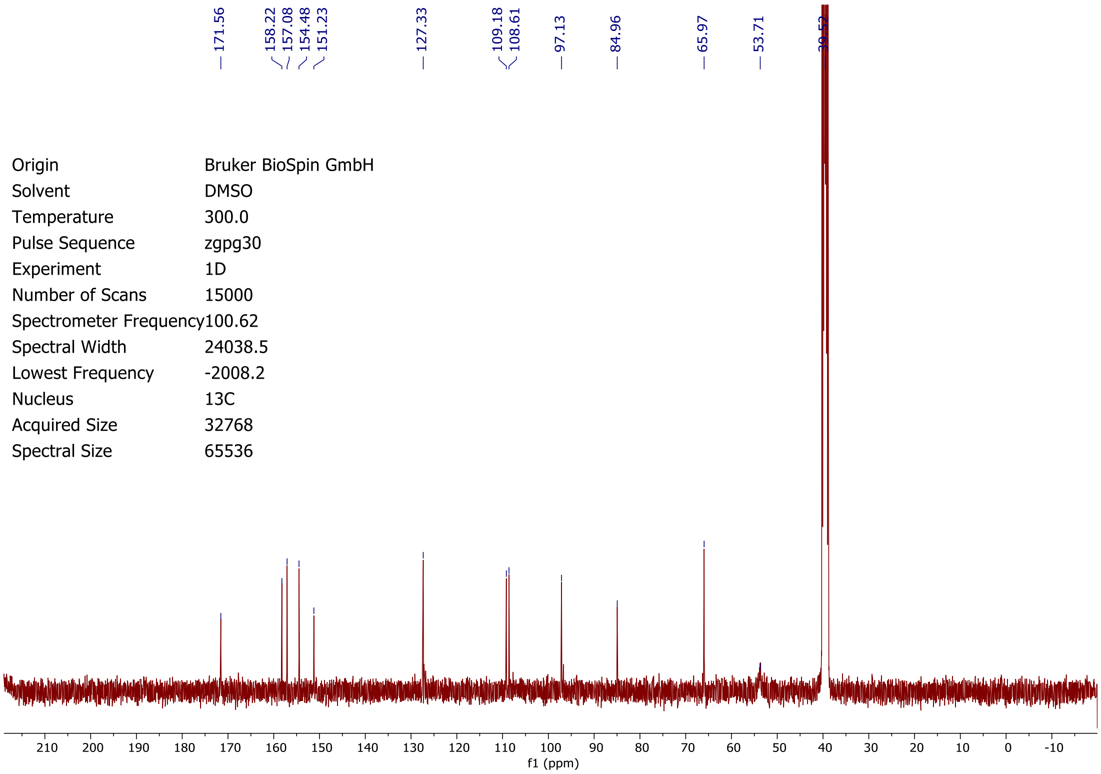
171.56
158.22
157.08
154.48
151.23
127.33
109.18
108.61
97.13
84.96
65.97
53.71
Origin Bruker BioSpin GmbH
Solvent DMSO
Temperature 300.0
Pulse Sequence zgpg30
Experiment 1D
Number of Scans 15000
Spectrometer Frequency 100.62
Spectral Width 24038.5
Lowest Frequency -2008.2
Nucleus 13C
Acquired Size 32768
Spectral Size 65536
f1 (ppm)

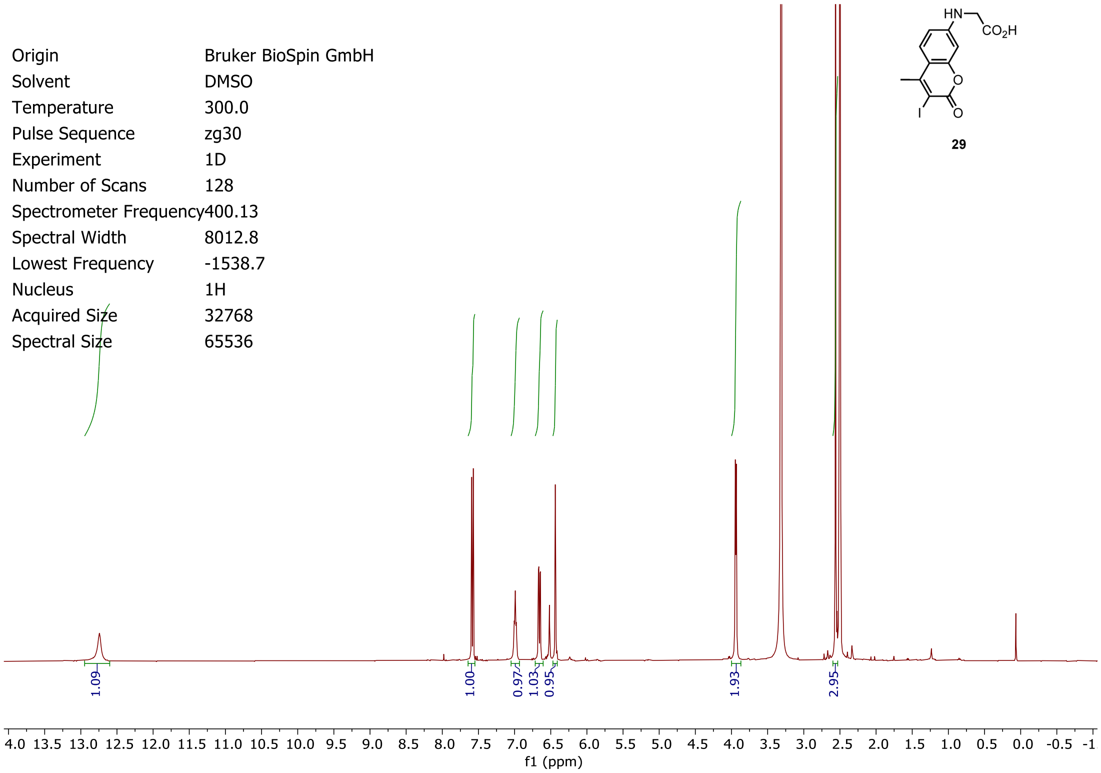

29
Origin Bruker BioSpin GmbH
Solvent DMSO
Temperature 300.0
Pulse Sequence zg30
Experiment 1D
Number of Scans 128
Spectrometer Frequency 400.13
Spectral Width 8012.8
Lowest Frequency -1538.7
Nucleus 1H
Acquired Size 32768
Spectral Size 65536
1.09
1.00
0.97
1.03
0.95
1.93
2.95
f1 (ppm)

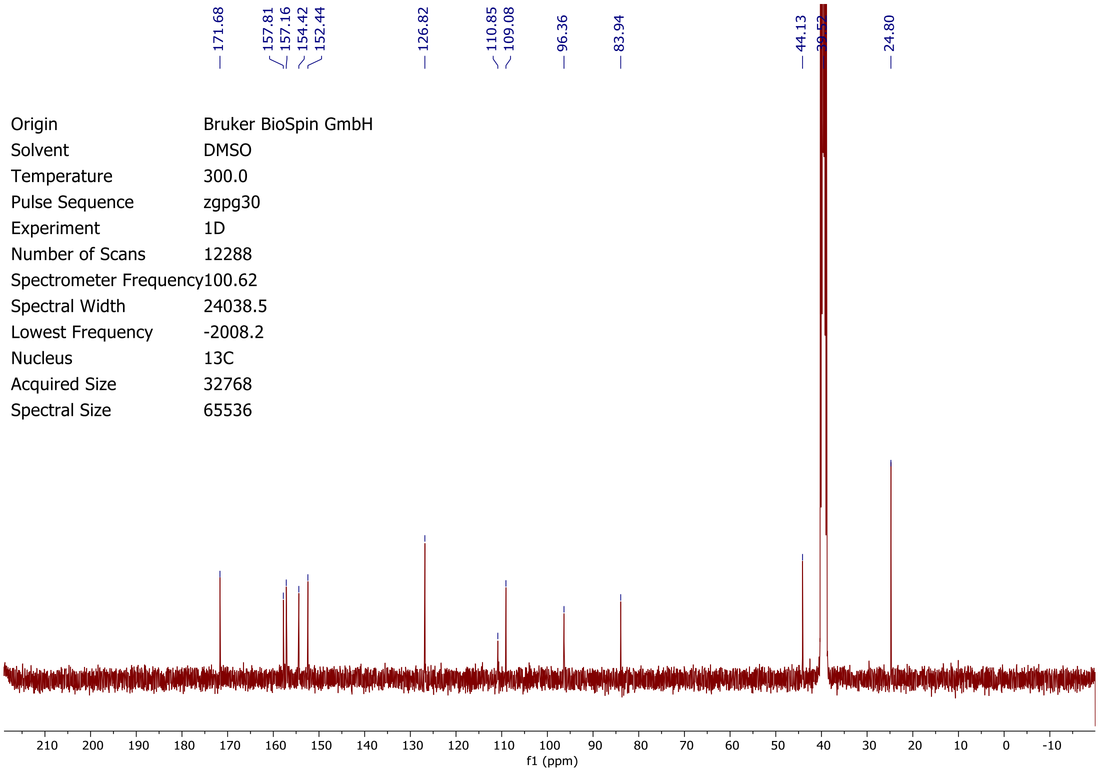

171.68
157.81
157.16
154.42
152.44
126.82
110.85
109.08
96.36
83.94
44.13
24.80
Origin Bruker BioSpin GmbH
Solvent DMSO
Temperature 300.0
Pulse Sequence zgpg30
Experiment 1D
Number of Scans 12288
Spectrometer Frequency 100.62
Spectral Width 24038.5
Lowest Frequency -2008.2
Nucleus 13C
Acquired Size 32768
Spectral Size 65536
f1 (ppm)

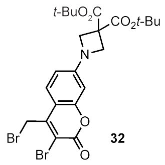

32

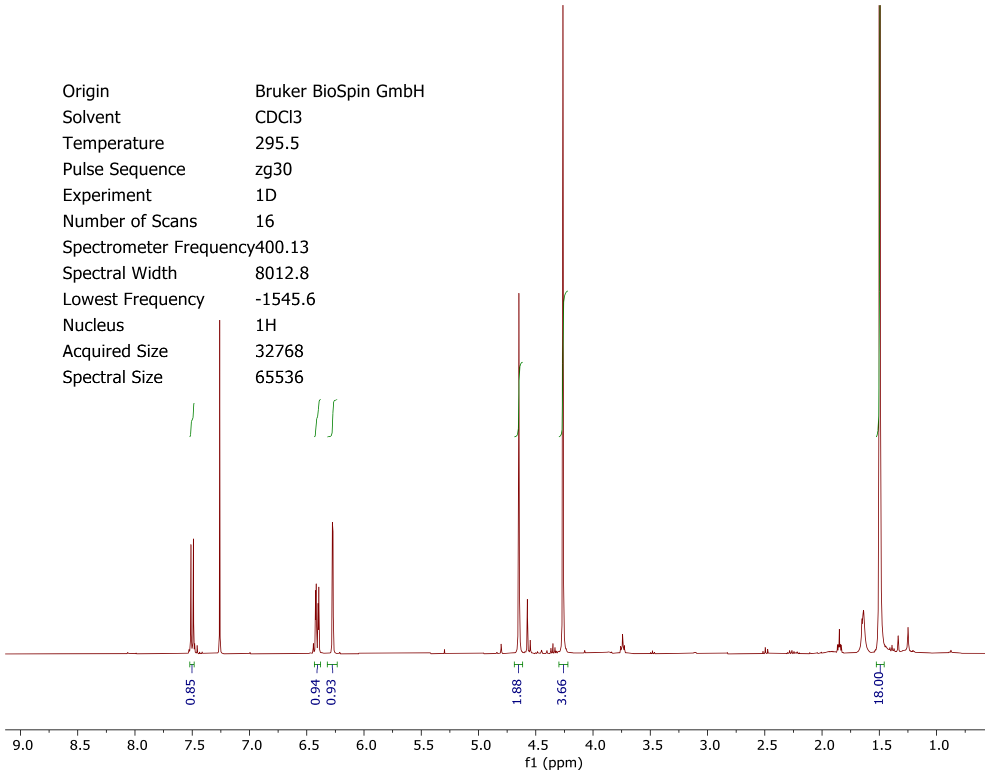

Origin	Bruker BioSpin GmbH
Solvent	CDCl3
Temperature	295.5
Pulse Sequence	zg30
Experiment	1D
Number of Scans	16
Spectrometer Frequency	400.13
Spectral Width	8012.8
Lowest Frequency	-1545.6
Nucleus	1H
Acquired Size	32768
Spectral Size	65536

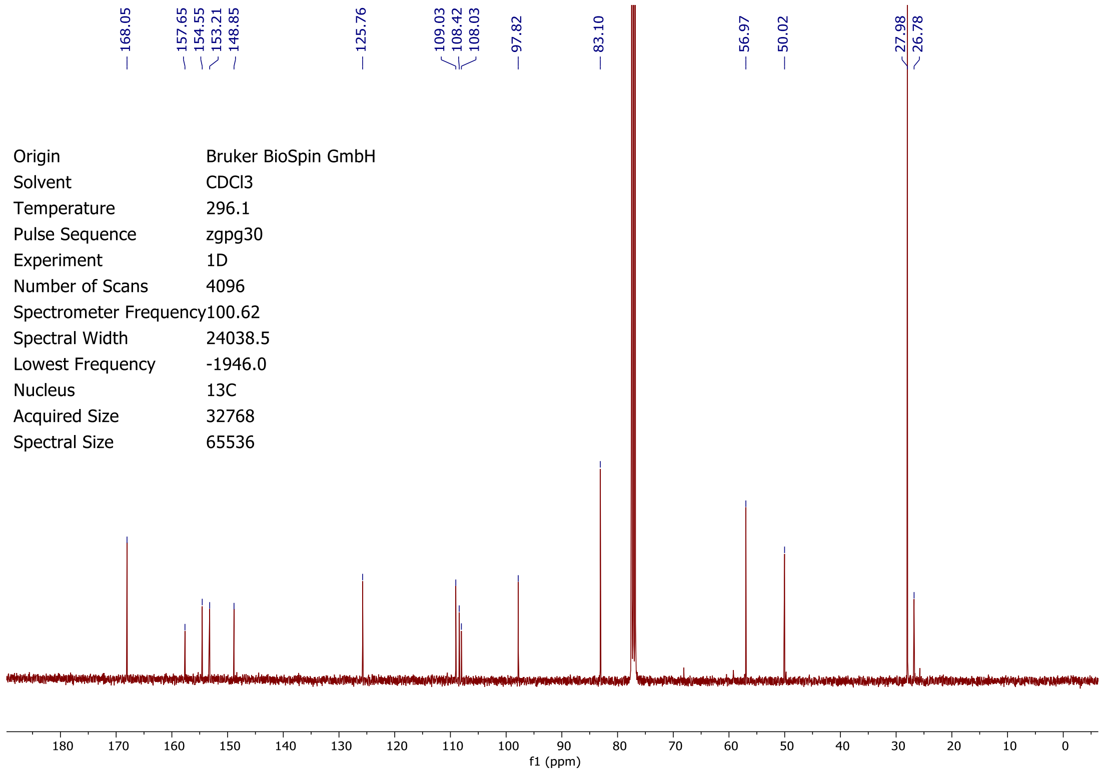

Origin	Bruker BioSpin GmbH
Solvent	CDCl3
Temperature	296.1
Pulse Sequence	zgpg30
Experiment	1D
Number of Scans	4096
Spectrometer Frequency	100.62
Spectral Width	24038.5
Lowest Frequency	-1946.0
Nucleus	13C
Acquired Size	32768
Spectral Size	65536

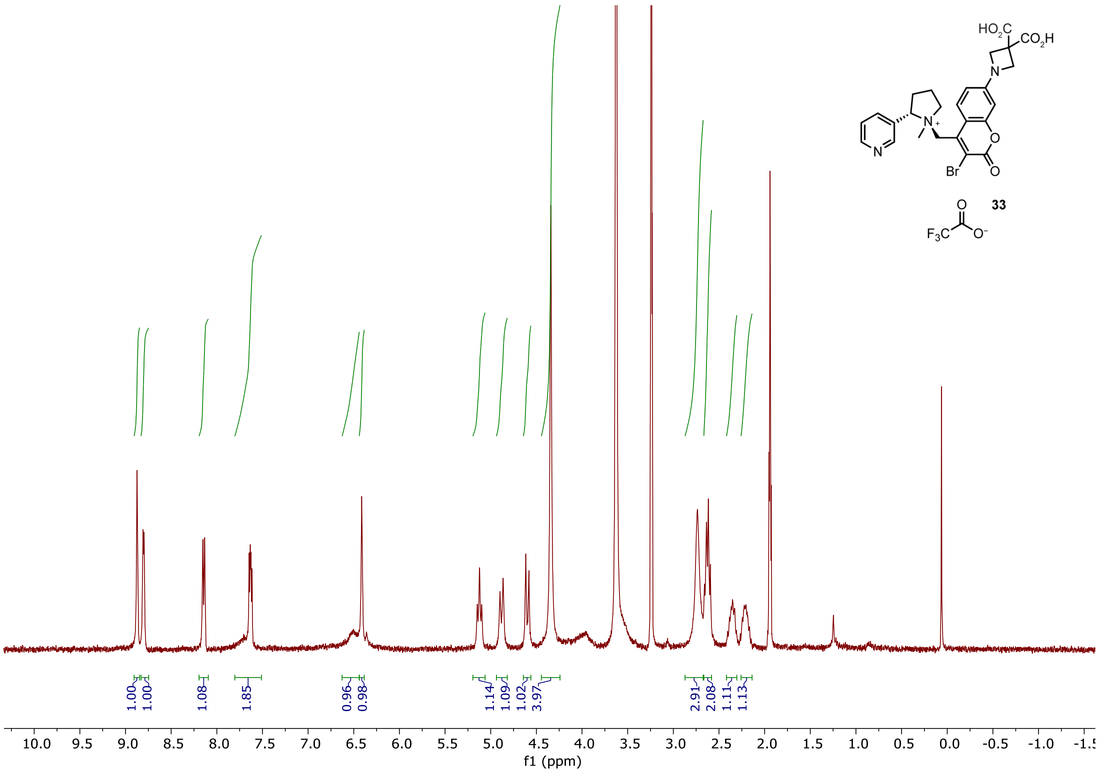

DAD1 A, Sig=254,4 Ref=off (2020_03\DEF_LC 2020-03-30 21-28-12\1DH-0501.D)

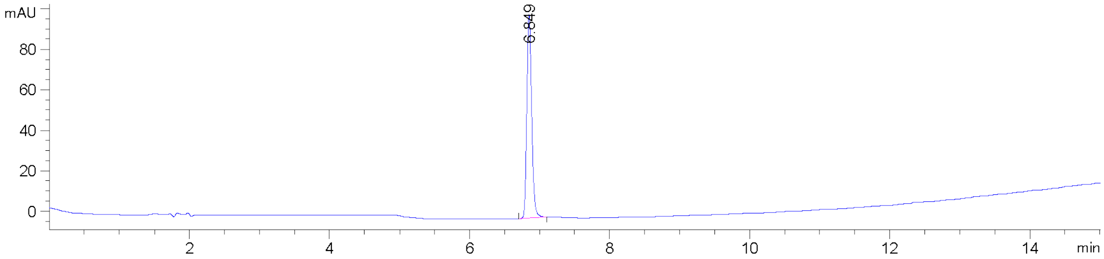

*MSD1 SPC, time=6.948:7.120 of C:\CHEM32\1\DATA\2020_03\DEF_LC 2020-03-30 21-28-12\1DH-0501.D ES-API, Pos, Scan, Fra

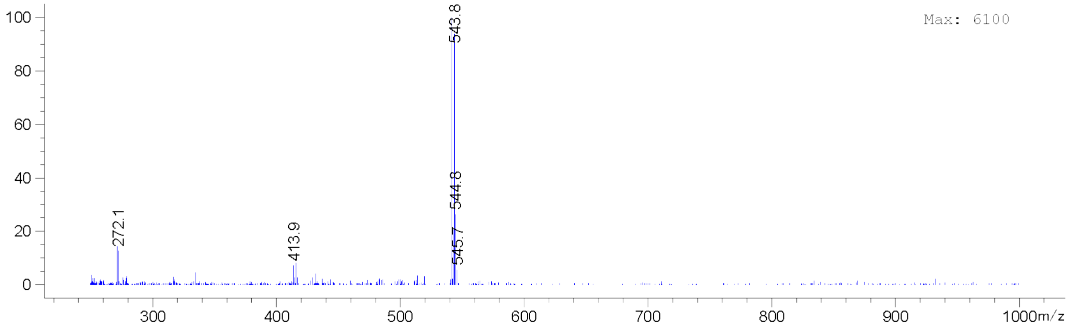

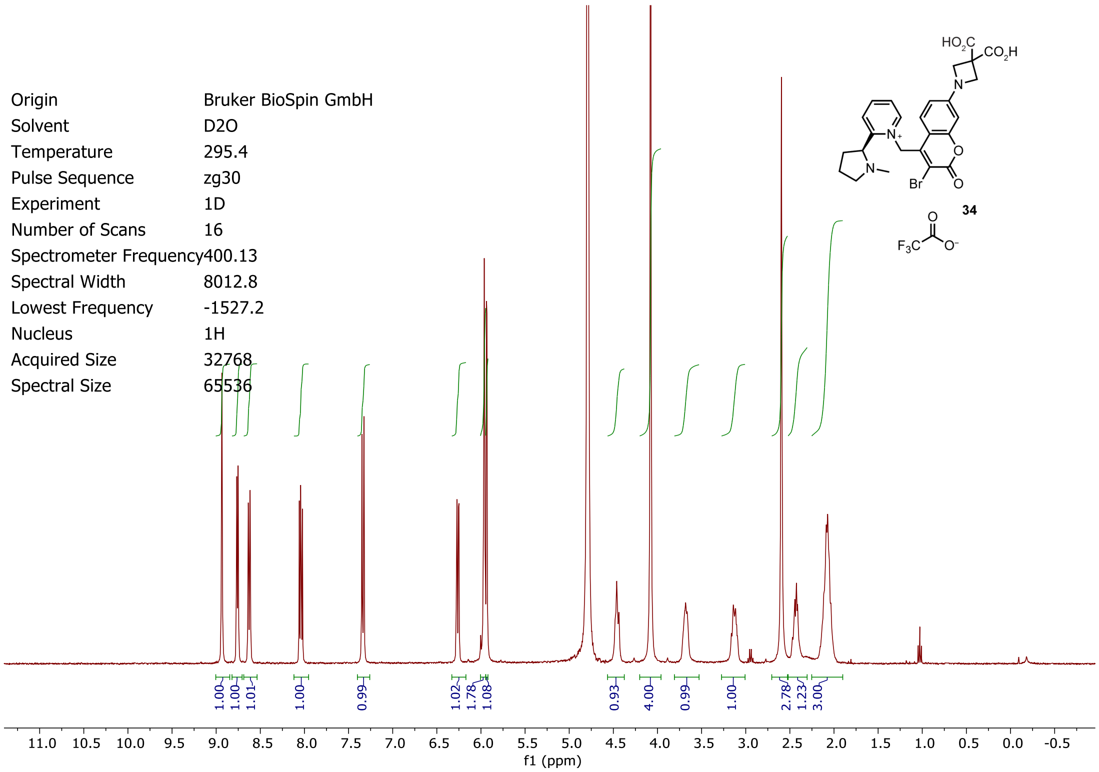

34

Origin	Bruker BioSpin GmbH
Solvent	D2O
Temperature	295.4
Pulse Sequence	zg30
Experiment	1D
Number of Scans	16
Spectrometer Frequency	400.13
Spectral Width	8012.8
Lowest Frequency	-1527.2
Nucleus	1H
Acquired Size	32768
Spectral Size	65536

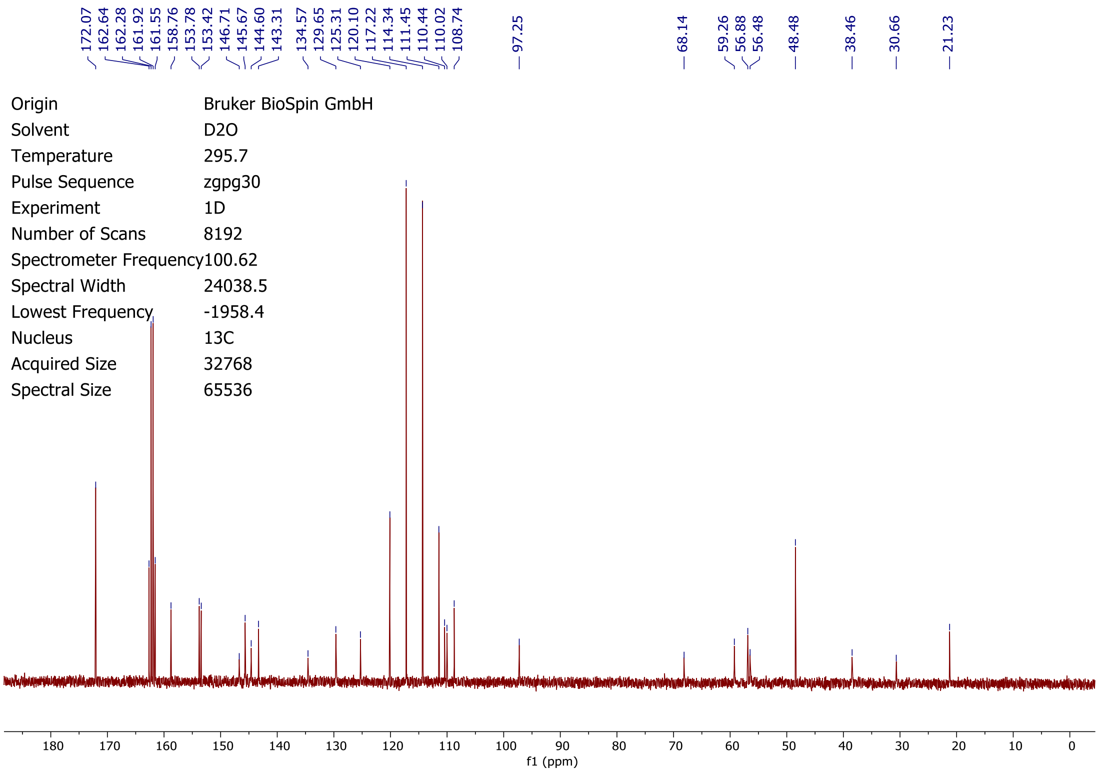

Origin	Bruker BioSpin GmbH
Solvent	D2O
Temperature	295.7
Pulse Sequence	zgpg30
Experiment	1D
Number of Scans	8192
Spectrometer Frequency	100.62
Spectral Width	24038.5
Lowest Frequency	-1958.4
Nucleus	13C
Acquired Size	32768
Spectral Size	65536

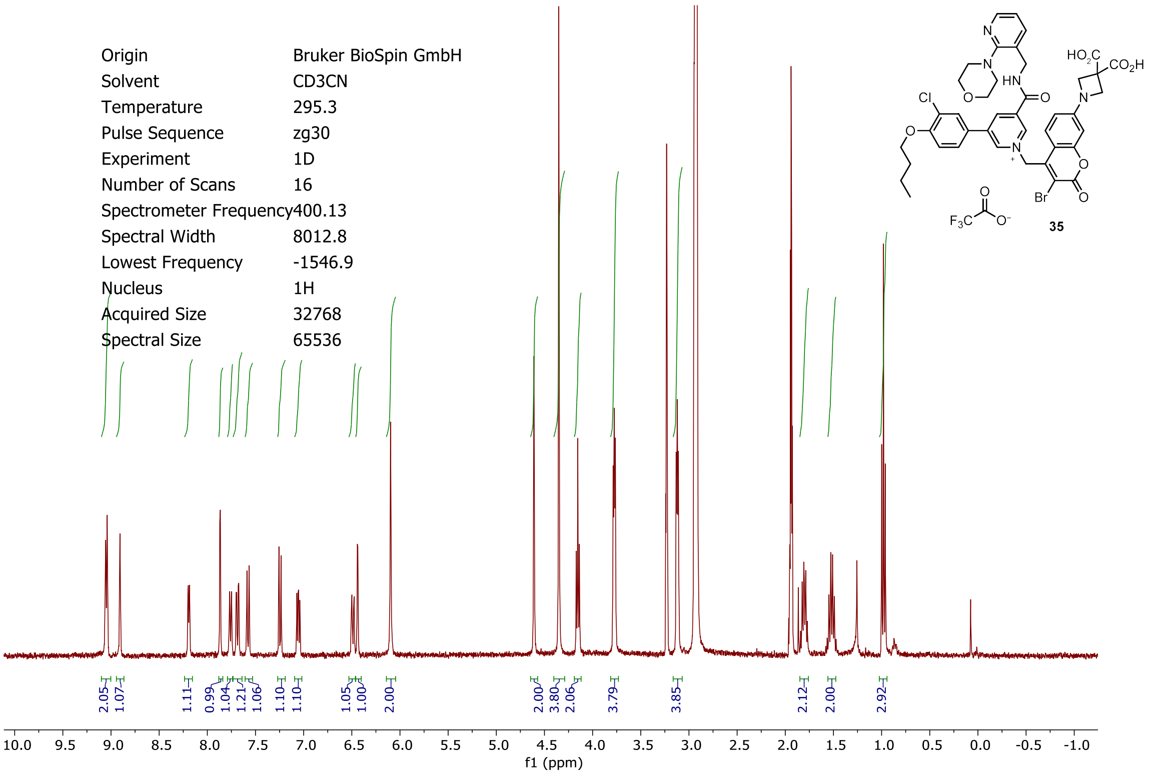

Origin Bruker BioSpin GmbH
Solvent CD3CN
Temperature 295.3
Pulse Sequence zg30
Experiment 1D
Number of Scans 16
Spectrometer Frequency 400.13
Spectral Width 8012.8
Lowest Frequency -1546.9
Nucleus 1H
Acquired Size 32768
Spectral Size 65536
2.05 1.07 1.11 0.99 1.04 1.21 1.06 1.10 1.10 1.05 1.00 2.00 2.00 3.80 2.06 3.79 3.85 2.12 2.00 2.92
10.0 9.5 9.0 8.5 8.0 7.5 7.0 6.5 6.0 5.5 5.0 4.5 4.0 3.5 3.0 2.5 2.0 1.5 1.0 0.5 0.0 -0.5 -1.0
f1 (ppm)
N
N
O
HN
O
Cl
O
N
HO2C
CO2H
N
O
O
Br
O
F3C
O⁻

35

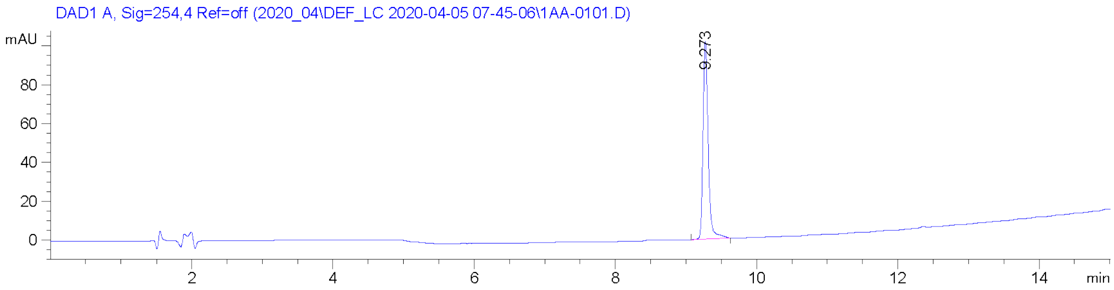

DAD1 A, Sig=254,4 Ref=off (2020_04\DEF_LC 2020-04-05 07-45-06\1AA-0101.D)
mAU
80
60
40
20
0
9.273
2
4
6
8
10
12
14
min

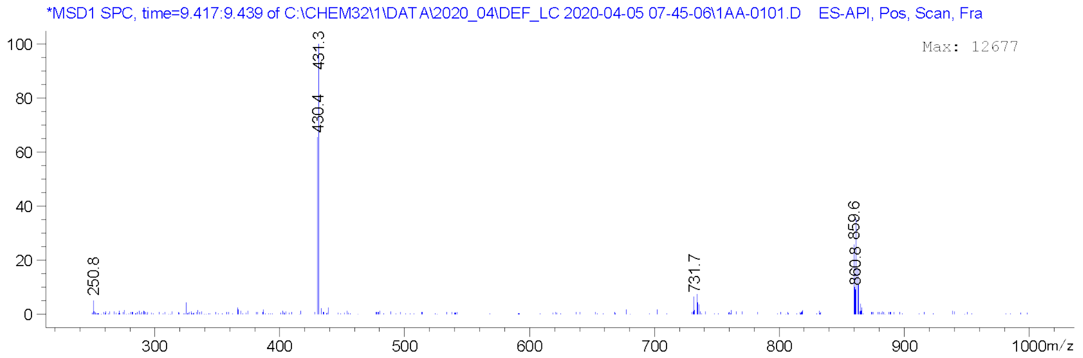

*MSD1 SPC, time=9.417:9.439 of C:\CHEM32\1\DATA\2020_04\DEF_LC 2020-04-05 07-45-06\1AA-0101.D ES-API, Pos, Scan, Fra
Max: 12677
100
80
60
40
20
0
250.8
431.3
430.4
731.7
860.8
859.6
300
400
500
600
700
800
900
1000
m/z

Origin	Bruker BioSpin GmbH
Solvent	DMSO
Temperature	303.0
Pulse Sequence	zg30
Experiment	1D
Number of Scans	128
Spectrometer Frequency	400.13
Spectral Width	8012.8
Lowest Frequency	-1566.3
Nucleus	1H
Acquired Size	32768
Spectral Size	65536

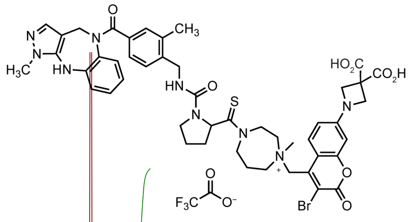

37

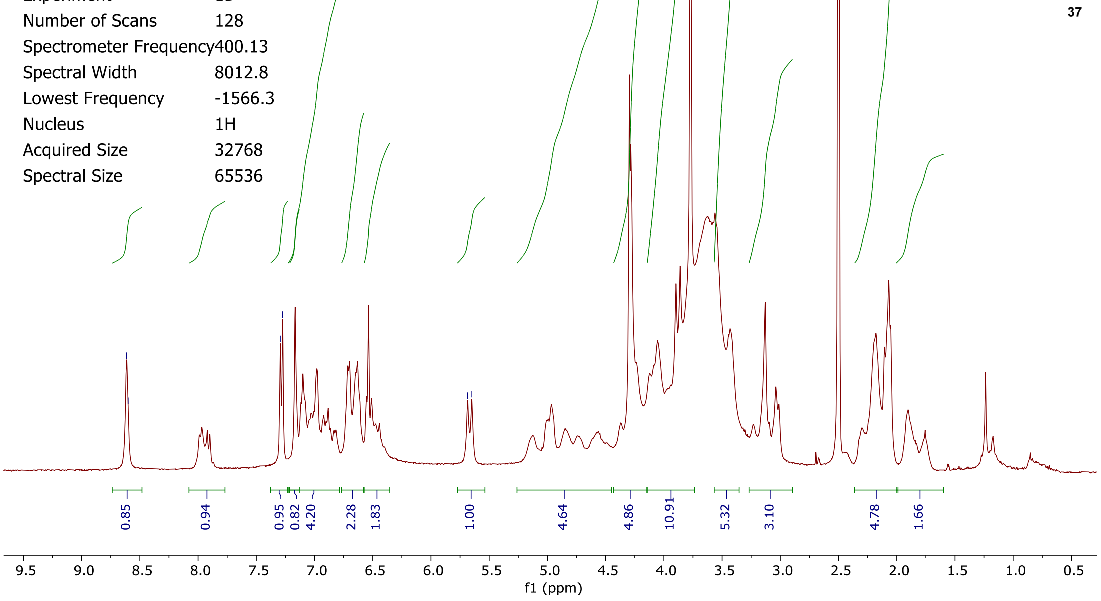

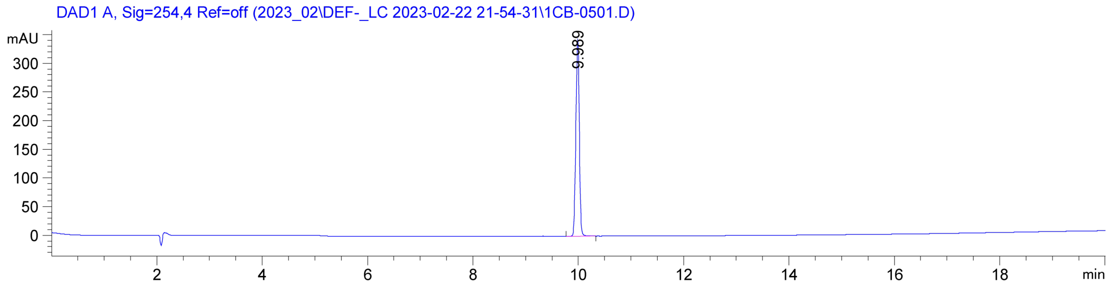

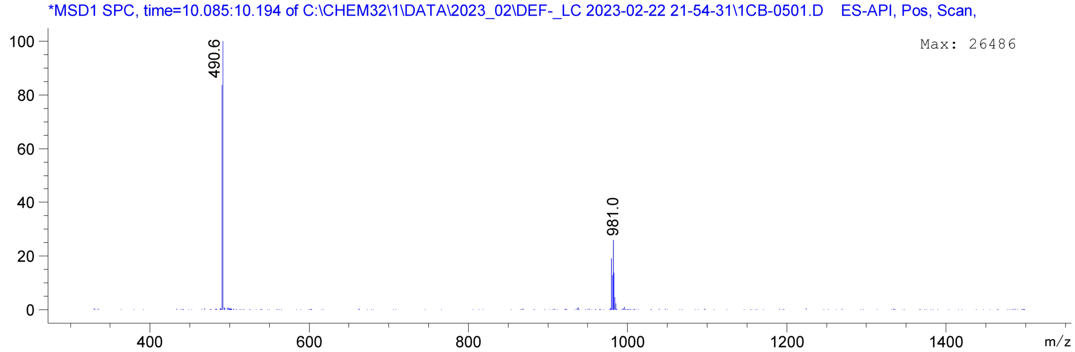

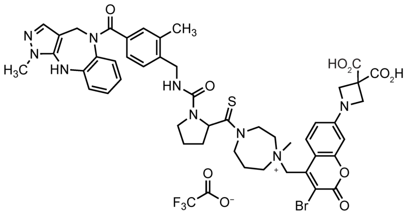

37

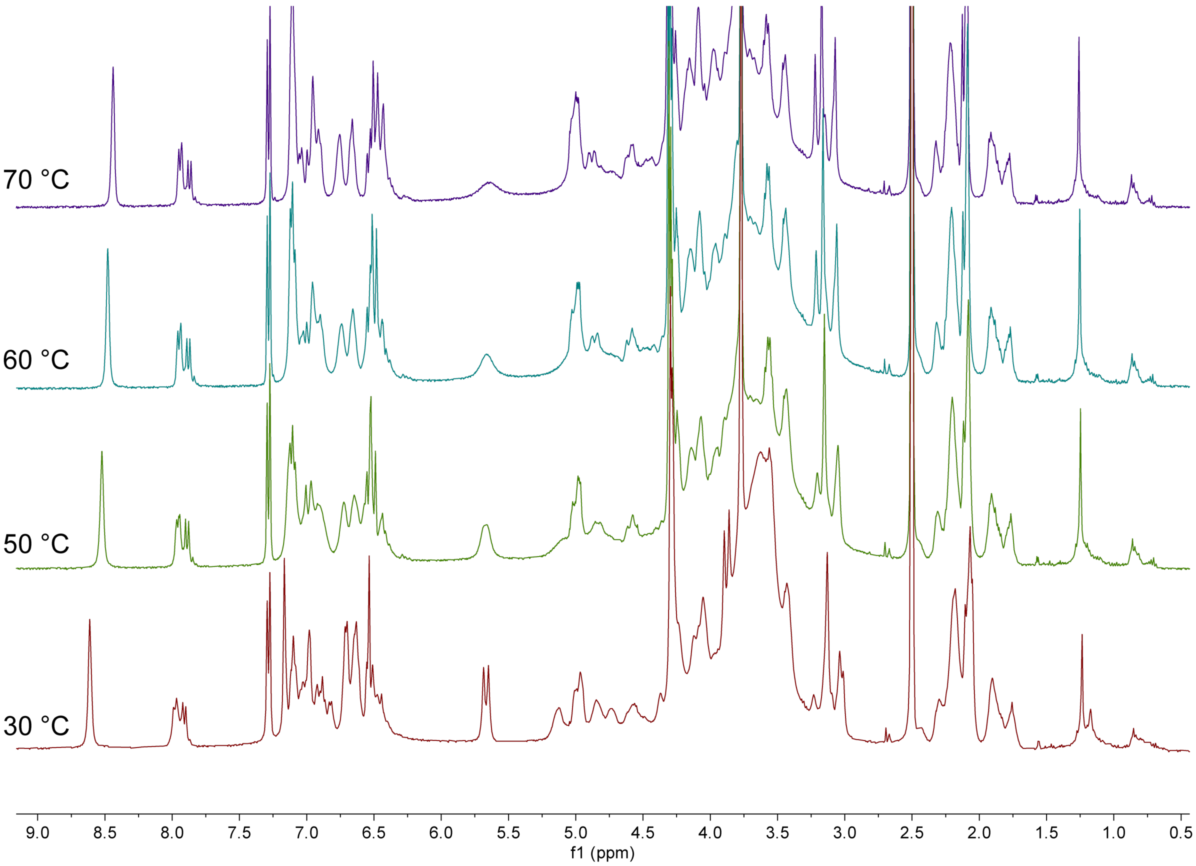

^{1}H-NMR of compound **37** in DMSO-d_6 at different temperatures.

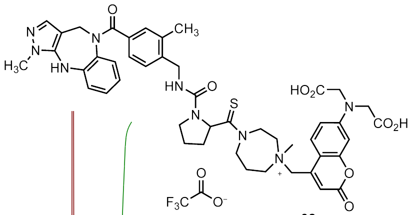

N
N
H3C
HN
O
N
CH3
HN
O
N
S
N
N
+
HO2C
N
CO2H
O
O
O
F3C
O-

38

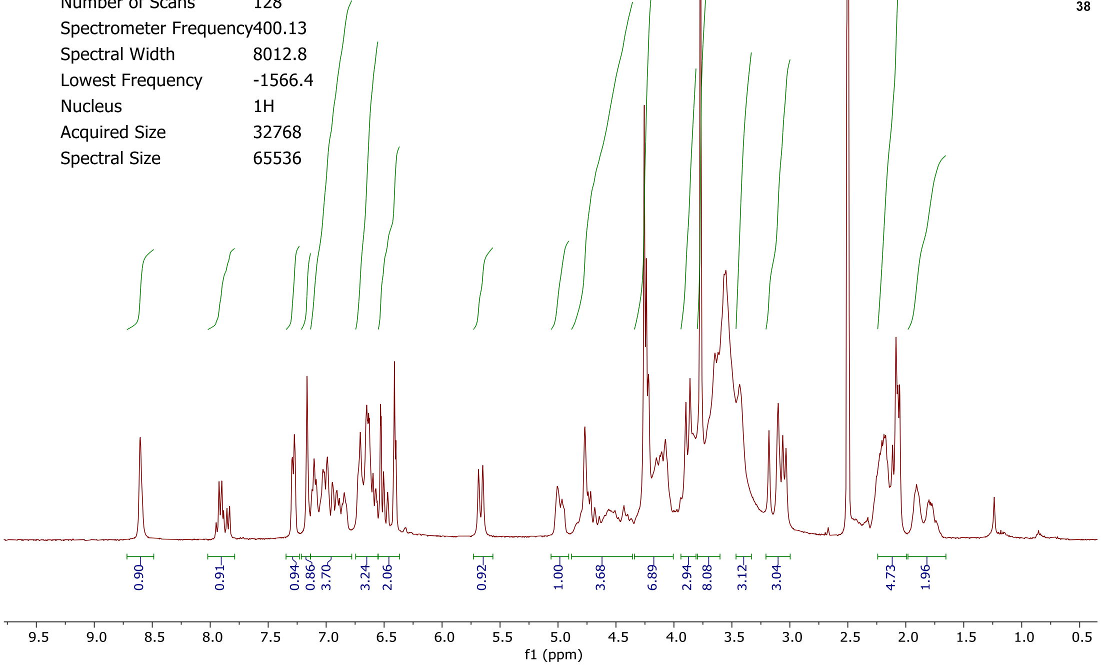

Origin Bruker BioSpin GmbH
Solvent DMSO
Temperature 303.0
Pulse Sequence zg30
Experiment 1D
Number of Scans 128
Spectrometer Frequency 400.13
Spectral Width 8012.8
Lowest Frequency -1566.4
Nucleus 1H
Acquired Size 32768
Spectral Size 65536
0.90
0.91
0.94
0.86
3.70
3.24
2.06
0.92
1.00
3.68
6.89
2.94
8.08
3.12
3.04
4.73
1.96
9.5 9.0 8.5 8.0 7.5 7.0 6.5 6.0 5.5 5.0 4.5 4.0 3.5 3.0 2.5 2.0 1.5 1.0 0.5
f1 (ppm)

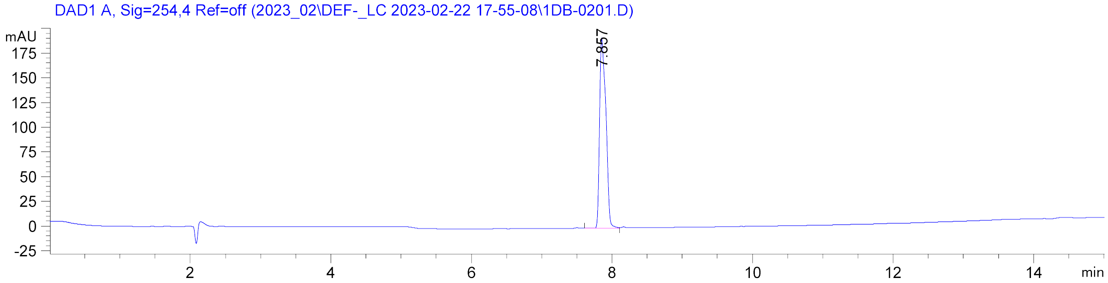

DAD1 A, Sig=254,4 Ref=off (2023_02\DEF-_LC 2023-02-22 17-55-08\1DB-0201.D)
mAU
175
150
125
100
75
50
25
0
-25
7.857
2 4 6 8 10 12 14 min

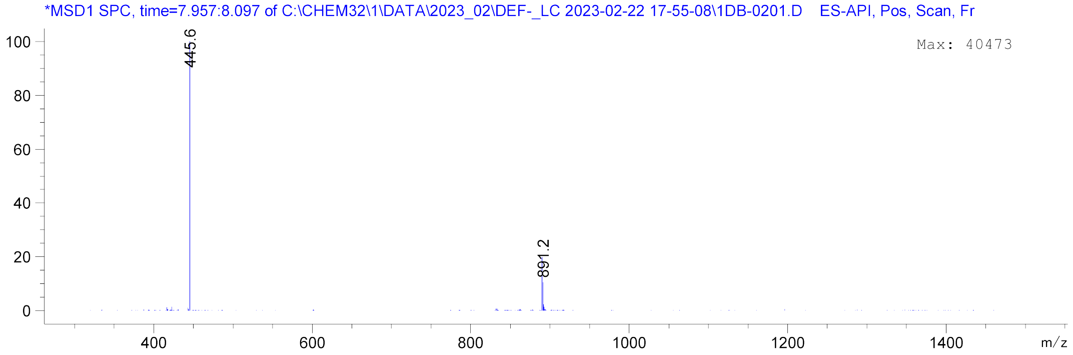

*MSD1 SPC, time=7.957:8.097 of C:\CHEM32\1\DATA\2023_02\DEF-_LC 2023-02-22 17-55-08\1DB-0201.D ES-API, Pos, Scan, Fr
Max: 40473
100
80
60
40
20
0
445.6
891.2
400 600 800 1000 1200 1400 m/z

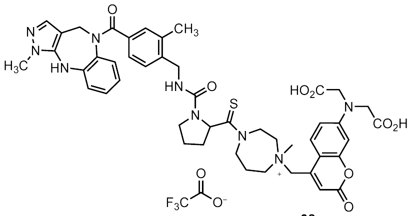

38

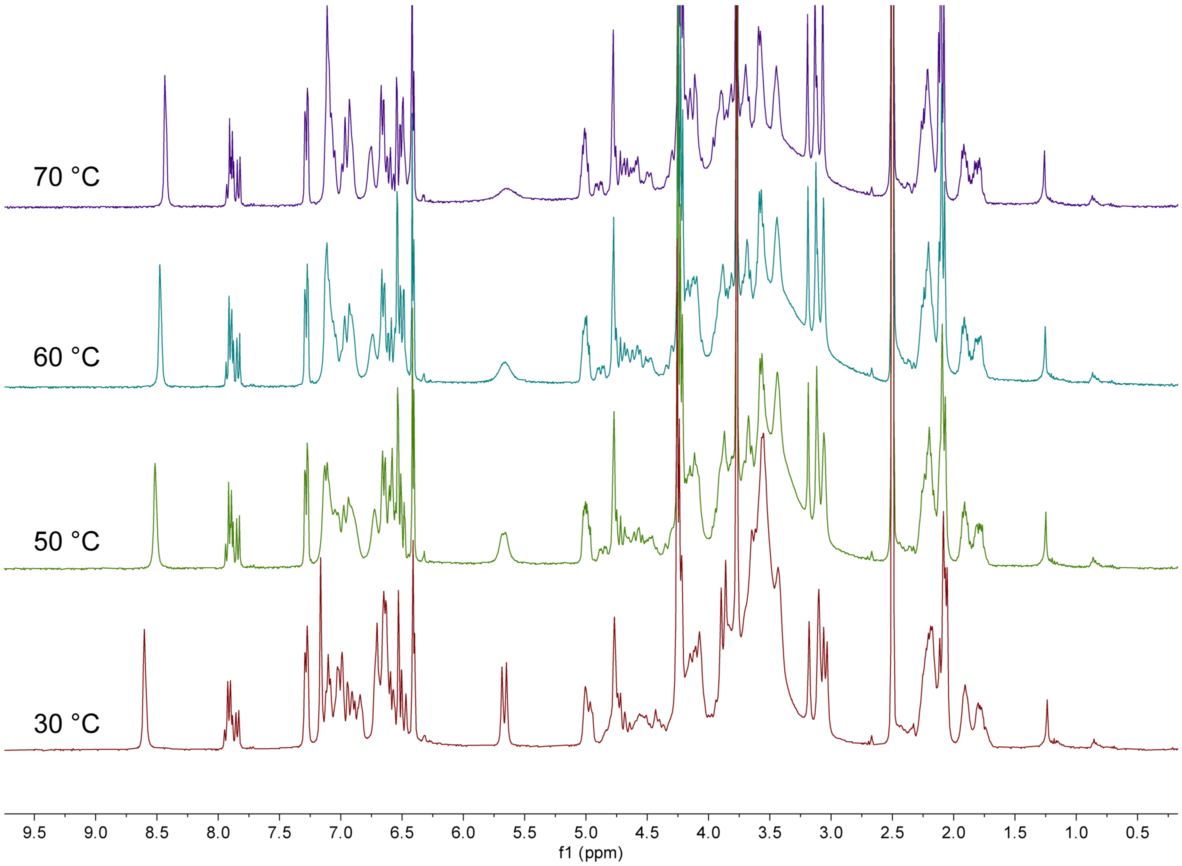

^{1}H-NMR of compound **38** in DMSO-d_6 at different temperatures.

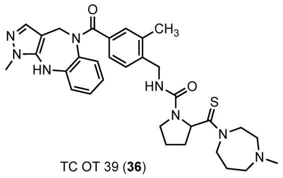

TC OT 39 (**36**)

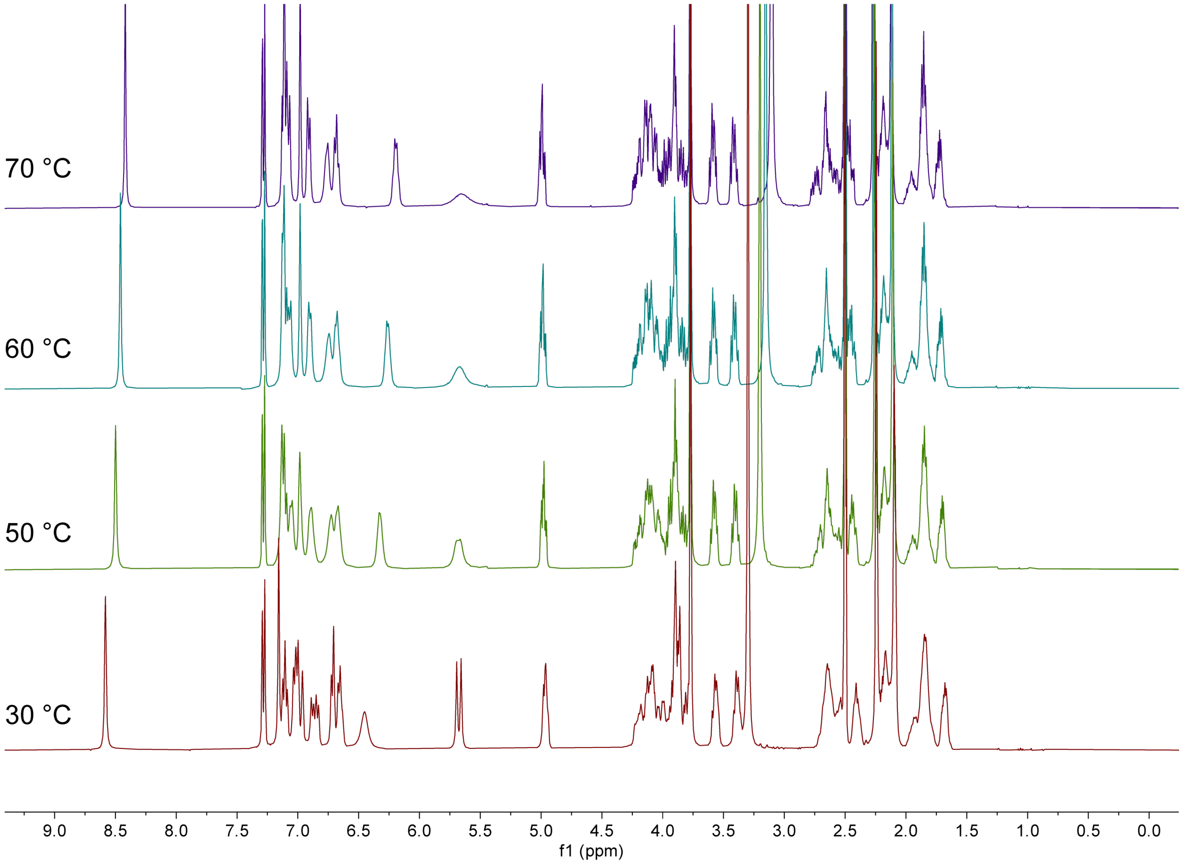

^{1}H-NMR of compound **36**, used for synthesis of **37** and **38**, in DMSO-d_6 at different temperatures.

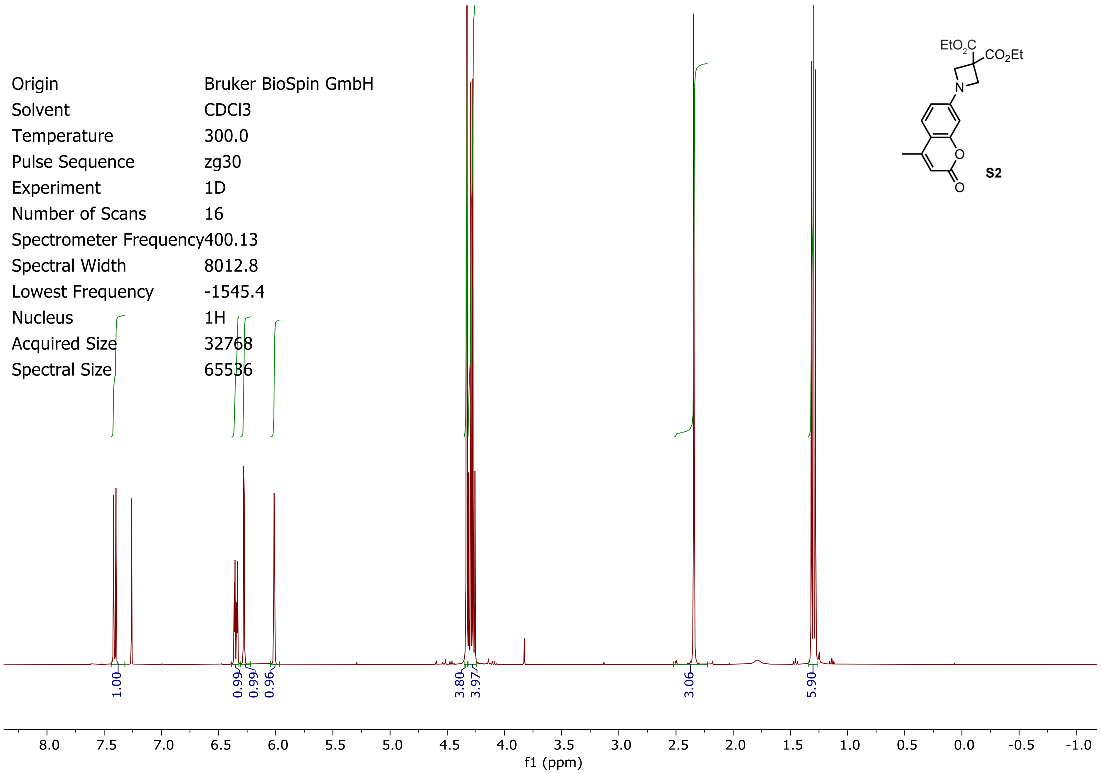
EtO2C
CO2Et
N
O
O
S2
Origin Bruker BioSpin GmbH
Solvent CDCl3
Temperature 300.0
Pulse Sequence zg30
Experiment 1D
Number of Scans 16
Spectrometer Frequency 400.13
Spectral Width 8012.8
Lowest Frequency -1545.4
Nucleus 1H
Acquired Size 32768
Spectral Size 65536
1.00
0.99
0.99
0.96
3.80
3.97
3.06
5.90
8.0 7.5 7.0 6.5 6.0 5.5 5.0 4.5 4.0 3.5 3.0 2.5 2.0 1.5 1.0 0.5 0.0 -0.5 -1.0
f1 (ppm)

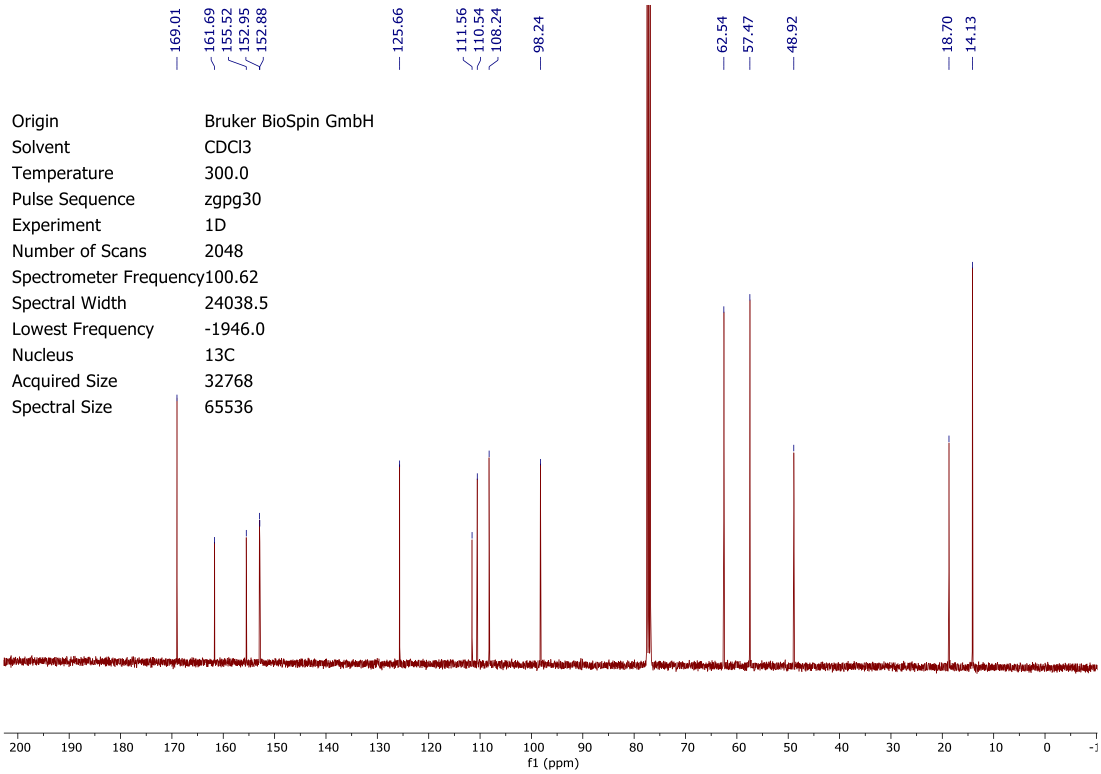
169.01
161.69
155.52
152.95
152.88
125.66
111.56
110.54
108.24
98.24
62.54
57.47
48.92
18.70
14.13
Origin Bruker BioSpin GmbH
Solvent CDCl3
Temperature 300.0
Pulse Sequence zgpg30
Experiment 1D
Number of Scans 2048
Spectrometer Frequency 100.62
Spectral Width 24038.5
Lowest Frequency -1946.0
Nucleus 13C
Acquired Size 32768
Spectral Size 65536
200 190 180 170 160 150 140 130 120 110 100 90 80 70 60 50 40 30 20 10 0
f1 (ppm)

S3

Origin	Bruker BioSpin GmbH
Solvent	CDCl3
Temperature	295.4
Pulse Sequence	zg30
Experiment	1D
Number of Scans	16
Spectrometer Frequency	400.13
Spectral Width	8012.8
Lowest Frequency	-1545.4
Nucleus	1H
Acquired Size	32768
Spectral Size	65536

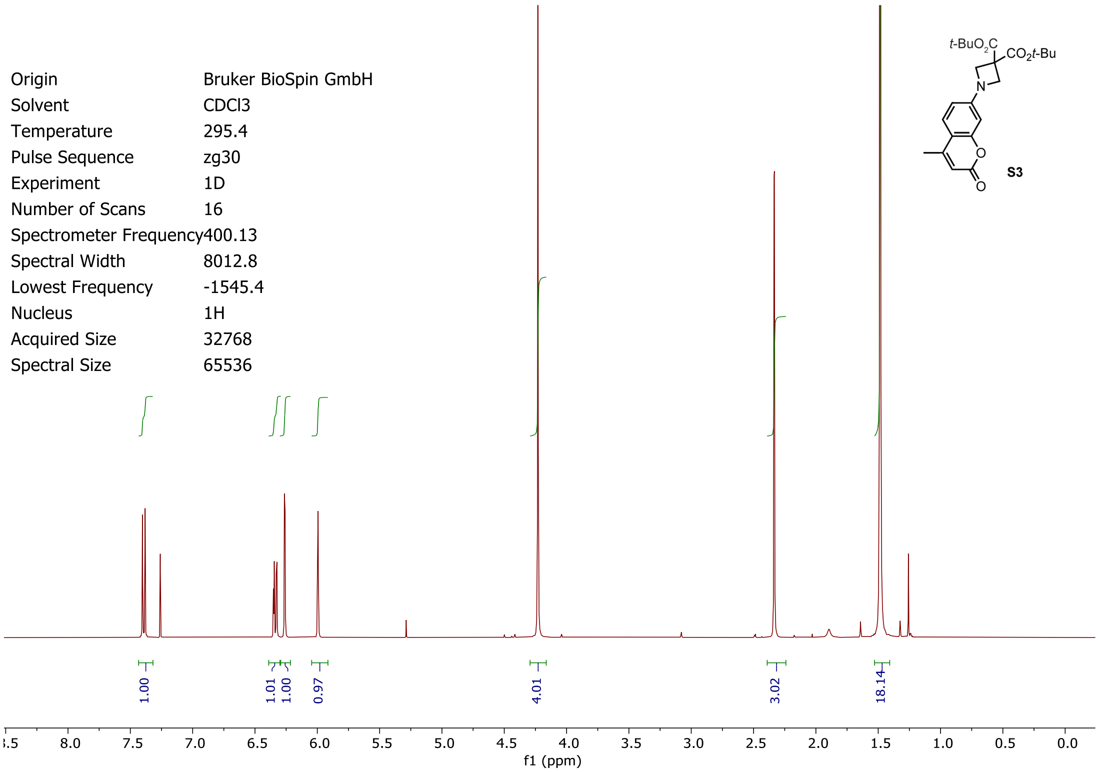

Origin	Bruker BioSpin GmbH
Solvent	CDCl3
Temperature	296.0
Pulse Sequence	zgpg30
Experiment	1D
Number of Scans	4096
Spectrometer Frequency	100.62
Spectral Width	24038.5
Lowest Frequency	-1948.6
Nucleus	13C
Acquired Size	32768
Spectral Size	65536

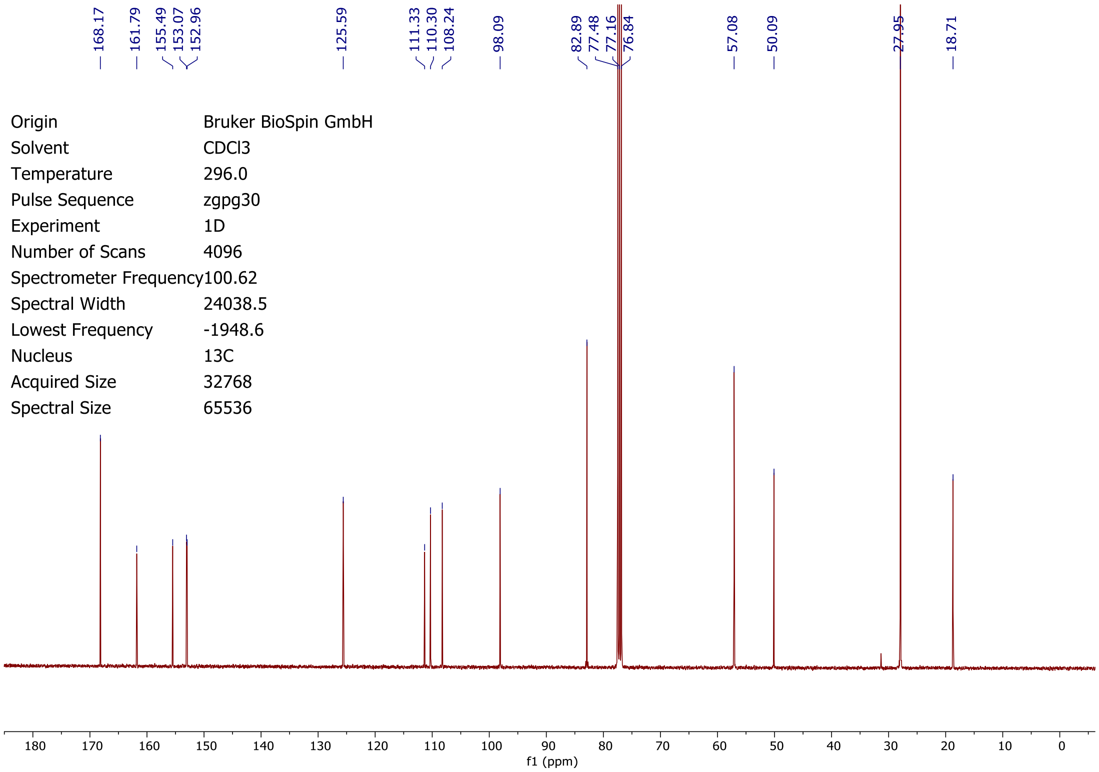

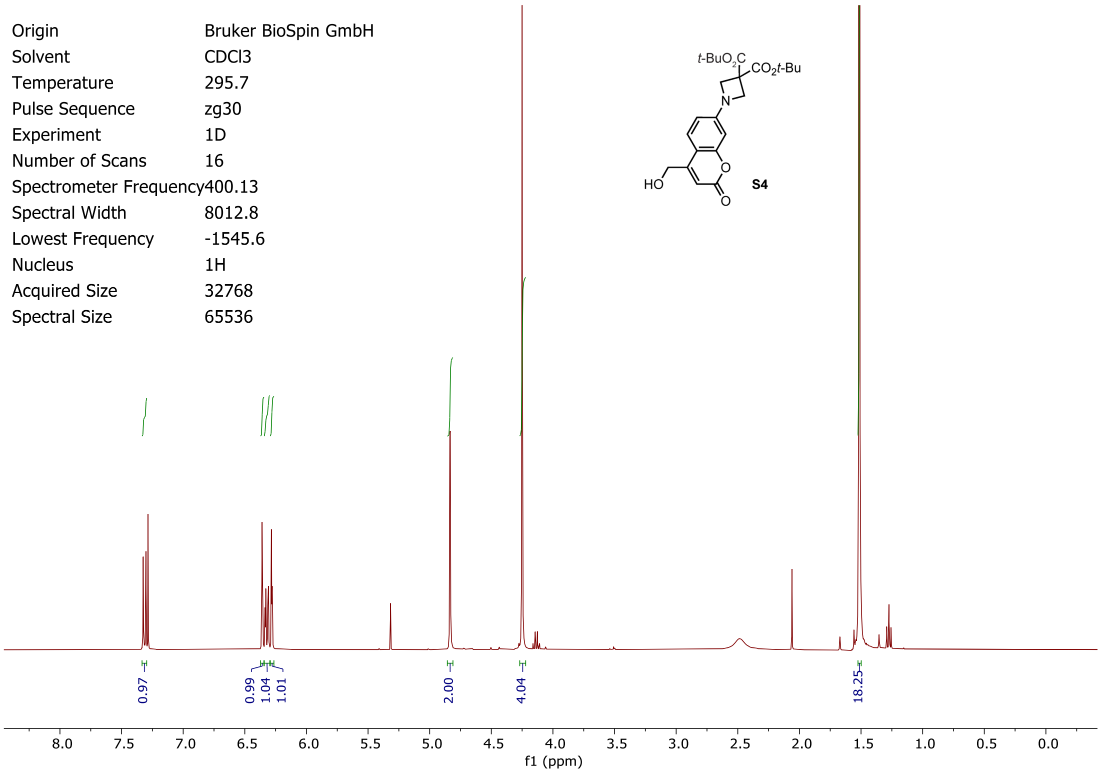
Origin Bruker BioSpin GmbH
Solvent CDCl3
Temperature 295.7
Pulse Sequence zg30
Experiment 1D
Number of Scans 16
Spectrometer Frequency 400.13
Spectral Width 8012.8
Lowest Frequency -1545.6
Nucleus 1H
Acquired Size 32768
Spectral Size 65536
t-BuO2C
CO2t-Bu
N
HO
O
O
S4
0.97
0.99
1.04
1.01
2.00
4.04
18.25
8.0 7.5 7.0 6.5 6.0 5.5 5.0 4.5 4.0 3.5 3.0 2.5 2.0 1.5 1.0 0.5 0.0
f1 (ppm)

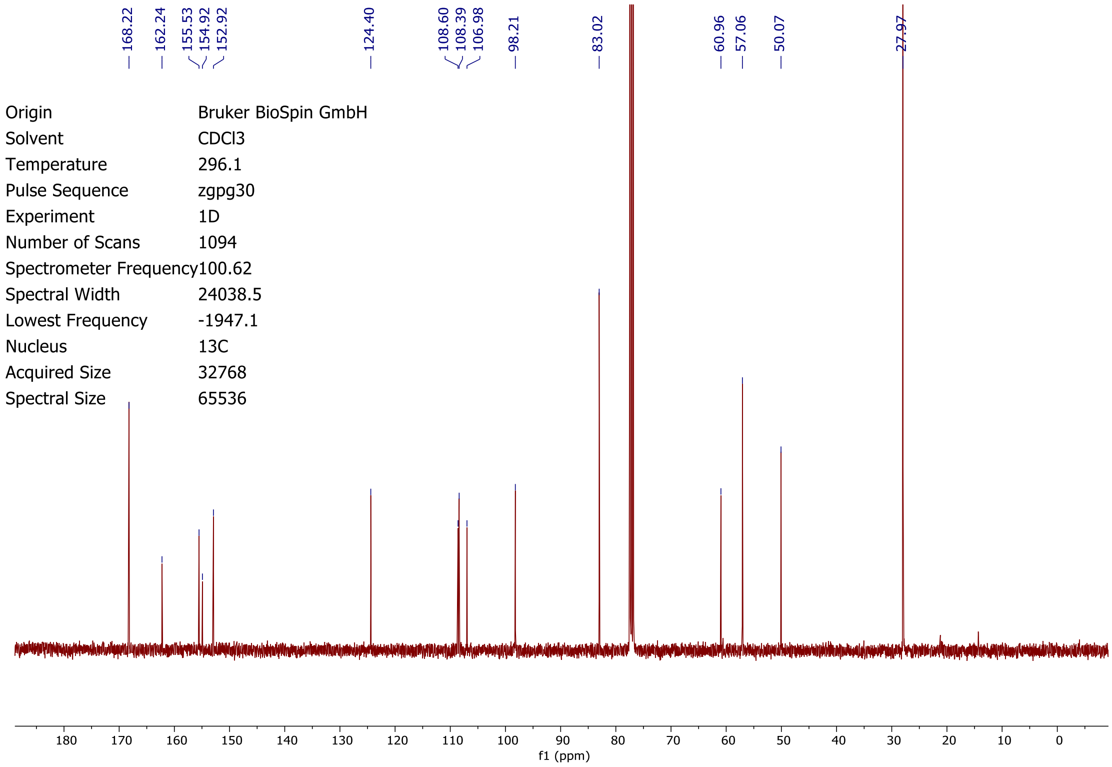
168.22
162.24
155.53
154.92
152.92
124.40
108.60
108.39
106.98
98.21
83.02
60.96
57.06
50.07
27.97
Origin Bruker BioSpin GmbH
Solvent CDCl3
Temperature 296.1
Pulse Sequence zgpg30
Experiment 1D
Number of Scans 1094
Spectrometer Frequency 100.62
Spectral Width 24038.5
Lowest Frequency -1947.1
Nucleus 13C
Acquired Size 32768
Spectral Size 65536
180 170 160 150 140 130 120 110 100 90 80 70 60 50 40 30 20 10 0
f1 (ppm)

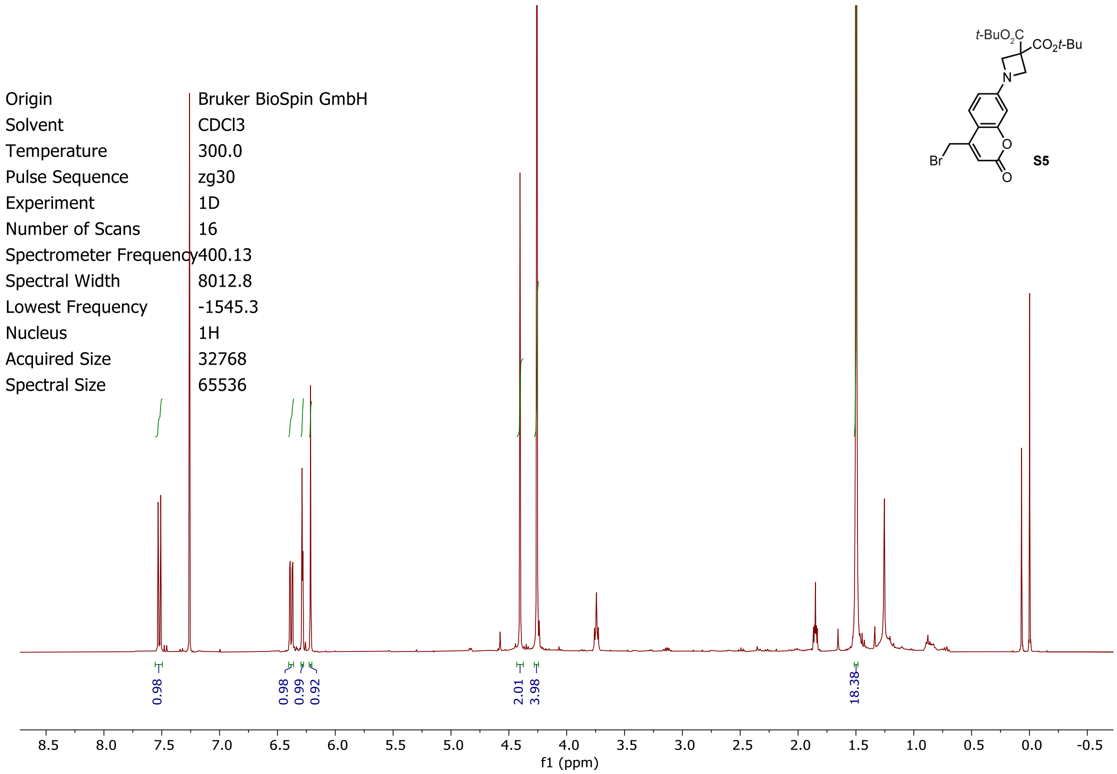
Origin Bruker BioSpin GmbH
Solvent CDCl3
Temperature 300.0
Pulse Sequence zg30
Experiment 1D
Number of Scans 16
Spectrometer Frequency 400.13
Spectral Width 8012.8
Lowest Frequency -1545.3
Nucleus 1H
Acquired Size 32768
Spectral Size 65536
t-BuO2C
CO2t-Bu
N
Br
O
O
S5
0.98
0.98
0.99
0.92
2.01
3.98
18.38
8.5 8.0 7.5 7.0 6.5 6.0 5.5 5.0 4.5 4.0 3.5 3.0 2.5 2.0 1.5 1.0 0.5 0.0 -0.5
f1 (ppm)

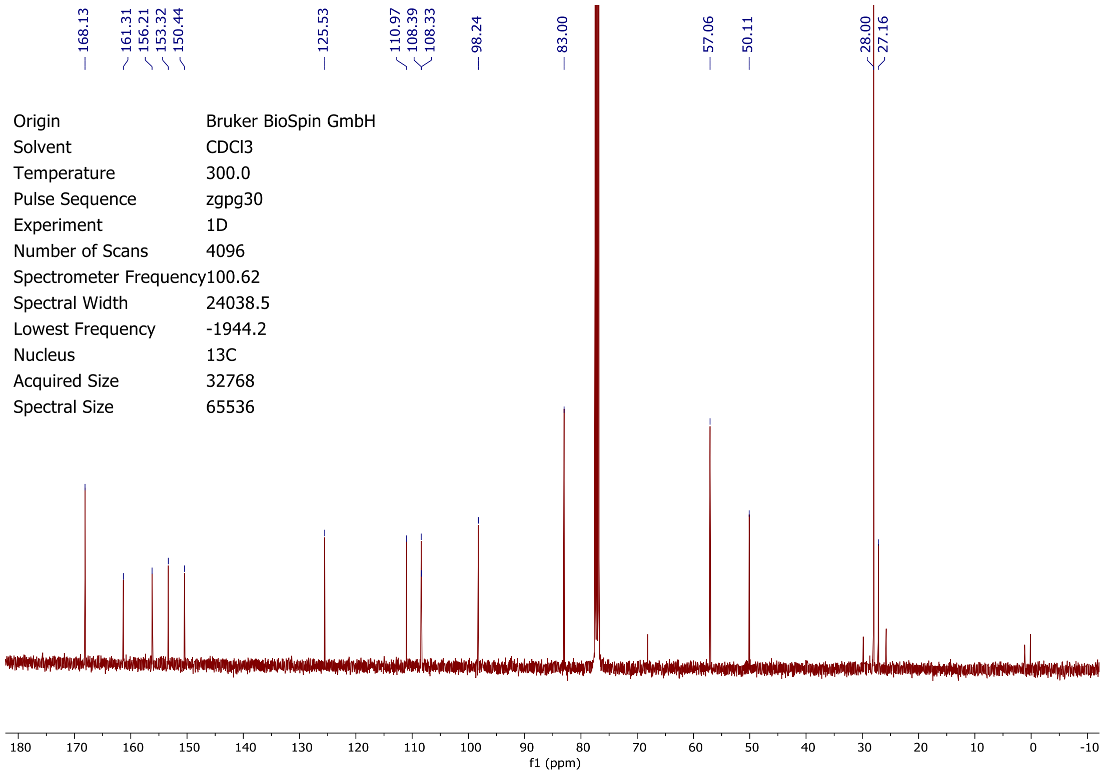
168.13
161.31
156.21
153.32
150.44
125.53
110.97
108.39
108.33
98.24
83.00
57.06
50.11
28.00
27.16
Origin Bruker BioSpin GmbH
Solvent CDCl3
Temperature 300.0
Pulse Sequence zgpg30
Experiment 1D
Number of Scans 4096
Spectrometer Frequency 100.62
Spectral Width 24038.5
Lowest Frequency -1944.2
Nucleus 13C
Acquired Size 32768
Spectral Size 65536
180 170 160 150 140 130 120 110 100 90 80 70 60 50 40 30 20 10 0 -10
f1 (ppm)

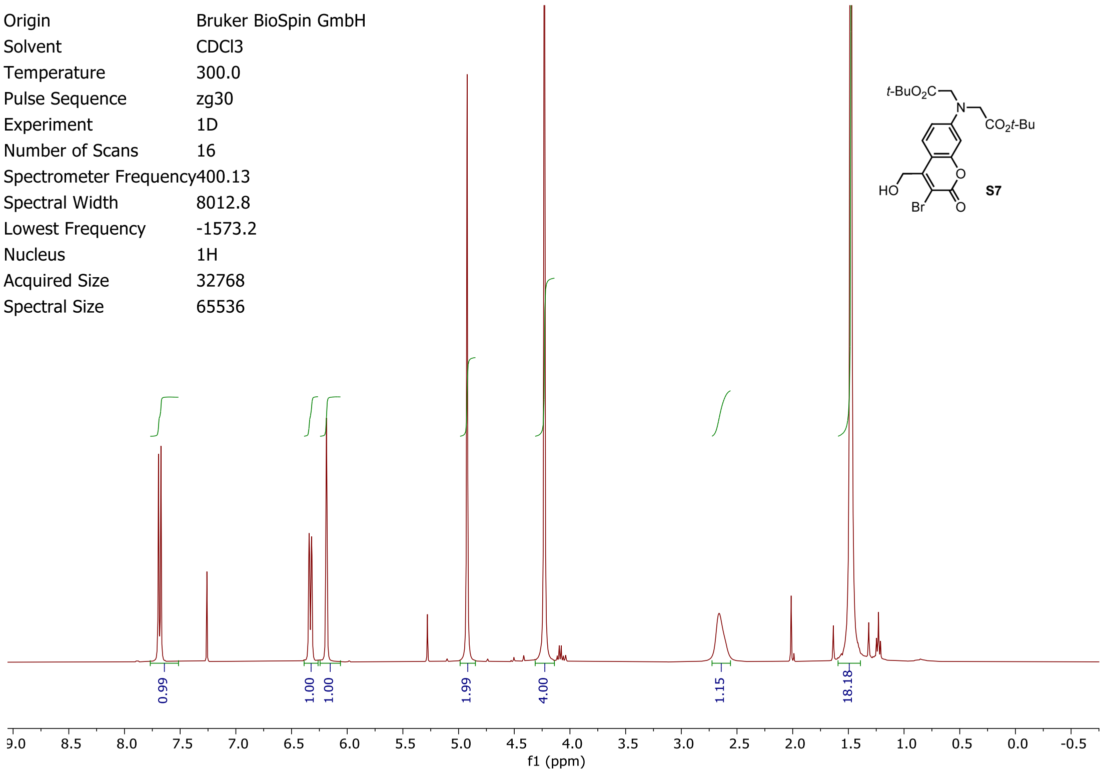

Origin Bruker BioSpin GmbH
Solvent CDCl3
Temperature 300.0
Pulse Sequence zg30
Experiment 1D
Number of Scans 16
Spectrometer Frequency 400.13
Spectral Width 8012.8
Lowest Frequency -1573.2
Nucleus 1H
Acquired Size 32768
Spectral Size 65536
0.99
1.00
1.00
1.99
4.00
1.15
18.18
f1 (ppm)
t-BuO2C
N
CO2t-Bu
HO
O
Br
O

S7

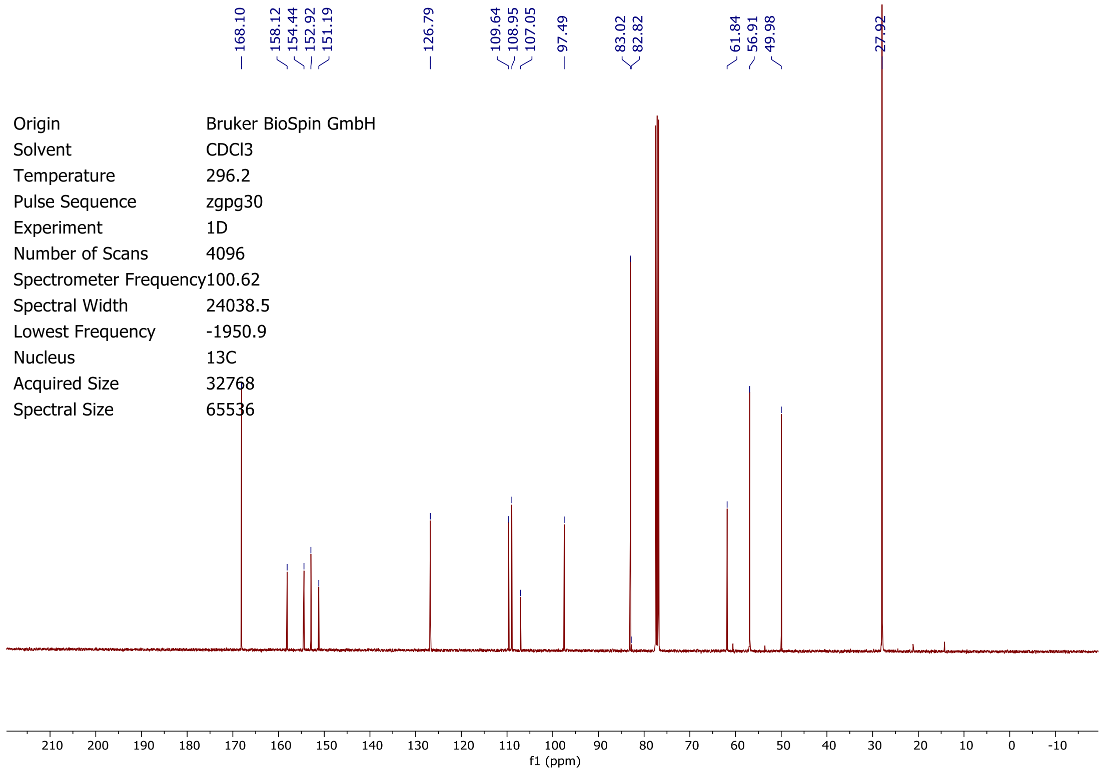

168.10
158.12
154.44
152.92
151.19
126.79
109.64
108.95
107.05
97.49
83.02
82.82
61.84
56.91
49.98
27.92
Origin Bruker BioSpin GmbH
Solvent CDCl3
Temperature 296.2
Pulse Sequence zgpg30
Experiment 1D
Number of Scans 4096
Spectrometer Frequency 100.62
Spectral Width 24038.5
Lowest Frequency -1950.9
Nucleus 13C
Acquired Size 32768
Spectral Size 65536
f1 (ppm)

Origin	Bruker BioSpin GmbH
Solvent	CDCl3
Temperature	300.0
Pulse Sequence	zg30
Experiment	1D
Number of Scans	16
Spectrometer Frequency	400.13
Spectral Width	8012.8
Lowest Frequency	-1545.6
Nucleus	1H
Acquired Size	32768
Spectral Size	65536

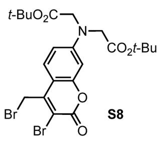

S8

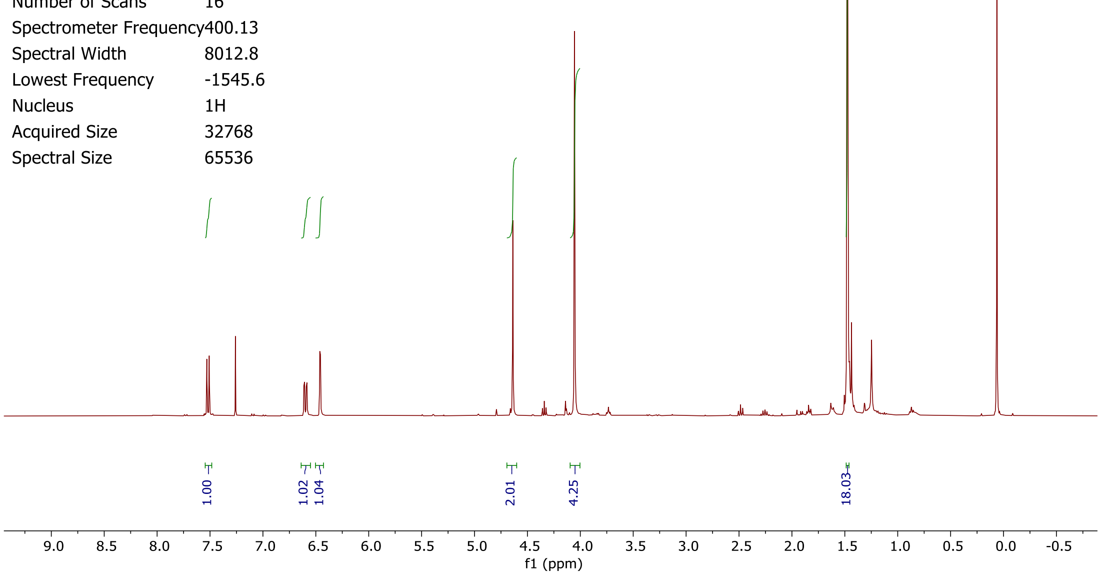

Origin	Bruker BioSpin GmbH
Solvent	CDCl3
Temperature	300.0
Pulse Sequence	zgpg30
Experiment	1D
Number of Scans	3084
Spectrometer Frequency	100.62
Spectral Width	24038.5
Lowest Frequency	-1946.0
Nucleus	13C
Acquired Size	32768

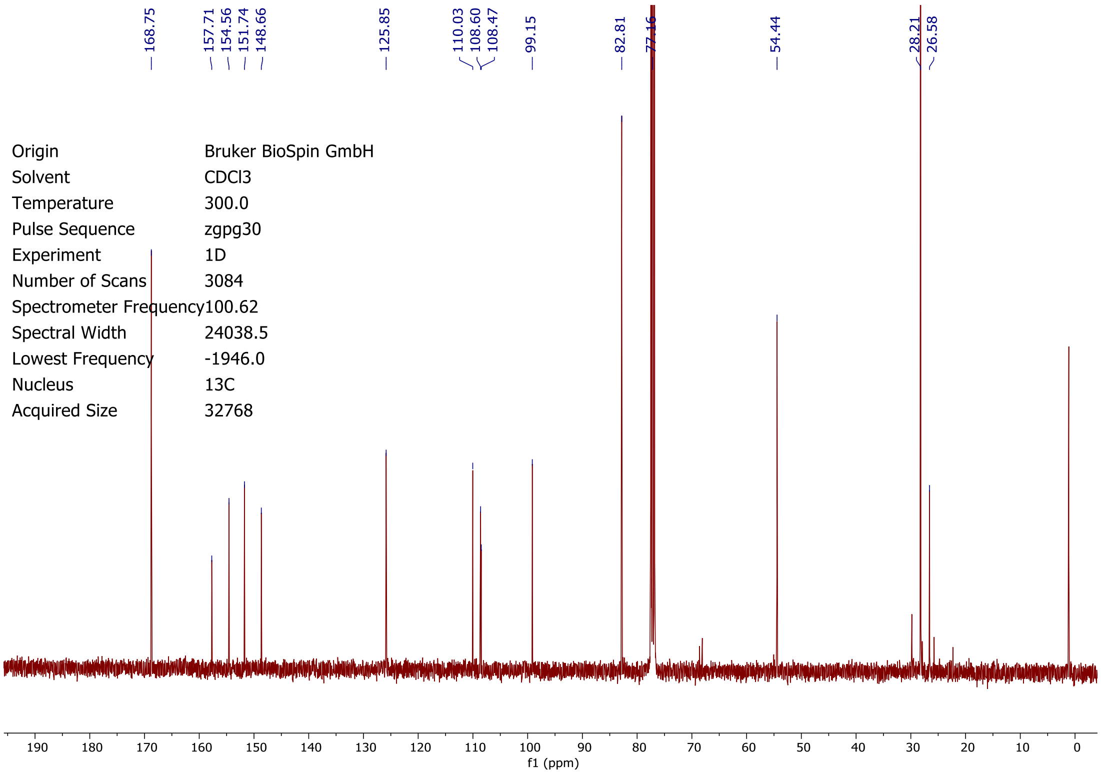

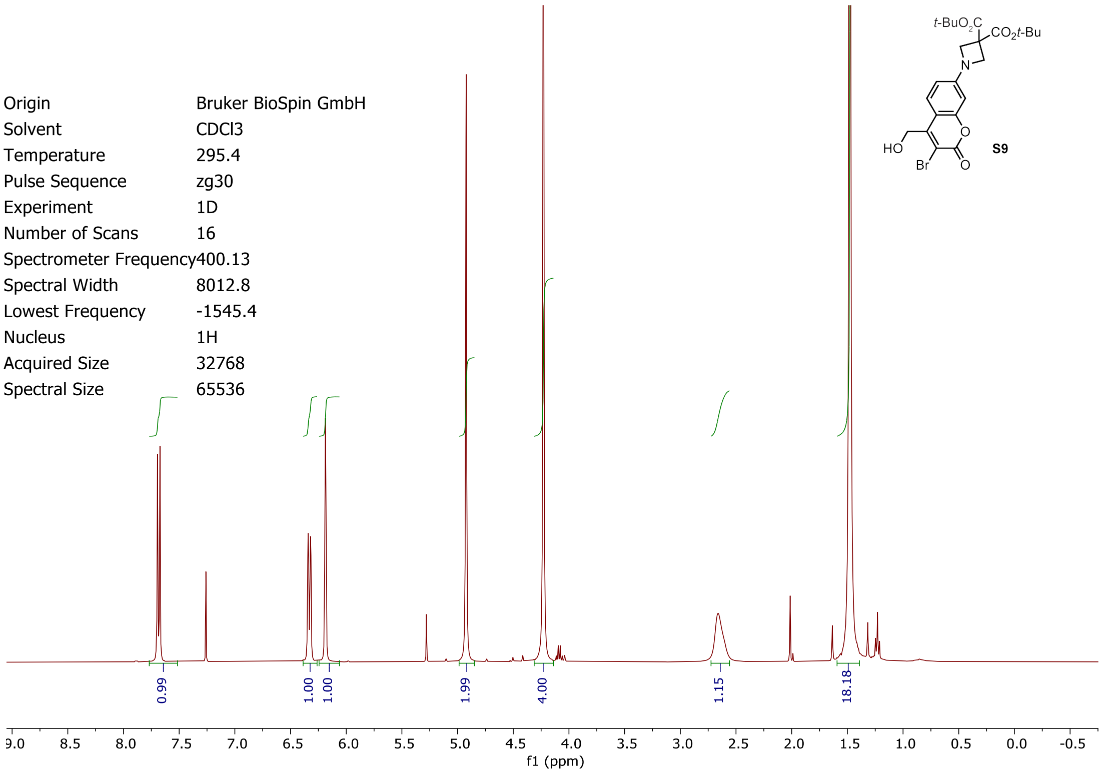
t-BuO2C
CO2t-Bu
N
HO
O
Br
O
S9
Origin Bruker BioSpin GmbH
Solvent CDCl3
Temperature 295.4
Pulse Sequence zg30
Experiment 1D
Number of Scans 16
Spectrometer Frequency 400.13
Spectral Width 8012.8
Lowest Frequency -1545.4
Nucleus 1H
Acquired Size 32768
Spectral Size 65536
0.99
1.00
1.00
1.99
4.00
1.15
18.18
9.0 8.5 8.0 7.5 7.0 6.5 6.0 5.5 5.0 4.5 4.0 3.5 3.0 2.5 2.0 1.5 1.0 0.5 0.0 -0.5
f1 (ppm)

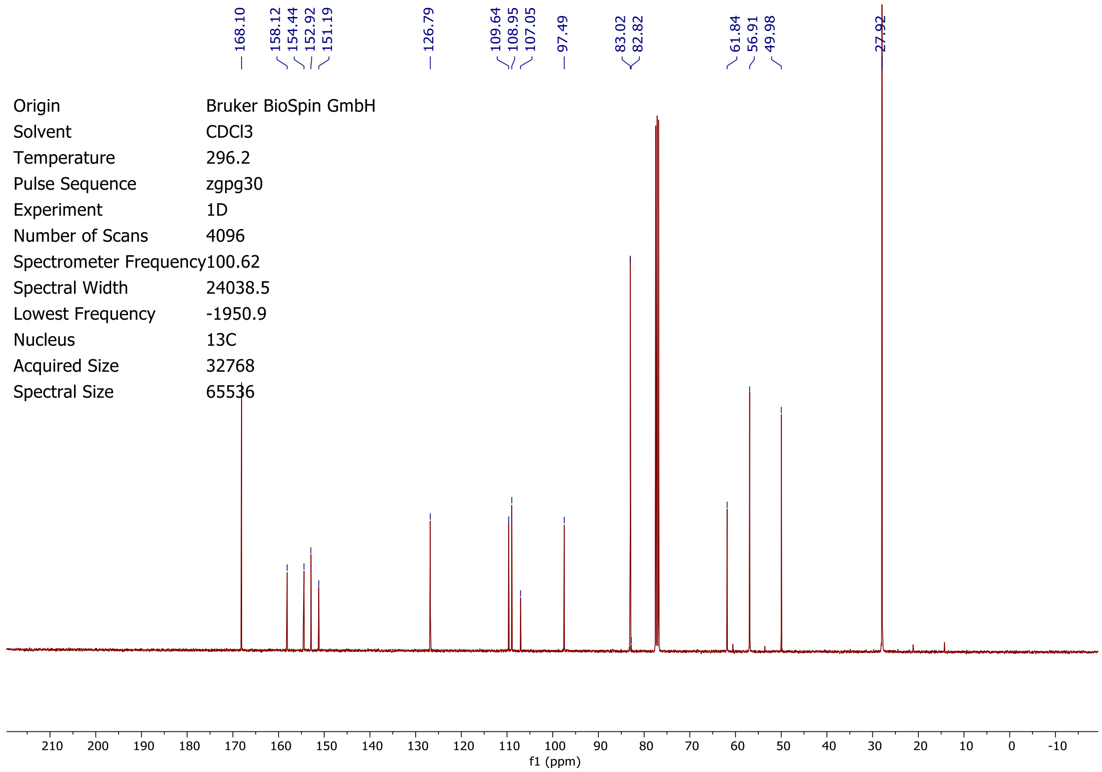
168.10
158.12
154.44
152.92
151.19
126.79
109.64
108.95
107.05
97.49
83.02
82.82
61.84
56.91
49.98
27.92
Origin Bruker BioSpin GmbH
Solvent CDCl3
Temperature 296.2
Pulse Sequence zgpg30
Experiment 1D
Number of Scans 4096
Spectrometer Frequency 100.62
Spectral Width 24038.5
Lowest Frequency -1950.9
Nucleus 13C
Acquired Size 32768
Spectral Size 65536
210 200 190 180 170 160 150 140 130 120 110 100 90 80 70 60 50 40 30 20 10 0 -10
f1 (ppm)

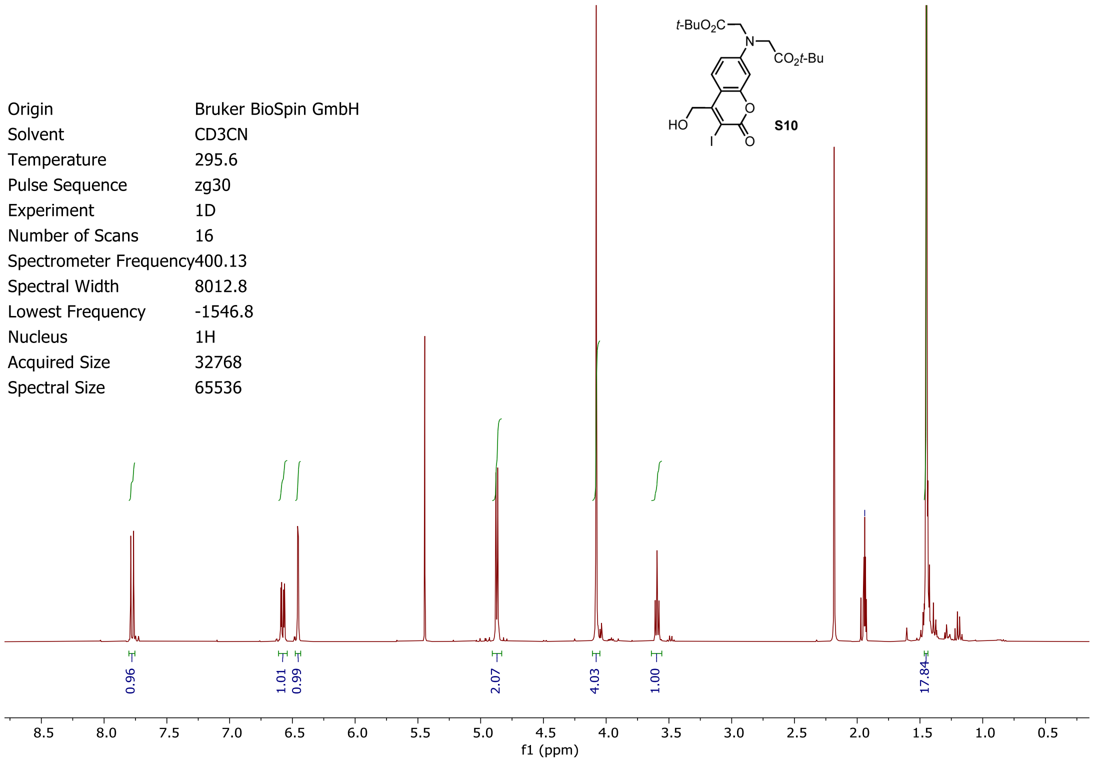

t-BuO2C
N
CO2t-Bu
HO
O
I
O
S10
Origin Bruker BioSpin GmbH
Solvent CD3CN
Temperature 295.6
Pulse Sequence zg30
Experiment 1D
Number of Scans 16
Spectrometer Frequency 400.13
Spectral Width 8012.8
Lowest Frequency -1546.8
Nucleus 1H
Acquired Size 32768
Spectral Size 65536
0.96
1.01
0.99
2.07
4.03
1.00
17.84
8.5 8.0 7.5 7.0 6.5 6.0 5.5 5.0 4.5 4.0 3.5 3.0 2.5 2.0 1.5 1.0 0.5
f1 (ppm)

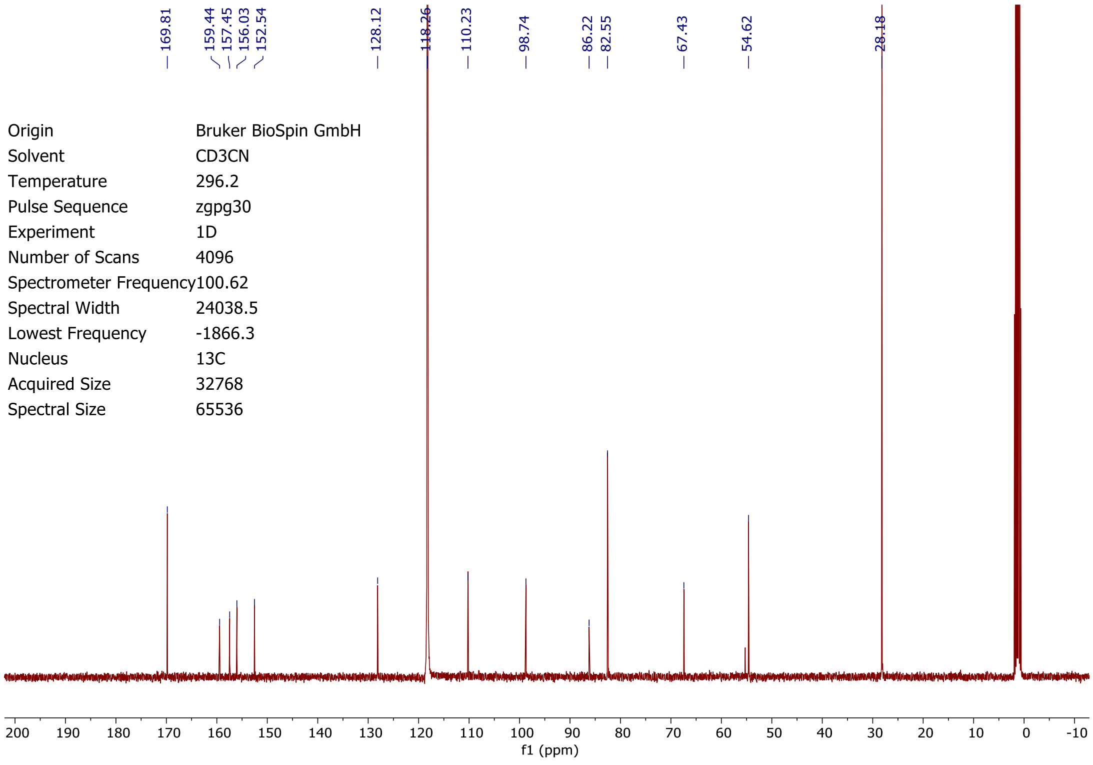

169.81
159.44
157.45
156.03
152.54
128.12
118.26
110.23
98.74
86.22
82.55
67.43
54.62
28.18
Origin Bruker BioSpin GmbH
Solvent CD3CN
Temperature 296.2
Pulse Sequence zgpg30
Experiment 1D
Number of Scans 4096
Spectrometer Frequency 100.62
Spectral Width 24038.5
Lowest Frequency -1866.3
Nucleus 13C
Acquired Size 32768
Spectral Size 65536
200 190 180 170 160 150 140 130 120 110 100 90 80 70 60 50 40 30 20 10 0 -10
f1 (ppm)

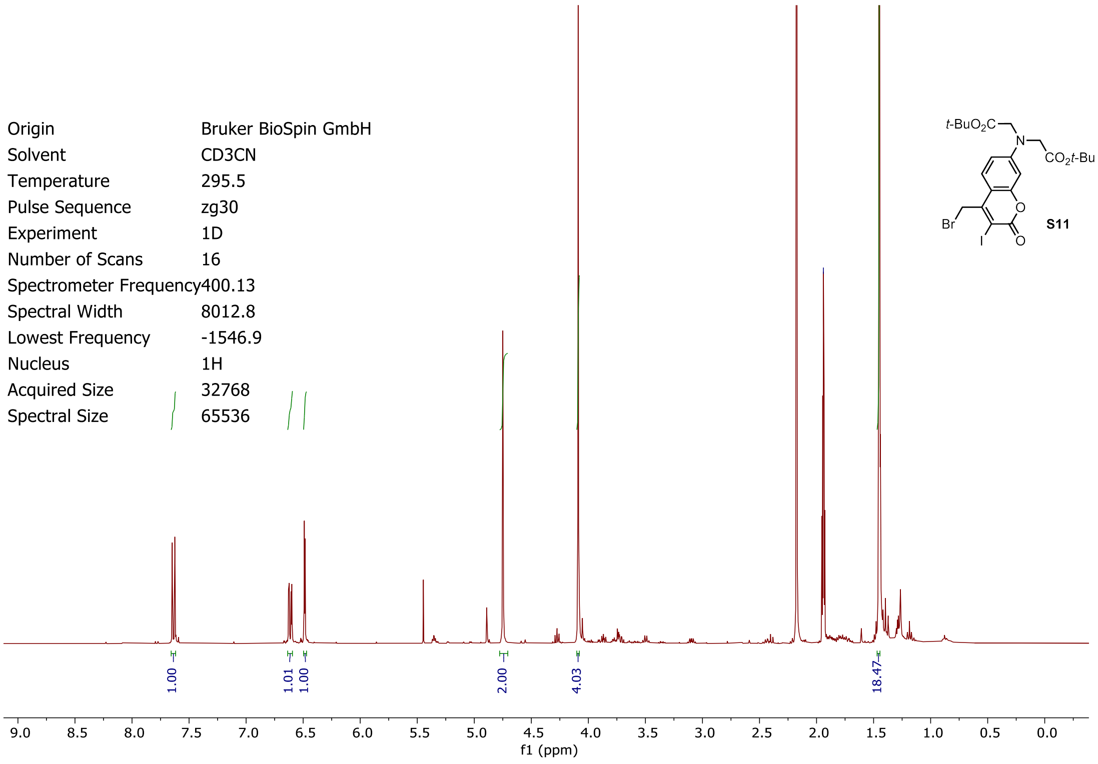
Origin Bruker BioSpin GmbH
Solvent CD3CN
Temperature 295.5
Pulse Sequence zg30
Experiment 1D
Number of Scans 16
Spectrometer Frequency 400.13
Spectral Width 8012.8
Lowest Frequency -1546.9
Nucleus 1H
Acquired Size 32768
Spectral Size 65536
1.00
1.01
1.00
2.00
4.03
18.47
f1 (ppm)
t-BuO2C
N
CO2t-Bu
O
Br
I
O

S11

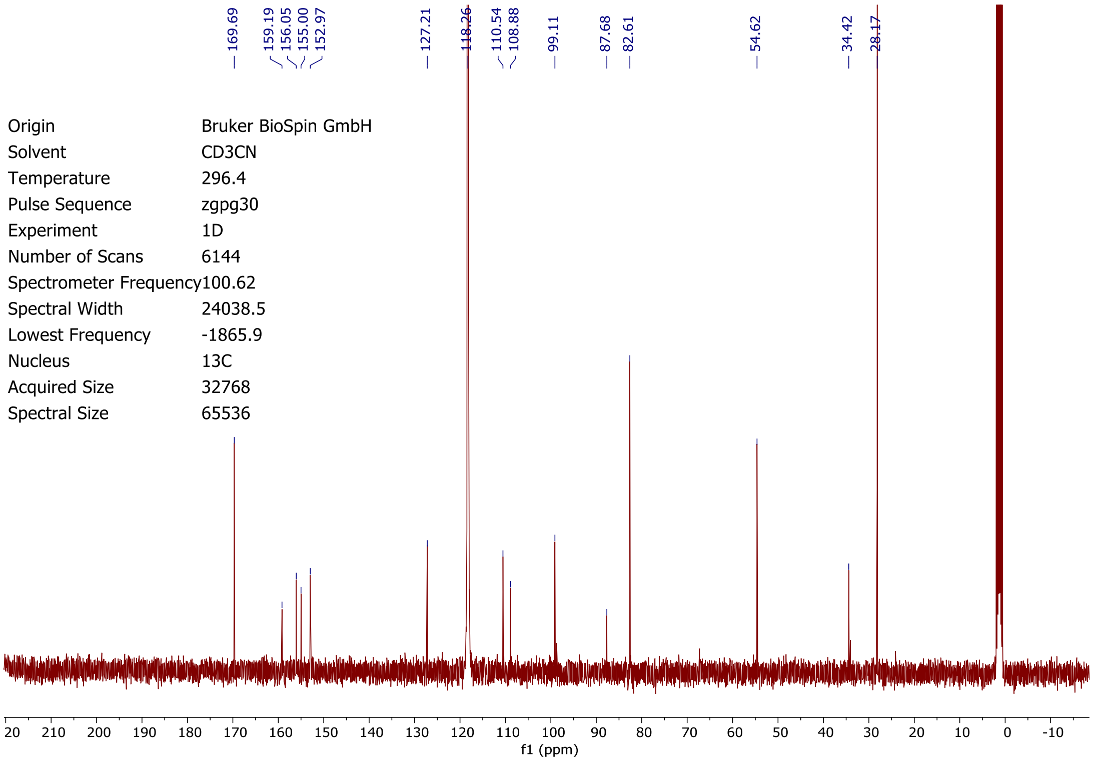
169.69
159.19
156.05
155.00
152.97
127.21
118.26
110.54
108.88
99.11
87.68
82.61
54.62
34.42
28.17
Origin Bruker BioSpin GmbH
Solvent CD3CN
Temperature 296.4
Pulse Sequence zgpg30
Experiment 1D
Number of Scans 6144
Spectrometer Frequency 100.62
Spectral Width 24038.5
Lowest Frequency -1865.9
Nucleus 13C
Acquired Size 32768
Spectral Size 65536
f1 (ppm)

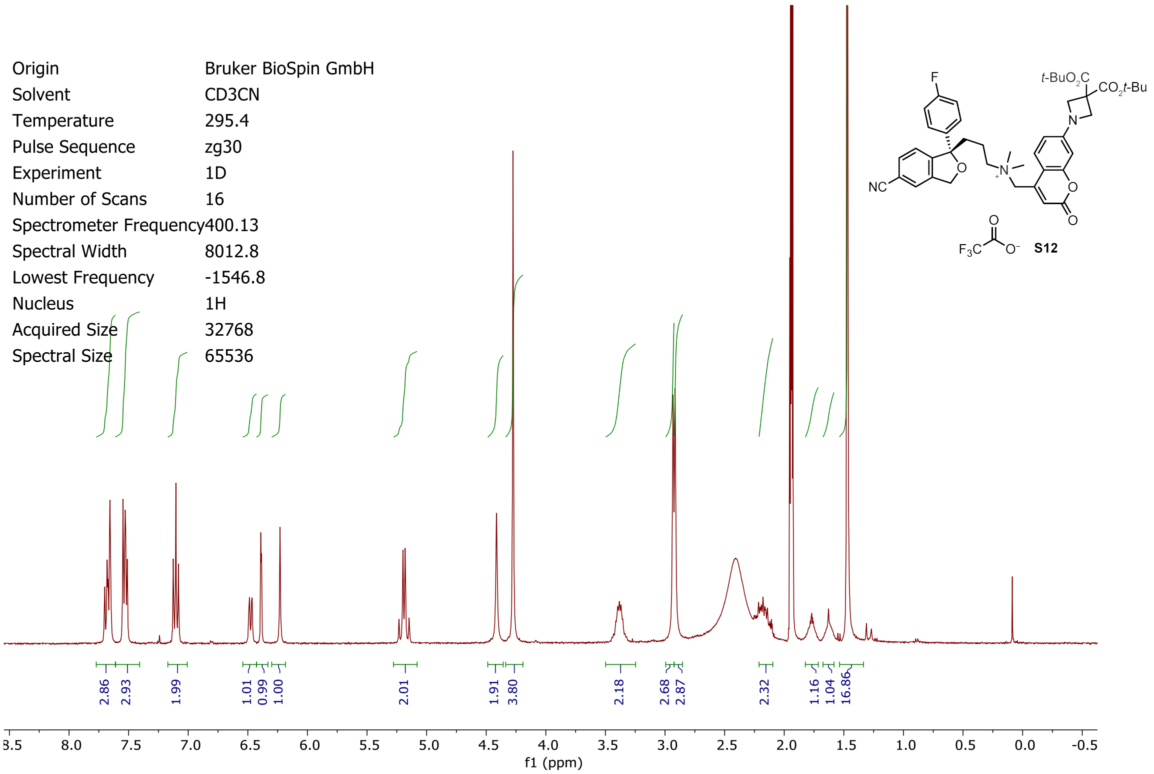

Origin Bruker BioSpin GmbH
Solvent CD3CN
Temperature 295.4
Pulse Sequence zg30
Experiment 1D
Number of Scans 16
Spectrometer Frequency 400.13
Spectral Width 8012.8
Lowest Frequency -1546.8
Nucleus 1H
Acquired Size 32768
Spectral Size 65536
2.86 2.93 1.99 1.01 0.99 1.00 2.01 1.91 3.80 2.18 2.68 2.87 2.32 1.16 1.04 16.86
f1 (ppm)
F
NC
O
N
t-BuO2C
CO2t-Bu
F3C
O⁻
S12

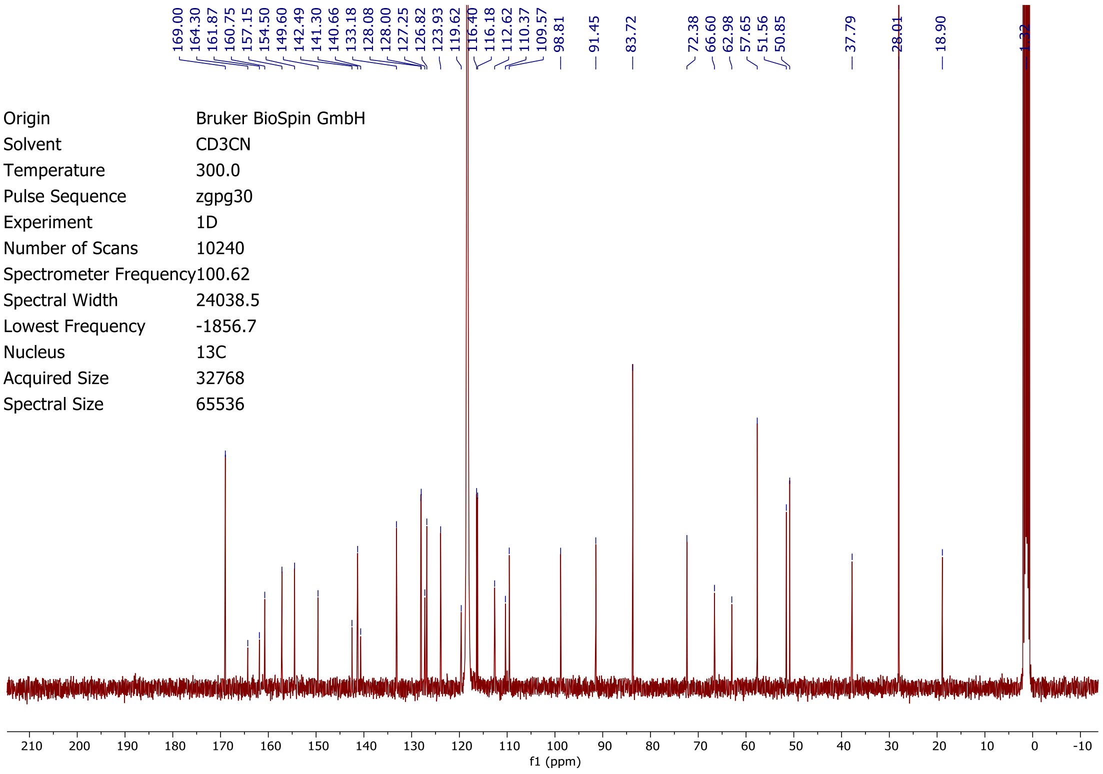

169.00 164.30 161.87 160.75 157.15 154.50 149.60 142.49 141.30 140.66 133.18 128.08 128.00 127.25 126.82 123.93 119.62 116.40 116.18 112.62 110.37 109.57 98.81 91.45 83.72 72.38 66.60 62.98 57.65 51.56 50.85 37.79 28.01 18.90
Origin Bruker BioSpin GmbH
Solvent CD3CN
Temperature 300.0
Pulse Sequence zgpg30
Experiment 1D
Number of Scans 10240
Spectrometer Frequency 100.62
Spectral Width 24038.5
Lowest Frequency -1856.7
Nucleus 13C
Acquired Size 32768
Spectral Size 65536
f1 (ppm)

Origin	Bruker BioSpin GmbH
Solvent	CD3CN
Temperature	295.4
Pulse Sequence	zg30
Experiment	1D
Number of Scans	16
Spectrometer Frequency	400.13
Spectral Width	8012.8
Lowest Frequency	-1546.8
Nucleus	1H
Acquired Size	32768
Spectral Size	65536

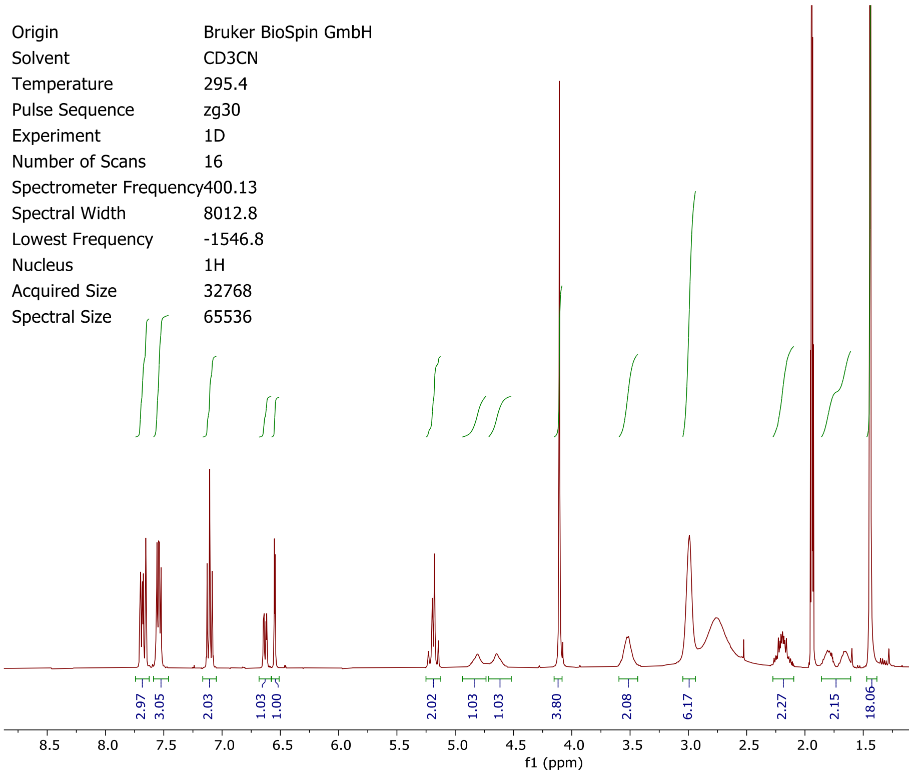

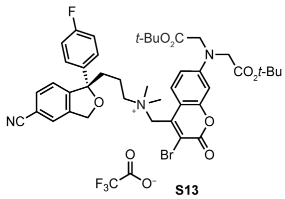

S13

Origin	Bruker BioSpin GmbH
Solvent	CD3CN
Temperature	296.2
Pulse Sequence	zgpg30
Experiment	1D
Number of Scans	10240
Spectrometer Frequency	100.62
Spectral Width	24038.5
Lowest Frequency	-1866.2
Nucleus	13C
Acquired Size	32768
Spectral Size	65536

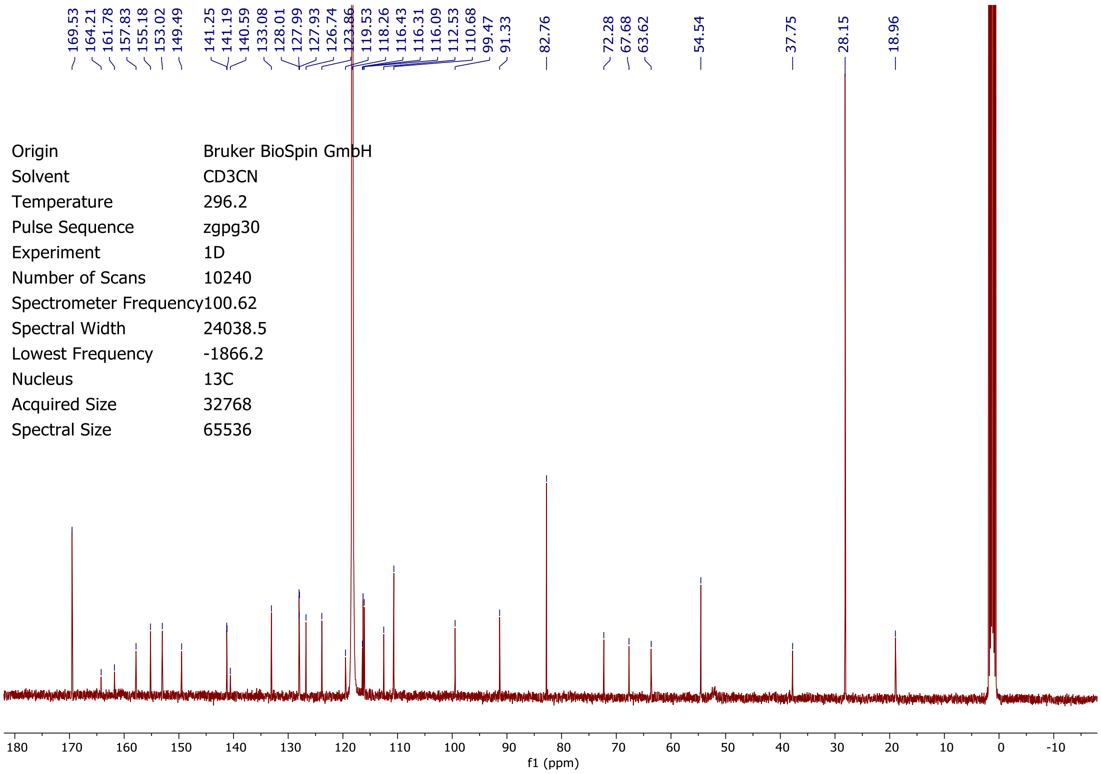

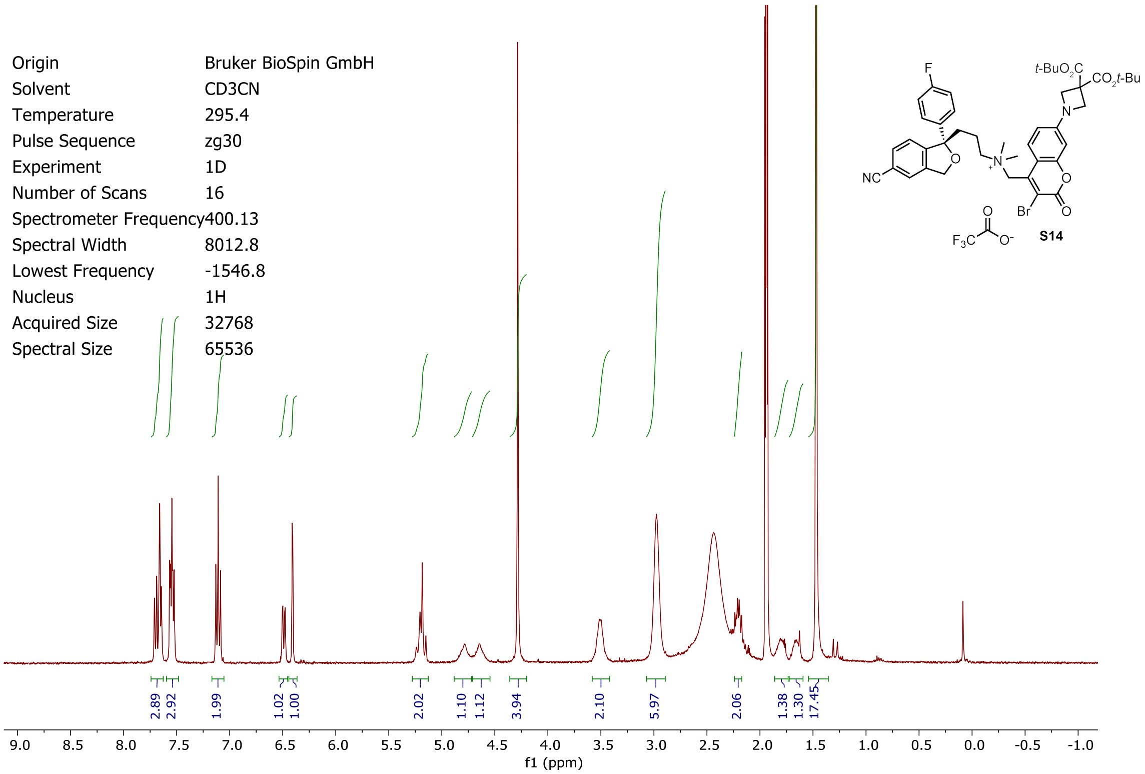
F
t-BuO2C
CO2t-Bu
N
NC
O
N+
O
Br
O
O
F3C
O-
Origin Bruker BioSpin GmbH
Solvent CD3CN
Temperature 295.4
Pulse Sequence zg30
Experiment 1D
Number of Scans 16
Spectrometer Frequency 400.13
Spectral Width 8012.8
Lowest Frequency -1546.8
Nucleus 1H
Acquired Size 32768
Spectral Size 65536
2.89
2.92
1.99
1.02
1.00
2.02
1.10
1.12
3.94
2.10
5.97
2.06
1.38
1.30
17.45
9.0 8.5 8.0 7.5 7.0 6.5 6.0 5.5 5.0 4.5 4.0 3.5 3.0 2.5 2.0 1.5 1.0 0.5 0.0 -0.5 -1.0
f1 (ppm)

S14

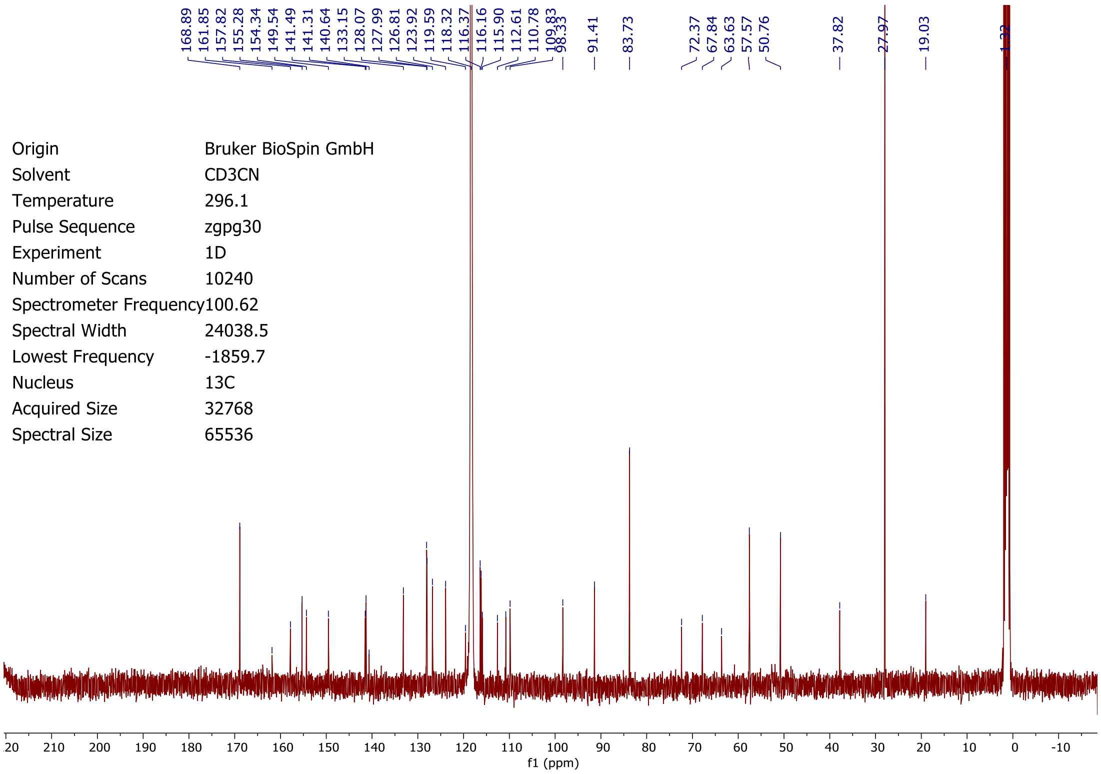
168.89
161.85
157.82
155.28
154.34
149.54
141.49
141.31
140.64
133.15
128.07
127.99
126.81
123.92
119.59
118.32
116.37
116.16
115.90
112.61
110.78
109.83
98.33
91.41
83.73
72.37
67.84
63.63
57.57
50.76
37.82
27.97
19.03
1.32
Origin Bruker BioSpin GmbH
Solvent CD3CN
Temperature 296.1
Pulse Sequence zgpg30
Experiment 1D
Number of Scans 10240
Spectrometer Frequency 100.62
Spectral Width 24038.5
Lowest Frequency -1859.7
Nucleus 13C
Acquired Size 32768
Spectral Size 65536
20 210 200 190 180 170 160 150 140 130 120 110 100 90 80 70 60 50 40 30 20 10 0 -10
f1 (ppm)

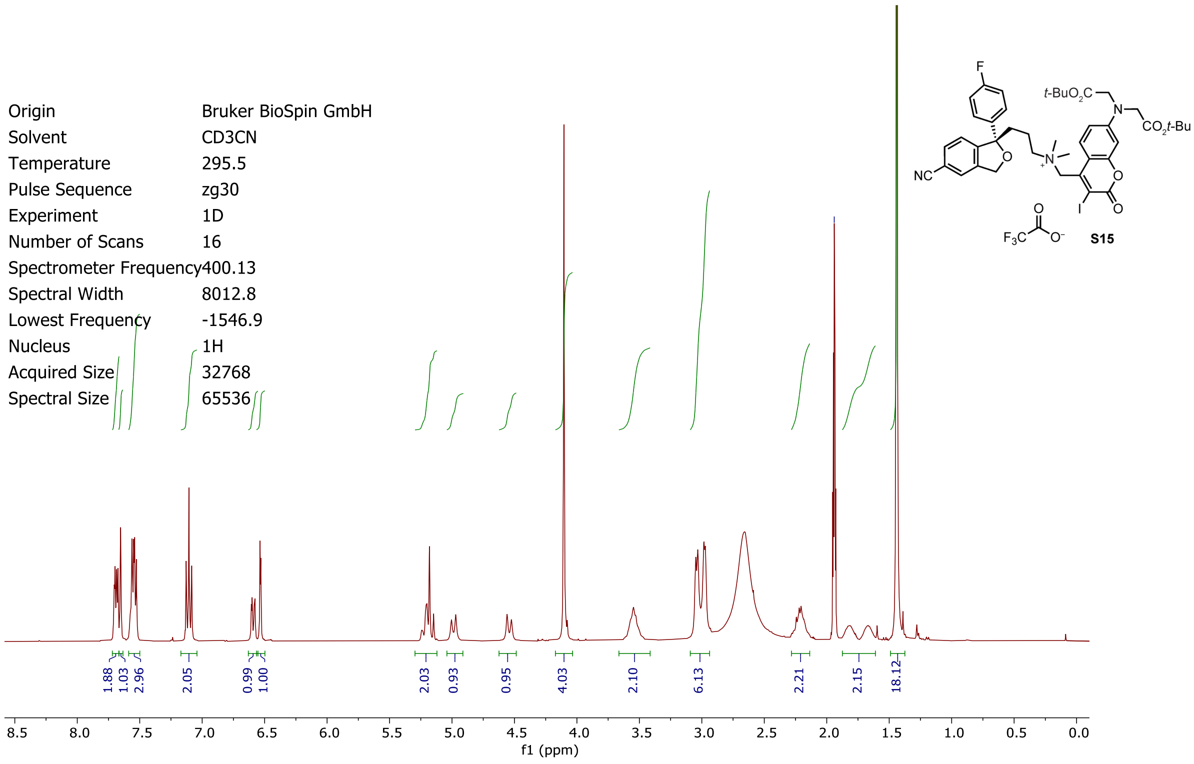

Origin Bruker BioSpin GmbH
Solvent CD3CN
Temperature 295.5
Pulse Sequence zg30
Experiment 1D
Number of Scans 16
Spectrometer Frequency 400.13
Spectral Width 8012.8
Lowest Frequency -1546.9
Nucleus 1H
Acquired Size 32768
Spectral Size 65536
1.88
1.03
2.96
2.05
0.99
1.00
2.03
0.93
0.95
4.03
2.10
6.13
2.21
2.15
18.12
8.5 8.0 7.5 7.0 6.5 6.0 5.5 5.0 4.5 4.0 3.5 3.0 2.5 2.0 1.5 1.0 0.5 0.0
f1 (ppm)
F
t-BuO2C
N
CO2t-Bu
NC
O
N+
O
O
I
O
F3C
O−

S15

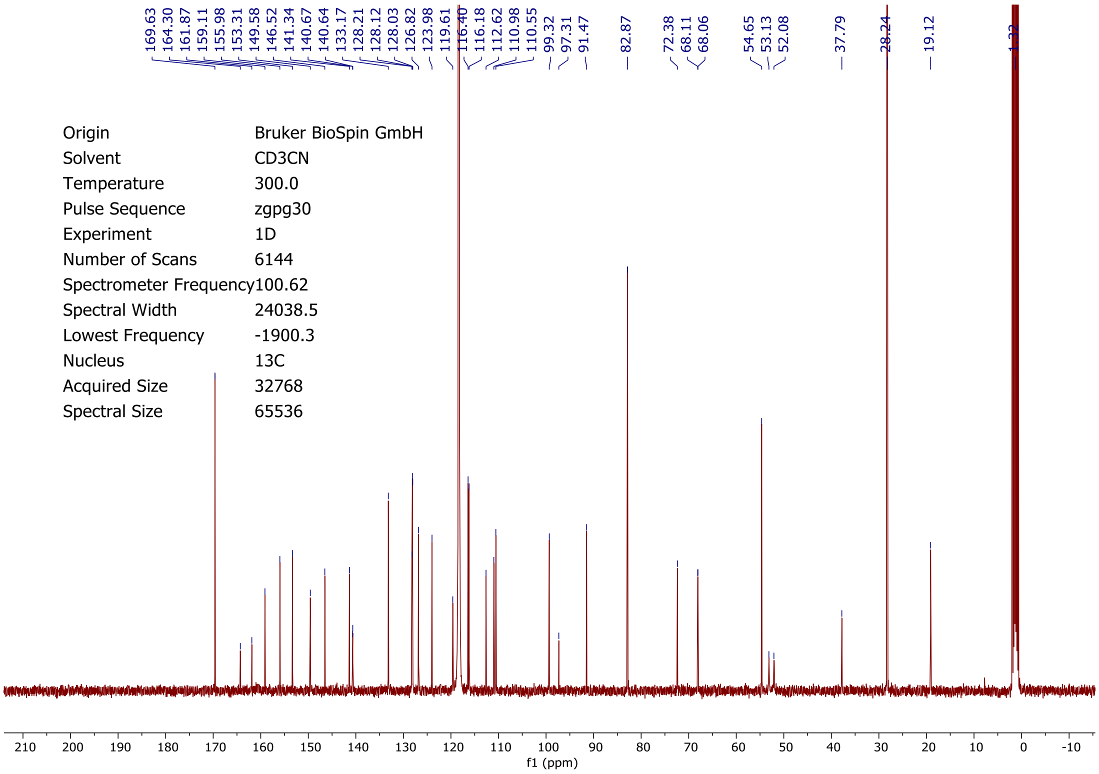

169.63
164.30
161.87
159.11
155.98
153.31
149.58
146.52
141.34
140.67
140.64
133.17
128.21
128.12
128.03
126.82
123.98
119.61
116.40
116.18
112.62
110.98
110.55
99.32
97.31
91.47
82.87
72.38
68.11
68.06
54.65
53.13
52.08
37.79
28.24
19.12
Origin Bruker BioSpin GmbH
Solvent CD3CN
Temperature 300.0
Pulse Sequence zgpg30
Experiment 1D
Number of Scans 6144
Spectrometer Frequency 100.62
Spectral Width 24038.5
Lowest Frequency -1900.3
Nucleus 13C
Acquired Size 32768
Spectral Size 65536
210 200 190 180 170 160 150 140 130 120 110 100 90 80 70 60 50 40 30 20 10 0 -10
f1 (ppm)

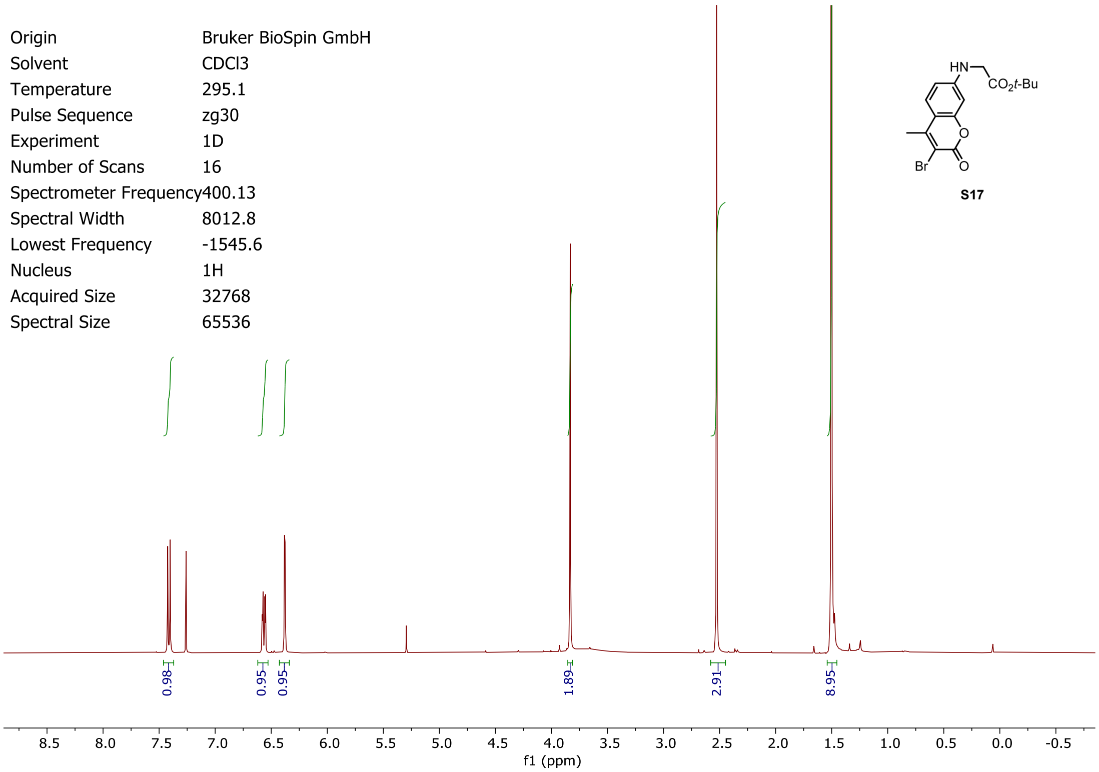

HN
CO_2t-Bu
O
O
Br
S17
Origin Bruker BioSpin GmbH
Solvent CDCl3
Temperature 295.1
Pulse Sequence zg30
Experiment 1D
Number of Scans 16
Spectrometer Frequency 400.13
Spectral Width 8012.8
Lowest Frequency -1545.6
Nucleus 1H
Acquired Size 32768
Spectral Size 65536
0.98
0.95
0.95
1.89
2.91
8.95
8.5 8.0 7.5 7.0 6.5 6.0 5.5 5.0 4.5 4.0 3.5 3.0 2.5 2.0 1.5 1.0 0.5 0.0 -0.5
f1 (ppm)

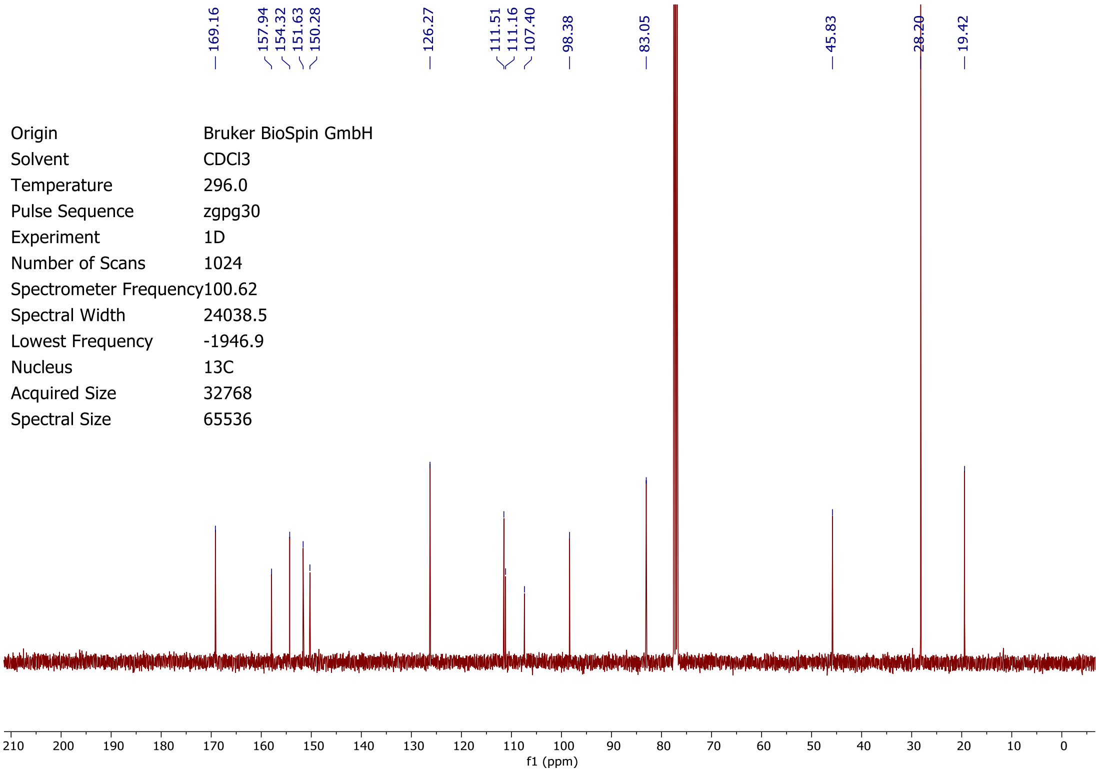

169.16
157.94
154.32
151.63
150.28
126.27
111.51
111.16
107.40
98.38
83.05
45.83
28.20
19.42
Origin Bruker BioSpin GmbH
Solvent CDCl3
Temperature 296.0
Pulse Sequence zgpg30
Experiment 1D
Number of Scans 1024
Spectrometer Frequency 100.62
Spectral Width 24038.5
Lowest Frequency -1946.9
Nucleus 13C
Acquired Size 32768
Spectral Size 65536
210 200 190 180 170 160 150 140 130 120 110 100 90 80 70 60 50 40 30 20 10 0
f1 (ppm)

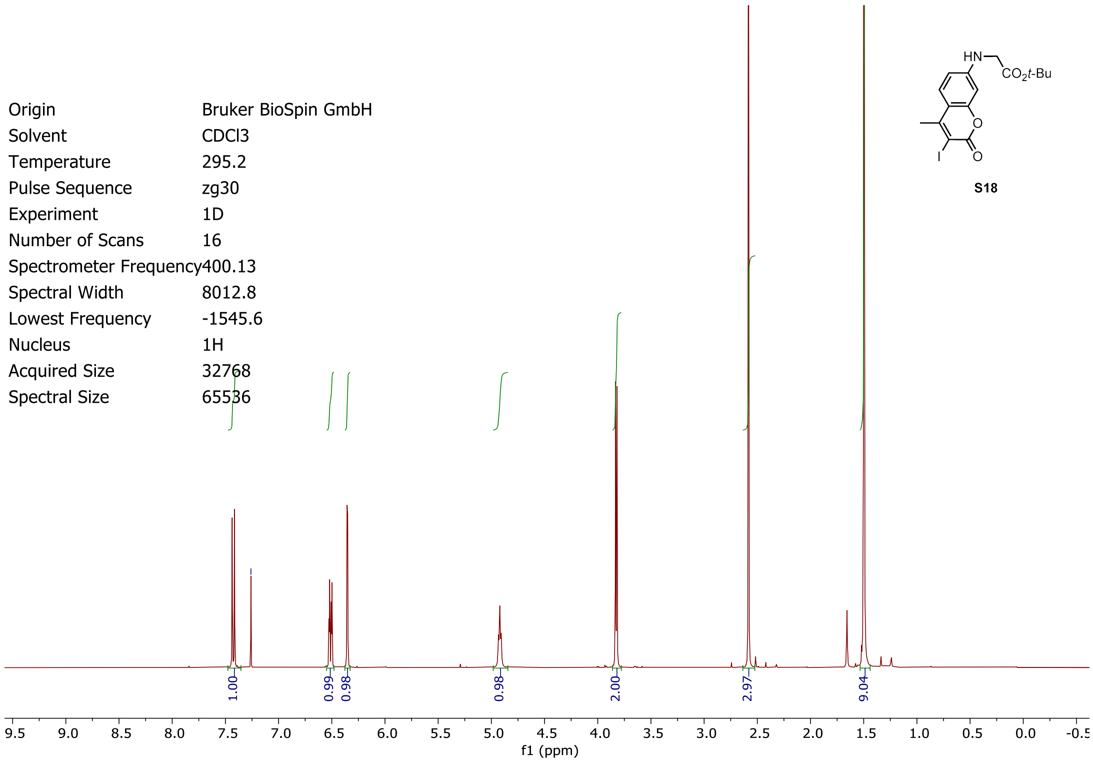

Origin Bruker BioSpin GmbH
Solvent CDCl3
Temperature 295.2
Pulse Sequence zg30
Experiment 1D
Number of Scans 16
Spectrometer Frequency 400.13
Spectral Width 8012.8
Lowest Frequency -1545.6
Nucleus 1H
Acquired Size 32768
Spectral Size 65536
HN
CO_2t-Bu
O
O
I
S18
1.00
0.99
0.98
0.98
2.00
2.97
9.04
f1 (ppm)

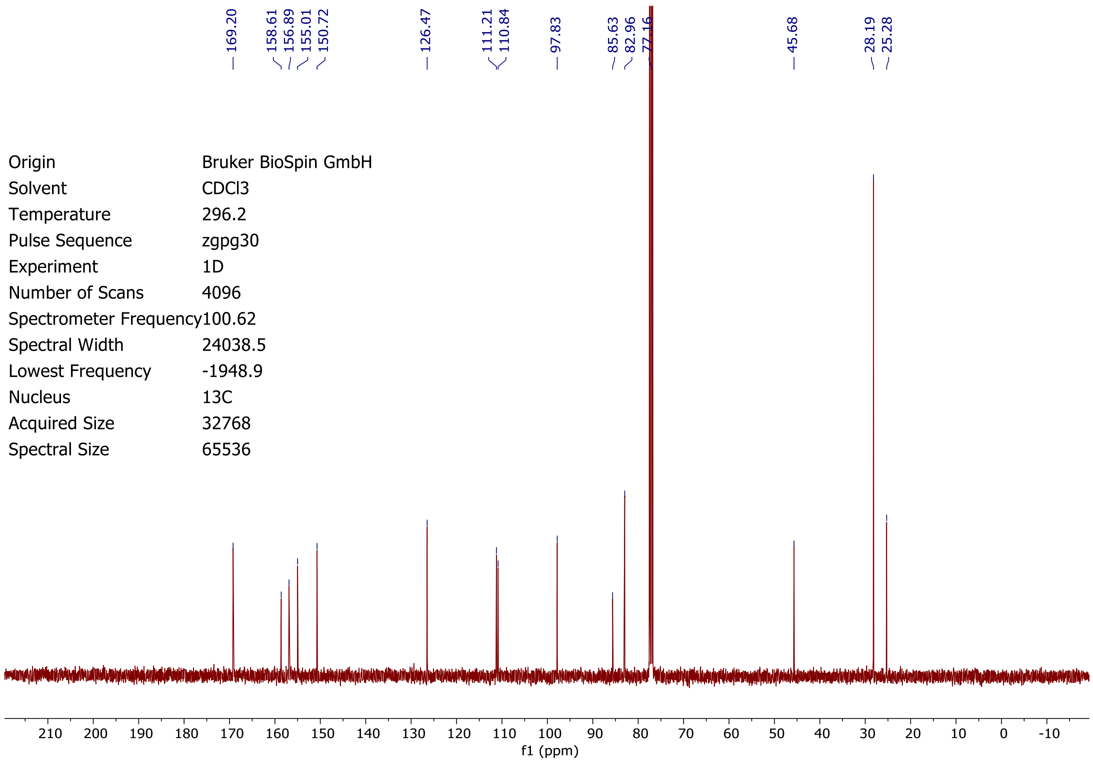

169.20
158.61
156.89
155.01
150.72
126.47
111.21
110.84
97.83
85.63
82.96
77.16
45.68
28.19
25.28
Origin Bruker BioSpin GmbH
Solvent CDCl3
Temperature 296.2
Pulse Sequence zgpg30
Experiment 1D
Number of Scans 4096
Spectrometer Frequency 100.62
Spectral Width 24038.5
Lowest Frequency -1948.9
Nucleus 13C
Acquired Size 32768
Spectral Size 65536
f1 (ppm)

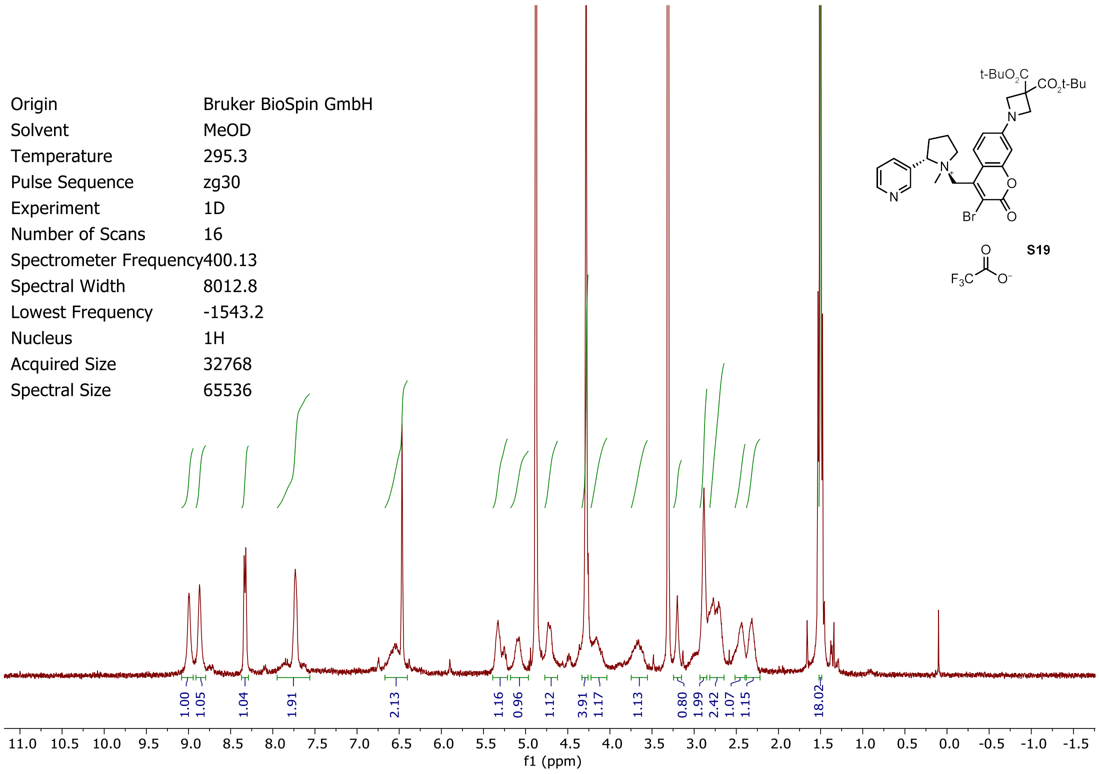
Origin Bruker BioSpin GmbH
Solvent MeOD
Temperature 295.3
Pulse Sequence zg30
Experiment 1D
Number of Scans 16
Spectrometer Frequency 400.13
Spectral Width 8012.8
Lowest Frequency -1543.2
Nucleus 1H
Acquired Size 32768
Spectral Size 65536
1.00
1.05
1.04
1.91
2.13
1.16
0.96
1.12
3.91
1.17
1.13
0.80
1.99
2.42
1.07
1.15
18.02
11.0 10.5 10.0 9.5 9.0 8.5 8.0 7.5 7.0 6.5 6.0 5.5 5.0 4.5 4.0 3.5 3.0 2.5 2.0 1.5 1.0 0.5 0.0 -0.5 -1.0 -1.5
f1 (ppm)
t-BuO2C
CO2t-Bu
N
N
N+
O
O
Br
O
F3C
O-

S19

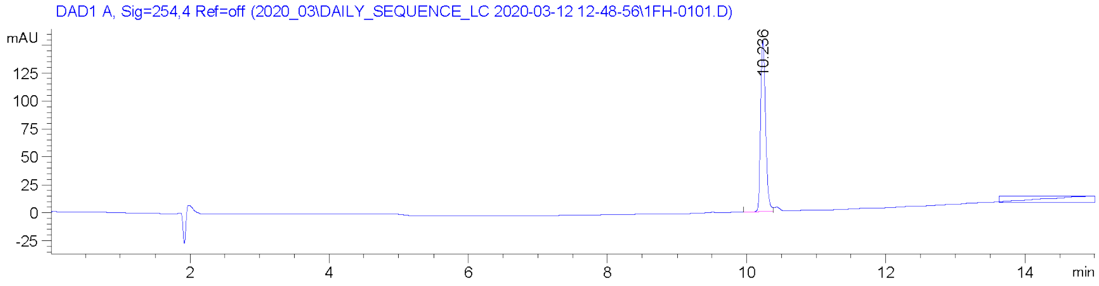
DAD1 A, Sig=254,4 Ref=off (2020_03\DAILY_SEQUENCE_LC 2020-03-12 12-48-56\1FH-0101.D)
mAU
125
100
75
50
25
0
-25
10.236
2
4
6
8
10
12
14
min

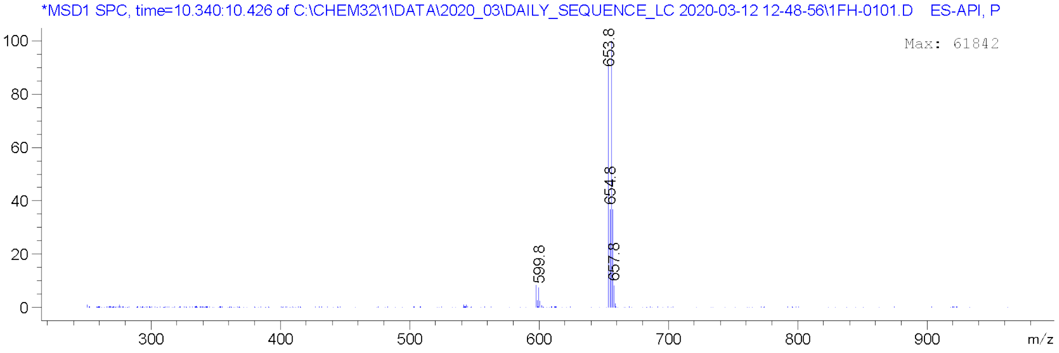
*MSD1 SPC, time=10.340:10.426 of C:\CHEM32\1\DATA\2020_03\DAILY_SEQUENCE_LC 2020-03-12 12-48-56\1FH-0101.D ES-API, P
Max: 61842
100
80
60
40
20
0
653.8
654.8
599.8
657.8
300
400
500
600
700
800
900
m/z

Origin	Bruker BioSpin GmbH
Solvent	CD3CN
Temperature	295.4
Pulse Sequence	zg30
Experiment	1D
Number of Scans	16
Spectrometer Frequency	400.13
Spectral Width	8012.8
Lowest Frequency	-1546.8
Nucleus	1H
Acquired Size	32768
Spectral Size	65536

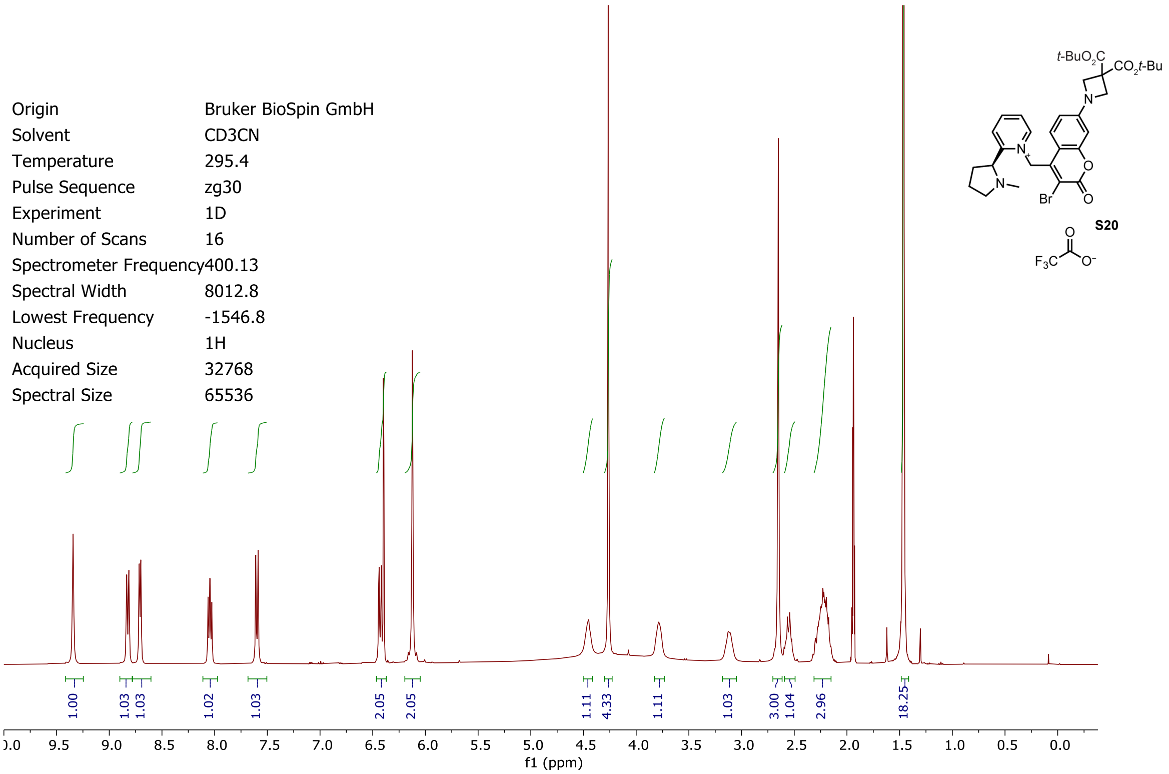

Origin	Bruker BioSpin GmbH
Solvent	CD3CN
Temperature	296.2
Pulse Sequence	zgpg30
Experiment	1D
Number of Scans	512
Spectrometer Frequency	100.62
Spectral Width	24038.5
Lowest Frequency	-1860.0
Nucleus	13C
Acquired Size	32768
Spectral Size	65536

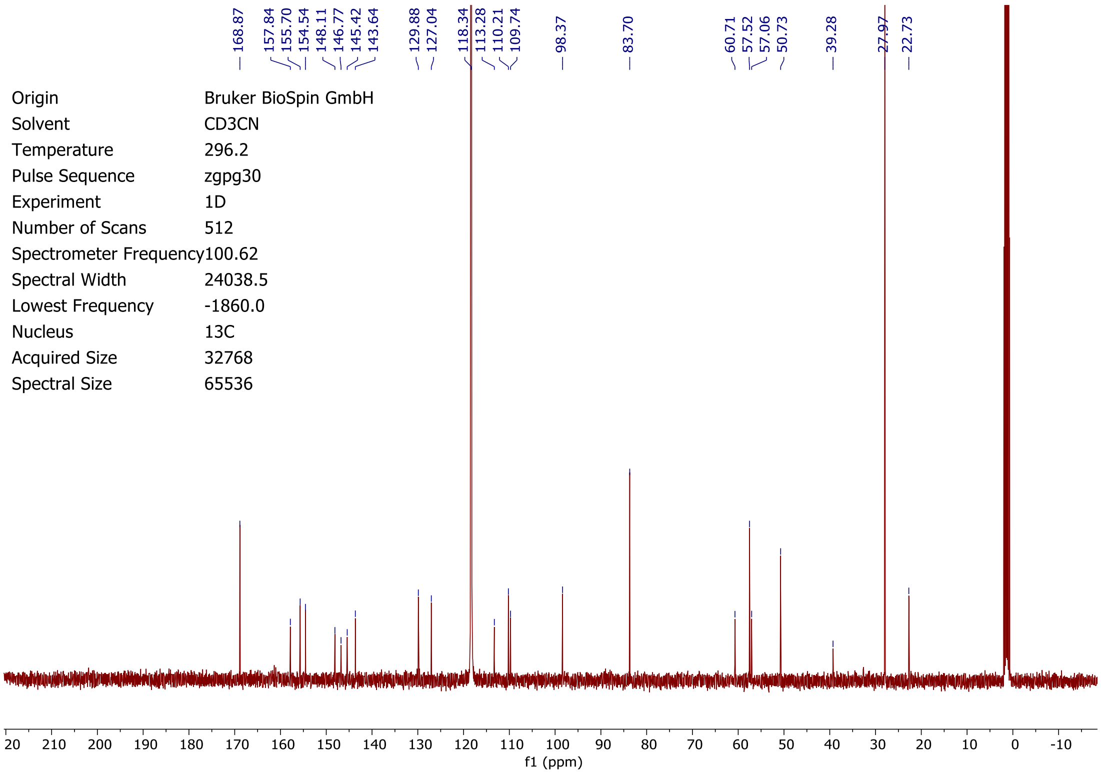

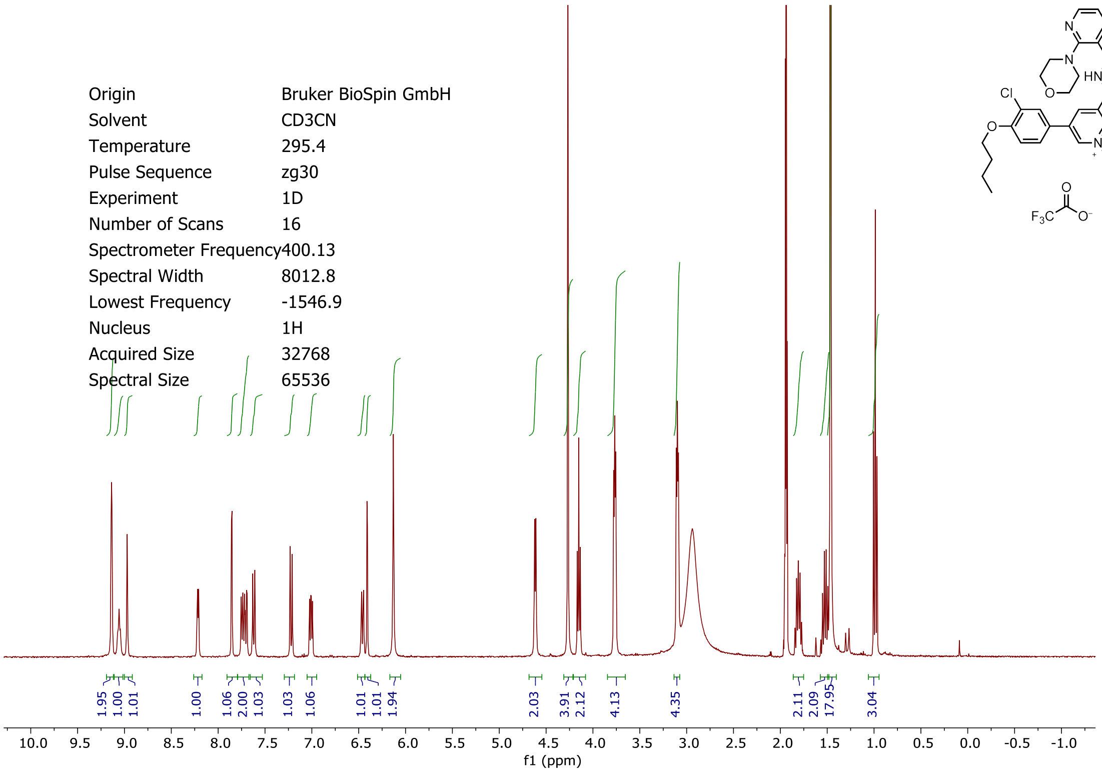
Origin Bruker BioSpin GmbH
Solvent CD3CN
Temperature 295.4
Pulse Sequence zg30
Experiment 1D
Number of Scans 16
Spectrometer Frequency 400.13
Spectral Width 8012.8
Lowest Frequency -1546.9
Nucleus 1H
Acquired Size 32768
Spectral Size 65536
1.95 1.00 1.01 1.00 1.06 2.00 1.03 1.03 1.06 1.01 1.01 1.94 2.03 3.91 2.12 4.13 4.35 2.11 2.09 17.95 3.04
10.0 9.5 9.0 8.5 8.0 7.5 7.0 6.5 6.0 5.5 5.0 4.5 4.0 3.5 3.0 2.5 2.0 1.5 1.0 0.5 0.0 -0.5 -1.0
f1 (ppm)

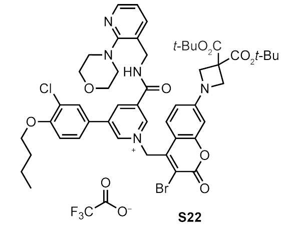
t-BuO2C
CO2t-Bu
N
HN
O
Cl
O
N
O
Br
O
F3C
O-

S22

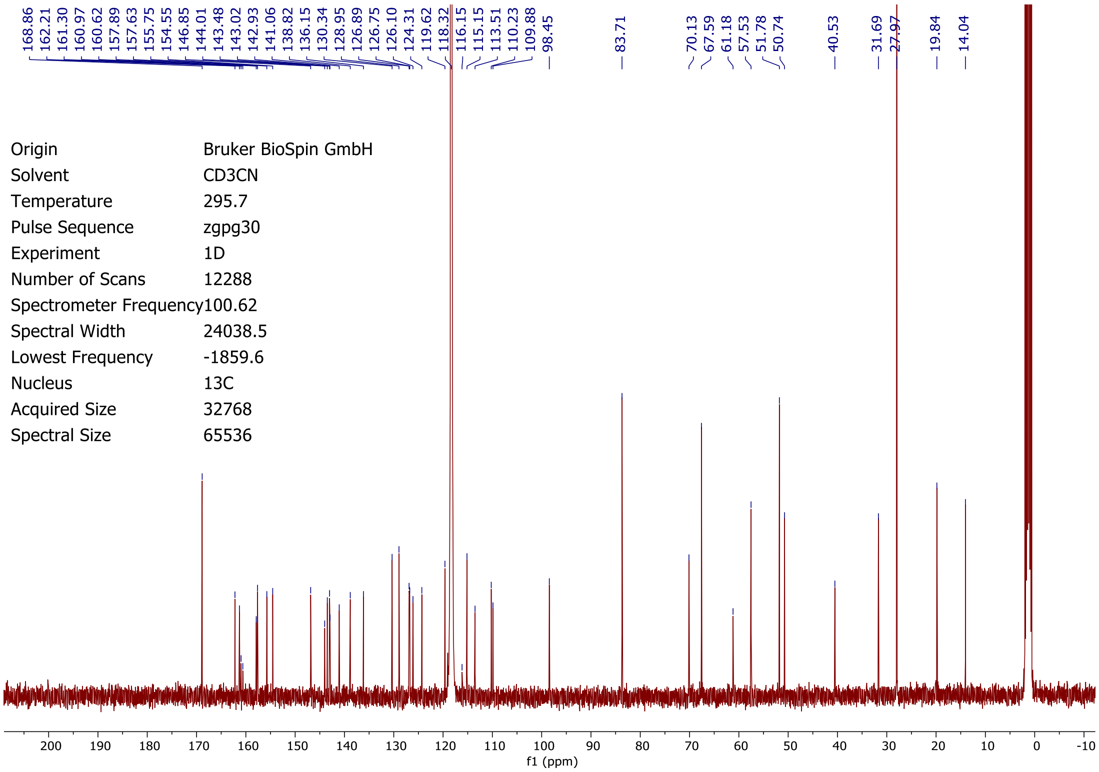
168.86 162.21 161.30 160.97 160.62 157.89 157.63 155.75 154.55 146.85 144.01 143.48 143.02 142.93 141.06 138.82 136.15 130.34 128.95 126.89 126.75 126.10 124.31 119.62 118.32 116.15 115.15 113.51 110.23 109.88 98.45 83.71 70.13 67.59 61.18 57.53 51.78 50.74 40.53 31.69 27.97 19.84 14.04
Origin Bruker BioSpin GmbH
Solvent CD3CN
Temperature 295.7
Pulse Sequence zgpg30
Experiment 1D
Number of Scans 12288
Spectrometer Frequency 100.62
Spectral Width 24038.5
Lowest Frequency -1859.6
Nucleus 13C
Acquired Size 32768
Spectral Size 65536
200 190 180 170 160 150 140 130 120 110 100 90 80 70 60 50 40 30 20 10 0 -10
f1 (ppm)

Origin	Bruker BioSpin GmbH
Solvent	DMSO
Temperature	300.0
Pulse Sequence	zg30
Experiment	1D
Number of Scans	128
Spectrometer Frequency	400.13
Spectral Width	8012.8
Lowest Frequency	-1538.7
Nucleus	1H
Acquired Size	32768
Spectral Size	65536

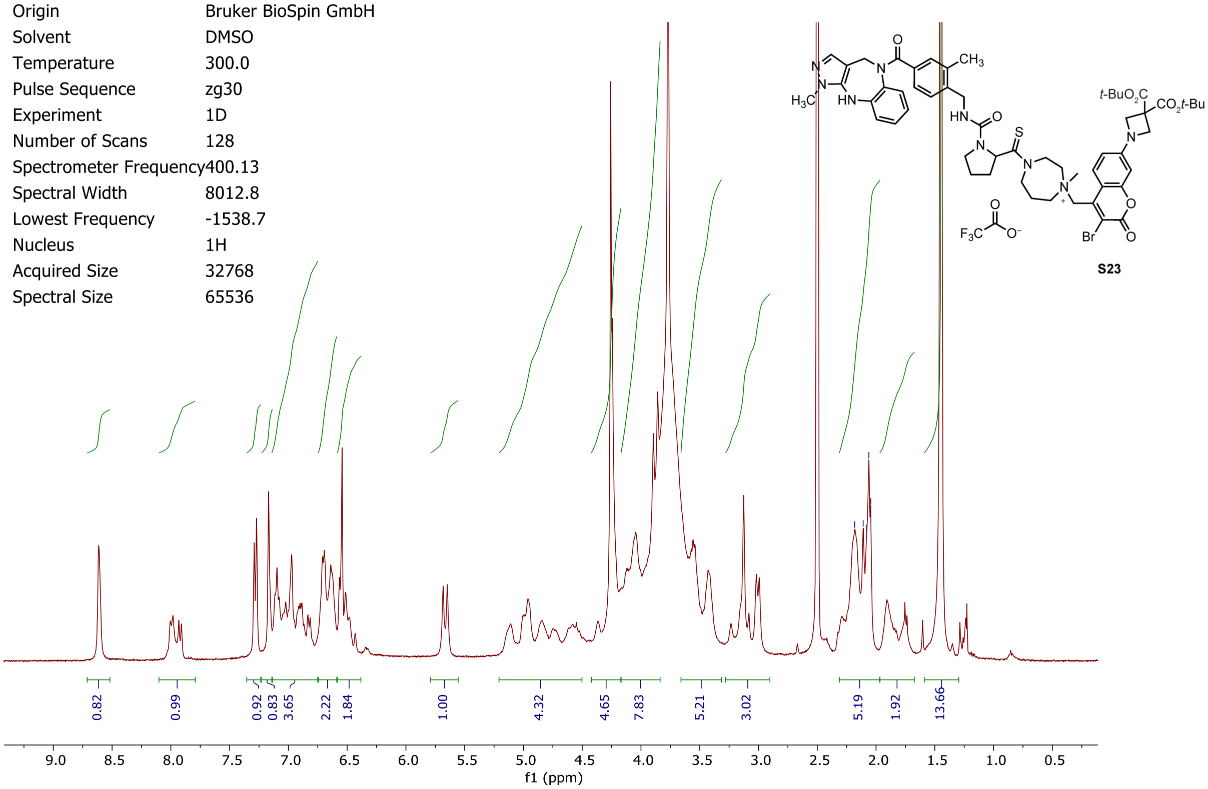

S23

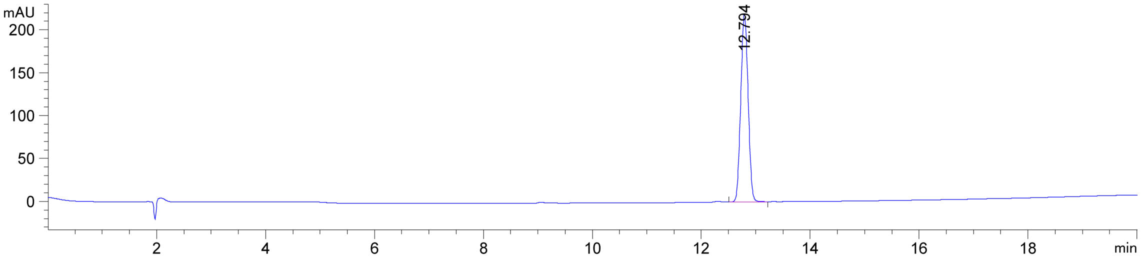

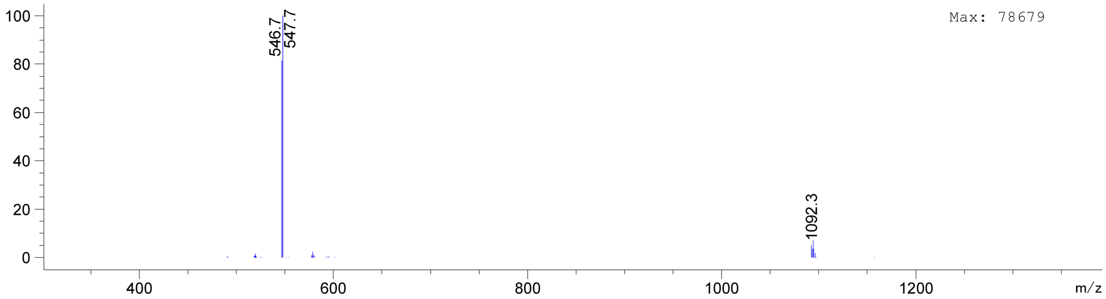

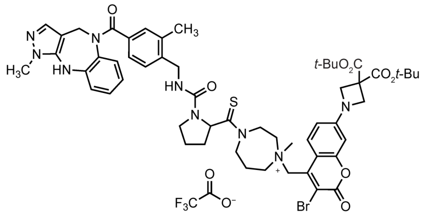

S23

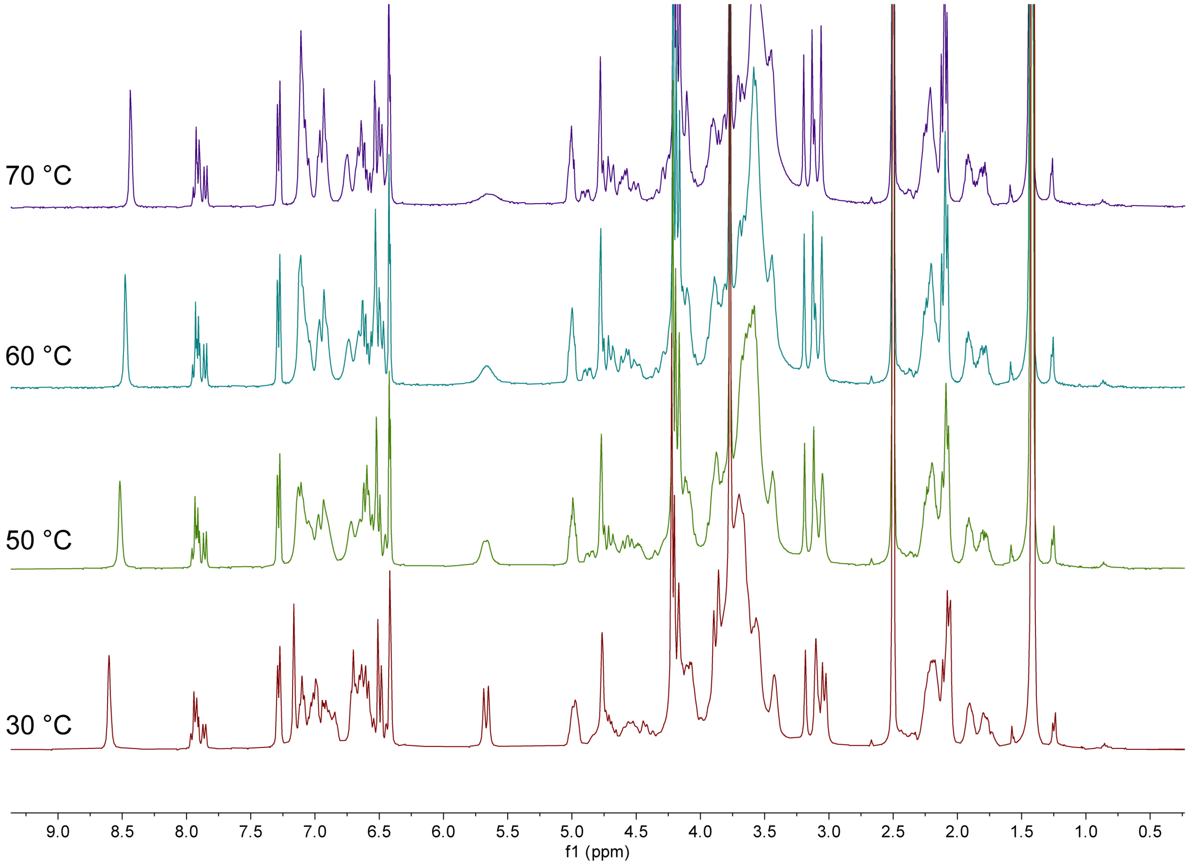

^{1}H-NMR of compound **S23** in DMSO-d_6 at different temperatures.

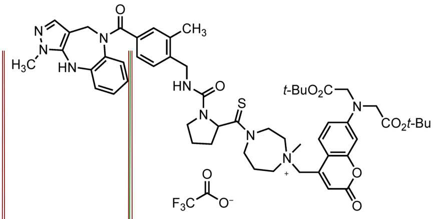

S25

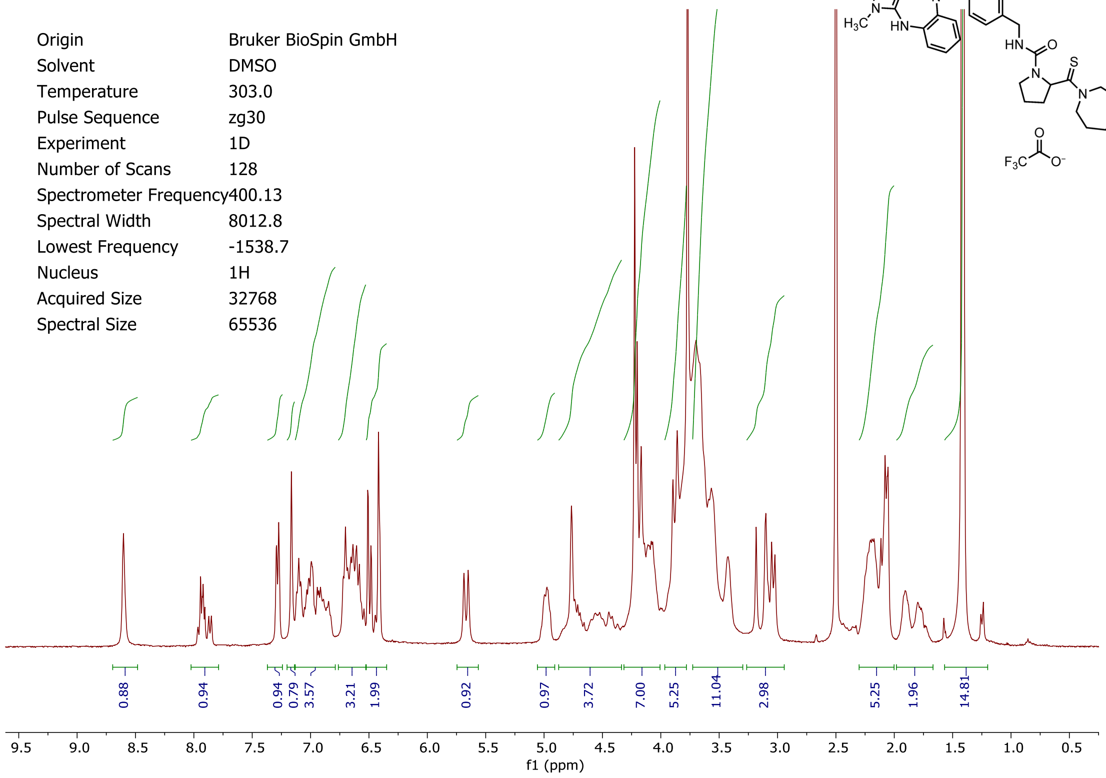
Origin Bruker BioSpin GmbH
Solvent DMSO
Temperature 303.0
Pulse Sequence zg30
Experiment 1D
Number of Scans 128
Spectrometer Frequency 400.13
Spectral Width 8012.8
Lowest Frequency -1538.7
Nucleus 1H
Acquired Size 32768
Spectral Size 65536
0.88
0.94
0.94
0.79
3.57
3.21
1.99
0.92
0.97
3.72
7.00
5.25
11.04
2.98
5.25
1.96
14.81
f1 (ppm)

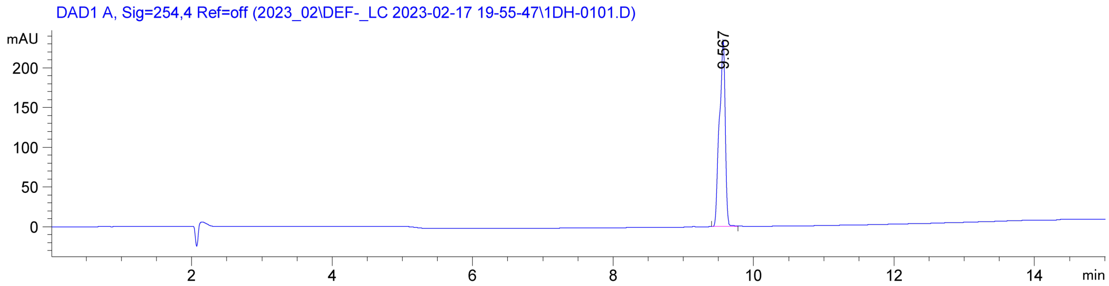
DAD1 A, Sig=254,4 Ref=off (2023_02\DEF-_LC 2023-02-17 19-55-47\1DH-0101.D)
mAU
9.567
min

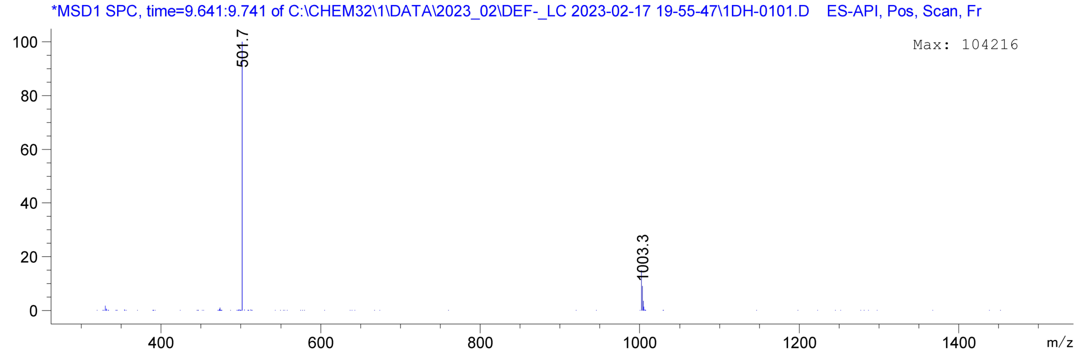
*MSD1 SPC, time=9.641:9.741 of C:\CHEM32\1\DATA\2023_02\DEF-_LC 2023-02-17 19-55-47\1DH-0101.D ES-API, Pos, Scan, Fr
Max: 104216
501.7
1003.3
m/z

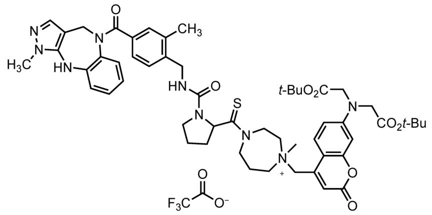

S25

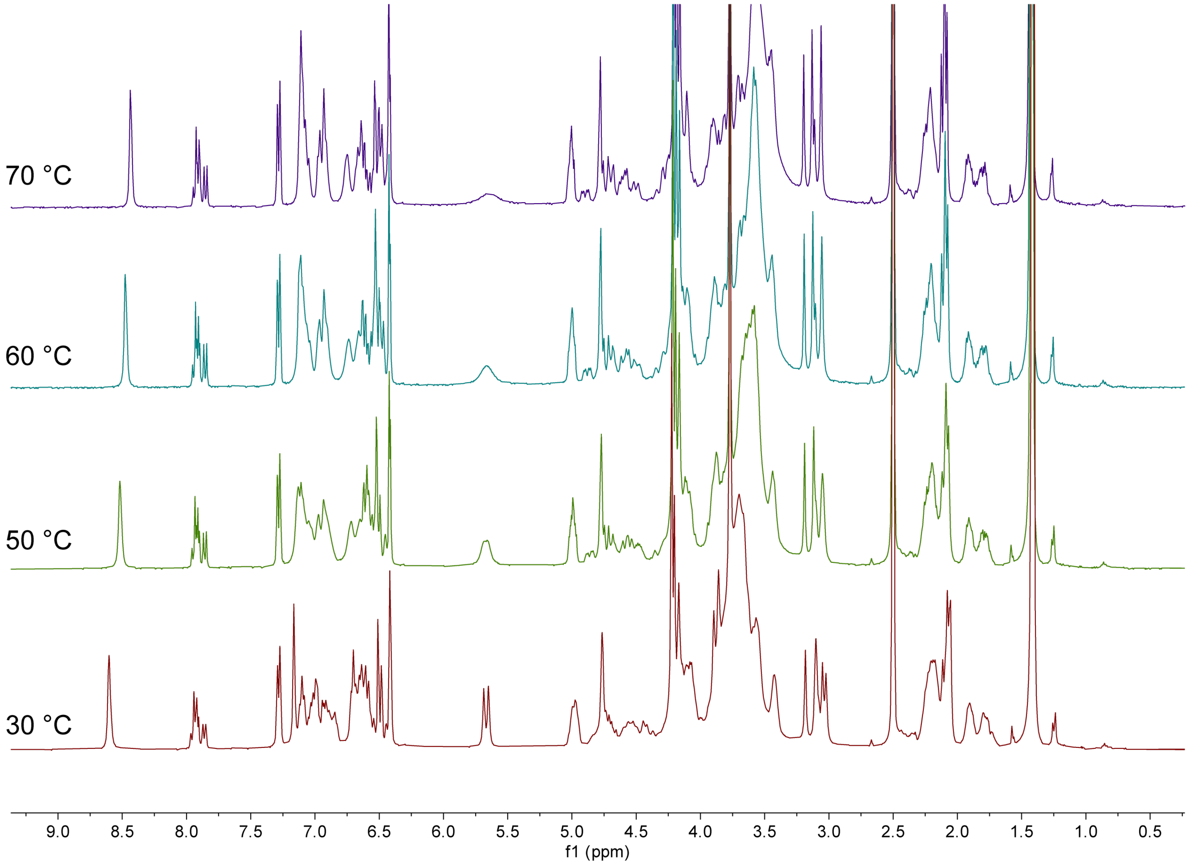

^{1}H-NMR of compound **S25** in DMSO-d_6 at different temperatures.

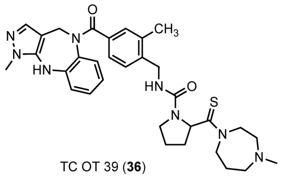

TC OT 39 (**36**)

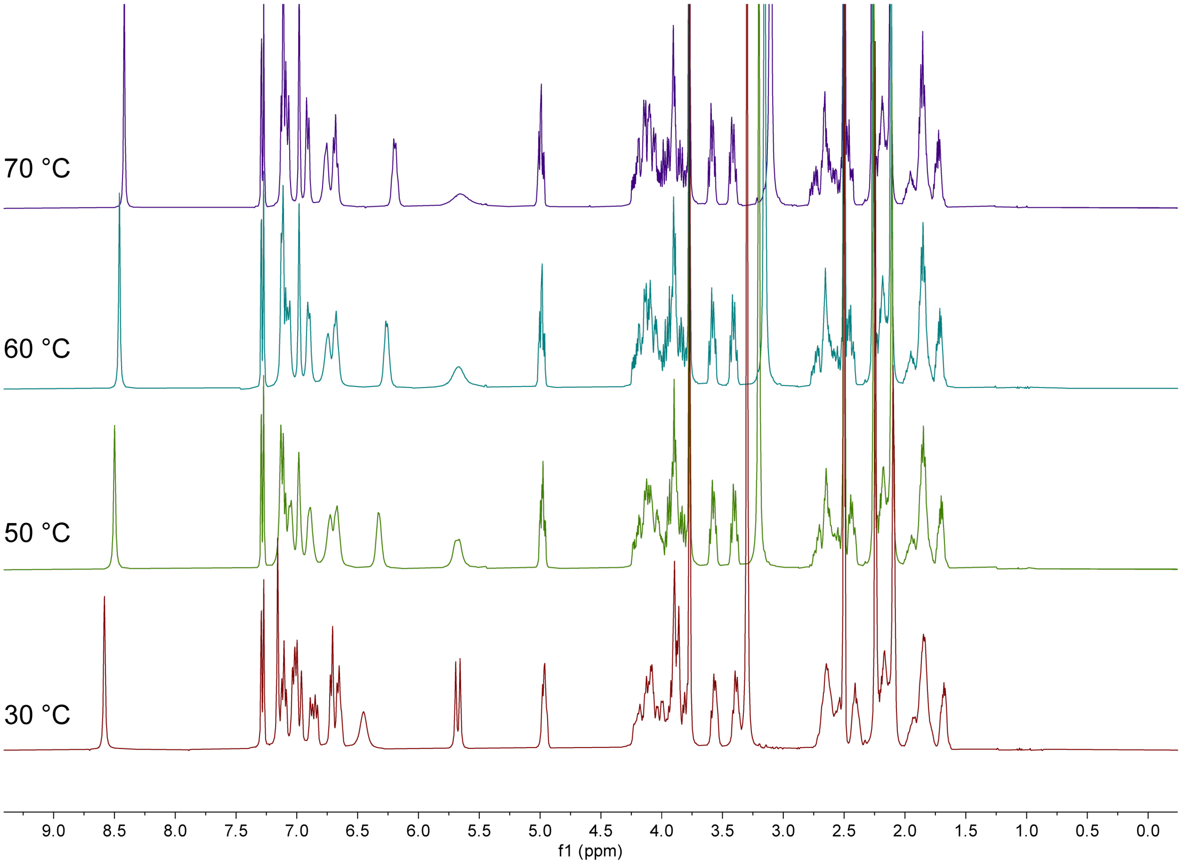

^{1}H-NMR of compound **36**, used for synthesis of **37** and **38**, in DMSO-d_6 at different temperatures.
